# Interplay of neuronal and non-neuronal genes regulates intestinal DAF-16-mediated immune response during *Fusarium* infection of *Caenorhabditis elegans*

**DOI:** 10.1038/cddiscovery.2017.73

**Published:** 2017-11-13

**Authors:** Papri Nag, Pooja Rani Aggarwal, Sudip Ghosh, Kanika Narula, Rajul Tayal, Nidhi Maheshwari, Niranjan Chakraborty, Subhra Chakraborty

**Affiliations:** 1 National Institute of Plant Genome Research, Aruna Asaf Ali Marg, New Delhi, India

## Abstract

Although precisely controlled innate immune response is governed by conserved cellular events in phylogenetically diverse hosts, the underlying molecular mechanisms by which this process is regulated against a multi-host pathogen remain unknown. *Fusarium oxysporum* is a model multi-host pathogen, known to be associated with neuronal stress in humans and vascular wilt in plants. The interaction between innate immune and neuronal pathways is the basis of many diverse biological responses. How these processes are coordinated in response to fungal disease is not well understood. Here, we show that *F. oxysporum* f. sp*. ciceri* causes neuronal stress and intestinal disintegration, ultimately leading to the death of *Caenorhabditis elegans*. To explore the regulatory framework of *Fusarium-*associated disease, we analysed the gene expression during infection, integrated temporal gene expression, and network analysis with genetic inactivation data in *Caenorhabditis elegans.* We identified 1024 genes showing significant changes in expression (corrected *P*-values <0.05) in response to *Fusarium* infection. Co-expression network analysis of our data identified prognostic genes related to disease progression. These genes were dynamically expressed in various neuronal and non-neuronal tissues exhibiting diverse biological functions, including cellular homeostasis, organ patterning, stress response, and lipid metabolism. The RNA-seq analysis further identified shared and unique signalling pathways regulated by DAF-16/FOXO and SIR-2.1 linking neuronal stress, which facilitates negative regulation of intestinal innate immunity. Genetic analysis revealed that GCY-5 in ASE functions upstream of DAF-16, whereas ASI-specific SRD-1 regulates behavioural immunity. Overall, our results indicate that a ubiquitous response occurs during *Fusarium* infection mediated by highly conserved regulatory components and pathways, which can be exploited further for the identification of disease-responsive genes conserved among animals and plants. Finally, this study provided a novel insight into cross-species immune signalling and may facilitate the discovery of cellular therapeutic targets for *Fusarium*-associated disease.

## Introduction

Morbidity and mortality associated with fungal infections and emergence of resistant fungal strains necessitate the study of fungal pathogenesis and host innate immunity. Evidences suggest that a common virulence mechanism exists for a wide array of pathogenic fungi with broad host ranges.^1^ Several pathogens, including *Fusarium,* have the ability to infect both animals and plants.^2^ It has emerged as the second most frequent mould causing invasive fungal infection in humans and exhibits a broad resistance to antifungal drugs.^3,4^ It acts as an opportunistic invader causing allergies, sinusitis, and pulmonary infections in immunocompromised and immunocompetent patients.^4^ *Fusarium*-induced neuronal stress and mycotoxicosis are considered as potential risk factors in humans and rats.^5,6^ In plants, it causes vascular wilt, head blight, root rot, seedling blight, and foot rot diseases,^7–9^ while *Fusarium-*mediated killing of *Caenorhabditis elegans* has recently been described.^10^ Previously, cross-kingdom pathogenicity of *F. oxysporum* f. sp*. lycopersici* was investigated in mice to unravel the disease mechanism in plants and mammals.^11^ Growing evidence indicates that Rho1, a signal component is indispensable for the virulence of *F. oxysporum* in plants, but not in mammalian hosts.^12^ In these reports, *Fusarium* pathogenicity in different host systems has been shown; however, which pathways might serve as the functional basis of *Fusarium*-associated disease or an immune state remains to be explored.

Mechanistic frameworks of defense and disease state accommodate certain common and contrasting themes that regulate host-specific pathogen surveillance system in eukaryotes.^13^ Innate immunity cannot be considered as autonomous. Increasing evidence suggests that actuation of the immune system is coordinated with the nervous system to regulate defense responses, as it perceives and responds to various pathogens in mammals.^14^ Their complexity led us to study how these systems influence each other at the molecular and cellular level in a well-characterised model organism *Caenorhabditis elegans*. Characterisation of nematode immunity is largely based on nosocomial bacterial pathogens.^15^ However, immune response directed towards a medically and agronomically important fungal pathogen, such as *F. oxysporum* has not been defined till date. Recent studies indicate that sensory and dopaminergic neurons regulate innate immune pathways in *C. elegans*.^16,17^ Furthermore, the regulation of DAF-16-mediated innate immunity in bacteria is well explored in worms.^18^ Epidermal DAF-16 is known to be involved in immunity against *Drechmeria coniospora*;^19^ however, the role of intestinal DAF-16 in fungal infection is yet unknown.

Although these studies provide targeted information associated with immune pathways, a global overview of gene expression and function in a spatiotemporal manner defining organ specificity and pathway conservation across kingdoms due to fungal invasion is lacking. Studies on transcriptional variations have been widely used to analyse inter-kingdom differences and dissect changes in regulatory sequences and expression divergence among them.^15^ In addition, signal transduction defines functional homology, and genetic screens offer the detection of candidate genes involved in immune system programming.^15^

Here, we employed integrated transcriptomic, genetic analysis, and a system-level approach to understand molecular parsimony associated with neuro-immune pathways. Using RNA-seq analysis, we created a transcriptional landscape of *C. elegans* invaded with *Fusarium* that exhibits an interconnected cascade of DAF-16- and SIR-2.1-regulated genes linking neuronal stress and immunity. We then constructed a correlation network and assessed the biological significance of modules focussing on disease-/immunity-related genes. Organ-based network shows a distinct disease/immune signatures for specific organs. Using a genetic screen, we observed that intestinal DAF-16 is mainly responsive to *F. oxysporum* infection. Altogether, our study demonstrates the ability of a fungus to induce neuronal stress and trigger a non-canonical pathway, which regulates pathogen-induced immune response through avoidance and the activation of several novel immune-responsive genes. In addition, the approach would be applicable to identify the analogous pathways of defense response and its regulation across kingdoms.

## Results

### *F. oxysporum* infection leads to intestinal disintegration in *C. elegans*

**Figure 1:**
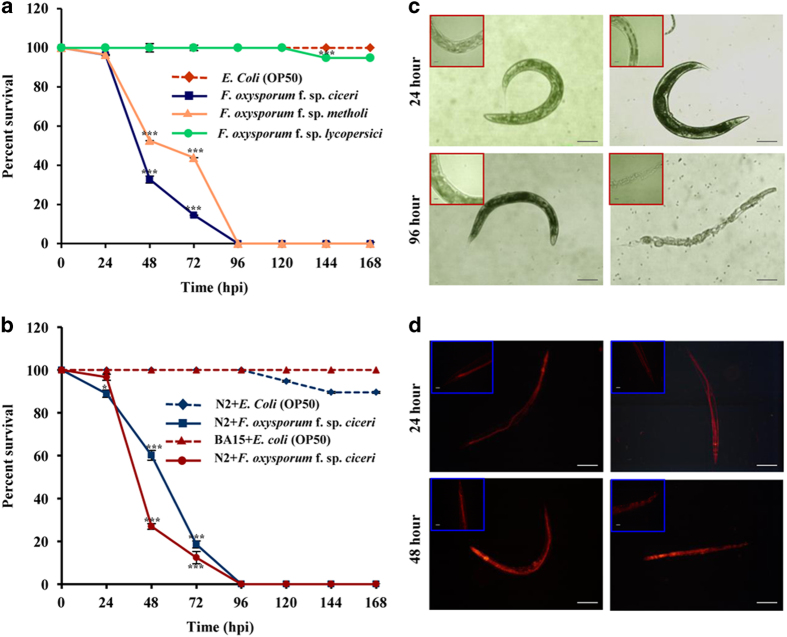
*F. oxysporum*-mediated intestinal disintegrity causes killing of BA15. (**a**) BA15 worms [*rrf-3(hc15)*] were exposed to different strains of *F. oxysporum. n*=90 adult worms for each strain (*P*<0.0001). (**b**) BA15 worms were exposed to *E. coli* and *F. oxysporum* under non-avoidance conditions*. n*=90 adult worms for each strain (*P*<0.0001). Error bars represent S.E. from three independent experiments. *P<0.05, and ***P<0.001 by one-way ANOVA and Tukey’s *post hoc* test. *P*-values are relative to *E. coli*-fed worms. (**c**) BA15 [*rrf-3 (hc15)*] was exposed to *E. coli* and *F. oxysporum* under non-avoidance conditions for 24 and 96 h and then visualised using a Nikon 80i DIC microscope. (**d**) Propidium iodide staining of wild-type worms of N2 exposed to *F. oxysporum*. The worms were fed with either *F. oxysporum* or *E. coli* OP50 for 48 h and then visualised using a Nikon 80i fluorescence microscope. Scale bars represent 10 *μ*m.

To establish potential *C. elegans*–*F. oxysporum* pathosystem, we screened *F. oxysporum* f. sp*. ciceri*, *F. oxysporum* f. sp*. methioli*, and *F. oxysporum* f. sp*. lycopersici* for worm survivability. Worms showed a high susceptibility to *F. oxysporum* f. sp*. ciceri*, as compared to *F. oxysporum* f. sp*. methioli*. However, survivability of the worms grown on *F. oxysporum* f. sp*. lycopersici* was comparable to control worms grown on *Escherichia coli* OP50 (Figure 1a). Although *F. oxysporum* is known to infect plants in a host-specific manner,^8^ we found that both wild-type N2 and BA15 (*rrf-3*) exhibited a susceptibility to *F. oxysporum* f. sp*. ciceri* under non-avoidance conditions (Figure 1b). In contrast, under avoidance conditions, Fusarium-infected *C. elegans* survived longer, exhibiting avoidance behaviour (Supplemental Figure 1). Microscopic studies revealed that infected worms ingest fungal spores in the absence of a food source, such as *E. coli* (Figure 1c). Histopathological analysis depicted the signs of fungal pathogenesis, including intestinal colonisation of germinating hyphae, resulting in their gross disintegration leading to death (Figure 1d). Of note, Fusarium pathogenesis exhibits similarities among diverse kingdoms. In plants, it causes clogging of vascular bundles and hypoxia, and in humans, the infection leads to fusariosis and haematological malignancies associated with inflammation and hypoxia.

### Global transcriptional reprogramming in response to Fusarium

**Figure 2:**
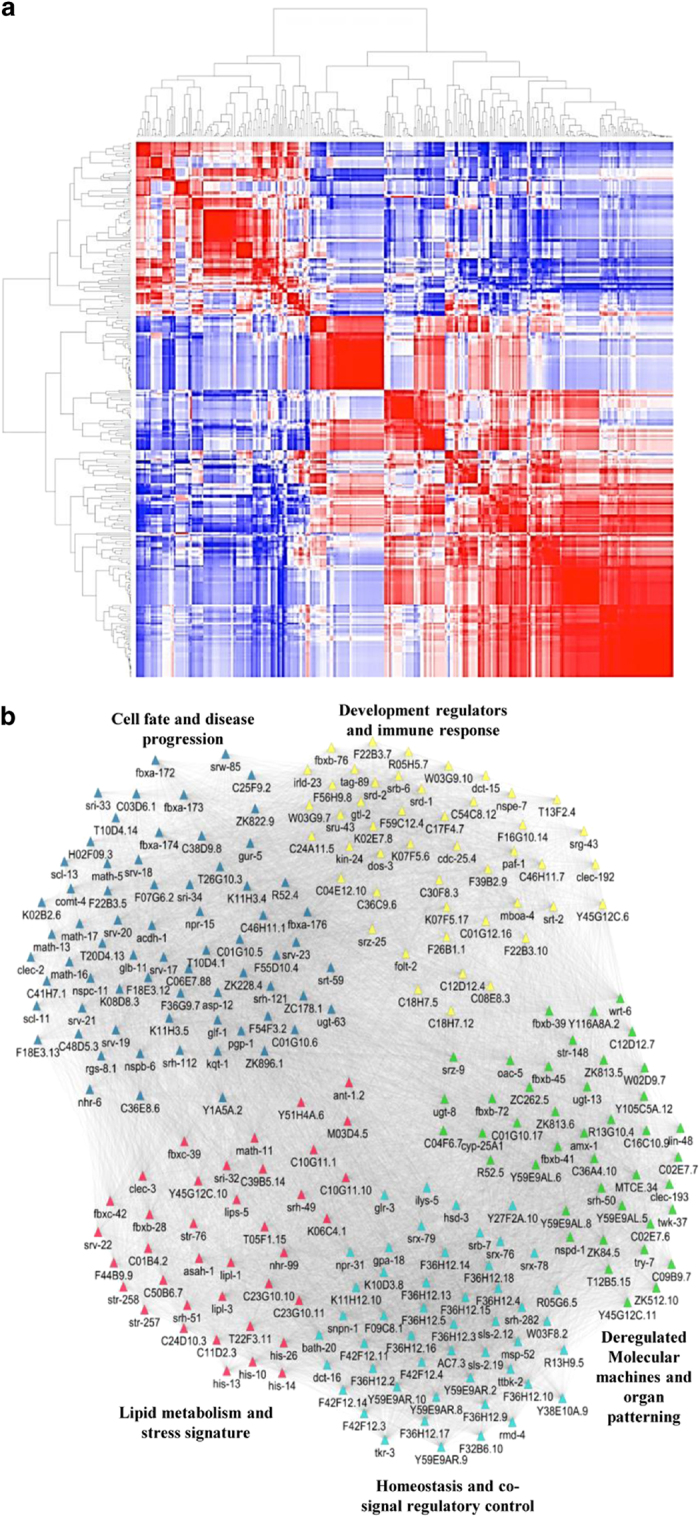
Transcriptional dynamics during *F. oxysporum* infection. (**a**) Unsupervised hierarchical clustering of DEGs in the samples. The heatmap indicates Pearson’s correlation between pairwise sample comparisons, and the dendrogram indicates the average linkage distance between the samples. (**b**) Co-expression network representing five functional modules in *C. elegans* during *F. oxysporum* infection. The node colour denotes network modules, as determined using Cytoscape and the edge width represents a sign of association.

Next, to understand the complexity of disease mechanism and the associated molecular parsimony, we performed RNA-seq analysis of patho-stressed worms (Supplemental Figure 2; also detailed in Supplemental Information). Comprehensive analysis of a transcriptome by plotting the alignments of reads matched along the exons of *C. elegans* chromosomes revealed an extensive transcriptional activity in the genome (Supplemental Figure 3). As expected, RNA-seq reads matched multiple locations in the genome. Differential expression analysis led to the identification of 1312 dysregulated transcripts representing 1024 genes with the false-discovery rate-corrected *P*<0.05 (Supplemental Table 4). A total of 473 protein-coding and 826 non-coding transcripts were found to be differentially expressed, of which 51, 48, and 169 genes were uniquely expressed at 6, 24, and 48 h post infection (hpi), respectively (Supplemental Figures 4a and b). Our data show the importance of both protein-coding and non-coding genes in pathogen-induced response. Next, we segregated DEGs into 10 clusters, as early and late reprogrammers have distinct gene signatures. We performed hierarchical clustering, in which all analysed regions/areas were followed across time, showing stress-regulated clustering of transcripts. Increased correlations between some of the transcripts indicate that transcriptional differences are pronounced during invasion (Figure 2a). Further analysis revealed that the most variable transcripts during infection were predominantly protein coding, for example, *gcy-4, gcy-5, srd-1, kqt-1, ceh-57, nhr-17, hsp-12.6, hsp-16.41*, and *hsp-70*; whereas relatively stable transcripts were dominated by non-coding RNAs. Using Gene Ontology (GO), common and specific themes across the disease state were determined that included a response to stimuli, membrane-bound receptors, signal transducer, and enzymatic activity (Supplemental Figure 4c). To validate the results, we investigated expression levels of 10 DEGs with highest-reads abundance by qRT-PCR and obtained a positive correlation for 6 DEGs (a true positive rate between 50 and 60%) (Supplemental Figure 5).

### Disease network reveals organ-specific deregulated cellular programmes as major drivers of pathogenesis

For delineating the global architecture of disease networks, subsequently, we developed a three-step methodology to construct biological modules associated with pathogenesis. First, we identified differentially regulated disease pathways using literature search. We then built a co-expression network of 245 nodes and 17 857 edges using WGCNA that identified prognostic genes with dense interconnections (Figure 2b). Finally, network modules were examined for disease gene signatures that allowed the identification of novel targets to combat pathogenicity, particularly in worms and other host systems. We classified these modules into five functional categories, namely module 1 (homeostasis and co-signal regulatory control) mapped to 47 transcripts involved in signal transduction and transcriptional regulation, including *tkr-3, ttbk-2, AC7.3, bath-20, dct-16, gpa-18, ilys-5, msp-52,* and *rmd-4*. A closer scrutiny of the module revealed the activation of signalling programmes densely linked to G-protein signalling, serpentine receptor, and chemosensory regulation. Deregulated molecular machines and organ patterning-related module 2 with 41 DEGs was functionally distinguishable in modular organisation. It is hypothesised that multiple but relatively independent regulatory programmes might govern organ-specific disease-associated factors during invasion. Also, a group of stress-related genes associated with a common disorder, particularly fusariosis are expected to share similar cellular and functional attributes. Next, we detected module 3 of 62 DEGs including *asp-12, rgs-8.1, srv-21, srw-85, acdh-1, C01G10.5, clec-2, fbxa-172, fbxa-176, glb-11, math-13, nhr-6, npr-15, pgp-1,* and *scl-11* linked to cellular homeostasis and disease progression. These genes may exert a fine-tuned control of cellular processes and prioritise as potential candidates for drug discovery to treat fusariosis in humans and vascular wilt in plants. Further, we interrogated module 4 (46 DEGs) encompassing development regulators and immune-related factors associated with morphogenesis, organogenesis, development, and response to stimuli. A coordinated interplay of DEGs in this module indicates the role of genome plasticity during patho-stress. Finally, module 5 (38 DEGs) represents a molecular signature associated with lipid metabolism and stress. Of these, *asah-1, lipl-3, C11D2.3, lipl-1*, and *lips-5* are the core element of lipid biogenesis. Whereas, *ant-1.2, C01B4.2, str-76, T22F3.11, C10G11.1, clec-3, fbxb-28, his-10,* and *math-11* are known to be involved in stress-associated processes linking metabolism to immune response.

**Figure 3:**
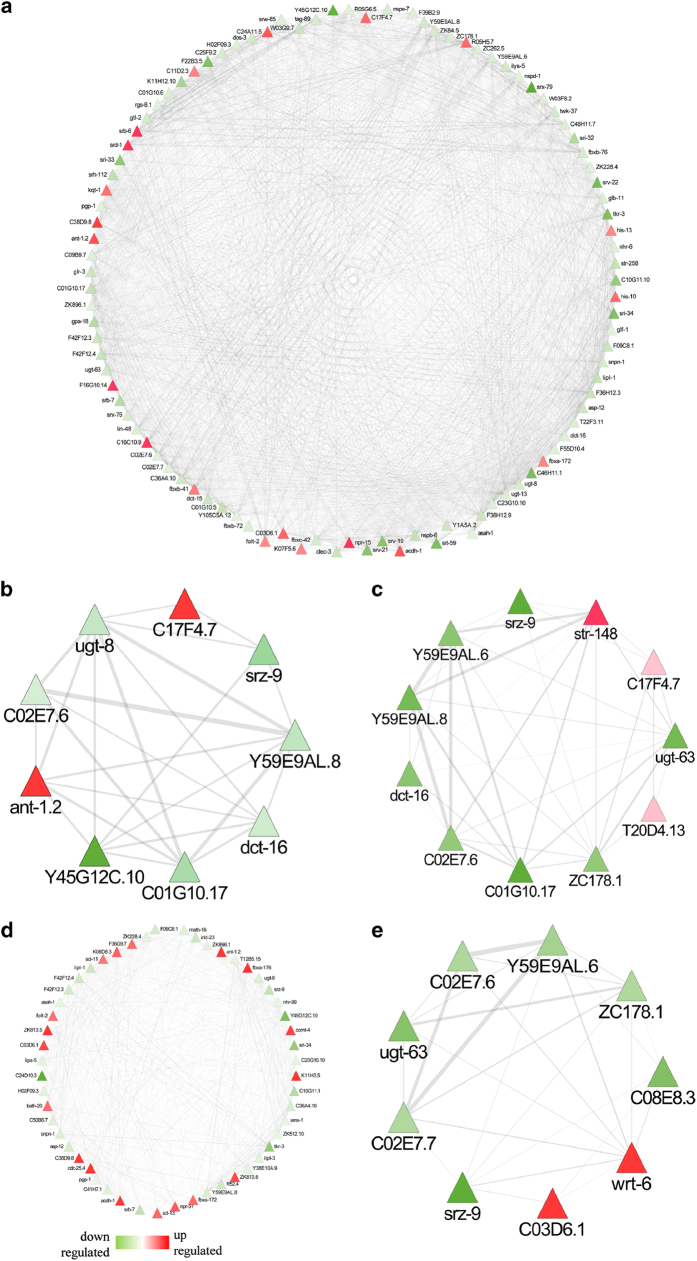
Distribution of tissue-specific gene expression. Tissue-specific genes identified in RNA-Seq data and their distribution in (**a**) neuron, (**b**) phyranx, (**c**) muscle, (**d**) intestine, and (**e**) hypodermis.

We took a step further to explore the crosstalk among different organs (neuron, pharynx, muscle, intestine, and hypodermis) during fungal invasion (Figure 3). A total of 227 DEGs enriched in neurons were deregulated, including 37 *daf-16*-controlled genes, suggesting that *F. oxysporum* infection induces neuronal stress in worms. Further, human orthologues of 4 genes enriched in neurons, namely *tkr-3* (orthologue GPBAR1), *ugt-8* (orthologue UGT3A2), *R05G6.5.1* (orthologue NME5), and *srw-85* (orthologue GPR142) might act as potential candidates to unravel the link between neuronal stress and fusariosis. In addition, the enrichment of 139 intestinal genes associated with disease response detected human orthologues in worms such as *ant-1.2* (orthologue adenine nucleotide translocase ANT genes), *C01B4.2* (orthologue progranulin), and *ZK813.6* (orthologue SPINK5). Molecular changes were also evidenced in hypodermis (14 DEGs), pharynx (24 DEGs), and muscle (35 DEGs). Organ-specific global disease network analyses suggest that the nervous system distinctly perceives stress signals, while pharynx, intestine, muscle, and hypodermis present a consistent tendency and functionality to respond to a fungal pathogen.

### Subnetwork analyses of DAF-16 and SIR-2.1-regulated genes

**Figure 4:**
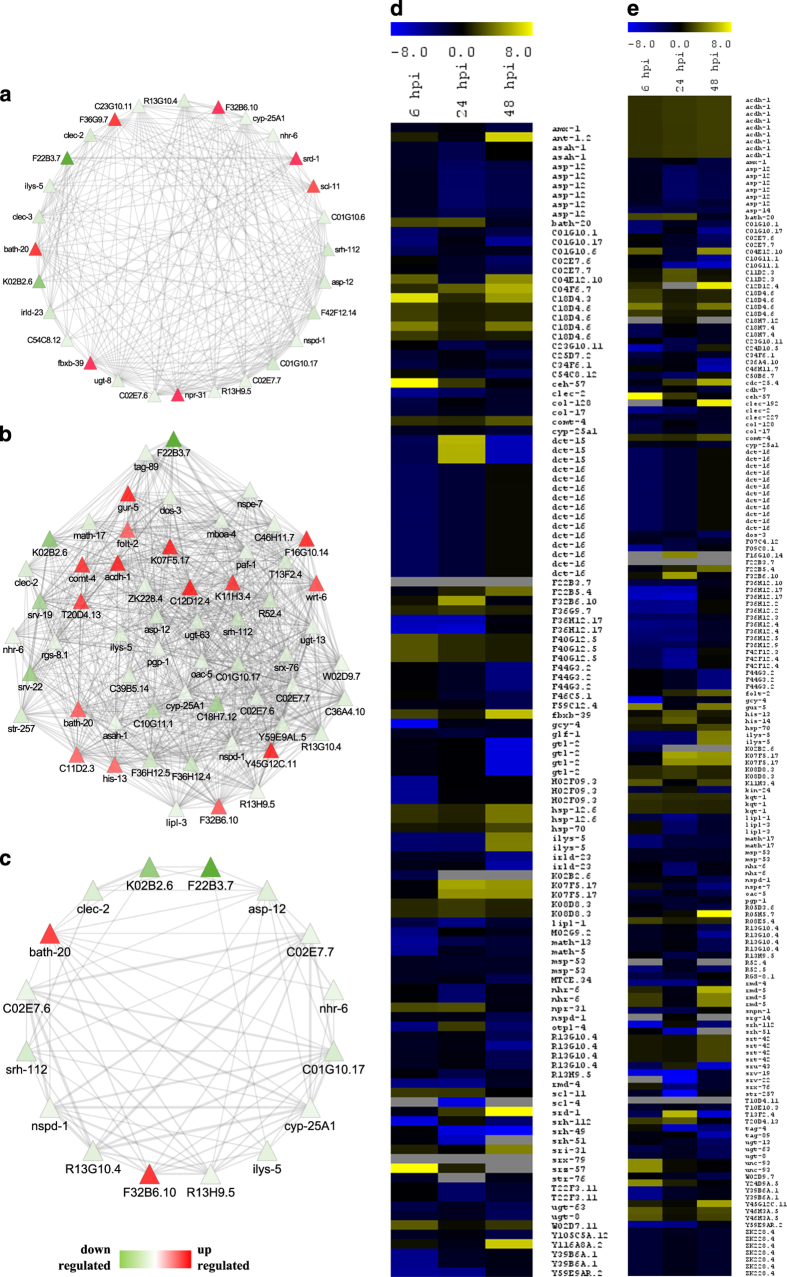
*Fusarium oxysporum* infection activates DAF-16- and SIR-2.1-mediated responses in *C. elegans.* (**a** and **d**) Network and cluster of DAF-16-regulated genes that are differentially expressed in BA15. (**b** and **e**) Network and cluster of SIR-2.1-regulated genes that are differentially expressed in BA15. (**c**) Network representing the genes regulated by both DAF-16 and SIR-2.1 in BA15.

A disease network detected several genes known to be regulated by DAF-16, SIR-2.1, or both. Given that DAF-16 controls innate immunity in *C. elegans,* particularly the Ins/IGF-1-signalling pathway, age-related disorders, and inflammations in humans,^20,21^ we focussed on 78 DAF-16-regulated DEGs that might be associated with *Fusarium* pathogenesis in worms (Figures 4a and d). To address their relevance in stress response, we constructed a targeted gene expression network, encompassing *asp-12* (human orthologue NAPSA), *clec-2*, *hsp-16.41, ilys-5, cyp-25A1, bath-20, R13G-10.4, R13H-9.5,* and *nhr-6* having a distinct functionality during disease condition.

Further, it is known that SIR-2.1 in worms modulates DAF-16 activity.^22^ Interestingly, 126 DEGs found in this study are known to be regulated by SIR-2.1, and a few of them have human orthologues such as *C04E12.10* (orthologue NGLY1), *T20D4.13* (orthologue NGLY1), *ZK896.1* (orthologue EPHX1), *col-128* (orthologue COL22A1), and *rgs-8.1* (orthologue RGSL1) (Figures 4b and e). A correlation of SIR-2.1 with Module-1, 3, 4, and 5 in *Fusarium*-responsive disease network indicates the regulatory relationships between disease and immune sectors through epigenetic regulation. We, therefore, constructed a targeted gene expression network for SIR-2.1 and noted that histones (*his-13*), heat shock proteins (*hsp-16.41, hsp-70*), serpentine receptors (*srh-112, srv-19, srv-22, srx-76,* and *str-257*), transmembrane proteins (*pgp-1, str-257, unc-93, mboa-4,* and *R13G10.4*), and proteins related to metabolism and metabolic disease (*comt-4, ugt-13, F16G10.14, acdh-1, ugt-63, asah-1, cyp-25A1,* and *oac-5*) were enriched in the network.

Both SIR-2.1 and DAF-16 co-regulate diverse biological processes, including stress response, UPR, and longevity.^23^ As gene expression may vary both as a cause and a consequence of the disease, we investigated a set of common genes co-regulated by both DAF-16 and SIR-2.1 to construct a targeted gene expression network, including *asp-12, cyp-25A1, bath-20, ceh-57, clec-2,* and *nhr-6* for dysregulated transcriptional programmes associated with *Fusarium* disease (Figure 4c). In our analysis, regulatory relationships were found to be common among DEGs enriched in diverse organs. We detected neuronal genes of diverse categories such as *srd-1, gcy-4, gcy-5,* and *ceh-57* regulated by an interplay of the above-mentioned genes.^24^ Interestingly, of the 74 intestinal DEGs, 32 were regulated by SIR-2.1 and 21 were regulated by DAF-16. Many genes in the network were related to mitochondria (MT) and endoplasmic reticulum (ER) stress, including *hsp-16.41, hsp-70, cyp-25A1,* and *bath-20*. In addition, UPR-related genes were also regulated by DAF-16 and SIR-2.1. Therefore, we hypothesised that *F. oxysporum* infection in *C. elegans* activate intestinal and neuronal immunity mediated by DAF-16 and SIR-2.1 signal transduction pathway. This analysis prioritises functional recapitulation of a transcriptional complex involved in disease response, including *asp-12, ceh-57, clec-2,* and *nhr-6*. Further, a targeted gene expression network detected regulatory ‘hotspots’ for a disease or immunity with implications in *Fusarium*-associated aetiology.

### Genetic interaction reveals DAF-16-mediated regulation of TGF-β pathway during infection

**Figure 5:**
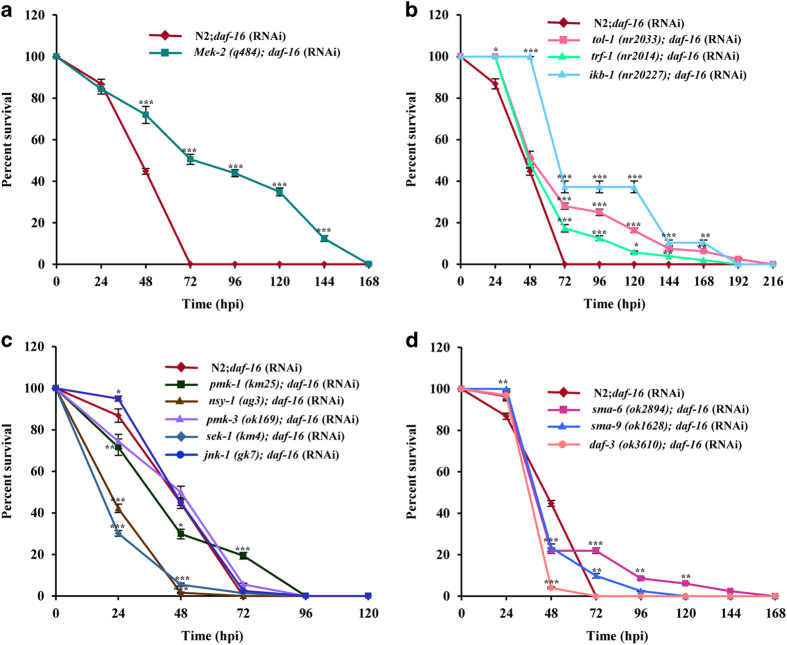
DAF-16 can influence other innate immune pathways. Survivability assay of wild-type N2 and ERK pathway mutants (**a**), Toll pathway mutants (**b**), p38MAPK pathway mutants (**c**), and DBL-1 and TGF-b pathway mutants (**d**) fed with *daf-16* RNAi following *F. oxysporum* infection*. N*=50 adult animals for each strain. Error bars represent S.E. from three independent experiments. *P<0.05, **P<0.01, and ***P<0.001 by one-way ANOVA and Tukey’s *post hoc* test. *P*-values are relative to N2*; daf-16* (RNAi) worms.

Global gene expression and disease network indicated that *F. oxysporum* invasion induces *daf-16*-mediated response in *C. elegans*. As the canonical immune-signalling pathways are known to respond to various bacterial and fungal infections in *C. elegans*,^15^ we carried out survivability assays of different pathway mutants to understand whether DAF-16 influences other immune pathways or acts independently. Worms fed on *daf-16*:RNAi died significantly earlier than the control worms fed on vector control (*P*<0.0001). We observed that silencing of *daf-16* by RNAi had no effect on the survival of ERK and TOLL pathway mutants (*P*>0.05, Figures 5a and b). Thus, we assume that ERK and TOLL pathways operate independently of Ins/IGF-1. We further demonstrate that *daf-*16 was epistatic to p38MAPK pathway mutants except *sek-1* and *nsy-1,* which exhibited enhanced resistance compared to *daf-16* RNAi worms (*P*=0.0024 and 0.0016, respectively (Figure 5c). Hence, from our screen, we conclude that the p38MAPK cascade might be involved during the initial stages of infection when the worms come in contact with the fungal pathogen, *F. oxysporum* and triggers a signalling cascade similar to the response during *Pseudomonas* infection in *C. elegans.*^25^ Similarly, *daf-16* was epistatic to TGF-*β* and DBL-1 pathway mutants (*P*>0.05, Figure 5d). It has been shown that the ASI neuron expressing TGF-*β* influences longevity through *daf-16.*^26^ Thus, Ins/IGF-1 is mainly responsive to *F. oxysporum* infection, while DBL-1, TGF-*β*, TOLL, and ERK-1 are involved in behavioural response through pathogen avoidance.

### ASE and ASI neurons are important for the regulation of innate immunity through DAF-16

**Figure 6:**
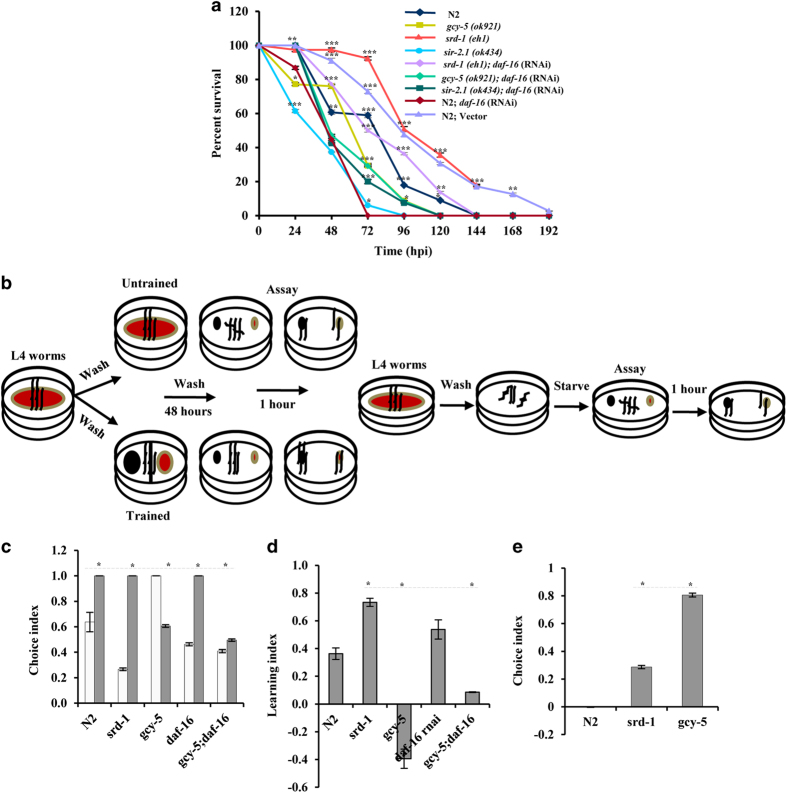
Influence of DAF-16 on neuronal and non-neuronal genes and aversion assay for studying pathogen avoidance in *C. elegans*. (**a**) Wild-type *daf-16* RNAi, *gcy-5*, *srd-1*, *sir-2.1* mutants, *gcy-5*; *daf-16* RNAi, *srd-1*; *daf-16* RNAi, and *sir-2.1*; *daf-16* RNAi were infected with *F. oxysporum* under non-avoidance conditions. *N*=50 adult animals for each strain. Error bars represent S.E. from three independent experiments. *P<0.05, **P<0.01, and ***P<0.001 by one-way ANOVA and Tukey’s *post hoc* test. *P*-values are relative to N2*; daf-16* (RNAi) worms. (**b**) A schematic representation of the assays developed for understanding the behavioural response to *F. oxysporum* in *C. elegans.* (**c**) Choice index of wild-type *srd-1, gcy-5, daf-16,* and *gcy-5;daf-16* worms. The white bar represents the choice index for *E. coli* OP50 and the coloured bar represents the choice index for *F. oxysporum.* (**d**) Learning index of wild-type *srd-1, gcy-5, daf-16,* and *gcy-5;daf-16* worms. (**e**) Normalised choice index of wild-type *srd-1* and *gcy-5* worms. *N*=30 adult animals for each strain. Error bars represent S.E. from three independent experiments. *P<0.05, *t* test with Bonferroni correction.

An organ-based network and transcriptional profiling depicted that subsets of DEGs were enriched in the intestine and neurons. Among deregulated genes in a chemosensory neuron, *gcy-5* and *gcy-4* expressed in ASER^27^ showed downregulation at 6 hpi. In contrast, CEH-57, a homeodomain box transcription factor was upregulated, supporting that it acts in concert with GCY-5 to regulate downstream genes. Also, *srd-1* expressed in ASI^28^ exhibited downregulation at 6 hpi followed by upregulation till 48 hpi, suggesting that chemosensory neurons function in fungal perception. Furthermore, we postulated that *F. oxysporum* infection causes neuronal stress that directly or indirectly affects the expression of DAF-16- and SIR-2.1-regulated genes. To understand the interplay between innate immune systems, neuronal, and intestinal pathways, we compared a set of DAF-16- and SIR-2.1-regulated genes with DEGs identified in response to other bacterial and fungal pathogens.^15^ Few DEGs show a commonality, possibly due to the conservation of innate immune regulators in *C. elegans* during patho-stress (Supplemental Figure 8). Genetic analysis displayed that *daf-16*, *sir-2.1,* and *sir-2.1;daf-16* RNAi worms exhibited a comparable survivability during infection (Figure 6a). To explore the influence of DAF-16 on GCY-5 and SRD-1, we performed *F. oxysporum*-mediated killing of *gcy-5*, *srd-1, gcy5;daf-16* RNAi*, srd1;daf-16* RNAi, and *daf-16*-silenced worms. *gcy-5* worms died earlier than control worms, whereas no difference was observed for *gcy-5;daf-16* RNAi worms (Figure 6a). In contrast, *srd-1* worms, known to be influenced by DAF-16^24^ and TGF-*β* pathways^26^ showed enhanced resistance than *gcy-5* worms. Survivability of *srd-1;daf-16* worms was intermediate of single mutants, implying that SRD-1 contributes to immunity indirectly through DAF-16 and might be involved in avoidance. To further explain the avoidance behaviour, we performed an aversive olfactory-learning assay on *srd-1*, *gcy-5,* and wild-type worms (Figure 6b). The choice index (CI) of the wild-type worms showed that it can naturally avoid *F. oxysporum*; however, previous exposure of the wild-type worms (training) to a fungus altered their behaviour (Figure 6c). Further, a learning index (LI) showed that both *srd-1* and *daf-16*-silenced worms avoid a fungus better than wild-type worms. Interestingly, *daf-16*-silenced *srd-1* worms exhibited negative LI, reiterating that DAF-16 influences SRD-1 for avoidance (Figure 6d). In contrast, a *gcy-5* mutant had better CI under naive conditions, but was defective in learning to avoid a fungus (Figure 6e), as shown previously by Stein and Murphy.^29^ These observations indicate that both *gcy-5* and *srd-1* cause *F. oxysporum* avoidance at an early stage, while during later stages, it may lead to attraction and ingestion of spores. Altogether, our results pointed that *srd-1* governs short- and long-term memory, whereas *gcy-5* might have a role in memory accusation. Finally, DAF-16 controls SRD-1 and regulates behavioural response and innate immunity in worms during *Fusarium* infection.

**Figure 7:**
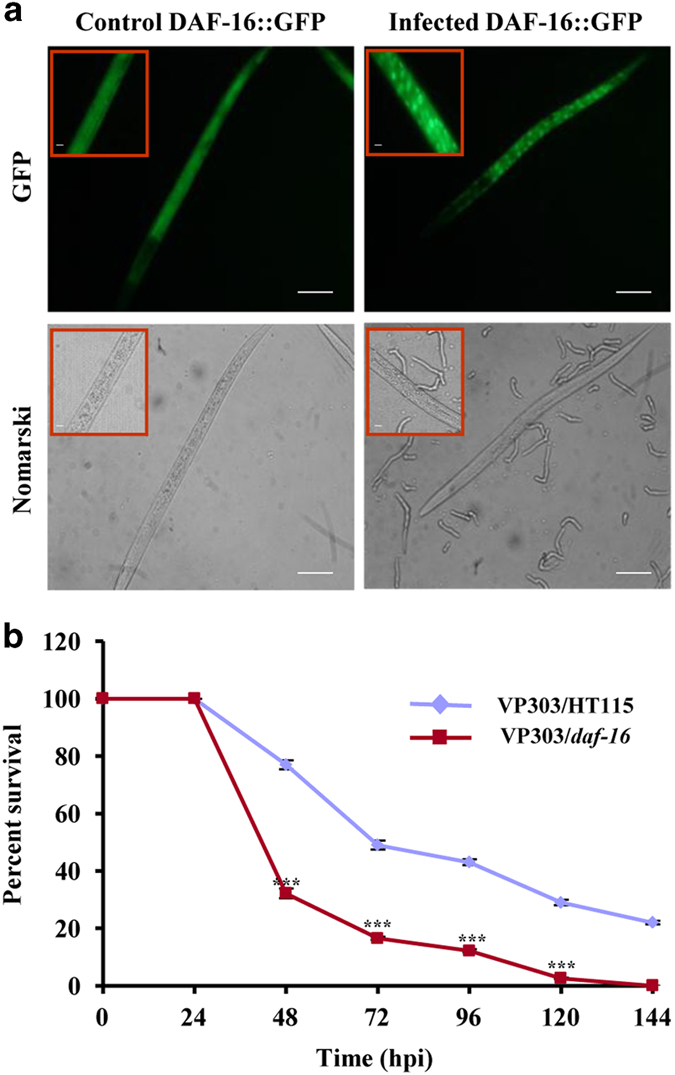
Activation of intestinal DAF-16. (**a**) Translocation of DAF-16::GFP into the nucleus when exposed to *F. oxysporum* at 48 hpi. Scale bars represent 10 *μ*m. (**b**) Survivality assay of VP303 control and VP303 *daf-16* RNAi. *n*=90 adult worms per strain. Error bars represent S.E. from three independent experiments. ***P<0.001 by one-way ANOVA and Tukey’s *post hoc* test. *P*-values are relative to VP303 control worms.

DAF-16 activity is tightly controlled in varied subcellular localisation and by post-translational modifications. Nuclear localisation of DAF-16 in the intestine is required for developmental decisions.^20^ Besides, *daf-16* isoforms have tissue specificity and functional patterns.^21^ Conversely, epidermal DAF-16 controls innate immunity against bacterial pathogens.^19^ Surprisingly, in our study, no expression change was observed for DAF-16. To understand how DAF-16 exerts its function in the signalling cascade, we examined transgenic worms expressing DAF-16::GFP reporter and found a nuclear translocation of DAF-16 in the intestine at 24–48 hpi (Figure 7a). Next, we analysed intestinal *daf-16* RNAi to understand tissue-specific response. We found that VP303 worms^30^ fed on *daf-16* RNAi died significantly earlier than control worms (*P*<0.0001, Figure 7b). Thus, we show that non-neuronal *daf-16* regulates the avoidance and immune response in *C. elegans*. Conclusively, nuclear localisation of intestinal DAF-16 activates signal transduction to ASI, leading to upregulation of *srd-1*. We conclude that *gcy-5* downregulation affects the expression of other stress-related genes that might translocate DAF-16 to the nucleus directly or indirectly through SIR-2.1. During the initial phase, ASE works upstream to intestinal *daf-16*; later on, nuclear *daf-16* and *srd-1* in ASI primes invasion.

## Discussion

**Figure 8:**
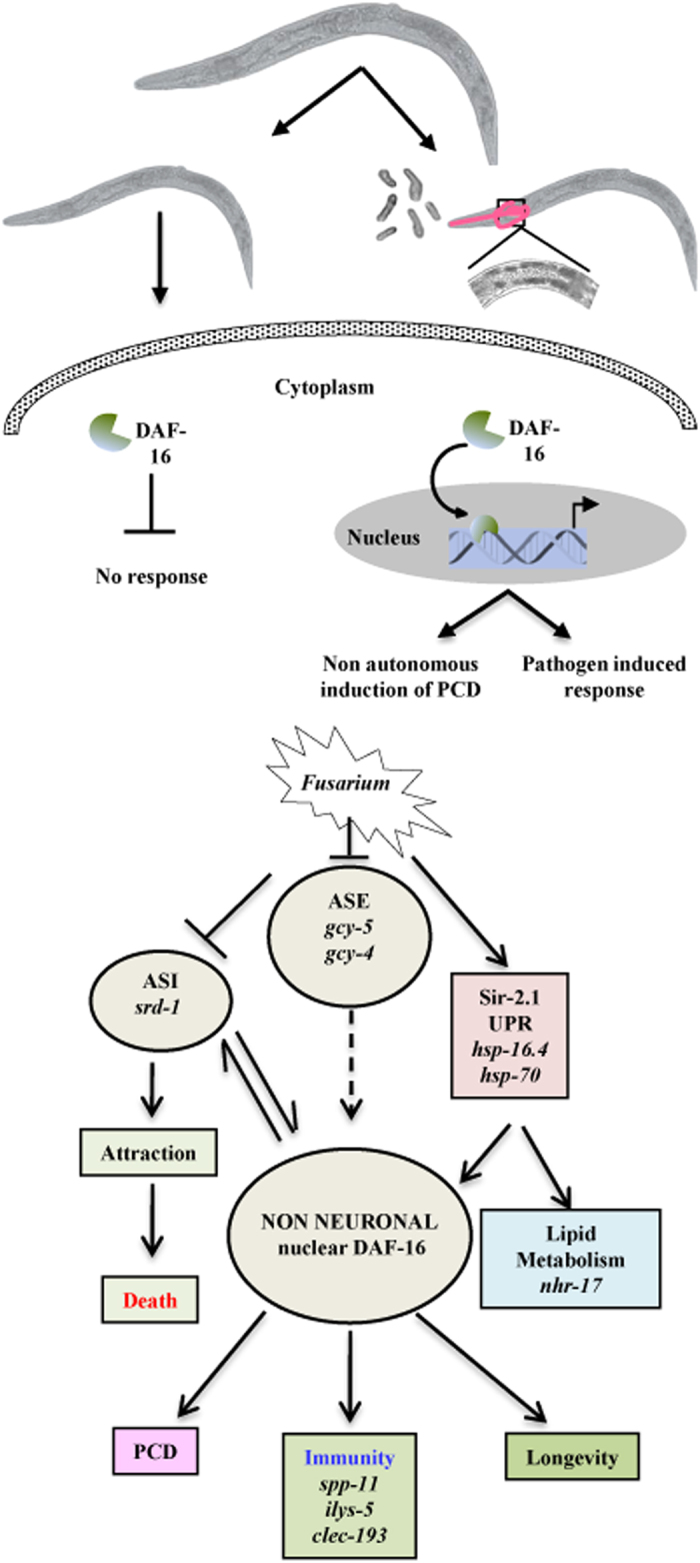
ASE and ASI neurons regulate innate immunity through DAF-16. A model for the mechanism of immune response controlled by the nervous system.

Here, we studied the complex regulatory network and molecular mechanism of *Fusarium* pathogenesis in *C. elegans*. This study indicates the crosstalk of Ins/IGF-1-signalling pathway and neuronal stress response, facilitating the negative regulation of intestinal innate immunity in a patho-stressed worm (Figure 8). RNA perturbation during invasion represents a global signature of infection, including lipid metabolism and neural development, which is known to be regulated by NHR transcription factors and *Fusarium* toxin, fusimosin.^31^ A lipolytic enzyme, ASAH-1 is essential for neuronal development^32^ and GLF-1 plays a crucial role in the synthesis of a surface coat.^33^ Moreover, stress signals from MT and ER lead to altered lipid metabolism and unfolded protein response (UPR). UPR^MT^ is known to be stimulated by enhanced SIR-2.1 (Sirtuin orthologue in *C. elegans*) activity and a lower level of mitochondrial ribosomal proteins.^23^ Thus, the imbalance of neuronal mitonuclear proteins results in intestinal UPR^MT^.^34^ SIR-2.1, an epigenetic regulator involved in stress and longevity exhibits a neuroprotective role through the activation of multiple targets, indicating the conservation of neuronal signalling across kingdoms.^24^ It also controls mitochondrial function through deacetylation of DAF-16/FOXO and plays a role in aging and disease.^35^ CEP-1 (mammalian homologue p53) acts as a regulatory component for stress signals. However, its role in innate immunity is less explored. Focussed RNA-seq analysis used in this study demonstrated that lipid biosynthetic genes, namely *asah-1* and *glf-1* were downregulated, suggesting the deposition of ceramides in tissues and decline in sphingolipids synthesis. *acdh-1*, a mitochondrial enzyme and *ant-1.2*, a *C. elegans* orthologue of adenine nucleotide translocase were upregulated, whereas nduo-3 was downregulated (Supplemental Table 4), reflecting an altered respiration state of the cell. Interestingly, six DEGs involved in lipid biosynthesis had human orthologues such as *asah-1* (orthologues of N-acylsphingosine amidohydrolase), *C18H9.5* (orthologues of SLC), *mboa-4* (orthologues of MBOAT1), *lipl-3* (orthologues of lipase), *hsd-3* (orthologues of SDR43E2), and *ZK896.1* (orthologues of EPHX1) that might be potential targets to link metabolism and immunity. In addition, a nuclear hormone receptor (*nhr-17*), C-type lectins (*clec-192* and *clec-193*), and *ilys-5* were upregulated at 48 hpi. An altered expression of 109 DEGs (~9.25%) regulated by SIR-2.1, 29 CEP-1-regulated DEGs, and three by both CEP-1 and SIR-2.1 might be related to *Fusarium-*induced UPR^MT^ response and points towards the possible interplay of CEP-1, DAF-16, and SIR-2.1. Further, UPR^ER^ leads to chaperones activation and its role in innate immunity has recently been recognised.^36^ This is consistent with our results, as ER chaperones such as *hsp-12.6*, *hsp-16*.4, and *hsp-70* were upregulated during invasion (Supplemental Table 4). Interestingly, Notch receptors had non-developmental roles in nervous systems of adult mammals, Drosophila, and *C. elegans.*^37^ DOS-3 is a transmembrane protein and is predicted to function as a bipartite ligand activating *C. elegans* Notch receptors. Downregulation of *dos-3* in our study may affect the susceptibility of worms to *Fusarium*. Thus, regulatory pathways affect cellular homeostasis and provided a link between metabolism and fungal pathogenesis in worms.

FOXO orthologue of DAF-16 induces the expression of antimicrobial peptides in Drosophila and humans. Further, a transcriptional target of DAF-16, *srz-57* belongs to a serpentine receptor present in plants and regulates various cellular processes associated with extracellular stimuli. It is also known that targets of *mir-59, M02G9.2,* and 21UR-14847, and *H04M03.6* present in our data set are influenced by DAF-16, (http://www.wormbase.org/) suggesting that non-coding RNAs might control the immune response through DAF-16. Our findings were in concordance with the previous data^38^ reporting non-coding RNA-mediated regulation of PMK-1 and DAF-2/DAF-16 deactivation of innate immune system. The identification of upstream genetic regulators of these pathways in worms might offer opportunities for understanding disease aetiology. It has been well documented that the interplay of a neuro-immune system in the context of non-neuronal tissues forms the basis of crosstalk among different organs; however, the underlying mechanisms by which these processes are coordinated in response to fungal disease remain to be poorly understood. Of these, the intestine and nervous system occupy a central role in environmental adaptation. Elegant work has shown that non-autonomous signals from different neurons have the potential to regulate non-neuronal tissues^39^ and immunity.^16,17^ However, a mechanistic crosstalk between the intestine and nervous system that influences innate immunity through forkhead transcription factor DAF-16 remains to be less explored. Differential expression of GCY-4 and GCY-5, expressed in ASE and SRD-1, and expressed in ASI highlights the role of ASE and ASI neurons in sensing the fungus. This hypothesis is further supported by genetic analysis, depicting that *daf-16*, *sir-2.1,* and *sir-2.1;daf-16* RNAi worms exhibited a comparable survivability. Further, *gcy-5* worms died earlier than control worms; whereas *srd-1* worms showed enhanced resistance. In addition, CEH-57 and KQT-1 (an orthologue of human cardiac KvLQT-1 channel) were also differentially expressed. Our finding indicates hypoxia as a major cause and concern in the case of *Fusarium* pathogenesis in *C. elegans* similar to its functional homology in plants and humans. Concomitantly, we identified an oxygen sensor (BAG neuron) such as *R05G6.5*, DEGs related to pharyngeal neurons (*W03F8.2*) and musculature (*C17F4.7, C24D10.5, dct-16, M02H5.8,* and *Y45G12C.10*) in patho-stressed *C. elegans.* Additionally, pan-neuronal G-protein-coupled receptor was predominantly dysregulated in ASH and ASI during *Pseudomonas aeruginosa* infection in *C. elegans.*^17^ Ligand-gated ion channel LGC-8, the gap junction protein; INX-18,^40^ and GLR-3, an extracellular-glutamate-gated ion channel^41^ expressed in neurons, coelomocytes, and excretory cells, respectively, was downregulated (Supplementary Table 4). Also, WRT-6, a hedgehog-like protein expressed in hypodermis, neuronal sheath cells, and socket cells was upregulated at 48 hpi, suggesting that intercellular signalling is enhanced during infection. Thus, these DEGs might provide potential targets to unravel the relation between neuronal synapsis and innate immunity across a kingdom. In addition, our data identified diverse regulatory hubs displaying synergistic or antagonistic associations of disease or immune regulators and signal components.

In this study, we showed for the first time how a functional interplay of neuronal genes, intestinal DAF-16 regulatory components, and innate immune system regulates fungal pathogenesis. Potential mechanisms include the dysregulated expression of neuronal genes, translocation and combinatorial expression of DAF-16- and SIR-2.1-regulated DEGs, and perturbation of upstream-and downstream-signalling cascades. *C. elegans–F. oxysporum* pathosystem might be instrumental in testing disease orthologues in plants and humans. Our study identifies novel molecular cassettes that perceive and respond to fungal virulence factors and provides mechanistic and diagnostic implications. Finally, we detected compendia of *C. elegans* orthologues of human genes in various innate immune pathways, including *asp-12* (orthologue NAPSA), *R08F11.4* (orthologue Williams–Beuren syndrome), and *C18H7.4* (orthologue FES pro-oncogene). These orthologues might possess translational potential to understand fusariosis in humans.

## Materials and methods

### Strain maintenance

*C. elegans* strains were maintained on nematode growth medium (NGM) agar plates seeded with OP_50_ *E. coli* at 25 °C, as described earlier, except for BA15 *(rrf-3).*^42^ The strains used in this study were obtained from the *C. elegans* Genetics Center (University of Minnesota, MN, USA) and are listed in Supplemental Table 5.

*E. coli* strain OP50 was grown in Luria-Bertani (LB) broth at 37 °C, while strain HT115 was grown in LB supplemented with 25 *μ*g/*μ*l tetracycline.^43^ The fungal strains used in this study are *Fusarium oxysporum* f. sp*. ciceri*,^8^ *Fusarium oxysporum* f. sp*. methioli*,^44^ and *Fusarium oxysporum* f. sp*. lycopersici*,^11^ which are known to infect *Cicer arietinum*, *Arabidopsis thaliana,* and *Solanum lycopersicum*, respectively. These stains were maintained on potato dextrose agar at 28 °C.

### *C. elegans* killing assay

*F. oxysporum* was grown on PDB medium for 3–5 days, the culture was filtered, and the spores were washed twice with NGM broth. The spores were resuspended at a concentration of 10^7^/ml of NGM liquid medium for the plate assay. The avoidance assay was performed, as described earlier^45^ and small lawns were prepared by spreading spores covering a small area on a 60-mm plate. A non-avoidance assay was performed in a 12-well plate with the agar fully covered with spores (60 *μ*l/well).^46^ The plates were dried at room temperature (RT) for 2 h. Synchronised L4 worms were raised as described^45^ and further washed with a medium containing 100 *μ*g/ml kanamycin. The worms were placed in NGM broth containing 100 *μ*g/ml kanamycin and 1 mg/ml 5-fluoro-2′-deoxyuridine (Sigma), except when BA15 was used and incubated at 25 °C for 2 h for starvation so as not to hamper the growth of *F. oxysporum.*^42^ The worms were examined for viability using either Nikon 80i DIC microscope or SZX7 Olympus stereozoom microscope and scored at the times indicated, and were considered dead when they did not show pharyngeal pumping. All the experiments were conducted in two to three biological replicates.

### Statistical analysis of experimental data

Statistical significance was calculated using PRISM v.6 and R software environment. Longrank Mantel–Heanszel test was used to compare the survival curves. Survival curves were considered to be different than the corresponding control, as indicated in Supplemental Table 5 when the *P*-value was <0.05.

### Localisation study

Synchronised worms were exposed to *F. oxysporum* till 48 h at 25 °C and mounted on 2% agarose with 30 mM NaN_3_ in M9 medium for DAF-16 localisation study. The worms were visualised using Nikon 80i DIC microscope using the FITC filter. At least two independent biological replicates were screened for GFP expression.

### Cellular integrity assay

The integrity of intestinal cells was visualised using propidium iodide staining, as described previously.^47^ Briefly, wild-type worms were transferred to plates seeded with either *F. oxysporum* under non-avoidance conditions or OP50 at 24 and 48 h. Worms were stained with 20 mg/ml propidium iodide for 30 min, washed twice with M9 buffer to remove the excess dye, and visualised under Nikon 80i DIC microscope using the TRITC filter.

### RNA extraction, library preparation, and sequencing

Worms were grown in NGM broth, collected, and the total RNA was isolated using TRIzol reagent-based extraction method (Invitrogen, Carlsbad, CA, USA). RNA was further purified using RNAeasy kit (Qiagen, Hilden, Germany) and the quality was assessed on an Agilent 2100 bioanalyser. High-quality RNA samples were used for subsequent studies. RNA-seq experiments were performed using RNA libraries generated from synchronised L4 worms representing three distinct time points. Altogether, seven samples were collected at 6, 24, and 48 h with and without infection and at 0 h as control (Supplemental Table 6). Libraries were sequenced on an Illumina HiSeq 2000 using paired-end chemistry and 101-bp cycles. qRT-PCR was performed to validate the expression of differentially expressed transcripts. To attain a higher depth, libraries were prepared from two replicates.

### Raw reads mapping and assembly

Libraries were analysed with quality control tools in CLC Genomics Workbench (version 7.0.4) and FastQC (http://www.bioinformatics.babraham.ac.uk/projects/fastqc/). We proceeded with an adapter sequence that removed high-quality reads for the RNA-Seq analysis using the workflow, as depicted in Supplemental Figure 2. Our replicates were significantly correlated. Therefore, we merged the replicates together to improve statistical power and further analyses were performed using the merged data. The TopHat-Cufflinks pipeline (version 2.0.9)^48^ was used to align the sequences, with the default parameters allowing two mismatches. The expected fragment length and the ‘small-anchor-fraction’ were set to default, 200 bp and 0.09, respectively, with at least 9 bp on each side of an exon junction for the 101-bp RNA-seq data. The sequence-aligned files generated by Tophat were subjected to Cufflinks (version 2.1.1),^49^ which assembled the aligned data set into transfrags independently of the existing gene annotations.^50^ Isoform prediction file generated from all the samples and the reference annotations were merged for differential expression analysis by Cuffmerge. The expected fragment length and ‘min-alignment count’ was set to default, 200 bp and 10, respectively. Distribution of the FPKM values across the sample was plotted as a box-and-whisker plot (Supplemental Figure 6a). From the total RNA-Seq data, we detected the expression of 30–57 transcripts with an FPKM>5000 (Supplemental Figure 6b). Pearson correlation coefficient was calculated using R-package.^51^ We also compared the expression level of transcripts and found that many genes, as measured by FPKM were expressed at a similar level, across all the samples with high Pearson correlation coefficients between different samples (Supplemental Table 7).

Trinity (trinityrnaseq_r2014_04_13pm)^52^ with default parameters (k-mer=25) was used to assemble the reads *de novo* from each of the seven RNA-seq samples generated after merging the replicates.

### Identification of unannotated coding, non-coding RNAs, and novel splice junctions

Trinity and Cufflinks that generated fasta files were analysed further (Supplemental Figure 2) to identify regions of active transcription that do not overlap to the existing gene annotations using BLASTn (*E*-value=1e−10) against the following databases: (1) *C. elegans* genome (WS 240) and (2) *C. elegans* ESTs. Further, we searched for any possible match against *F. oxysporum* using Fusarium comparative database from Broad Institute (https://www.broadinstitute.org/annotation/genome/fusarium_group/MultiHome.html). The annotated contigs and the contigs having a match with *Fusarium* genome were filtered and the contigs that did not have a significant BLASTn hit to *C. elegans* genome, ESTs, and *Fusarium* were processed using BLASTX (*E*-value⩽1e−10) against NCBI nr, PfamA, and PfamB,^53^ and UniProtKB/Swiss-Prot^54^ databases to identify potential protein-coding genes. The identified novel protein-coding contigs were then filtered and the final processing was performed on the remaining unannotated contigs using BLASTX (*E*-value⩽1e−10) against the NONCODE database^55^ and BLASTn against Rfam database^56^ to curate a confident set of novel non-coding transcripts. To predict splice junctions, RNA-seq data sets were subjected to SpliceMap^57^ using default parameters.

### Differential gene expression and gene ontology analyses

We used EdgeR,^58^ to normalise tag distribution per library and determined the transcript abundance by mapping reads against the reference genome (WS240). The Benjamini and Hochberg’s approach was used to adjust the resulting *P*-values for controlling the false-discovery rate. The transcripts having a false-discovery rate of <0.05 were considered as differentially expressed. GO analysis was performed using Blast2GO.^59^

### Heatmap and tissue-enrichment analyses

K-means clustering was performed on differentially expressed genes with the K-means/K-medians support module and heatmaps were generated using MeV v4.9.0 (MultiExperiment Viewer) (http://www.tm4.org/).^60^ Tissue-enrichment study was done using Wormmine tool (http://www.wormbase.org/tools/wormmine) based on previous studies^24,61–64^ and genes were classified as intestine and neuron associated. In order to compare between different releases of the Wormbase, we used the software ‘WormBase Converter’.^15^

### Gene co-expression network construction

The differentially expressed genes identified in time-lapse analyses were used to construct the weighted gene co-expression network using the R package WGCNA (version 1.51).^65,66^ A total of 327 transcripts were used to construct the network of all 7 samples (C0, C6, I6, C24, I24, C48, and I48). In the WGCNA algorithm, the elements in the co-expression matrix are defined as the weighted value of the correlation coefficient. The absolute values of Pearson’s correlation coefficients were calculated for all possible gene pairs and the correlation matrix was transformed into a weighted adjacency matrix using a *β*-power of 6, so that the final correlation matrix followed an approximate scale-free topology.^67^ The connection strength between each set of gene pairs varies with the expression profile and is used to calculate the topological overlap measure (TOM). Genes were clustered using an average linkage with their TOM distances. Co-expression modules were defined as branches of the resulting clustering tree by specifying the branch height to cut, as well as the minimal number of genes to be included into a module. WGCNA cut-tree hybrid algorithm was used optimising the minimum module size to 30 and a tree-cut height of 0.25 in order to merge the neighbouring network modules with similar expression trends in different samples. Subnetworks were extracted for DAF-16- and SIR-2.1-regulated genes and genes expressed in specific organs. Co-expression networks were visualised in Cytoscape (http://www.cytoscape.org/) with topological overlap values as the edge weight.^67^ The CPM values were log2 transformed, converted into RGB colour codes, and used to display the relative expression levels in different networks.

### miRNA and piRNA target prediction

miRNA-binding sites were obtained from Miranda with an mirSVR score (a measure of the likelihood that an miRNA targets a certain sequence) less than −0.3. Targets for piRNA were identified based on Bagjin *et al.*^68^

### Quantitative RT-PCR analysis

The total RNA was used to generate double-stranded cDNA using the High Capacity cDNA Reverse Transcription Kit (Applied Biosystems). qRT-PCR was done using the Power SYBER green PCR master mix (Applied Biosystems) on an Applied Biosystems 7500 real-time PCR machine in a 96-well-plate format. Actin (*act-1*) was used as the endogenous control and relative fold changes were calculated using the comparative *C*_*T*_(2^−ΔΔ*CT*^) method.^69^ Primer sequences are listed in Supplemental Table 8.

### RNAi experiments

RNAi phenotypes were generated by feeding worms with *E. coli* strain HT115 (DE3) expressing double-stranded RNA that is homologous to a target gene, as described earlier.^43^ HT115 (DE3) expressing the appropriate vector was grown on LB agar plates containing 50 *μ*g/ml ampicillin and 25 *μ*g/ml tetracycline. For seeding, a single colony was inoculated in LB broth containing 100 *μ*g/ml ampicillin at 37 °C overnight and plated onto NGM plates containing 100 *μ*g/ml carbenicillin and 3 mM isopropyl *β*-D thiogalactoside. Synchronised L1 worms were placed on RNAi or vector control plates for 24 h at 25 °C and L4 worms were used for subsequent infection assays.

### Aversive olfactory-learning assay

The aversive olfactory-training assays were performed, as described previously with modifications.^70^ Briefly, worms were synchronised by bleaching and L4-stage larvae were used for further studies on training and naive plates. One group was grown on a control NGM plate with ~300 *μ*l of an overnight culture of *E. coli* OP50 (untrained or naive plate) and the other group was grown on a NGM plate with ~200 *μ*l of spore culture *F. oxysporum* on one side and ~50 *μ*l of an *E. coli* OP50 culture on the other side (training plate). After 48 h of training at 25 °C, the worms from both trained and untrained plates were placed on assay plates and counting was done after 1 h. Assay plates were prepared on 35-mm NGM plates with 20 *μ*l of *F. oxysporum* and OP was placed on each end of the plate.

## Additional information

**Publisher’s note:** Springer Nature remains neutral with regard to jurisdictional claims in published maps and institutional affiliations.
