# Supplementary material for: Interplay of neuronal and non-neuronal genes regulates intestinal DAF-16-mediated immune response during Fusarium infection of Caenorhabditis elegans
Source: Cell Death Discov. 2017 Nov 13;3:17073–. doi: 10.1038/cddiscovery.2017.73 (PMC5684781; doi:10.1038/cddiscovery.2017.73)
Supplement: Supplemental Information [file cddiscovery201773-s1.pdf]

Supplementary Data:

Results

RNA-seq analysis and discovery of novel transcribed regions

We implemented an experimental framework involving sequencing, *ab initio* and *de novo* assembly and mapping of seven RNA-seq libraries (Supplementary Fig. 2), which yielded over 35-46 million reads for each sample. In total, 23,906.7 million bp of the mapped sequence were generated representing 278.8-fold coverage of the genome using Tophat (Supplementary Table 1). Overall, we identified 98.30% and 83.2% reads which were mapped to the annotated *C. elegans* genome and wormbase (WS240) in all the samples. Our data revealed that numbers of contigs were high in *de novo* assembly but mean length of the transcripts were 3.36 times longer in map based assembled data (Supplementary Fig. 6). Cufflinks transcripts showed 100% match with the reference genome (WS240) (E-value $\leq 1e^{-10}$) while Trinity contigs showed 90.5% match in the annotation. Furthermore, the unaligned transcripts having match with *Fusarium* genome were removed. Transcripts were analysed further for novel transcribed regions (NTRs), novel splice junctions and differential gene expression (DEGs). Moreover, we identified few novel transcripts and segregated them further into protein-coding transcripts and the noncoding RNAs (Supplementary Fig. 7a). CuffMerge was used to analyze all the individual contigs generated by cufflinks for each sample and an overall set of 71,626 unique transcripts was obtained, of which 78.11% showed match with the reference genome. We detected the expression of 55,951 transcripts representing 30,519 annotated genes in the reference genome. Among the unannotated contigs (21.89% of the CuffMerge transcripts), we identified three novel protein-

coding transcripts which are conserved in closely related *Caenorhabditis briggsae* showing hit in the NCBInr database (Supplementary Table 2 and 3).

For potential noncoding RNAs, we searched the Rfam and NONCODE databases with 15,672 Cufflinks transcripts that passed through the protein-coding filters, resulting into the identification of 15,672 transcripts as putative noncoding RNAs. We also examined the Trinity contigs for potential protein coding as well as noncoding transcripts. Out of 8537 unannotated trinity transcripts identified in all samples, 2910 found to be novel protein coding transcripts, of which 964 were analyzed for the presence of protein domain (Supplementary Table 3). Whereas 28 transcripts showed significant hit in NONCODE and Rfam databases and were categorized as definite non-coding transcripts, leaving 5,599 transcripts as putative non-coding. Stimulatory and inhibitory pathways with their impact on functional attributes were further interrogated for small RNAs and their targets in *C. elegans* during invasion. We identified five miRNAs (*mir-59, mir-269, mir-271, mir-2217, mir-4922.1*) and several piRNAs in our datasets. miRNA can trigger or suppress its target genes, however, function of piRNA in silencing endogenous genes during path-stress is unknown. We searched transcriptome dataset for non-coding RNAs' targets and found one miRNA and twelve piRNA targets differentially expressed during infection. Furthermore, we identified 89,614 to 95,622 spliced junctions, including approximately 10,000 to 13,000 novel junctions for each of the seven samples that might belong to different gene models (Supplementary Fig. 7b). Our results have revealed further scope for determining whether these protein-coding as well as non-coding RNAs and novel splice junctions have important regulatory function during host specific immune response.

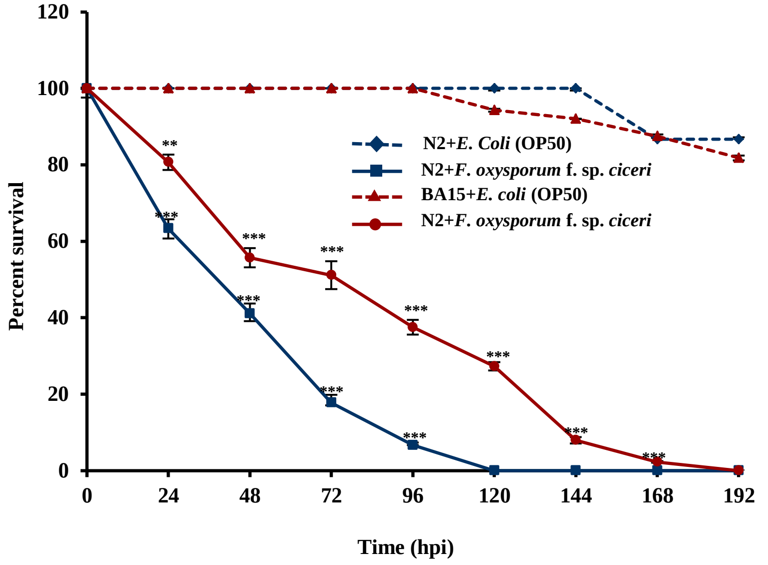

Supplementary Figure 1 *F. oxysporum* mediated killing of worms is delayed under avoidance conditions. BA15 [*rrf-3 (hc15)*] and N2 were exposed to *F. oxysporum* under avoidance condition. ~~Shown is a representative assay of three independent experiments.~~ n=90 adult worms. Error bars represent SE from three independent experiments. *p < 0.05, **p < 0.01, ***p < 0.001 by one way ANOVA and Tukey's post-hoc test.

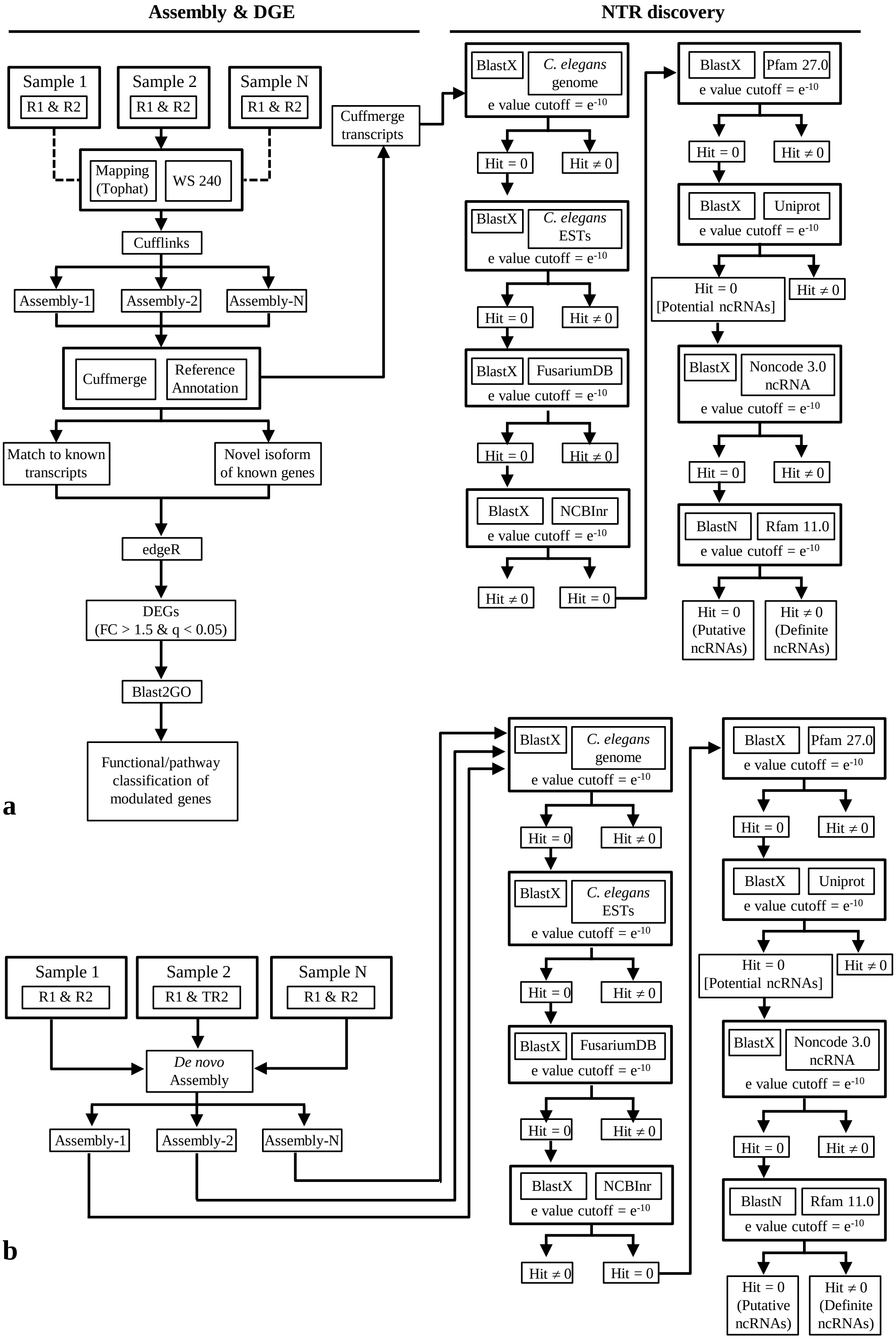

Supplementary Figure 2 RNA-Seq analysis. (a) Reference based assembly, differential gene expression analyses and novel gene discovery using Tophat-Cufflinks. (b) *de novo* assembly and novel gene discovery using Trinity.

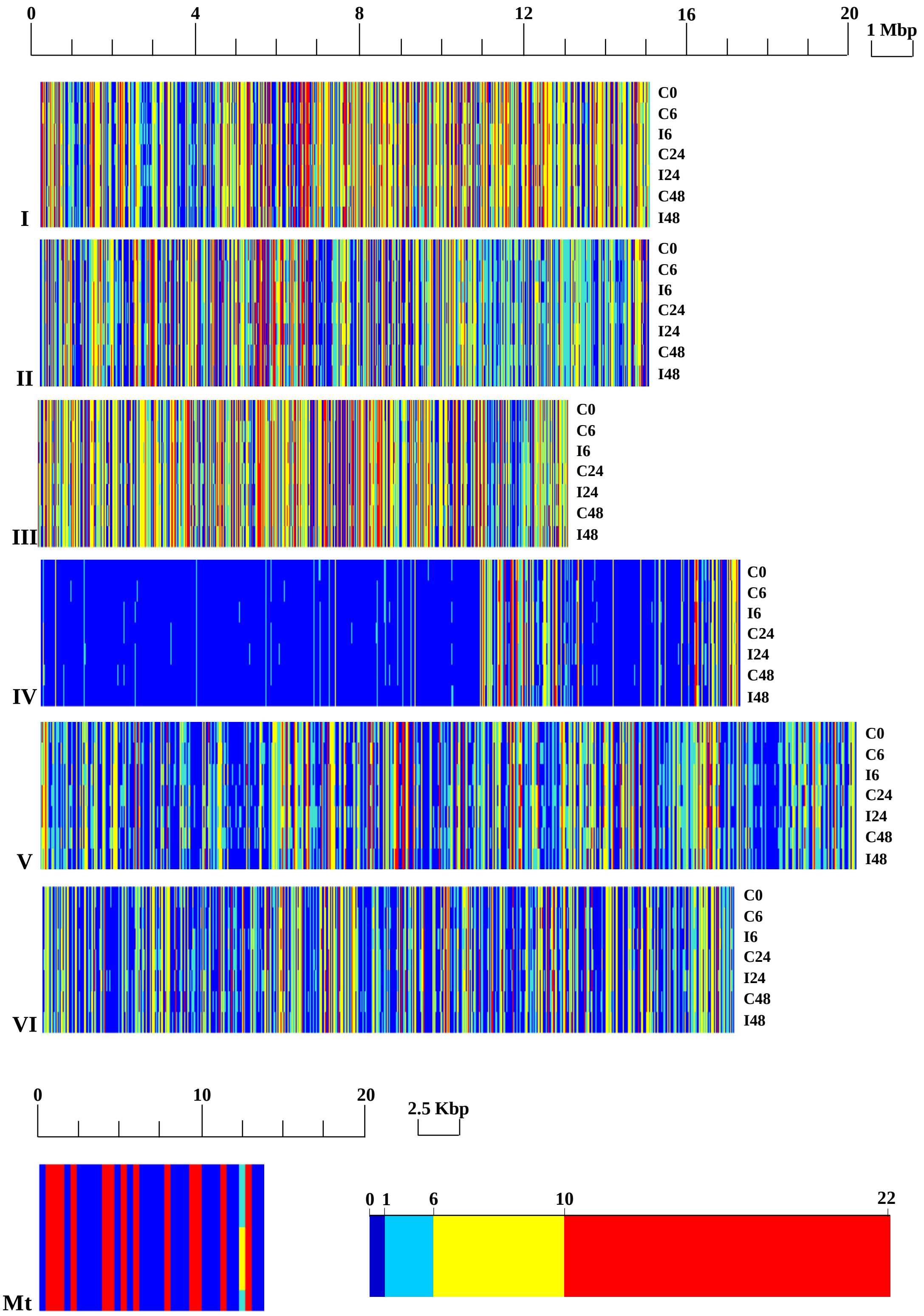

Supplementary Figure 3 The genome distribution of transcribed regions in *C. elegans*. A schematic drawing of the chromosomes shown below the paired-end read density. Colors represent different transcription levels for each base (log2 tag count). Numbers of chromosome regions are shown on the left. Mito, mitochondria.

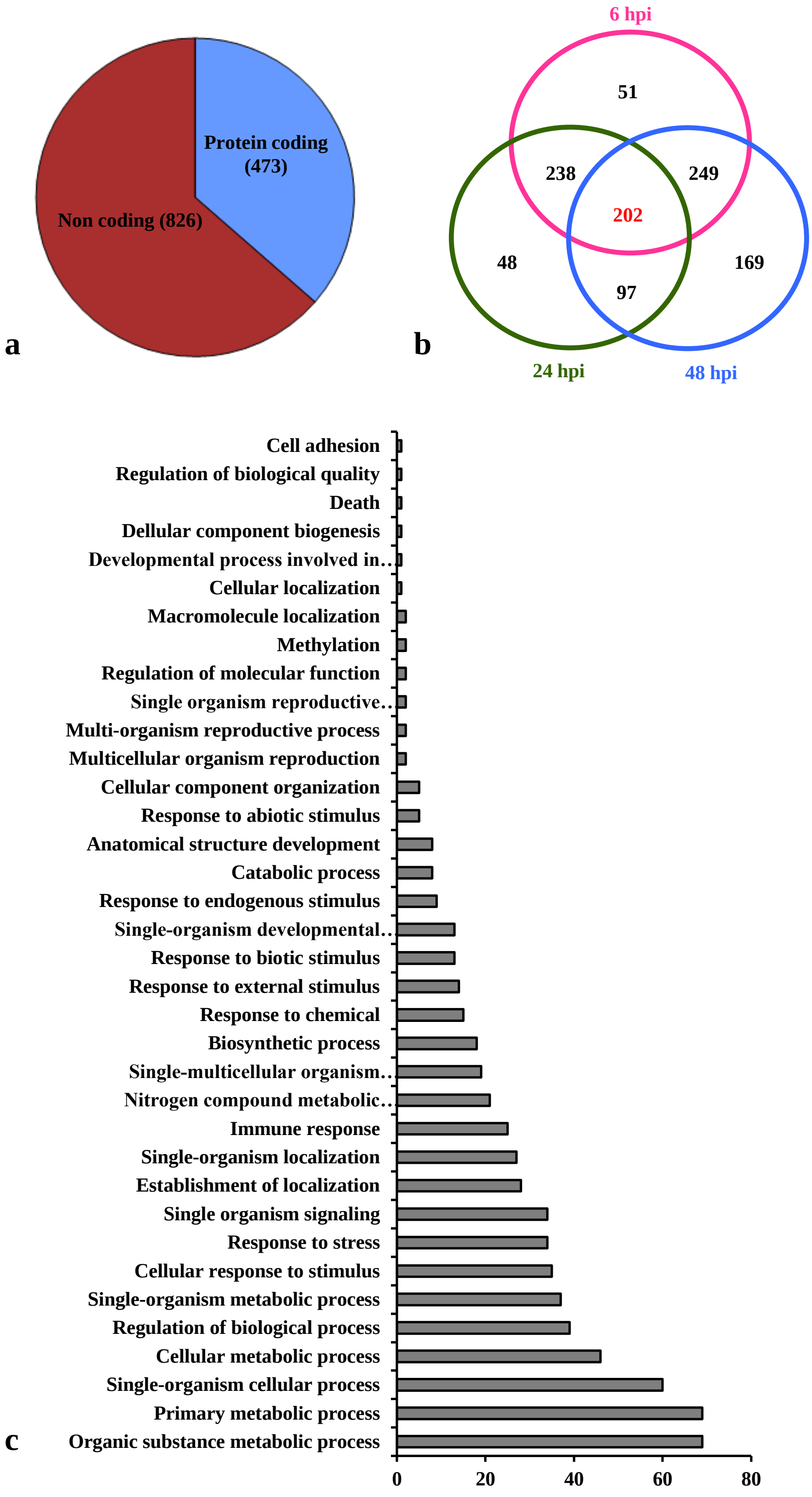

Supplementary Figure 4 *Fusarium oxysporum* mediated responses in *C. elegans*. (a) Pie chart depicting protein coding and definite non-coding transcripts among differentially expressed set of genes. (b) Venn diagrams showing common and unique sets of genes that were deregulated in wild-type worms exposed to *F. oxysporum*. (c) Bar graph showing Gene Ontology (GO) analysis of differentially expressed genes.

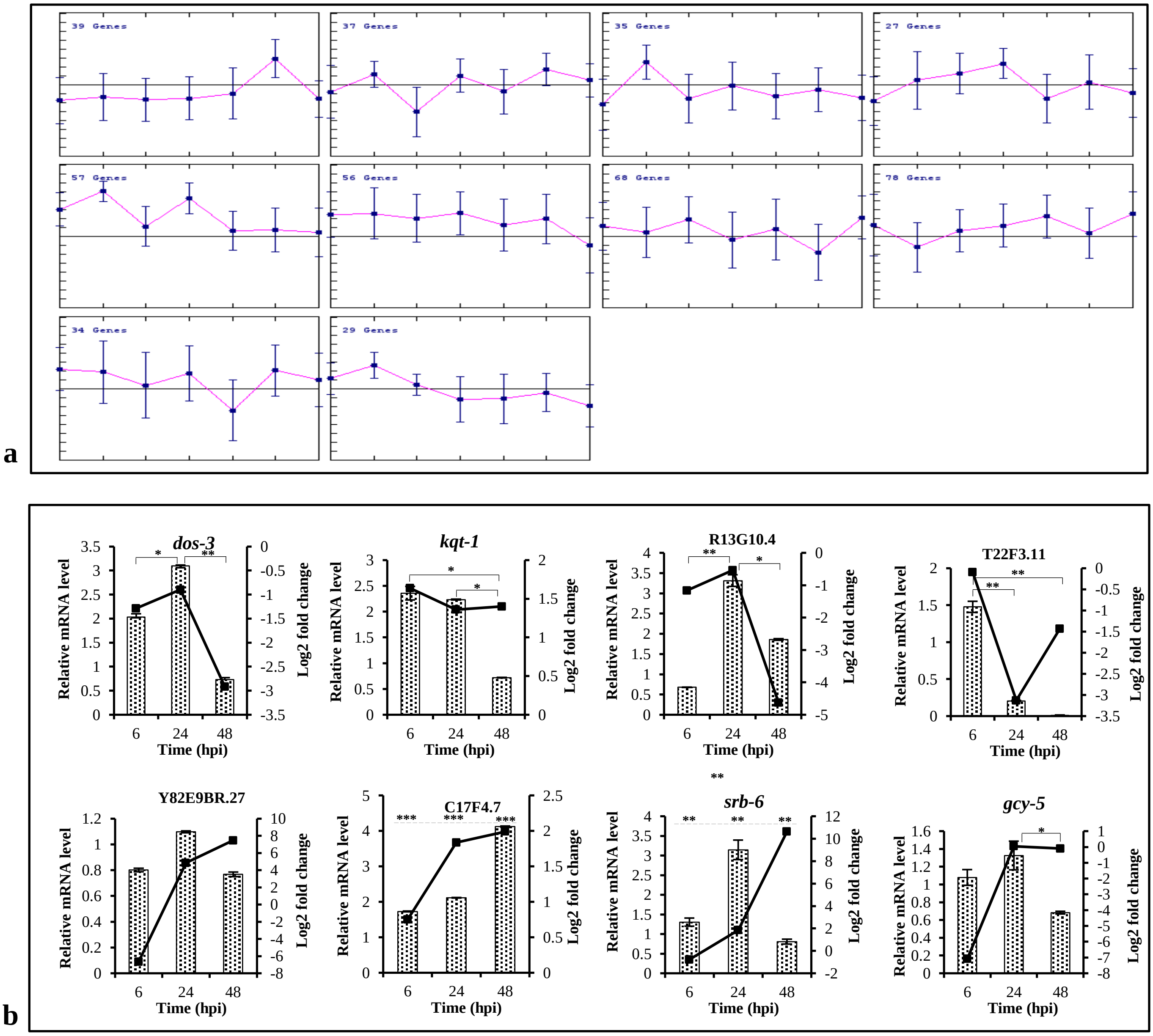

Supplementary Figure 5 Analysis and validation of differentially expressed genes. (a) Complete K-means cluster maps of differentially expressed genes. Lines show the expression pattern at 6 hpi, 24 hpi and 48 hpi. (b) Quantitative RT-PCR analysis of differentially expressed genes in WT worms infected with *F. oxysporum* relative to WT animals fed *E. coli* OP50. Data were analyzed by normalization to pan-actin and relative mRNA level (bar) was determined using the comparative cycle threshold method and plotted with the Log2 fold change (solid line) obtained from RNA-seq analysis. ~~Shown is the average of two-three independent experiments.~~ Error bars represent SE from two-three independent experiments. *p < 0.05, **p < 0.01, ***p < 0.001 by one way ANOVA and Tukey's post-hoc test.

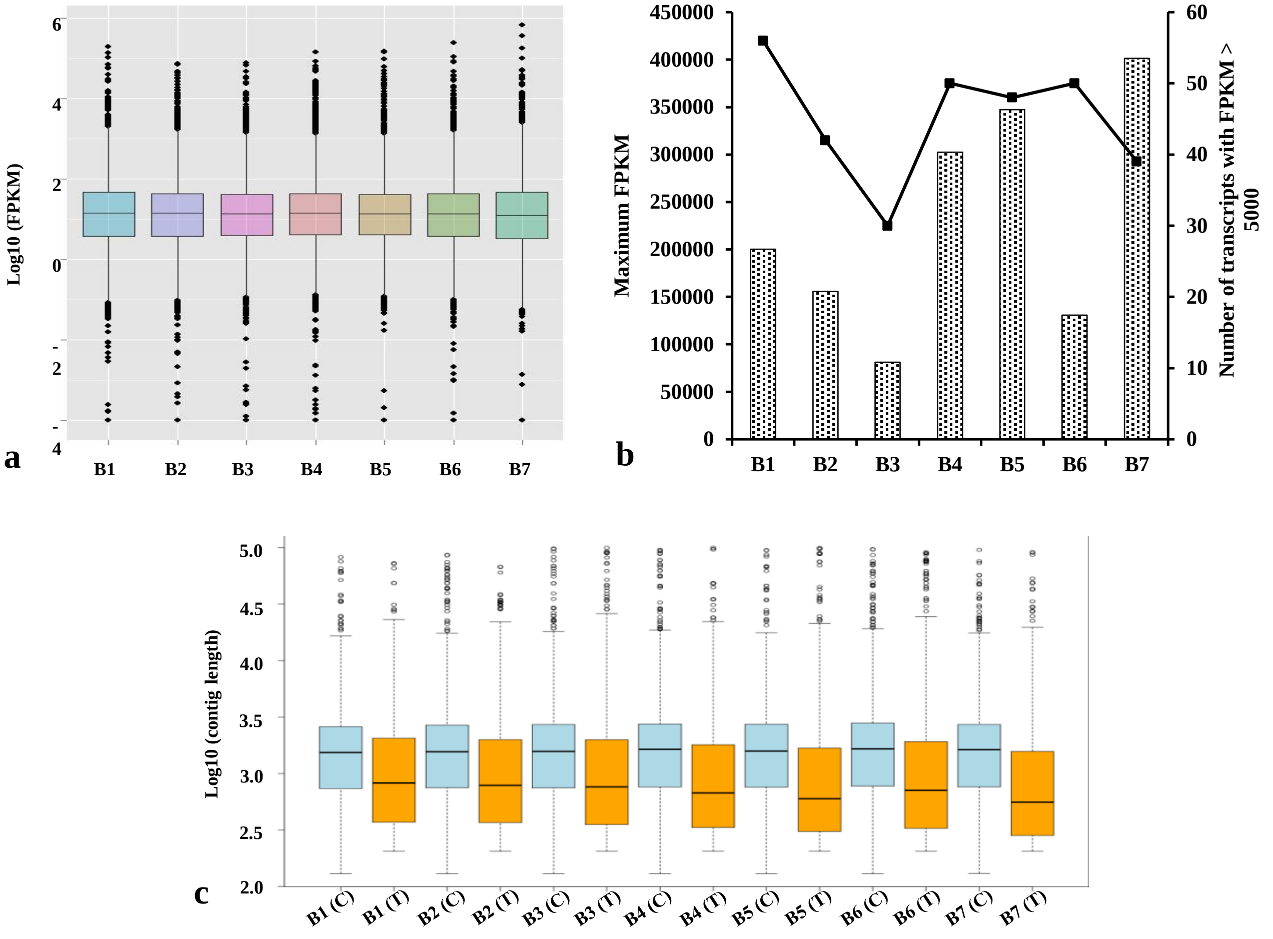

Supplementary Figure 6 A summary of RNA-Seq analysis. (a) A box-and whisker plot showing distribution of FPKMs. The top and bottom of each box correspond to the 75th percentile and the 25th percentile respectively, while the black line inside the box corresponds to the 50th percentile (the median). The whiskers (dotted lines) extend to the most extreme data point that is no more than 1.5 times the interquartile range from the box. The open circles correspond to the outliers that are outside the range of the whiskers. (b) Maximum FPKM for each sample. (c) A box-and whisker plot showing length distribution of assembled contigs for each sample. The top and bottom of each box correspond to the 75th percentile and the 25th percentile respectively, while the black line inside the box corresponds to the 50th percentile (the median). The whiskers (dotted lines) extend to the most extreme data point that is no more than 1.5 times the interquartile range from the box. The open circles correspond to the outliers that are outside the range of the whiskers. The blue boxes represent the Cufflinks assembled transcripts while orange boxes represent the Trinity assembled contigs. For the x-axis, C stands for the cufflinks, while T stands for Trinity.

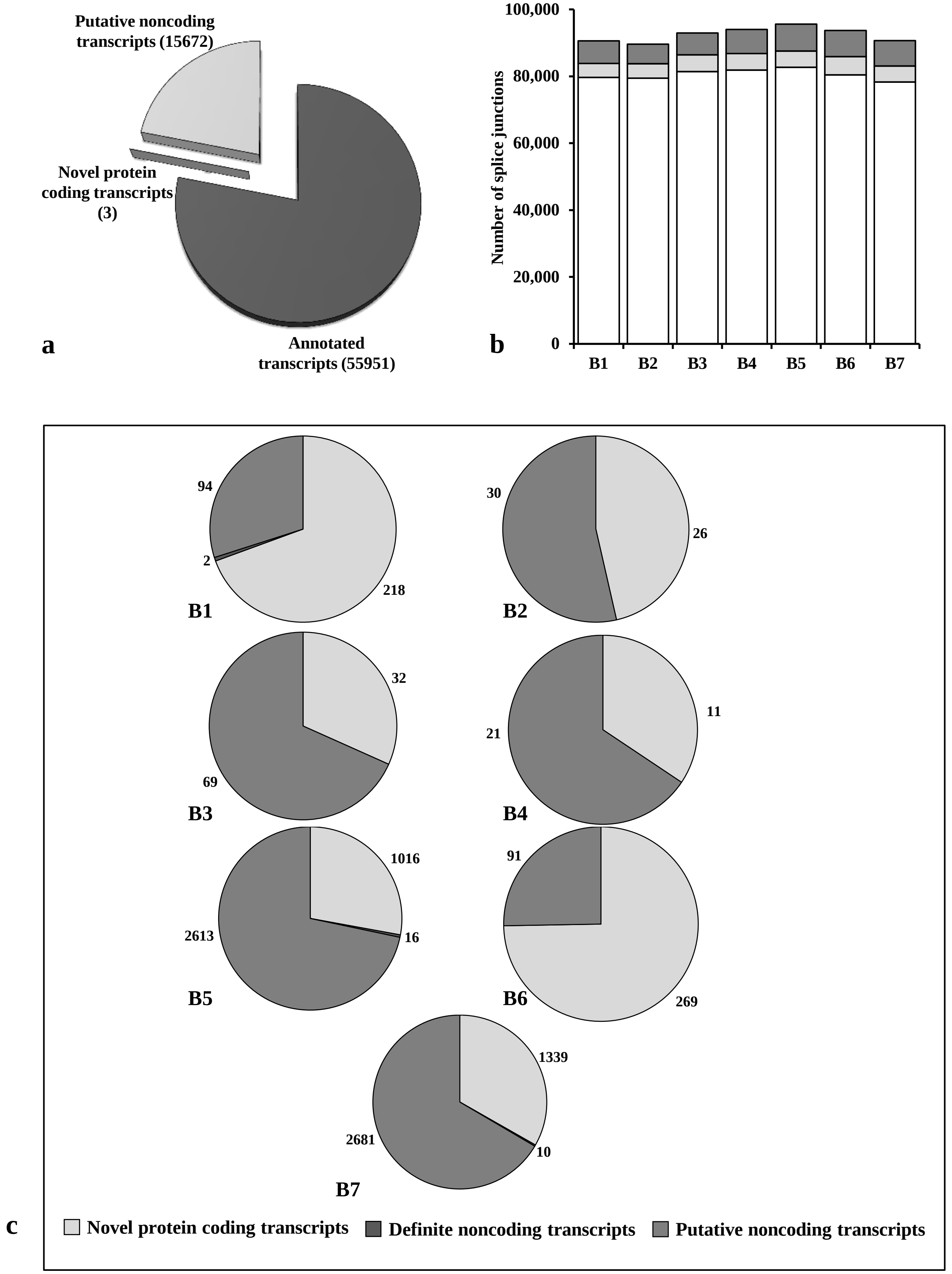

Supplementary Figure 7 Identification of novel transcribed region and splice junction. (a) Breakdown of the Cufflinks contigs into annotated, protein-coding and putative non-coding transcript. (b) Junction detected in each sample annotated in worm base (white portion), no. of novel junction (shaded portions). Novel junction supported by two or more non-redundant reads (nNR>) are shown in light grey and novel junction supported by only one non-redundant read (nNR=1) are shown in dark grey. (c) Breakdown of the unannotated Trinity contigs into protein-coding, definite non-coding, and putative non-coding transcripts in samples B1 to B7.

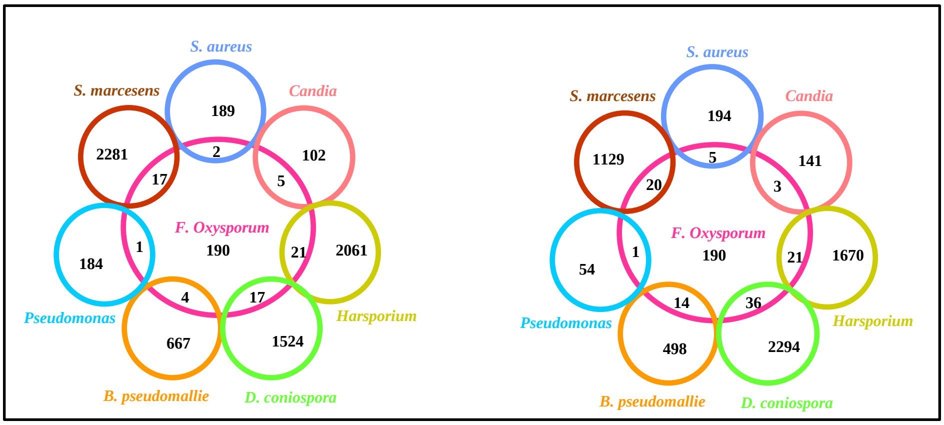

Supplementary Figure 8 Comparison of differentially expressed genes. Venn diagram depicting the common and uniquely up-regulated and down-regulated genes in worms infected with *F. oxysporum*, *S. aureus*, *S. marcesens*, *Candida*, *Harposporium*, *D. coniospora*, *B. pseudomallie*, *and Pseudomonas.*

Supplementary Table 1 Summary of RNA-Seq reads and mapping statistics with Cufflinks.

Sample Description	Read Length (bp)	Total Reads (millions)	Sequenced bases (Mb)	Aligned Reads (millions)	% Aligned Reads	Uniquely Mapped Reads (millions)	Uniquely Mapped bases	Reads with multiple matches
**B1**	101	40.3	4068.3	34.3	85.11166	28.8	2908.8	5.5
**B2**	101	35.9	36383.1	30.2	84.12256	26	2626	4.2
**B3**	101	38.9	3925.1	33	84.8329	28.6	2888.6	4.4
**B4**	101	42.8	4325.5	36.5	85.28037	30.6	3090.6	5.9
**B5**	101	46.2	4663.9	36.7	79.43723	30.6	3090.6	6.1
**B6**	101	41	4135.9	34.6	84.39024	29.7	2999.7	4.9
**B7**	101	39.3	3968.1	31.2	79.38931	27.3	2757.3	3.9
**Total**		284.4	61469.9	236.5	582.5643	201.6	20361.6	34.9

Supplementary Table 2 Discovery of new features.

Feature	Cufflinks	Trinity
Annotated transcripts	55951	231920
Novel Protein-coding transcripts	3	2,910
Novel Non-coding transcripts	15672	5599
Novel snRNA	-	2
Novel snoRNA	-	3
Novel tRNA	-	6

Supplementary Table 3 (a) List of novel protein coding transcript detected by RNA-Seq using Cufflinks.

Transcript_ID	Chromosome	Locus	Length
TCONS_00017396	III	WBGene00200776:168489-168766	278
TCONS_00061002	V	WBGene00199551:17629238-17629408	399
TCONS_00061003	V	WBGene00201191:17629472-17629673	171

Supplementary Table 3 (b) Novel transcripts and known protein domains identified from Trinity.

Novel transcripts	Number of Hits in InterPro	InterPro-IDs;InterPro-Descriptions					
comp88_c1_seq1	5	IPR1394;Peptidase C19, ubiquitin carboxyl-terminal hydrolase	PTHR246 (PANTHER); Peptidase C19, ubiquitin carboxyl-terminal hydrolase	PTHR246:SF22 (PANTHER);Peptidase C19, ubiquitin carboxyl-terminal hydrolase	IPR28889; Ubiquitin specific protease domain	SSF541 (SUPERFAMILY);Ubiquitin specific protease domain	
comp220_c1_seq1	2	IPR2293;Amino acid/polyamine transporter I	PTHR11785:SF29 (PANTHER); Amino acid/polyamine transporter I				
comp271_c0_seq1	4	G3DSA:3.3.45.2 (GENE3D);null	PTHR24423:SF462 (PANTHER); null	PTHR24423 (PANTHER);null	IPR14;PAS domain		
comp299_c0_seq1	6	PR853 (PRINTS);null	IPR686;XPG-I domain	IPR296;PIN domain-like	IPR144;XPG/Rad2 endonuclease, eukaryotes	PTHR1181 (PANTHER);XPG/Rad2 endonuclease, eukaryotes	IPR19974;XPG conserved site
comp303_c0_seq1	5	IPR1351;Ribosomal protein S3, C-terminal	IPR444;K Homology domain, type 2	PTHR1176:SF17 (PANTHER);K Homology domain, type 2	PTHR1176 (PANTHER);K Homology domain, type 2	IPR919;K homology domain, prokaryotic type	
comp339_c0_seq1	2	G3DSA:1.1.51.1 (GENE3D);null	IPR119;Protein kinase-like domain				
comp485_c0_seq1	1	IPR2712;Structural maintenance of chromosomes Smc2					
comp572_c0_seq1	3	IPR21131;Ribosomal protein L18e/L15P	PTHR11721 (PANTHER); Ribosomal protein L18e/L15P	PTHR11721:SF3 (PANTHER);Ribosomal protein L18e/L15P			
comp580_c0_seq1	1	IPR3656;Zinc finger, BED-type					
comp606_c0_seq1	2	PTHR247 (PANTHER);Zinc finger, RING-type	PTHR247:SF7 (PANTHER); Zinc finger, RING-type				
comp691_c0_seq1	1	IPR2179;Protein of unknown					

		function DUF3292					
comp935_c1_seq1	2	PTHR11439 (PANTHER);null	PTHR11439:SF137 (PANTHER);null				
comp969_c0_seq1	1	IPR1286;Arf3-interacting protein 1, N-terminal domain					
comp982_c0_seq1	2	IPR146;NRAMP family	PTHR1176:SF33 (PANTHER);NRAMP family				
comp1061_c0_seq1	1	IPR2999;Actin-related protein 1 family					
comp1118_c1_seq1	3	IPR1623;DnaJ domain	PTHR2476 (PANTHER);DnaJ domain	PTHR2476:SF18 (PANTHER);DnaJ domain			
comp1199_c0_seq1	1	PTHR13198 (PANTHER);Zinc finger, RING-type					
comp1226_c0_seq1	3	IPR2958;Alpha/Beta hydrolase fold	IPR73;Alpha/beta hydrolase fold-1	PTHR1992 (PANTHER);Alpha/beta hydrolase fold-1			
comp1227_c1_seq1	2	PTHR1796:SF35 (PANTHER);null	PTHR1796 (PANTHER);null				
comp1232_c0_seq1	2	IPR1932;Protein phosphatase 2C (PP2C)-like domain	IPR222;Protein phosphatase 2C, manganese/magnesium aspartate binding site				
comp1259_c0_seq1	2	PTHR2431:SF219 (PANTHER);DEAD/DEAH box helicase domain	IPR1414;RNA helicase, DEAD-box type, Q motif				
comp1277_c0_seq1	6	IPR11992;EF-hand domain pair	PF13833 (PFAM);EF-hand domain pair	PTHR2349 (PANTHER);EF-hand domain pair	PTHR2349:SF33 (PANTHER);EF-hand domain pair	IPR248;EF-hand domain	SSF47473 (SUPERFAMILY);EF-hand domain
comp1308_c1_seq1	3	G3DSA:1.1.61.1 (GENE3D);null	PTHR22747:SF16 (PANTHER);null	IPR431;Nucleoplasmin			
comp1473_c0_seq2	4	G3DSA:3.9.12.1 (GENE3D);null	IPR2575;Aminoglycoside phosphotransferase	IPR24165;Aminoglycoside 3-phosphotransferase	PTHR2131:SF3 (PANTHER);Aminoglycoside 3-phosphotransferase		
comp1510_c0_seq1	2	PTHR12642 (PANTHER);null	PTHR12642:SF (PANTHER);null				
comp1515_c0_seq1	3	IPR896;HAT dimerisation domain, C-terminal	PTHR23272 (PANTHER);HAT dimerisation domain, C-	IPR12337;Ribonuclease H-like domain			

			terminal				
comp1529_c0_seq1	2	IPR29229;Alkyl sulfatase, C-terminal	IPR333;SCP2 sterol-binding domain				
comp1529_c1_seq1	1	IPR29228;Alkyl sulfatase dimerisation domain					
comp1561_c1_seq1	1	IPR1878;Zinc finger, CCHC-type					
comp1575_c0_seq1	1	PTHR2473:SF353 (PANTHER);Small GTPase superfamily					
comp1638_c0_seq1	2	G3DSA:3.2.2.14 (GENE3D);null	SSF51556 (SUPERFAMILY);null				
comp1646_c0_seq1	5	IPR2125;CMP/dCMP deaminase, zinc-binding	G3DSA:3.4.14.1 (GENE3D);CMP/dCMP deaminase, zinc-binding	IPR15517;Deoxycytidylate deaminase-related	PTHR1186:SF4 (PANTHER);Deoxycytidylate deaminase-related	IPR16193;Cytidine deaminase-like	
comp1661_c0_seq1	3	IPR1752;Kinesin motor domain	IPR2764;Kinesin-like protein	PTHR24115:SF388 (PANTHER);Kinesin-like protein			
comp1698_c0_seq1	1	PTHR23244:SF249 (PANTHER);Jacalin-like lectin domain					
comp1766_c0_seq1	4	IPR569;HECT	G3DSA:3.3.216.1 (GENE3D);HECT	PTHR11254 (PANTHER);HECT	PTHR11254:SF294 (PANTHER);HECT		
comp1812_c0_seq1	3	IPR352;Peptide chain release factor class I/class II	PTHR1175 (PANTHER); Peptide chain release factor class I/class II	SSF7562 (SUPERFAMILY); Peptide chain release factor class I/class II			
comp1819_c0_seq1	1	PTHR2431:SF57 (PANTHER);P-loop containing nucleoside triphosphate hydrolase					
comp1904_c0_seq1	1	IPR2916;GAF domain-like					
comp1928_c0_seq1	6	G3DSA:3.1.2.1 (GENE3D);null	IPR23573;Ribosomal protein L18a/LX	PTHR152:SF6 (PANTHER);Ribosomal protein L18a/LX	PTHR152 (PANTHER);Ribosomal protein L18a/LX	IPR28877;5S ribosomal protein L18Ae/6S ribosomal protein L2 and L18a	SSF16374 (SUPERFAMILY);5S ribosomal protein L18Ae/6S ribosomal protein L2 and L18a
comp1946_c0_seq	2	PTHR1351:SF42 (PANTHER);null	PTHR1351 (PANTHER);				

1			null		
comp2012_c0_seq1	2	IPR64;Translation elongation factor EFG, V domain	IPR922;Elongation factor G, III-V domain		
comp2018_c0_seq1	3	IPR885;Fibrillar collagen, C-terminal	PTHR2423:SF54 (PANTHER);Fibrillar collagen, C-terminal	PTHR2423 (PANTHER);Fibrillar collagen, C-terminal	
comp2050_c0_seq1	2	PTHR24356:SF1 (PANTHER);null	PTHR24356 (PANTHER);null		
comp2074_c0_seq1	1	IPR1763;Rhodanese-like domain			
comp2138_c0_seq1	4	IPR23329;Chlorophyll a/b binding protein domain	IPR22796;Chlorophyll A-B binding protein	IPR1344;Chlorophyll A-B binding protein, plant	PTHR21649:SF9 (PANTHER);Chlorophyll A-B binding protein, plant
comp2238_c0_seq1	1	IPR15915;Kelch-type beta propeller			
comp2349_c0_seq1	1	PTHR11752 (PANTHER);null			
comp2430_c0_seq1	1	PF13521 (PFAM);null			
comp2514_c0_seq1	1	IPR182;GNAT domain			
comp2520_c0_seq1	4	IPR6111;Archaeal RpoK/eukaryotic RPB6 RNA polymerase subunit	IPR12293;RNA polymerase subunit, RPB6/omega	IPR611;RNA polymerase, subunit omega/K/RPB6	IPR278;DNA-directed RNA polymerase, 14-18kDa subunit, conserved site
comp2582_c0_seq1	4	IPR128;Mini-chromosome maintenance, DNA-dependent ATPase	PTHR1163 (PANTHER);Mini-chromosome maintenance, DNA-dependent ATPase	IPR85;DNA replication licensing factor Mcm7	IPR18525;Mini-chromosome maintenance, conserved site
comp2736_c0_seq1	3	IPR968;S15/NS1, RNA-binding	IPR589;Ribosomal protein S15	PD15743 (PRODOM);Ribosomal protein S15	
comp2790_c0_seq1	4	G3DSA:1.2.125.2 (GENE3D);null	PTHR243 (PANTHER);null	PTHR243:SF57 (PANTHER);null	IPR2846;Major facilitator superfamily domain
comp2809_c0_seq1	4	NON_CYTOPLASMIC_DOMAIN (PHOBIUS);null	TRANSMEMBRANE (PHOBIUS);null	CYTOPLASMIC_DOMAIN (PHOBIUS);null	TMhelix (TMHMM);null
comp3188_c0_seq1	1	IPR3256;Ribosomal protein L24			
comp3236_c0_seq	1	IPR2932;Glutamate synthase domain			

1						
comp3342_c0_seq1	4	IPR23173;NADPH-cytochrome p450 reductase, FAD-binding, alpha-helical domain-3	IPR397;FAD-binding, type 1	PTHR19384 (PANTHER);FAD-binding, type 1	PTHR19384:SF39 (PANTHER);FAD-binding, type 1	
comp3645_c0_seq1	4	IPR27417;P-loop containing nucleoside triphosphate hydrolase	PTHR2431 (PANTHER);P-loop containing nucleoside triphosphate hydrolase	PTHR2431:SF221 (PANTHER);P-loop containing nucleoside triphosphate hydrolase	IPR165;Helicase, C-terminal	
comp3659_c0_seq1	1	PTHR24199 (PANTHER);Ankyrin repeat-containing domain				
comp4202_c0_seq1	4	IPR6139;D-isomer specific 2-hydroxyacid dehydrogenase, catalytic domain	PTHR1996:SF91 (PANTHER);D-isomer specific 2-hydroxyacid dehydrogenase, catalytic domain	PTHR1996 (PANTHER);D-isomer specific 2-hydroxyacid dehydrogenase, catalytic domain	SSF52283 (SUPERFAMILY);D-isomer specific 2-hydroxyacid dehydrogenase, catalytic domain	
comp4211_c0_seq1	1	IPR583;Class II glutamine amidotransferase domain				
comp4217_c0_seq1	2	PTHR31944:SF16 (PANTHER);null	PTHR31944 (PANTHER);null			
comp4360_c0_seq1	1	IPR2968;Glyoxalase/Bleomycin resistance protein/Dihydroxybiphenyl dioxygenase				
comp4437_c0_seq1	3	IPR1757;P-type ATPase	IPR23214;HAD-like domain	PTHR2493:SF127 (PANTHER);HAD-like domain		
comp4523_c0_seq1	2	IPR1715;Calponin homology domain	PTHR11915 (PANTHER);Calponin homology domain			
comp4648_c0_seq1	5	IPR647;Glycosyl hydrolase, family 13, catalytic domain	IPR13781;Glycoside hydrolase, catalytic domain	PTHR1357:SF77 (PANTHER);Glycoside hydrolase, catalytic domain	IPR1592;Glycoside hydrolase, family 13	IPR17853;Glycoside hydrolase superfamily
comp5088_c0_seq1	4	IPR719;Protein kinase domain	PTHR24343 (PANTHER);Protein kinase domain	PTHR24343:SF124 (PANTHER);Protein kinase domain	IPR8271;Serine/threonine-protein kinase, active site	
comp5201_c0_seq1	3	IPR19134;Cactin, C-terminal	PTHR21737:SF4 (PANTHER);Cactin, C-terminal	PTHR21737 (PANTHER);Cactin, C-terminal		
comp5464_c0_seq1	1	PTHR11695:SF46 (PANTHER);Alcohol dehydrogenase superfamily, zinc-				

		type						
comp5542_c0_seq1	1	IPR21842;Protein of unknown function DUF3435						
comp5566_c0_seq1	1	IPR1196;Ribosomal protein L15, conserved site						
comp5573_c0_seq1	2	IPR3594;Histidine kinase-like ATPase, C-terminal domain	PTHR24423:SF429 (PANTHER); Histidine kinase-like ATPase, C-terminal domain					
comp5716_c0_seq1	7	IPR1389;ENTH domain	IPR11417;AP18 N-terminal homology (ANTH) domain	IPR14712;Phosphoinositide-binding clathrin adaptor, domain 2	IPR8942;ENTH/VHS	PTHR22951 (PANTHER);ENTH/VHS	PTHR22951:SF5 (PANTHER);ENTH/VHS	SSF899 (SUPERFAMILY); ENTH/VHS
comp5726_c0_seq1	2	IPR1563;Peptidase S1, serine carboxypeptidase	PTHR1182:SF28 (PANTHER); Peptidase S1, serine carboxypeptidase					
comp5759_c0_seq1	5	IPR259;Cold-shock protein, DNA-binding	IPR11129;Cold shock protein	IPR12156;Cold shock, CspA	PTHR11544 (PANTHER);Cold shock, CspA	IPR19844;Cold-shock conserved site		
comp5783_c0_seq1	1	IPR961;AGC-kinase, C-terminal						
comp5889_c0_seq1	2	PTHR24423:SF41 (PANTHER);Signal transduction histidine kinase EnvZ-like, dimerisation/phosphoacceptor domain	IPR982;Signal transduction histidine kinase, homodimeric domain					
comp5939_c0_seq1	1	PTHR11711:SF151 (PANTHER);Small GTPase superfamily, ARF/SAR type						
comp5966_c1_seq1	1	PTHR2489:SF197 (PANTHER);Mitochondrial carrier domain						
comp6008_c1_seq1	1	PTHR11695:SF451 (PANTHER);Alcohol dehydrogenase superfamily, zinc-type						
comp6038_c0_seq1	1	IPR12511;S-adenosyl-l-methionine decarboxylase leader peptide						
comp6113_c1_seq1	1	IPR1394;Transcriptional regulatory protein RXT2, N-						

		terminal					
comp6293_c0_seq1	3	IPR211;Ankyrin repeat	IPR2683;Ankyrin repeat-containing domain	PTHR24198 (PANTHER);Ankyrin repeat-containing domain			
comp6363_c0_seq1	2	PTHR2121 (PANTHER);null	PTHR2121:SF (PANTHER); null				
comp6732_c0_seq1	6	Coil (COILS);null	IPR2957;Keratin, type I	IPR1664;Intermediate filament protein	PTHR23239:SF17 (PANTHER);Intermediate filament protein	SSF9257 (SUPERFAMILY);Intermediate filament protein	SSF64593 (SUPERFAMILY);Intermediate filament protein
comp6798_c1_seq1	1	PTHR11699:SF128 (PANTHER);Aldehyde dehydrogenase N-terminal domain					
comp6819_c1_seq1	4	IPR1451;Bacterial transferase hexapeptide repeat	G3DSA:2.16.1.1 (GENE3D);Bacterial transferase hexapeptide repeat	IPR27777;Dynactin subunit 6	IPR114;Trimeric LpxA-like		
comp7129_c0_seq1	3	IPR12951;Berberine/berberine-like	PTHR13878:SF38 (PANTHER); Berberine/berberine-like	PTHR13878 (PANTHER);Berberine/berberine-like			
comp7162_c0_seq1	5	IPR1856;Protein of unknown function DUF1479	IPR27443;Isopenicillin N synthase-like	PTHR3613:SF1 (PANTHER);Isopenicillin N synthase-like	PTHR3613 (PANTHER);Isopenicillin N synthase-like	SSF51197 (SUPERFAMILY);Isopenicillin N synthase-like	
comp7284_c0_seq1	1	SignalP-noTM (SIGNALP_EUK);null					
comp7354_c0_seq1	4	IPR95;Gcp-like domain	PTHR11735:SF6 (PANTHER); Gcp-like domain	PTHR11735 (PANTHER);Gcp-like domain	SSF5367 (SUPERFAMILY);Gcp-like domain		
comp7417_c0_seq1	3	IPR4358;Signal transduction histidine kinase-related protein, C-terminal	PTHR24423:SF437 (PANTHER); Histidine kinase-like ATPase, C-terminal domain	IPR5467;Signal transduction histidine kinase, core			
comp7487_c0_seq1	3	IPR21151;GINS complex	IPR8591;GINS complex, subunit Sld5	SSF158573 (SUPERFAMILY); GINS complex, subunit Sld5			
comp7734_c0_seq1	3	IPR121;Ribosomal protein S17e	PTHR1732:SF (PANTHER); Ribosomal protein S17e	IPR18273;Ribosomal protein S17e, conserved site			
comp7804_c0_seq1	3	IPR1313;Ferric reductase transmembrane component-like	PTHR32361 (PANTHER); Ferric reductase	PTHR32361:SF1 (PANTHER);Ferric reductase transmembrane			

		domain	transmembrane component-like domain	component-like domain		
comp7931_c0_seq1	1	IPR2921;Protein-tyrosine phosphatase-like				
comp7948_c0_seq1	1	PTHR2493:SF232 (PANTHER);P-type ATPase				
comp8090_c0_seq1	4	IPR1825;VHS subgroup	IPR214;VHS	PTHR13856 (PANTHER);VHS	PTHR13856:SF43 (PANTHER);VHS	
comp8264_c0_seq1	2	PTHR11384 (PANTHER);null	PTHR11384:SF2 (PANTHER);null			
comp8386_c0_seq1	5	IPR6186;Serine/threonine-specific protein phosphatase/bis(5-nucleosyl)-tetraphosphatase	IPR2952;Metallo-dependent phosphatase-like	IPR4843;Calcineurin-like phosphoesterase domain, apaH type	PTHR11668 (PANTHER);Calcineurin-like phosphoesterase domain, apaH type	PTHR11668:SF25 (PANTHER);Calcineurin-like phosphoesterase domain, apaH type
comp8556_c0_seq1	3	IPR591;DEP domain	IPR11991;Winged helix-turn-helix DNA-binding domain	SSF46785 (SUPERFAMILY);DEP domain		
comp8681_c0_seq1	5	IPR2641;Patatin/Phospholipase A2-related	G3DSA:3.4.19.1 (GENE3D);Patatin/Phospholipase A2-related	PTHR1622 (PANTHER);Patatin/Phospholipase A2-related	PTHR1622:SF7 (PANTHER);Patatin/Phospholipase A2-related	IPR1635;Acyl transferase/acyl hydrolase/lysophospholipase
comp8683_c0_seq1	5	IPR6689;Small GTPase superfamily, ARF/SAR type	IPR6687;Small GTPase superfamily, SAR1-type	IPR24156;Small GTPase superfamily, ARF type	PTHR11711:SF26 (PANTHER);Small GTP-binding protein domain	PTHR11711 (PANTHER);Small GTP-binding protein domain
comp8744_c0_seq1	3	IPR13332;Ketopantoate reductase, N-terminal domain	PTHR2178 (PANTHER);Ketopantoate reductase, N-terminal domain	PTHR2178:SF25 (PANTHER);Ketopantoate reductase, N-terminal domain		
comp8798_c0_seq1	5	IPR2347;Glucose/ribitol dehydrogenase	IPR2198;Short-chain dehydrogenase/reductase SDR	PTHR24322:SF319 (PANTHER);Short-chain dehydrogenase/reductase SDR	PTHR24322 (PANTHER);Short-chain dehydrogenase/reductase SDR	IPR294;Short-chain dehydrogenase/reductase, conserved site
comp8805_c0_seq1	2	PTHR2117 (PANTHER);null	PTHR2117:SF2 (PANTHER);null			
comp8813_c0_seq1	4	G3DSA:3.3.42.4 (GENE3D);null	IPR18484;Carbohydrate kinase, FGGY, N-terminal	PTHR1196 (PANTHER);Carbohydrate kinase, FGGY, N-terminal	PTHR1196:SF57 (PANTHER);Carbohydrate	

					kinase, FGGY, N-terminal	
comp8844_c0_seq1	5	IPR223;NADH-quinone oxidoreductase subunit E-like	IPR12336;Thioredoxin-like fold	PF1257 (PFAM);Thioredoxin-like fold	PTHR1371 (PANTHER);Thioredoxin-like fold	PTHR1371:SF3 (PANTHER);Thioredoxin-like fold
comp8929_c0_seq1	3	IPR1816;Heterokaryon incompatibility Het-C	PTHR1495:SF7 (PANTHER);Heterokaryon incompatibility Het-C	PTHR1495 (PANTHER);Heterokaryon incompatibility Het-C		
comp8967_c0_seq1	1	PTHR1188:SF8 (PANTHER);Ribosomal protein S19/S15				
comp9003_c0_seq1	2	IPR8854;TPMT family	PTHR118:SF774 (PANTHER);S-adenosyl-L-methionine-dependent methyltransferase			
comp9019_c0_seq1	2	G3DSA:3.4.22.1 (GENE3D);null	SSF52949 (SUPERFAMILY);null			
comp9029_c0_seq1	3	IPR2776;Mitochondrial PGP phosphatase	PTHR19288 (PANTHER);Mitochondrial PGP phosphatase	PTHR19288:SF25 (PANTHER);Mitochondrial PGP phosphatase		
comp9181_c0_seq1	3	IPR969;Cysteine alpha-hairpin motif superfamily	PTHR13523 (PANTHER);Cysteine alpha-hairpin motif superfamily	PTHR13523:SF2 (PANTHER);Cysteine alpha-hairpin motif superfamily		
comp9184_c0_seq1	3	IPR2616;tRNA-guanine(15) transglycosylase-like	IPR483;Queuine tRNA-ribosyltransferase	PTHR11962:SF6 (PANTHER);tRNA-guanine(15) transglycosylase-like		
comp9205_c0_seq1	4	IPR1353;Proteasome, subunit alpha/beta	PTHR11599:SF6 (PANTHER);Nucleophile aminohydrolases, N-terminal	IPR165;Proteasome beta-type subunit, conserved site	IPR23333;Proteasome B-type subunit	
comp9294_c0_seq1	4	IPR7715;Ubiquinone biosynthesis protein Coq4	PTHR12922 (PANTHER);Ubiquinone biosynthesis protein Coq4	PTHR12922:SF7 (PANTHER);Ubiquinone biosynthesis protein Coq4	IPR2754;Ubiquinone biosynthesis protein Coq4, eukaryotes	
comp9304_c1_seq1	3	IPR1619;Sec1-like protein	IPR27482;Sec1-like, domain 2	PTHR11679:SF2 (PANTHER);Sec1-like protein		
comp9362_c1_seq1	1	IPR18545;Btz domain				
comp9552_c0_seq1	2	PTHR12126 (PANTHER);NAD(P)-binding domain	PTHR12126:SF2 (PANTHER);NAD(P)-binding domain			

comp9554_c0_seq1	1	PTHR11599:SF49 (PANTHER);Nucleophile aminohydrolases, N-terminal					
comp9561_c1_seq1	3	IPR845;Nucleoside phosphorylase domain	IPR1817;Nucleoside phosphorylase	PTHR21234:SF16 (PANTHER);Nucleoside phosphorylase			
comp9599_c0_seq1	6	PR1166 (PRINTS);null	IPR8972;Cupredoxin	IPR2429;Cytochrome c oxidase subunit II C-terminal	PTHR22888 (PANTHER);Cytochrome c oxidase subunit II C-terminal	PTHR22888:SF2 (PANTHER);Cytochrome c oxidase subunit II C-terminal	IPR155;Copper centre Cu(A)
comp9607_c0_seq1	3	IPR13845;Ribosomal protein S4e, central region	PTHR11581:SF7 (PANTHER);Ribosomal protein S4e, central region	IPR876;Ribosomal protein S4e			
comp9607_c1_seq1	4	SIGNAL_PEPTIDE_H_REGION (PHOBIUS);null	SIGNAL_PEPTIDE_N_REGION (PHOBIUS);null	SIGNAL_PEPTIDE_C_REGION (PHOBIUS);null	SIGNAL_PEPTIDE (PHOBIUS);null		
comp9707_c0_seq1	1	IPR4123;mRNA splicing factor, thioredoxin-like U5 snRNP					
comp9778_c0_seq1	2	IPR866;Alkyl hydroperoxide reductase subunit C/ Thiol specific antioxidant	PTHR1681 (PANTHER);Alkyl hydroperoxide reductase subunit C/ Thiol specific antioxidant				
comp9813_c0_seq1	1	PTHR11998 (PANTHER);AP complex, mu/sigma subunit					
comp9832_c0_seq2	2	IPR3397;Mitochondrial inner membrane translocase subunit Tim17/Tim22/Tim23/peroxisomal protein PMP24	PTHR1546 (PANTHER);Mitochondrial inner membrane translocase subunit Tim17/Tim22/Tim23/peroxisomal protein PMP24				
comp9834_c0_seq1	1	IPR8949;Isoprenoid synthase domain					
comp9836_c0_seq2	1	IPR4365;OB-fold nucleic acid binding domain, AA-tRNA synthetase-type					
comp9901_c0_seq1	3	IPR2677;Ribosomal protein L32p	PTHR2126 (PANTHER);Ribosomal protein L32p	PTHR2126:SF2 (PANTHER);Ribosomal protein L32p			
comp9914_c0_seq1	5	IPR5198;Glycoside hydrolase, family 76	IPR12341;Six-hairpin glycosidase	PTHR12145 (PANTHER);Six-hairpin glycosidase	PTHR12145:SF16 (PANTHER);Six-	IPR8928;Six-hairpin glycosidase-like	

					hairpin glycosidase			
comp9925_c0_seq1	1	IPR7741;Ribosomal protein/NADH dehydrogenase domain						
comp9957_c0_seq1	2	IPR5326;Plectin/S1, N-terminal	PTHR12146 (PANTHER);Plectin/S1, N-terminal					
comp10034_c0_seq1	4	IPR1897;Porin, gammaproteobacterial	IPR172;Porin, Gram-negative type	IPR23614;Porin domain	SSF56935 (SUPERFAMILY);Porin domain			
comp10054_c0_seq1	5	IPR4834;Fungal chitin synthase	IPR13616;Chitin synthase N-terminal	IPR4835;Chitin synthase	PTHR22914:SF11 (PANTHER);Chitin synthase	IPR2944;Nucleotide-diphospho-sugar transferases		
comp10088_c0_seq1	2	PTHR3151 (PANTHER);null	PTHR3151:SF1 (PANTHER);null					
comp10104_c0_seq1	6	IPR673;GTP binding domain	IPR495;TGS	PTHR1172 (PANTHER);P-loop containing nucleoside triphosphate hydrolase	IPR674;GTP1/OBG, conserved site	PS5171 (PROSITE_PROFILES);GTP1/OBG, conserved site	IPR12676;TGS-like	
comp10136_c0_seq1	3	IPR2312;Aspartyl/Asparaginyl-tRNA synthetase, class IIb	IPR4523;Aspartyl-tRNA synthetases	IPR6195;Aminoacyl-tRNA synthetase, class II				
comp10165_c0_seq1	6	IPR1754;Orotidine 5'-phosphate decarboxylase domain	IPR13785;Aldolase-type TIM barrel	PTHR19278:SF9 (PANTHER);Orotidine 5'-phosphate decarboxylase domain	PTHR19278 (PANTHER);Orotidine 5'-phosphate decarboxylase domain	IPR1889;Orotidine 5'-phosphate decarboxylase, active site	IPR116;Ribulose-phosphate binding barrel	
comp10166_c0_seq1	2	IPR26847;Vacuolar protein sorting-associated protein 13	PTHR16166:SF93 (PANTHER);Vacuolar protein sorting-associated protein 13					
comp10285_c0_seq1	2	IPR19591;Mrp/NBP35 ATP-binding protein	IPR88;Mrp, conserved site					
comp10368_c0_seq1	7	IPR2867;Zinc finger, C6HC-type	IPR1841;Zinc finger, RING-type	IPR1383;Zinc finger, RING/FYVE/PHD-type	PTHR11685 (PANTHER);Zinc finger, C6HC-type	PTHR11685:SF1 (PANTHER);Zinc finger, C6HC-type	IPR17907;Zinc finger, RING-type, conserved site	SSF5785 (SUPERFAMILY);Zinc finger, RING-type
comp10382_c0_seq1	1	IPR1822;Adenylosuccinate synthase, active site						
comp10392_c1_seq1	3	IPR19315;Kinase phosphorylation domain	PTHR1458 (PANTHER);Kinase phosphorylation domain	PTHR1458:SF (PANTHER);Kinase phosphorylation domain				

comp10400_c0_seq1	2	IPR1578;Peptidase C12, ubiquitin carboxyl-terminal hydrolase	PTHR1589:SF16 (PANTHER);Peptidase C12, ubiquitin carboxyl-terminal hydrolase					
comp10439_c0_seq1	2	PTHR14269:SF14 (PANTHER);HAD-like domain	PTHR14269 (PANTHER);HAD-like domain					
comp10471_c1_seq1	3	IPR229;Serine/threonine/dual specificity protein kinase, catalytic domain	PTHR2455 (PANTHER);Protein kinase domain	PTHR2455:SF183 (PANTHER);Protein kinase domain				
comp10493_c1_seq1	3	IPR18392;LysM domain	PTHR21666:SF182 (PANTHER);LysM domain	PTHR21666 (PANTHER);LysM domain				
comp10608_c0_seq1	2	PTHR3164 (PANTHER);null	PTHR3164:SF2 (PANTHER);null					
comp10671_c0_seq1	1	IPR18485;Carbohydrate kinase, FGGY, C-terminal						
comp10711_c0_seq1	3	IPR1397;Stress responsive alpha-beta barrel	G3DSA:3.3.7.9 (GENE3D);Stress responsive alpha-beta barrel	IPR118;Dimeric alpha-beta barrel				
comp10745_c1_seq1	4	IPR4148;BAR domain	IPR27267;Arfaptin homology (AH) domain/BAR domain	PTHR1321 (PANTHER);Arfaptin homology (AH) domain/BAR domain	SSF13657 (SUPERFAMILY);BAR domain			
comp10838_c0_seq1	1	PTHR11668:SF27 (PANTHER);Calcineurin-like phosphoesterase domain, apaH type						
comp10852_c0_seq1	2	PTHR325:SF31 (PANTHER);Zn(2)-C6 fungal-type DNA-binding domain	PTHR325 (PANTHER);Zn(2)-C6 fungal-type DNA-binding domain					
comp10964_c0_seq1	7	IPR14758;Methionyl-tRNA synthetase	IPR14729;Rossmann-like alpha/beta/alpha sandwich fold	IPR15413;Methionyl/Leucyl tRNA synthetase	G3DSA:2.17.22.1 (GENE3D);Methionyl/Leucyl tRNA synthetase	PTHR11946:SF63 (PANTHER);Methionyl/Leucyl tRNA synthetase	PTHR11946 (PANTHER);Methionyl/Leucyl tRNA synthetase	SSF52374 (SUPERFAMILY);Methionyl/Leucyl tRNA synthetase
comp10971_c0_seq1	1	IPR9554;Phage shock protein, PspB						
comp10971_c1_seq1	3	IPR7157;PspA/IM3	PTHR3188 (PANTHER);PspA/IM3	PTHR3188:SF (PANTHER);PspA/IM3				
comp11069_c0_seq2	1	PTHR2473:SF335 (PANTHER);Small GTPase						

			superfamily		
comp11238_c0_seq1	2		IPR1115;Cutinase, monofunctional	IPR675;Cutinase	
comp11306_c0_seq1	1		IPR59;Ribosomal protein L36e		
comp11319_c0_seq1	3		IPR683;Oxidoreductase, N-terminal	PTHR2264 (PANTHER); Oxidoreductase, N-terminal	PTHR2264:SF15 (PANTHER);Oxidoreductase, N-terminal
comp11503_c0_seq1	2		PTHR1121:SF (PANTHER);null	IPR2759;Coatomer delta subunit	
comp11539_c0_seq1	2		IPR1614;Bifunctional inhibitor/plant lipid transfer protein/seed storage helical domain	G3DSA:1.1.11.1 (GENE3D);Bifunctional inhibitor/plant lipid transfer protein/seed storage helical domain	
comp11589_c1_seq1	1		IPR16163;Aldehyde dehydrogenase, C-terminal		
comp11634_c0_seq1	3		IPR741;Fructose-bisphosphate aldolase, class-I	PTHR11627:SF7 (PANTHER); Aldolase-type TIM barrel	IPR29769;Fructose-bisphosphate aldolase class-I, eukaryotic-type
comp11658_c0_seq1	2		IPR4217;Tim1/DDP family zinc finger	PTHR21535 (PANTHER); Tim1/DDP family zinc finger	
comp11773_c0_seq1	3		PF13646 (PFAM);null	PTHR1648 (PANTHER); Armadillo-like helical	IPR21133;HEAT, type 2
comp11785_c0_seq1	3		IPR7123;Gelsolin-like domain	PTHR11141 (PANTHER); ADF-H/Gelsolin-like domain	SSF82754 (SUPERFAMILY); ADF-H/Gelsolin-like domain
comp11785_c1_seq1	1		IPR69;Sec23/Sec24, helical domain		
comp11787_c0_seq1	3		IPR5828;General substrate transporter	PTHR2463:SF297 (PANTHER); General substrate transporter	PTHR2463 (PANTHER);General substrate transporter
comp11796_c0_seq1	1		PF12756 (PFAM);Zinc finger, C2H2-like		
comp11851_c0_seq1	3		IPR1984;Stm1, N-terminal	IPR6861;Hyaluronan/mRNA-binding protein	PTHR12299 (PANTHER);Hyaluronan/mRNA-binding protein
comp11867_c0_seq1	3		IPR15285;RIO2 kinase winged helix domain, N-terminal	PTHR1593 (PANTHER); Winged helix-turn-helix DNA-binding domain	IPR3484;Serine/threonine-protein kinase Rio2

comp11875_c0_seq1	1	IPR8386;ATPase, F complex, subunit E, mitochondrial						
comp11896_c1_seq1	2	PR45 (PRINTS);null	IPR28846;Recoverin family					
comp11940_c0_seq1	1	IPR2962;Class I glutamine amidotransferase-like						
comp12045_c0_seq1	7	IPR1635;Mannose-6-phosphate isomerase	G3DSA:1.1.441.1 (GENE3D);Mannose-6-phosphate isomerase	IPR1471;RmlC-like jelly roll fold	IPR125;Mannose-6-phosphate isomerase, type I	PTHR139:SF (PANTHER);Mannose-6-phosphate isomerase	IPR185; Phosphomannose isomerase, type I, conserved site	IPR1151; RmlC-like cupin domain
comp12207_c0_seq1	2	IPR19956;Ubiquitin	IPR19954;Ubiquitin conserved site					
comp12210_c0_seq1	1	IPR25959;Winged helix-turn helix domain						
comp12295_c0_seq1	2	PTHR13947 (PANTHER);GNAT domain	PTHR13947:SF13 (PANTHER); GNAT domain					
comp12316_c0_seq1	1	IPR181;F-box domain						
comp12399_c0_seq1	2	IPR183;Copper fist DNA-binding	SM19 (SMART);Copper fist DNA-binding					
comp12436_c0_seq1	1	PTHR31944:SF27 (PANTHER);Transcription factor domain, fungi						
comp12494_c0_seq1	1	PTHR23115:SF97 (PANTHER);Elongation factor, GTP-binding domain						
comp12497_c0_seq1	1	IPR22272;Lipocalin family conserved site						
comp12569_c1_seq1	4	IPR11527;ABC transporter type 1, transmembrane domain	G3DSA:1.2.156.1 (GENE3D);ABC transporter type 1, transmembrane domain	PTHR24221 (PANTHER);ABC transporter type 1, transmembrane domain	PTHR24221:SF138 (PANTHER);ABC transporter type 1, transmembrane domain			
comp12598_c0_seq1	3	IPR267;Mitochondrial carrier protein	IPR2113;Adenine nucleotide translocator 1	PTHR2489:SF156 (PANTHER);Mitochondrial substrate/solute carrier				
comp12623_c1_seq1	3	IPR19559;Cullin protein, neddylation domain	PTHR11932:SF27 (PANTHER); Cullin protein, neddylation domain	PTHR11932 (PANTHER);Cullin protein, neddylation domain				
comp12693_c1_seq1	2	IPR164;Histone H3/CENP-A	IPR972;Histone-fold					

comp12732_c0_seq1	1	IPR18996;Inner nuclear membrane protein MAN1			
comp12736_c0_seq1	2	IPR23213;Chloramphenicol acetyltransferase-like domain	IPR348;Transferase		
comp12803_c0_seq1	4	IPR2384;Osteocalcin/matrix Gla protein	IPR294;Gamma-carboxyglutamic acid-rich (GLA) domain	IPR27118;Matrix Gla protein	PTHR119:SF (PANTHER);Matrix Gla protein
comp12873_c0_seq1	1	IPR271;Dilute			
comp12903_c1_seq1	3	IPR4813;Oligopeptide transporter, OPT superfamily	PTHR2261:SF5 (PANTHER);Oligopeptide transporter, OPT superfamily	PTHR2261 (PANTHER);Oligopeptide transporter, OPT superfamily	
comp12960_c1_seq1	4	G3DSA:3.3.154.1 (GENE3D);null	IPR2366;CoA-transferase family III domain	IPR3673;CoA-transferase family III	PTHR11837:SF6 (PANTHER);CoA-transferase family III
comp13037_c0_seq1	2	PTHR24345:SF (PANTHER);Protein kinase domain	PTHR24345 (PANTHER);Protein kinase domain		
comp13069_c0_seq1	2	IPR7133;RNA polymerase II-associated, Paf1	PTHR23188:SF12 (PANTHER);RNA polymerase II-associated, Paf1		
comp13152_c0_seq1	3	IPR1613;Pescadillo	IPR1357;BRCT domain	PTHR12221 (PANTHER);BRCT domain	
comp13181_c0_seq1	1	IPR7667;Hypoxia induced protein, domain			
comp13219_c1_seq1	1	PTHR21666:SF2 (PANTHER);null			
comp13230_c0_seq1	4	IPR2769;Translation initiation factor IF6	G3DSA:3.75.1.1 (GENE3D);Translation initiation factor IF6	PTHR1784:SF (PANTHER);Translation initiation factor IF6	SSF5599 (SUPERFAMILY);Translation initiation factor IF6
comp13331_c0_seq1	3	IPR12591;PRO8NT domain	PTHR1114:SF (PANTHER);PRO8NT domain	IPR27652;Pre-mRNA-processing-splicing factor 8	
comp13346_c0_seq1	2	PTHR2456:SF158 (PANTHER);null	PTHR2456 (PANTHER);null		
comp13408_c0_seq1	2	PTHR19876:SF1 (PANTHER);WD4/YVTN repeat-like-containing domain	PTHR19876 (PANTHER);WD4/YVTN repeat-like-containing domain		
comp13409_c0_se	2	PTHR334:SF2 (PANTHER);null	PTHR334 (PANTHER);		

q1			null		
comp13599_c0_seq1	4	IPR66;HTH CenpB-type DNA-binding domain	IPR4875;DDE superfamily endonuclease, CENP-B-like	PTHR1933 (PANTHER);DDE superfamily endonuclease, CENP-B-like	PTHR1933:SF223 (PANTHER);DDE superfamily endonuclease, CENP-B-like
comp13602_c0_seq1	1	IPR21827;Nucleoporin Nup186/Nup192/Nup25			
comp13639_c0_seq1	3	PTHR23293 (PANTHER);Rossmann-like alpha/beta/alpha sandwich fold	PTHR23293:SF9 (PANTHER);Rossmann-like alpha/beta/alpha sandwich fold	SSF5242 (SUPERFAMILY);Rossmann-like alpha/beta/alpha sandwich fold	
comp13700_c0_seq1	1	IPR4882;Luc7-related			
comp13717_c0_seq1	1	PTHR2318:SF22 (PANTHER);Arf GTPase activating protein			
comp13720_c0_seq1	2	PTHR16631 (PANTHER);Glycoside hydrolase, catalytic domain	PTHR16631:SF4 (PANTHER);Glycoside hydrolase, catalytic domain		
comp13734_c0_seq1	3	IPR43;Phosphatidylinositol 3-/4-kinase, catalytic domain	G3DSA:3.3.11.1 (GENE3D);Phosphatidylinositol 3-/4-kinase, catalytic domain	PTHR11139:SF55 (PANTHER);Phosphatidylinositol 3-/4-kinase, catalytic domain	
comp13809_c0_seq1	3	IPR13763;Cyclin-like	IPR2781;CyclinH/Ccl1	IPR15429;Cyclin C/H/T/L	
comp13809_c1_seq1	1	PTHR12197:SF144 (PANTHER);null			
comp13817_c0_seq1	4	G3DSA:3.9.83.1 (GENE3D);null	G3DSA:1.25.4.6 (GENE3D);Sec1-like, domain 2	G3DSA:3.4.5.26 (GENE3D);Sec1-like protein	PTHR11679:SF3 (PANTHER);Sec1-like protein
comp13852_c0_seq1	4	IPR2933;Histidine phosphatase superfamily	IPR1378;Histidine phosphatase superfamily, clade-1	PTHR2329:SF12 (PANTHER);Histidine phosphatase superfamily, clade-1	PTHR2329 (PANTHER);Histidine phosphatase superfamily, clade-1
comp13922_c0_seq1	2	IPR614;D-isomer specific 2-hydroxyacid dehydrogenase, NAD-binding domain	PTHR1996:SF16 (PANTHER);D-isomer specific 2-hydroxyacid dehydrogenas		

			e, NAD-binding domain				
comp14080_c0_seq1	1	PF13374 (PFAM);Tetratricopeptide-like helical domain					
comp14081_c0_seq1	2	IPR3958;Transcription factor CBF/NF-Y/archaeal histone	PTHR1164 (PANTHER);Histone-fold				
comp14180_c0_seq1	1	IPR1961;Oxoglutarate/iron-dependent oxygenase, C-terminal degradation domain					
comp14198_c1_seq1	2	IPR2136;Ribosomal protein L4/L1e	IPR23574;Ribosomal protein L4 domain				
comp14301_c1_seq1	5	IPR1214;SET domain	IPR2893;Zinc finger, MYND-type	G3DSA:2.17.27.1 (GENE3D);Zinc finger, MYND-type	PTHR12197 (PANTHER);Zinc finger, MYND-type	SSF82199 (SUPERFAMILY);Zinc finger, MYND-type	
comp14378_c1_seq1	5	IPR22;Carboxyl transferase	IPR2945;ClpP/crotonase-like domain	PTHR22855 (PANTHER);ClpP/crotonase-like domain	PTHR22855:SF13 (PANTHER);ClpP/crotonase-like domain	IPR11762;Acetyl-coenzyme A carboxyltransferase, N-terminal	
comp14390_c0_seq1	2	IPR2784;Ribosomal protein L11, N-terminal	PTHR11661:SF2 (PANTHER);Ribosomal protein L11/L12				
comp14505_c0_seq1	6	IPR8183;Aldose 1-/Glucose-6-phosphate 1-epimerase	IPR14718;Glycoside hydrolase-type carbohydrate-binding, subgroup	IPR25532;Glucose-6-phosphate 1-epimerase	PTHR11122 (PANTHER);Glucose-6-phosphate 1-epimerase	PTHR11122:SF13 (PANTHER);Glucose-6-phosphate 1-epimerase	IPR1113;Galactose mutarotase-like domain
comp14508_c1_seq1	2	PTHR2412 (PANTHER);Nucleotide-binding alpha-beta plait domain	PTHR2412:SF (PANTHER);Nucleotide-binding alpha-beta plait domain				
comp14634_c0_seq1	3	PTHR1937 (PANTHER);null	PTHR1937:SF63 (PANTHER);null	IPR17938;Riboflavin synthase-like beta-barrel			
comp14768_c0_seq1	3	IPR2483;PWI domain	PTHR14398:SF (PANTHER);PWI domain	PTHR14398 (PANTHER);PWI domain			
comp15005_c0_seq1	3	IPR198;Longin domain	PTHR21136:SF5 (PANTHER);Longin domain	PTHR21136 (PANTHER);Longin domain			
comp150	1	IPR4932;Retrieval					

39_c1_seq1		of early ER protein Rer1						
comp15162_c0_seq1	7	IPR17984;Chromo domain subgroup	IPR8251;Chromo shadow domain	IPR953;Chromo/chromo shadow domain	IPR2378;Chromo domain	G3DSA:2.4.5.4 (GENE3D);Chromo domain	PTHR22812 (PANTHER);Chromo domain	IPR23779;Chromo domain, conserved site
comp15169_c0_seq1	3	IPR3495;CobW/HypB/UreG domain	PTHR13748:SF4 (PANTHER);CobW/HypB/UreG domain	PTHR13748 (PANTHER);CobW/HypB/UreG domain				
comp15204_c0_seq1	1	PTHR1933:SF163 (PANTHER);Zinc finger, CCHC-type						
comp15251_c1_seq1	5	IPR8594;Scavenger mRNA decapping enzyme DcpS/DCS2	IPR11146;HIT-like domain	PF11969 (PFAM);HIT-like domain	PTHR12978:SF (PANTHER);Scavenger mRNA decapping enzyme DcpS/DCS2	IPR11145;Scavenger mRNA decapping enzyme, N-terminal		
comp15287_c0_seq1	3	IPR8628;Golgi phosphoprotein 3	G3DSA:1.1.363.1 (GENE3D);Golgi phosphoprotein 3	PTHR1274:SF2 (PANTHER);Golgi phosphoprotein 3				
comp15313_c0_seq1	1	IPR22197;Protein of unknown function DUF3722						
comp15329_c0_seq1	3	IPR534;Semialdehyde dehydrogenase, NAD-binding	PTHR1366:SF89 (PANTHER);NAD(P)-binding domain	PTHR1366 (PANTHER);NAD(P)-binding domain				
comp15406_c0_seq1	2	IPR222;DapA-like	PTHR12128:SF14 (PANTHER);Aldolase-type TIM barrel					
comp15421_c0_seq1	1	IPR29526;PiggyBac transposable element-derived protein						
comp15512_c0_seq1	1	PTHR2473:SF32 (PANTHER);Small GTPase superfamily						
comp15546_c0_seq3	3	IPR114;Ribosomal protein L16	PTHR1222:SF13 (PANTHER);Ribosomal protein L16	IPR2798;Ribosomal protein L16, conserved site				
comp15624_c0_seq1	2	IPR17998;Chaperone tailless complex polypeptide 1 (TCP-1)	IPR2194;Chaperonin TCP-1, conserved site					
comp15691_c1_seq1	1	G3DSA:3.4.5.2 (GENE3D);null						

comp15875_c0_seq1	7	IPR15815;Hydroxy monocarboxylic acid anion dehydrogenase, HIBADH-type	IPR6115;6-phosphogluconate dehydrogenase, NADP-binding	IPR29154;NADP-dependent 3-hydroxyisobutyrate dehydrogenase, NAD-binding domain	IPR13328; Dehydrogenase, multihelical	IPR11548;3-hydroxyisobutyrate dehydrogenase	PTHR22981:SF7 (PANTHER);3-hydroxyisobutyrate dehydrogenase	PTHR22981 (PANTHER);3-hydroxyisobutyrate dehydrogenase
comp15882_c0_seq1	2	IPR175;NADH:quinone oxidoreductase/Mrp antiporter, membrane subunit	PTHR22773:SF41 (PANTHER); NADH:quinone oxidoreductase/Mrp antiporter, membrane subunit					
comp15886_c0_seq1	2	IPR868;Isochorismatase-like	PTHR14119 (PANTHER);Isochorismatase-like					
comp15990_c0_seq1	3	G3DSA:3.3.45.7 (GENE3D);null	IPR7233;Sybindin-like protein	PTHR23249:SF16 (PANTHER);Sybindin-like protein				
comp15994_c0_seq1	3	IPR1279;Beta-lactamase-like	IPR17782;Hydroxyacylglutathione hydrolase	PTHR11935 (PANTHER);Beta-lactamase-like				
comp16143_c0_seq1	3	IPR754;H/ACA ribonucleoprotein complex, subunit Gar1/Naf1	G3DSA:2.4.1.23 (GENE3D);H/ACA ribonucleoprotein complex, subunit Gar1/Naf1	IPR21154;H/ACA ribonucleoprotein complex, subunit Gar1				
comp16214_c1_seq1	1	IPR911;Mannose-6-phosphate receptor binding domain						
comp16358_c0_seq1	2	IPR8238;Chorismate mutase, AroQ class, eukaryotic type	IPR2822;Chorismate mutase, type II					
comp16384_c0_seq1	5	IPR1951;Histone H4	IPR4823;TATA box binding protein associated factor (TAF)	IPR7125;Histone core	PTHR1484 (PANTHER);Histone-fold	IPR1989;Histone H4, conserved site		
comp16414_c0_seq1	3	IPR1592;Cellobiose dehydrogenase, cytochrome domain	IPR518;DOMON domain	SSF49344 (SUPERFAMILY); DOMON domain				
comp16610_c0_seq1	2	IPR4841;Amino acid permease/ SLC12A domain	PTHR11785:SF247 (PANTHER); Amino acid permease/ SLC12A domain					
comp16639_c0_seq1	1	IPR484;Amino acid permease, conserved site						
comp16655_c0_seq1	3	IPR54;RNA recognition motif domain	PTHR23147 (PANTHER); RNA recognition motif domain	SSF54928 (SUPERFAMILY); RNA recognition motif domain				

comp16725_c0_seq1	1	PTHR18934:SF19 (PANTHER);P-loop containing nucleoside triphosphate hydrolase					
comp16814_c0_seq1	2	IPR7889;DNA binding HTH domain, Psq-type	IPR957;Homeodomain-like				
comp16825_c0_seq1	6	IPR245;V-ATPase proteolipid subunit	IPR2379;V-ATPase proteolipid subunit C-like domain	IPR11555;V-ATPase proteolipid subunit C, eukaryotic	G3DSA:1.2.12.61 (GENE3D);V-ATPase proteolipid subunit C, eukaryotic	PTHR1263 (PANTHER);V-ATPase proteolipid subunit C, eukaryotic	PTHR1263:SF8 (PANTHER);V-ATPase proteolipid subunit C, eukaryotic
comp16847_c0_seq1	2	IPR3661;Signal transduction histidine kinase EnvZ-like, dimerisation/phosphoacceptor domain	PTHR24423:SF416 (PANTHER);Signal transduction histidine kinase EnvZ-like, dimerisation/phosphoacceptor domain				
comp16870_c0_seq1	1	PTHR11735:SF9 (PANTHER);Gcp-like domain					
comp16884_c0_seq1	4	IPR1164;Arf GTPase activating protein	PTHR2318:SF29 (PANTHER);Arf GTPase activating protein	PTHR2318 (PANTHER);Arf GTPase activating protein	SSF57863 (SUPERFAMILY);Arf GTPase activating protein		
comp16986_c0_seq1	5	IPR85;Adenylate kinase/UMP-CMP kinase	IPR367;HD/PDEase domain	IPR6674;HD domain	PF46 (PFAM);HD domain	SSF1964 (SUPERFAMILY);P-loop containing nucleoside triphosphate hydrolase	
comp17026_c0_seq1	1	IPR1898;Ribosomal S24e conserved site					
comp17140_c2_seq1	1	PTHR24353 (PANTHER);Protein kinase domain					
comp17177_c1_seq1	2	PTHR24361:SF285 (PANTHER);Protein kinase domain	PTHR24361 (PANTHER);Protein kinase domain				
comp17213_c1_seq1	1	G3DSA:3.4.367.2 (GENE3D);null					
comp17254_c0_seq1	1	IPR7248;Mpv17/PMP22					
comp17296_c0_seq1	1	PTHR11695:SF385 (PANTHER);Alcohol dehydrogenase superfamily, zinc-type					

comp17320_c0_seq1	4	IPR7641;RNA polymerase Rpb2, domain 7	IPR15712;DNA-directed RNA polymerase, subunit 2	PTHR2856:SF8 (PANTHER);DNA-directed RNA polymerase, subunit 2	SSF64484 (SUPERFAMILY);DNA-directed RNA polymerase, subunit 2
comp17373_c1_seq1	1	IPR24381;Protein of unknown function DUF2561			
comp17436_c0_seq3	3	IPR1377;Ribosomal protein S6e	PTHR1152:SF6 (PANTHER);Ribosomal protein S6e	IPR18282;Ribosomal protein S6e, conserved site	
comp17447_c0_seq1	2	IPR19328;GPI-GlcNAc transferase complex, PIG-H component, conserved domain	PTHR15231 (PANTHER);GPI-GlcNAc transferase complex, PIG-H component, conserved domain		
comp17477_c0_seq1	2	IPR15655;Protein phosphatase 2C	PTHR13832:SF1 (PANTHER);Protein phosphatase 2C		
comp17485_c1_seq1	3	IPR19529;SNARE-complex protein Syntaxin-18 N-terminal	PTHR15959 (PANTHER);SNARE-complex protein Syntaxin-18 N-terminal	PTHR15959:SF (PANTHER);SNARE-complex protein Syntaxin-18 N-terminal	
comp17513_c0_seq1	2	IPR5349;Uncharacterised protein family UPF136, Transmembrane	PTHR12668:SF5 (PANTHER);Uncharacterised protein family UPF136, Transmembrane		
comp17514_c0_seq1	4	IPR11545;DEAD/DEAH box helicase domain	PTHR1371:SF7 (PANTHER);P-loop containing nucleoside triphosphate hydrolase	PTHR1371 (PANTHER);P-loop containing nucleoside triphosphate hydrolase	IPR141;Helicase superfamily 1/2, ATP-binding domain
comp17571_c1_seq1	1	IPR2534;Protein of unknown function DUF4246			
comp17607_c0_seq1	4	IPR1789;Signal transduction response regulator, receiver domain	G3DSA:3.4.5.23 (GENE3D);Signal transduction response regulator, receiver domain	PTHR24423:SF442 (PANTHER);Signal transduction response regulator, receiver domain	IPR116;CheY-like superfamily
comp17622_c0_seq1	2	IPR1227;Acyl transferase domain	PTHR1982 (PANTHER);Acyl transferase domain		

comp17656_c1_seq2	4	IPR18826;WW-domain-binding protein	PTHR3166 (PANTHER); WW-domain-binding protein	PTHR3166:SF1 (PANTHER);WW-domain-binding protein	SSF5729 (SUPERFAMILY);WW-domain-binding protein			
comp17665_c1_seq1	1	IPR1138;Zn(2)-C6 fungal-type DNA-binding domain						
comp17679_c2_seq1	1	PTHR1176:SF35 (PANTHER);NR AMP family						
comp17723_c1_seq1	1	IPR22495;Serine/threonine-protein kinase Bud32						
comp17820_c0_seq1	3	IPR1114;Adenylosuccinate synthetase	G3DSA:1.1.3.1 (GENE3D);Adenylosuccinate synthetase	G3DSA:3.4.44.1 (GENE3D);Adenylosuccinate synthetase				
comp17887_c0_seq1	2	PTHR2458:SF17 (PANTHER);null	PTHR2458 (PANTHER); null					
comp17890_c0_seq1	3	IPR2922;Thiazole biosynthetic enzyme Thi4 family	PF1946 (PFAM);Thiazole biosynthetic enzyme Thi4 family	IPR27495;Thiamine thiazole synthase				
comp17965_c1_seq1	2	PTHR31845:SF4 (PANTHER);null	PTHR31845 (PANTHER); null					
comp17973_c0_seq1	7	IPR2831;Glyceraldehyde/Erythrose phosphate dehydrogenase family	IPR2828;Glyceraldehyde 3-phosphate dehydrogenase, NAD(P) binding domain	G3DSA:3.3.36.1 (GENE3D);Glyceraldehyde 3-phosphate dehydrogenase, NAD(P) binding domain	IPR164;NAD(P)-binding domain	IPR2829;Glyceraldehyde 3-phosphate dehydrogenase, catalytic domain	IPR6424;Glyceraldehyde-3-phosphate dehydrogenase, type I	IPR283;Glyceraldehyde 3-phosphate dehydrogenase, active site
comp18016_c0_seq1	1	IPR11759;Cytochrome C oxidase subunit II, transmembrane domain						
comp18045_c0_seq1	4	IPR18;Thioester reductase-like domain	IPR1312;Male sterility, NAD-binding	PTHR2495 (PANTHER);Male sterility, NAD-binding	PTHR2495:SF141 (PANTHER);Male sterility, NAD-binding			
comp18045_c1_seq1	3	IPR873;AMP-dependent synthetase/ligase	G3DSA:2.3.38.1 (GENE3D);AMP-dependent synthetase/ligase	SSF5681 (SUPERFAMILY); AMP-dependent synthetase/ligase				
comp18102_c0_seq1	1	PTHR1221 (PANTHER);null						
comp18195_c0_seq1	2	IPR1313;Reverse transcriptase, RNA-dependent DNA polymerase	PTHR11439:SF127 (PANTHER); Reverse transcriptase, RNA-dependent DNA					

			polymerase					
comp18256_c1_seq1	2	IPR26825;Vacuole morphology and inheritance protein 14	PTHR1623:SF (PANTHER); Vacuole morphology and inheritance protein 14					
comp18269_c2_seq1	5	IPR13567;EF hand associated, type-2	IPR13566;EF hand associated, type-1	IPR13684;Mitochondrial Rho-like	PTHR2472:SF73 (PANTHER);Mitochondrial Rho-like	IPR286;MIRO		
comp18360_c1_seq1	3	IPR1764;Glycoside hydrolase, family 3, N-terminal	PTHR348:SF4 (PANTHER); Glycoside hydrolase, family 3, N-terminal	PTHR348 (PANTHER);Glycoside hydrolase, family 3, N-terminal				
comp18414_c1_seq1	2	PTHR3244:SF (PANTHER);Calcineurin-like phosphoesterase domain, apaH type	PTHR3244 (PANTHER); Calcineurin-like phosphoesterase domain, apaH type					
comp18451_c0_seq1	2	PTHR1189 (PANTHER);P-loop containing nucleoside triphosphate hydrolase	PTHR1189:SF4 (PANTHER); P-loop containing nucleoside triphosphate hydrolase					
comp18461_c0_seq1	7	IPR2472;G-protein beta WD-4 repeat	IPR168;WD4 repeat	IPR15943;WD4/YVTN repeat-like-containing domain	PTHR22844 (PANTHER);WD4/YVTN repeat-like-containing domain	PTHR22844:SF12 (PANTHER);WD4/YVTN repeat-like-containing domain	IPR19775;WD4 repeat, conserved site	IPR772;Ricin B lectin domain
comp18463_c0_seq1	1	IPR3615;HNH nuclease						
comp18475_c0_seq1	2	PF3142 (PFAM);null	PTHR22914:SF15 (PANTHER); Chitin synthase					
comp18477_c2_seq1	1	PTHR24343:SF127 (PANTHER);Protein kinase domain						
comp18489_c0_seq1	1	PTHR22836 (PANTHER);WD4 repeat						
comp18491_c0_seq1	3	IPR2283;Ribosomal protein L5 domain	IPR2132;Ribosomal protein L5	IPR2929;Ribosomal protein L5, conserved site				
comp18493_c0_seq1	3	IPR1594;Zinc finger, DHHC-type, palmitoyltransferase	PTHR22883 (PANTHER); Zinc finger, DHHC-type, palmitoyltransferase	PTHR22883:SF43 (PANTHER);Zinc finger, DHHC-type, palmitoyltransferase				

comp18516_c0_seq1	2	PTHR1417 (PANTHER);WD4/YVTN repeat-like-containing domain	PTHR1417:SF16 (PANTHER); WD4/YVTN repeat-like-containing domain					
comp18530_c0_seq1	1	IPR1912;RNA cap guanine-N2 methyltransferase						
comp18530_c0_seq2	2	IPR4443;YjeF N-terminal domain	PTHR13232 (PANTHER); YjeF N-terminal domain					
comp18540_c0_seq1	2	IPR1457;NADH:ubiquinone/plastoquinone oxidoreductase, chain 6	PTHR11435 (PANTHER); NADH:ubiquinone/plastoquinone oxidoreductase, chain 6					
comp18576_c0_seq1	1	PTHR1297 (PANTHER);Armadillo-like helical						
comp18580_c0_seq1	1	PTHR1947 (PANTHER);null						
comp18581_c0_seq1	3	PF13393 (PFAM);null	IPR4516;Histidine-tRNA ligase/ATP phosphoribosyltransferase regulatory subunit	PTHR11476:SF1 (PANTHER);Histidine-tRNA ligase/ATP phosphoribosyltransferase regulatory subunit				
comp18584_c0_seq1	7	G3DSA:2.4.3.1 (GENE3D);null	IPR416;Translation elongation factor EFTu/EF1A, C-terminal	IPR4161;Translation elongation factor EFTu/EF1A, domain 2	PTHR23115 (PANTHER);Translation elongation factor EFTu/EF1A, domain 2	PTHR23115:SF14 (PANTHER);Translation elongation factor EFTu/EF1A, domain 2	IPR91;Translation elongation factor EF1A/initiation factor IF2gamma, C-terminal	IPR9;Translation protein, beta-barrel domain
comp18596_c1_seq1	4	IPR11989;Armadillo-like helical	IPR13598;Exportin-1/Importin-beta-like	PTHR15952 (PANTHER);Exportin-1/Importin-beta-like	IPR1624;Armadillo-type fold			
comp18616_c1_seq1	5	IPR1684;Haem oxygenase-like, multi-helical	IPR13749;Pyridoxamine kinase/Phosphomethylpyrimidine kinase	IPR435;Thiaminase-2/PQQC	PTHR2858:SF17 (PANTHER);Ribokinase-like	PTHR2858 (PANTHER);Ribokinase-like		
comp18715_c1_seq1	2	PTHR31123 (PANTHER);null	IPR3186;Protein AlcS					
comp18782_c1_seq1	1	PTHR22847:SF339 (PANTHER);WD4/YVTN repeat-like-containing domain						
comp18783_c1_seq1	3	IPR33;SNF2-related	PTHR1799:SF213 (PANTHER); SNF2-related	PTHR1799 (PANTHER);SNF2-related				

comp18799_c0_seq1	7	IPR19985;Ribosomal protein L23	IPR1325;Ribosomal protein L25/L23	IPR12677;Nucleotide-binding alpha-beta plait domain	IPR5633;Ribosomal protein L23/L25, N-terminal	PTHR1162 (PANTHER);Ribosomal protein L23/L25, N-terminal	IPR114; Ribosomal protein L23/L25, conserved site	IPR12678;Ribosomal protein L23/L15e core domain
comp18801_c1_seq1	3	IPR268;Alpha crystallin/Hsp2 domain	PTHR11527:SF18 (PANTHER); Alpha crystallin/Hsp2 domain	PTHR11527 (PANTHER);Alpha crystallin/Hsp2 domain				
comp18815_c1_seq1	1	PTHR244:SF34 (PANTHER);null						
comp18822_c0_seq1	4	IPR23626;Ribosomal protein L39e domain	IPR77;Ribosomal protein L39e	IPR283;Ribosomal protein L39e, conserved site	PD7914 (PRODOM);Ribosomal protein L39e, conserved site			
comp18835_c0_seq1	3	IPR592;Ribosomal protein S27e	IPR2347;Ribosomal protein S27e, zinc-binding domain	IPR11332;Zinc-binding ribosomal protein				
comp18837_c0_seq1	2	IPR1365;Adenosine/AMP deaminase domain	IPR6329;AMP deaminase					
comp18854_c1_seq1	4	IPR1171;Major facilitator superfamily	PTHR244:SF55 (PANTHER); Major facilitator superfamily	PTHR244 (PANTHER);Major facilitator superfamily	IPR5829;Sugar transporter, conserved site			
comp18920_c0_seq1	5	IPR655;Exonuclease	IPR28881;PAB-dependent poly(A)-specific ribonuclease subunit PAN2	IPR1352;Exonuclease, RNase T/DNA polymerase III	PTHR15728:SF (PANTHER);Ribonuclease H-like domain	PTHR15728 (PANTHER);Ribonuclease H-like domain		
comp18948_c0_seq1	3	IPR1898;Sodium/sulphate symporter	PTHR1283 (PANTHER); Sodium/sulphate symporter	PTHR1283:SF86 (PANTHER);Sodium/sulphate symporter				
comp18967_c0_seq1	5	IPR2523;Mg2+ transporter protein, CorA-like/Zinc transport protein ZntB	G3DSA:1.2.58.34 (GENE3D);Mg2+ transporter protein, CorA-like/Zinc transport protein ZntB	PTHR21535:SF35 (PANTHER);Mg2+ transporter protein, CorA-like/Zinc transport protein ZntB	SSF143865 (SUPERFAMILY);Mg2+ transporter protein, CorA-like/Zinc transport protein ZntB	SSF14483 (SUPERFAMILY);Mg2+ transporter protein, CorA-like/Zinc transport protein ZntB		
comp18996_c1_seq1	1	PTHR2463:SF339 (PANTHER);General substrate transporter						
comp19006_c0_seq1	5	IPR13787;S1/CaBP-9k-type, calcium binding, subdomain	PTHR11639 (PANTHER); EF-hand domain pair	PTHR11639:SF52 (PANTHER);EF-hand domain pair	IPR1751;S1/Calbindin-D9k, conserved site	IPR18247;EF-Hand 1, calcium-binding site		
comp19022_c0_se	2	IPR1621;Plexin-like fold	IPR2165;Plexin					

q1							
comp19024_c1_seq1	1	PTHR19375:SF94 (PANTHER);null					
comp19049_c0_seq1	6	IPR5824;KOW	IPR1141;Ribosomal protein L27e	IPR14722;Ribosomal protein L2 domain 2	PTHR1497:SF (PANTHER);Ribosomal protein L27e	IPR18262;Ribosomal protein L27e, conserved site	IPR8991;Translation protein SH3-like domain
comp19054_c2_seq1	5	IPR26541;MRG domain	IPR25995;RNA binding activity-knot of a chromodomain	G3DSA:2.3.3.27 (GENE3D);RNA binding activity-knot of a chromodomain	PTHR188:SF15 (PANTHER);RNA binding activity-knot of a chromodomain	IPR8676;MRG	
comp19080_c0_seq1	2	IPR554;Ribosomal protein S7e	PTHR11278:SF (PANTHER);Ribosomal protein S7e				
comp19089_c1_seq1	2	PTHR31778:SF2 (PANTHER);null	PTHR31778 (PANTHER);null				
comp19090_c0_seq1	1	PTHR244:SF73 (PANTHER);null					
comp19092_c0_seq1	3	IPR1588;Zinc finger, DNA-directed DNA polymerase, family B, alpha	PTHR1322 (PANTHER);Zinc finger, DNA-directed DNA polymerase, family B, alpha	IPR2972;DNA polymerase alpha catalytic subunit			
comp19131_c2_seq1	4	IPR8335;Eukaryotic molybdopterin oxidoreductase	IPR572;Oxidoreductase, molybdopterin-binding domain	PTHR19372 (PANTHER);Oxidoreductase, molybdopterin-binding domain	PTHR19372:SF2 (PANTHER);Oxidoreductase, molybdopterin-binding domain		
comp19134_c0_seq1	1	IPR149;Conserved oligomeric Golgi complex subunit 6					
comp19143_c0_seq1	1	IPR24222;CST complex subunit Ten1, fungal					
comp19161_c0_seq1	1	IPR1665;Eukaryotic translation initiation factor 3 subunit E					
comp19162_c0_seq1	1	IPR8728;Elongator complex protein 4					
comp19164_c0_seq1	2	PTHR11134:SF13 (PANTHER);Armadillo-like helical	IPR26739;AP complex subunit beta				
comp19169_c0_seq1	2	IPR662;Prolyl 4-hydroxylase, alpha subunit	PTHR1869:SF5 (PANTHER);Oxoglutarate/iron-dependent dioxygenase				

comp19178_c0_seq1	3	IPR294;Ribosomal L28e/Mak16	PTHR1544:SF (PANTHER); Ribosomal L28e/Mak16	IPR2672;Ribosomal protein L28e				
comp19207_c0_seq1	5	IPR438;Ribosomal protein L7Ae/L3e/S12e/Gadd45	IPR2964;5S ribosomal protein L3e-like	IPR231;Ribosomal protein L3e	PTHR11449:SF1 (PANTHER);Ribosomal protein L3e	IPR22991;Ribosomal protein L3e, conserved site		
comp19215_c0_seq1	2	PTHR31438 (PANTHER);Acyl-CoA N-acyltransferase	PTHR31438:SF4 (PANTHER); Acyl-CoA N-acyltransferase					
comp19221_c0_seq1	1	IPR28482;Protein S1-A11						
comp19222_c0_seq1	1	IPR19384;Retinoic acid induced 16-like protein						
comp19289_c0_seq1	1	PTHR11946:SF51 (PANTHER);Rossmann-like alpha/beta/alpha sandwich fold						
comp19290_c0_seq1	6	IPR1892;Ribosomal protein S13	G3DSA:1.1.8.5 (GENE3D);Ribosomal protein S13	IPR27437;3s ribosomal protein S13, C-terminal	PTHR1871 (PANTHER);3s ribosomal protein S13, C-terminal	IPR18269;Ribosomal protein S13, conserved site	IPR1979;Ribosomal protein S13-like, H2TH	
comp19296_c0_seq1	2	IPR4827;Basic-leucine zipper domain	SSF57959 (SUPERFAMILY);Basic-leucine zipper domain					
comp19301_c0_seq1	7	IPR12347;Ferritin-related	IPR8331;Ferritin/DPS protein domain	PTHR11431:SF37 (PANTHER);Ferritin/DPS protein domain	IPR1519;Ferritin	IPR1434;Ferritin, conserved site	IPR94;Ferritin-like diiron domain	IPR978;Ferritin-like superfamily
comp19303_c0_seq1	5	IPR1247;Exoribonuclease, phosphorolytic domain 1	IPR2748;PNPase/RNase PH domain	IPR15847;Exoribonuclease, phosphorolytic domain 2	PTHR1197:SF14 (PANTHER);Exoribonuclease, phosphorolytic domain 2	PTHR1197 (PANTHER);Exoribonuclease, phosphorolytic domain 2		
comp19304_c1_seq1	2	IPR16833;Putative sodium bile acid cotransporter	PTHR1864:SF5 (PANTHER); Putative sodium bile acid cotransporter					
comp19336_c0_seq1	3	PTHR362:SF2 (PANTHER);Glycoside hydrolase, family 3, N-terminal	IPR26892;Glycoside hydrolase family 3	IPR198;Glycoside hydrolase, family 3, active site				
comp19344_c0_seq1	2	PTHR1136 (PANTHER);null	PTHR1136:SF1 (PANTHER); null					
comp19360_c1_se	1	G3DSA:1.2.128.5 (GENE3D);null						

q1						
comp19367_c1_seq1	1	IPR1111;Zinc finger, FYVE/PHD-type				
comp19373_c0_seq1	1	IPR5756;Ribosomal protein L26/L24P, eukaryotic/archaeal				
comp19392_c0_seq2	5	G3DSA:3.3.3.11 (GENE3D);null	IPR3382;SAM-dependent methyltransferase TRM5/TYW2-type	PTHR23245:SF25 (PANTHER);SAM-dependent methyltransferase TRM5/TYW2-type	PTHR23245 (PANTHER);SAM-dependent methyltransferase TRM5/TYW2-type	IPR25792;tRNA (guanine(37)-N(1))-methyltransferase, eukaryotic
comp19408_c0_seq1	4	PF428 (PFAM);null	PTHR21141 (PANTHER);null	PTHR21141:SF3 (PANTHER);null	IPR27534;Ribosomal protein L12 family	
comp19433_c0_seq1	3	IPR11613;Glycoside hydrolase family 15	PTHR31616 (PANTHER);Glycoside hydrolase family 15	PTHR31616:SF (PANTHER);Glycoside hydrolase family 15		
comp19455_c0_seq1	2	IPR25755;6S ribosomal protein L4, C-terminal domain	PTHR19431 (PANTHER);6S ribosomal protein L4, C-terminal domain			
comp19462_c0_seq1	4	IPR13154;Alcohol dehydrogenase GroES-like	IPR1132;GroES (chaperonin 1)-like	IPR285;Alcohol dehydrogenase superfamily, zinc-type	IPR2328;Alcohol dehydrogenase, zinc-type, conserved site	
comp19502_c1_seq1	1	PTHR11751:SF31 (PANTHER);Pyridoxal phosphate-dependent transferase, major region, subdomain 1				
comp19503_c0_seq1	3	G3DSA:1.2.5.17 (GENE3D);null	IPR27695;Keratin, type I cytoskeletal 18	IPR1839;Intermediate filament protein, conserved site		
comp19507_c1_seq1	2	IPR1326;Exocyst complex component Sec6	PTHR21292:SF1 (PANTHER);Exocyst complex component Sec6			
comp19512_c5_seq1	3	IPR2534;ALIX V-shaped domain	PTHR233:SF21 (PANTHER);ALIX V-shaped domain	PTHR233 (PANTHER);ALIX V-shaped domain		
comp19541_c0_seq1	5	IPR23298;P-type ATPase, transmembrane domain	IPR668;Cation-transporting P-type ATPase, C-terminal	PTHR2493 (PANTHER);Cation-transporting P-type ATPase, C-terminal	PTHR2493:SF222 (PANTHER);Cation-transporting	SSF81665 (SUPERFAMILY);Cation-transporting

						P-type ATPase, C-terminal	P-type ATPase, C-terminal
comp19543_c0_seq1	2		IPR1931;Ribosomal protein S21e	IPR18279;Ribosomal protein S21e, conserved site			
comp19568_c1_seq1	3		IPR56;Histidine phosphatase superfamily, clade-2	PTHR2963:SF15 (PANTHER);Histidine phosphatase superfamily, clade-2	PTHR2963 (PANTHER);Histidine phosphatase superfamily, clade-2		
comp19570_c0_seq1	3		IPR178;Ribosomal protein L35A	PTHR192:SF (PANTHER);Ribosomal protein L35A	IPR18266;Ribosomal protein L35Ae, conserved site		
comp19572_c1_seq1	1		IPR1238;DNA ligase, ATP-dependent, N-terminal				
comp19593_c0_seq1	4		IPR2529;Fumarylacetoacetase, C-terminal	IPR11234;Fumarylacetoacetase, C-terminal-related	PTHR1182:SF76 (PANTHER);Fumarylacetoacetase, C-terminal-related	PTHR1182 (PANTHER);Fumarylacetoacetase, C-terminal-related	
comp19616_c0_seq1	2		IPR1971;Ribosomal protein S11	SSF53137 (SUPERFAMILY);Ribosomal protein S11			
comp19617_c1_seq1	2		PTHR139:SF (PANTHER);null	PTHR139 (PANTHER);null			
comp19621_c0_seq1	3		IPR23571;Ribosomal protein L14 domain	IPR218;Ribosomal protein L14b/L23e	IPR19972;Ribosomal protein L14 conserved site		
comp19631_c0_seq1	4		IPR266;Ribosomal protein S17	IPR28333;Ribosomal protein S17, archaeal/eukaryotic	IPR1234;Nucleic acid-binding, OB-fold	IPR19979;Ribosomal protein S17, conserved site	
comp19664_c1_seq1	2		PTHR22597 (PANTHER);null	PTHR22597:SF1 (PANTHER);null			
comp19674_c1_seq1	2		PTHR166:SF39 (PANTHER);P-loop containing nucleoside triphosphate hydrolase	IPR394;Fructose-2,6-bisphosphatase			
comp19677_c0_seq1	5		IPR74;Apolipoprotein A/E	G3DSA:1.2.12.2 (GENE3D);Apolipoprotein A/E	PTHR18976:SF2 (PANTHER);Apolipoprotein A/E	PTHR18976 (PANTHER);Apolipoprotein A/E	SSF47162 (SUPERFAMILY);Apolipoprotein A/E
comp19709_c0_seq1	5		IPR1815;Translationally controlled tumour protein	IPR11323;Mss4/translationally controlled tumour-associated TCTP	PTHR11991:SF3 (PANTHER);Translationally controlled tumour protein	IPR1813;Translationally controlled tumour protein, conserved site	IPR1157;Mss4-like
comp19724_c1_seq1	5		G3DSA:3.4.63.1 (GENE3D);null	IPR7484;Peptidase M28	PTHR12147:SF6 (PANTHER);Peptidase M28	PTHR12147 (PANTHE	SSF53187 (SUPERFAMILY);Pepti

						R);Peptidase M28	dase M28	
comp19749_c0_seq1	2		IPR147;Ribosomal protein S8e	IPR2239;Ribosomal protein S8e/ribosomal biogenesis NSA2				
comp19757_c0_seq1	2		IPR13926;CGI121/TPRKB	SSF14387 (SUPERFAMILY);CGI121/TPRKB				
comp19759_c1_seq1	2		IPR273;3'5'-cyclic nucleotide phosphodiesterase, catalytic domain	PTHR11347 (PANTHER); 3'5'-cyclic nucleotide phosphodiesterase, catalytic domain				
comp19762_c0_seq1	1		IPR851;Ribosomal protein S5					
comp19809_c0_seq1	1		PTHR21141:SF3 (PANTHER);null					
comp19819_c0_seq1	2		PF1457 (PFAM);Zinc finger, RING/FYVE/PHD-type	PTHR1263 (PANTHER); Zinc finger, RING/FYVE/PHD-type				
comp19825_c0_seq1	1		IPR1958;Pre-mRNA-processing-splicing factor 8, U6-snRNA-binding					
comp19826_c0_seq1	3		IPR2189;Translation elongation factor, IF5A C-terminal	PTHR11673:SF7 (PANTHER); Nucleic acid-binding, OB-fold	IPR1884;Translation elongation factor IF5A			
comp19841_c0_seq1	5		IPR2948;Heat shock protein 7kD, C-terminal domain	IPR13126;Heat shock protein 7 family	PTHR19375:SF15 (PANTHER);Heat shock protein 7 family	PTHR19375 (PANTHER);Heat shock protein 7 family	IPR2947;Heat shock protein 7kD, peptide-binding domain	
comp19842_c0_seq1	2		IPR552;Ribosomal protein L44e	PTHR1369:SF6 (PANTHER); Ribosomal protein L44e				
comp19866_c0_seq1	6		IPR5454;Profilin1/2/3, vertebrate	IPR5455;Profilin	G3DSA:3.3.45.3 (GENE3D);Profilin	PTHR13936 (PANTHER);Profilin	IPR29892;Profilin-1	SignalP-TM (SIGNALP_GRAM_POSITIVE);Profilin-1
comp19869_c0_seq1	5		IPR1965;Zinc finger, PHD-type	PF13771 (PFAM);Zinc finger, PHD-type	PTHR13793 (PANTHER);Zinc finger, PHD-type	PTHR13793:SF82 (PANTHER);Zinc finger, PHD-type	IPR19787;Zinc finger, PHD-finger	
comp19876_c0_se	3		IPR24;Ribosomal protein L6, alpha-	IPR72;Ribosomal protein L6	IPR2359;Ribosomal protein L6,			

q1		beta domain		conserved site-2				
comp19877_c0_seq1	3	IPR1662;Translation elongation factor EF1B, gamma chain, conserved	PTHR1126:SF161 (PANTHER); Translation elongation factor EF1B, gamma chain, conserved	PTHR1126 (PANTHER);Translation elongation factor EF1B, gamma chain, conserved				
comp19881_c0_seq1	7	IPR1713;Proteinase inhibitor I25A, stefin A	IPR1;Proteinase inhibitor I25, cystatin	G3DSA:3.1.45.1 (GENE3D);Proteinase inhibitor I25, cystatin	PTHR11414:SF19 (PANTHER);Proteinase inhibitor I25, cystatin	PTHR11414 (PANTHER);Proteinase inhibitor I25, cystatin	IPR1873;Proteinase inhibitor I25, cystatin, conserved site	SSF5443 (SUPERFAMILY); Proteinase inhibitor I25, cystatin, conserved site
comp19882_c0_seq1	3	IPR5485;Ribosomal protein L5 eukaryotic/L18 archaeal	G3DSA:3.3.42.1 (GENE3D);Ribosomal protein L5 eukaryotic/L18 archaeal	IPR2567;Ribosomal protein L5 eukaryotic/L18 archaeal, C-terminal				
comp19887_c0_seq1	2	IPR9;Protein phosphatase 2A, regulatory subunit PR55	IPR1867;Protein phosphatase 2A, regulatory subunit PR55, conserved site					
comp19974_c1_seq1	2	PTHR1617 (PANTHER);null	PTHR1617:SF98 (PANTHER); null					
comp19978_c0_seq1	2	G3DSA:3.4.5.98 (GENE3D);AMP-dependent synthetase/ligase	IPR2845;AMP-binding, conserved site					
comp19992_c2_seq1	3	IPR7282;NOT2/NOT3/NOT5	PTHR23326 (PANTHER); NOT2/NOT3/NOT5	PTHR23326:SF1 (PANTHER);NOT2/NOT3/NOT5				
comp20000_c0_seq1	5	IPR1197;Ribosomal protein L1e	IPR1618;Ribosomal protein L1e/L16	PTHR11726:SF9 (PANTHER);Ribosomal protein L1e/L16	PTHR11726 (PANTHER);Ribosomal protein L1e/L16	IPR18255;Ribosomal protein L1e, conserved site		
comp20075_c0_seq1	4	IPR1987;Glutathione S-transferase, C-terminal-like	IPR446;Glutathione S-transferase, C-terminal	PTHR11571 (PANTHER);Glutathione S-transferase, C-terminal	PTHR11571:SF96 (PANTHER);Glutathione S-transferase, C-terminal			
comp20084_c0_seq1	2	IPR13177;Domain of unknown function DUF1713, mitochondria	PTHR3235 (PANTHER); Domain of unknown function DUF1713, mitochondria					
comp20099_c1_seq1	2	IPR366;HAMP linker domain	SSF5814 (SUPERFAMILY);HAMP linker domain					
comp20102_c0_se	4	IPR1682;Ribosomal protein L3,	IPR12988;Ribosomal	PTHR11524 (PANTHER);Ribos	IPR1838;Ribosomal			

q1		ferredoxin-like fold domain	protein L3, N-terminal	omal protein L3, ferredoxin-like fold domain	protein L3, conserved site
comp20114_c0_seq1	2	PTHR1165 (PANTHER);null	PTHR1165:SF35 (PANTHER);null		
comp20129_c0_seq1	3	IPR2955;Nucleophile aminohydrolases, N-terminal	PTHR11599 (PANTHER);Nucleophile aminohydrolases, N-terminal	PTHR11599:SF5 (PANTHER);Nucleophile aminohydrolases, N-terminal	
comp20131_c0_seq1	1	IPR632;Ribosomal protein S12/S23			
comp20192_c0_seq1	1	PTHR23115:SF19 (PANTHER);Elongation factor, GTP-binding domain			
comp20204_c0_seq1	4	IPR5822;Ribosomal protein L13	IPR23564;Ribosomal protein L13 domain	IPR5755;Ribosomal protein L13, eukaryotic/archaeal	IPR23563;Ribosomal protein L13, conserved site
comp20208_c3_seq1	1	PTHR24188 (PANTHER);Ankyrin repeat-containing domain			
comp20209_c0_seq1	4	IPR1515;Ribosomal protein L32e	PTHR23413:SF3 (PANTHER);Ribosomal protein L32e	PTHR23413 (PANTHER);Ribosomal protein L32e	IPR18263;Ribosomal protein L32e, conserved site
comp20215_c1_seq1	4	IPR1223;Glycoside hydrolase, family 18, catalytic domain	PTHR11177:SF21 (PANTHER);Glycoside hydrolase, catalytic domain	PTHR11177 (PANTHER);Glycoside hydrolase, catalytic domain	IPR1579;Glycoside hydrolase, chitinase active site
comp20254_c1_seq1	4	IPR3593;AAA+ ATPase domain	IPR3959;ATPase, AAA-type, core	PTHR2374 (PANTHER);ATPase, AAA-type, core	IPR396;ATPase, AAA-type, conserved site
comp20255_c1_seq1	3	IPR21463;Protein of unknown function DUF3115	PTHR12618:SF15 (PANTHER);Protein of unknown function DUF3115	PTHR12618 (PANTHER);Protein of unknown function DUF3115	
comp20277_c0_seq1	4	IPR692;Fibrillarin	G3DSA:3.3.2.2 (GENE3D);Fibrillarin	PTHR1335:SF4 (PANTHER);Fibrillarin	PTHR1335 (PANTHER);Fibrillarin
comp20309_c0_seq1	4	IPR23797;RNA 3'-terminal phosphate cyclase domain	PTHR1196:SF (PANTHER);RNA 3'-terminal phosphate cyclase domain	IPR228;RNA 3'-terminal phosphate cyclase	IPR13792;RNA 3'-terminal phosphate cyclase/enolpyruvate transferase, alpha/beta

comp20353_c3_seq1	7	IPR977;DNA ligase, ATP-dependent	G3DSA:3.3.149.7 (GENE3D);DNA ligase, ATP-dependent	IPR1231;DNA ligase, ATP-dependent, central	G3DSA:3.3.47.3 (GENE3D); DNA ligase, ATP-dependent, central	PTHR1459:SF1 (PANTHER);DNA ligase, ATP-dependent, central	PTHR1459 (PANTHER);DNA ligase, ATP-dependent, central	IPR1659; DNA ligase, ATP-dependent, conserved site
comp20356_c0_seq1	6	IPR186;Small GTPase superfamily	IPR3579;Small GTPase superfamily, Rab type	IPR3578;Small GTPase superfamily, Rho type	IPR5225;Small GTP-binding protein domain	PTHR2472 (PANTHER);P-loop containing nucleoside triphosphate hydrolase	PTHR2472:SF1 (PANTHER);P-loop containing nucleoside triphosphate hydrolase	
comp20361_c0_seq1	2	IPR1593;Ribosomal protein S3Ae	PTHR1183 (PANTHER); Ribosomal protein S3Ae					
comp20399_c0_seq1	3	IPR597;Ribosomal protein L3	PTHR11363 (PANTHER); Ribosomal protein L3	PTHR11363:SF4 (PANTHER);Ribosomal protein L3				
comp20434_c1_seq1	4	IPR389;MIF4G-like, type 3	IPR1621;MIF4-like, type 1/2/3	PTHR23253 (PANTHER);MIF4-like, type 1/2/3	PTHR23253:SF9 (PANTHER);MIF4-like, type 1/2/3			
comp20455_c0_seq1	4	IPR425;Major intrinsic protein	IPR23271;Aquaporin-like	PTHR19139:SF9 (PANTHER);Major intrinsic protein	IPR22357; Major intrinsic protein, conserved site			
comp20484_c0_seq1	1	IPR2257;Chitin synthase III catalytic subunit						
comp20524_c4_seq1	2	PTHR1167:SF15 (PANTHER);null	PTHR1167 (PANTHER); null					
comp20544_c0_seq1	3	IPR24317;Dynein heavy chain, P-loop containing D4 domain	IPR26983;Dynein heavy chain	PTHR1676:SF28 (PANTHER);Dynein heavy chain				
comp20555_c0_seq1	2	IPR2836;PDCD5-related protein	PTHR184:SF (PANTHER); PDCD5-related protein					
comp20556_c1_seq1	5	G3DSA:1.1.8.6 (GENE3D);ATPase, AAA-type, core	G3DSA:1.2.272.1 (GENE3D);ATPase, AAA-type, core	PTHR11669:SF5 (PANTHER);P-loop containing nucleoside triphosphate hydrolase	PTHR11669 (PANTHER);P-loop containing nucleoside triphosphate hydrolase	IPR8921;DNA polymerase III, clamp loader complex, gamma/delta/delta subunit, C-terminal		
comp20562_c0_seq1	5	G3DSA:3.3.93.1 (GENE3D);null	IPR4364;Aminoacyl-tRNA synthetase, class II	IPR1815;Aminoacyl-tRNA synthetase, class II (D/K/N)-like	PTHR22594:SF4 (PANTHER);Aminoa	SSF55681 (SUPERFAMILY);Aminoacyl-tRNA		

			(D/K/N)		cyl-tRNA synthetase, class II (D/K/N)-like	synthetase, class II (D/K/N)-like	
comp20571_c0_seq1	6	IPR1557;L-lactate/malate dehydrogenase	IPR15955;Lactate dehydrogenase/glycoside hydrolase, family 4, C-terminal	IPR1236;Lactate/malate dehydrogenase, N-terminal	IPR22383;Lactate/malate dehydrogenase, C-terminal	PTHR1154:SF17 (PANTHER);NAD(P)-binding domain	IPR18177;L-lactate dehydrogenase, active site
comp20581_c0_seq1	4	IPR9851;Modifier of rudimentary, Modr	PTHR13678:SF2 (PANTHER);Modifier of rudimentary, Modr	PTHR13678 (PANTHER);Modifier of rudimentary, Modr	SSF14111 (SUPERFAMILY);Modifier of rudimentary, Modr		
comp20583_c1_seq1	4	IPR1951;Protein kinase A anchor protein, nuclear localisation signal domain	IPR997;RNA ligase/cyclic nucleotide phosphodiesterase	PTHR1336 (PANTHER);RNA ligase/cyclic nucleotide phosphodiesterase	PTHR1336:SF1 (PANTHER);RNA ligase/cyclic nucleotide phosphodiesterase		
comp20601_c0_seq1	1	IPR795;Elongation factor, GTP-binding domain					
comp20606_c0_seq1	3	IPR22669;Ribosomal protein L2, C-terminal	IPR2171;Ribosomal protein L2	PTHR13691:SF16 (PANTHER);Ribosomal protein L2			
comp20631_c0_seq1	3	IPR1128;Cytochrome P45	PTHR2435 (PANTHER);Cytochrome P45	PTHR2435:SF45 (PANTHER);Cytochrome P45			
comp20637_c0_seq1	3	G3DSA:3.1.1.1 (GENE3D);Ribosomal protein L18e/L15P	IPR39;Ribosomal protein L18e	IPR21132;Ribosomal protein L18e, conserved site			
comp20657_c0_seq1	2	IPR1976;Ribosomal protein S24e	PTHR1496:SF2 (PANTHER);Ribosomal protein S24e				
comp20676_c0_seq1	3	IPR26;NADH:ubiquinone oxidoreductase, chain 4, N-terminal	PTHR22773 (PANTHER);NADH:ubiquinone oxidoreductase, chain 4, N-terminal	PTHR22773:SF4 (PANTHER);NADH:ubiquinone oxidoreductase, chain 4, N-terminal			
comp20697_c0_seq1	2	PTHR21512 (PANTHER);null	PTHR21512:SF5 (PANTHER);null				
comp20699_c0_seq1	6	G3DSA:3.5.5.6 (GENE3D);null	G3DSA:3.3.41.1 (GENE3D);null	PTHR11552:SF64 (PANTHER);null	PTHR11552 (PANTHER);null	IPR172;Glucose-methanol-choline oxidoreductase, N-terminal	SSF5195 (SUPERFAMILY);Glucose-methanol-choline oxidoreductase, N-terminal
comp20699_c3_se	1	SSF54373 (SUPERFAMILY)					

q1		;null						
comp20715_c1_seq1	2	IPR3965;Fatty acid synthase	PTHR1982:SF9 (PANTHER); Acyl transferase domain					
comp20720_c0_seq1	1	PTHR2856:SF7 (PANTHER);DNA-directed RNA polymerase, subunit 2						
comp20721_c0_seq1	5	IPR1221;Phenol hydroxylase reductase	IPR1433;Oxidoreductase FAD/NAD(P)-binding	IPR8333;Oxidoreductase, FAD-binding domain	PTHR22924 (PANTHER);Oxidoreductase, FAD-binding domain	PTHR22924:SF15 (PANTHER);Oxidoreductase, FAD-binding domain		
comp20727_c0_seq1	1	PTHR22914:SF9 (PANTHER);null						
comp20743_c0_seq1	7	IPR5711;Ribosomal protein S5, eukaryotic/archaeal	IPR14721;Ribosomal protein S5 domain 2-type fold, subgroup	IPR1472;Double-stranded RNA-binding domain	IPR1381;Ribosomal protein S5, N-terminal	IPR5324;Ribosomal protein S5, C-terminal	SSF54768 (SUPERFAMILY);Ribosomal protein S5, N-terminal	IPR2568;Ribosomal protein S5 domain 2-type fold
comp20747_c0_seq1	1	IPR19344;Mitochondrial F1-F ATP synthase subunit F, predicted						
comp20769_c2_seq1	1	IPR25724;GAG-pre-integrase domain						
comp20777_c5_seq1	2	PTHR22763:SF3 (PANTHER);Zinc finger, RING/FYVE/PHD-type	PTHR22763 (PANTHER); Zinc finger, RING/FYVE/PHD-type					
comp20836_c0_seq1	1	IPR24332;MOZART2 family						
comp20840_c0_seq1	3	IPR1194;DENN domain	PTHR12296 (PANTHER); DENN domain	IPR5113;uDENN domain				
comp20859_c0_seq1	2	IPR1339;TIP49, C-terminal	IPR27238;RuvB-like					
comp20892_c1_seq1	1	PTHR11668:SF237 (PANTHER);Metallo-dependent phosphatase-like						
comp20914_c0_seq1	2	PTHR19856 (PANTHER);WD4/YVTN repeat-like-containing domain	PTHR19856:SF (PANTHER); WD4/YVTN repeat-like-containing domain					
comp20938_c0_seq1	1	IPR1828;Ribosomal protein S3, conserved site						

comp20964_c1_seq1	2	IPR4487;Clp protease, ATP-binding subunit ClpX	PTHR11262 (PANTHER); Clp protease, ATP-binding subunit ClpX			
comp20965_c0_seq1	4	IPR1452;DNA primase, small subunit, eukaryotic/archaeal	G3DSA:3.9.92.1 (GENE3D);DNA primase, small subunit, eukaryotic/archaeal	IPR2755;DNA primase, small subunit	SSF56747 (SUPERFAMILY);DNA primase, small subunit, eukaryotic/archaeal	
comp20969_c2_seq1	1	IPR44;NADH:ubiquinone/plastoquinone oxidoreductase, chain 3				
comp20977_c0_seq1	2	IPR196;Peptidase C13, legumain	IPR28361;GPI-anchor transamidase			
comp20980_c0_seq1	3	IPR15974;Ribosomal protein L19/L19e, domain 3	IPR196;Ribosomal protein L19/L19e domain	PTHR1722 (PANTHER);Ribosomal protein L19/L19e domain		
comp21000_c0_seq1	3	IPR911;Ribosomal protein L11/L12	IPR2783;Ribosomal protein L11, C-terminal	IPR2785;Ribosomal protein L11, conserved site		
comp21029_c0_seq1	1	SignalP-TM (SIGNALP_EUK);null				
comp21029_c2_seq1	1	PTHR24115:SF9 (PANTHER);null				
comp21035_c0_seq1	3	IPR195;Translation initiation factor SUI1	PTHR1388:SF1 (PANTHER); Translation initiation factor SUI1	PTHR1388 (PANTHER);Translation initiation factor SUI1		
comp21051_c1_seq1	4	IPR836;Phosphoribosyltransferase domain	IPR2957;Phosphoribosyltransferase-like	PTHR121:SF36 (PANTHER);Phosphoribosyltransferase-like	PTHR121 (PANTHER);Phosphoribosyltransferase-like	
comp21061_c0_seq1	2	PF366 (PFAM);null	IPR2457;Nucleoplasmin core domain			
comp21071_c0_seq1	4	IPR23395;Mitochondrial carrier domain	IPR1818;Mitochondrial substrate/solute carrier	PTHR2489:SF193 (PANTHER);Mitochondrial substrate/solute carrier	PTHR2489 (PANTHER);Mitochondrial substrate/solute carrier	
comp21083_c0_seq1	3	IPR2553;Clathrin/coatomer adaptor, adaptin-like, N-terminal	PTHR2278 (PANTHER); Armadillo-like helical	IPR1717;Adaptor protein complex AP-1, gamma subunit		
comp21084_c1_seq1	2	IPR249;Aflatoxin biosynthesis regulatory protein	PTHR325:SF29 (PANTHER); Zn(2)-C6 fungal-type DNA-binding domain			
comp21104_c0_seq1	5	IPR213;Cyclophilin-type peptidyl-prolyl cis-trans isomerase domain	IPR29;Cyclophilin-like domain	PTHR1171:SF63 (PANTHER);Cyclophilin-type peptidyl-prolyl cis-	IPR24936; Cyclophilin-type peptidyl-	IPR2892;Cyclophilin-type peptidyl-

				trans isomerase domain	prolyl cis-trans isomerase	prolyl cis-trans isomerase, conserved site
comp21112_c1_seq1	1	PTHR22838:SF (PANTHER);null				
comp21118_c0_seq1	1	IPR3197;Cytochrome b-c1 complex subunit 7				
comp21139_c0_seq1	3	IPR7219;Transcription factor domain, fungi	PTHR311:SF16 (PANTHER);Transcription factor domain, fungi	PTHR311 (PANTHER);Transcription factor domain, fungi		
comp21140_c2_seq1	4	IPR15425;Formin, FH2 domain	PTHR23213 (PANTHER);Formin, FH2 domain	PTHR23213:SF3 (PANTHER);Formin, FH2 domain	SSF11447 (SUPERFAMILY);Formin, FH2 domain	
comp21168_c0_seq1	3	IPR144;Heat shock protein Hsp9 family	PTHR11528:SF44 (PANTHER);Heat shock protein Hsp9 family	SSF11942 (SUPERFAMILY);Heat shock protein Hsp9 family		
comp21207_c2_seq1	1	PF13147 (PFAM);null				
comp21210_c0_seq1	3	IPR6941;Ribonuclease CAF1	PTHR1797 (PANTHER);Ribonuclease H-like domain	PTHR1797:SF (PANTHER);Ribonuclease H-like domain		
comp21213_c0_seq1	4	IPR1876;Zinc finger, RanBP2-type	PTHR23238:SF25 (PANTHER);Zinc finger, RanBP2-type	PTHR23238 (PANTHER);Zinc finger, RanBP2-type	SSF929 (SUPERFAMILY);Zinc finger, RanBP2-type	
comp21223_c1_seq1	1	PTHR2285 (PANTHER);WD4 repeat				
comp21242_c1_seq1	3	IPR17941;Rieske [2Fe-2S] iron-sulphur domain	PTHR21496 (PANTHER);Rieske [2Fe-2S] iron-sulphur domain	PTHR21496:SF2 (PANTHER);Rieske [2Fe-2S] iron-sulphur domain		
comp21252_c0_seq1	3	IPR1179;Domain of unknown function DUF165	PTHR18934 (PANTHER);Domain of unknown function DUF165	PTHR18934:SF12 (PANTHER);Domain of unknown function DUF165		
comp21281_c0_seq1	4	G3DSA:1.1.287.7 (GENE3D);null	IPR4178;Calmodulin-binding domain	IPR15449;Potassium channel, calcium-activated, SK	PTHR1153:SF4 (PANTHER);Potassium channel, calcium-activated, SK	
comp21293_c0_seq1	1	PTHR22883:SF23 (PANTHER);Zinc finger, DHHC-type, palmitoyltransferase				

comp21313_c0_seq1	1	SignalP-noTM (SIGNALP_GRAM_NEGATIVE);null					
comp21315_c2_seq1	1	PTHR11871:SF (PANTHER);Protein phosphatase 2A, regulatory subunit PR55					
comp21359_c0_seq1	2	G3DSA:3.9.143.1 (GENE3D);null	PTHR23115:SF126 (PANTHER);null				
comp21376_c0_seq1	3	IPR1269;tRNA-dihydrouridine synthase	PTHR1182:SF8 (PANTHER);tRNA-dihydrouridine synthase	SSF51395 (SUPERFAMILY);tRNA-dihydrouridine synthase			
comp21385_c4_seq1	1	PTHR2458:SF (PANTHER);Protein kinase domain					
comp21395_c0_seq1	4	IPR2423;Chaperonin Cpn6/TCP-1 family	IPR27413;GroEL-like equatorial domain	IPR12719;T-complex protein 1, gamma subunit	SSF48592 (SUPERFAMILY);T-complex protein 1, gamma subunit		
comp21425_c1_seq1	3	IPR6134;DNA-directed DNA polymerase, family B, multifunctional domain	PTHR1322:SF11 (PANTHER);DNA-directed DNA polymerase, family B, multifunctional domain	SSF56672 (SUPERFAMILY);DNA-directed DNA polymerase, family B, multifunctional domain			
comp21425_c3_seq1	6	IPR6172;DNA-directed DNA polymerase, family B	TIGR592 (TIGRFAM);Ribonuclease H-like domain	IPR6133;DNA-directed DNA polymerase, family B, exonuclease domain	G3DSA:1.1.287.69 (GENE3D);DNA-directed DNA polymerase, family B, exonuclease domain	IPR23211;DNA polymerase, palm domain	IPR17964;DNA-directed DNA polymerase, family B, conserved site
comp21433_c0_seq1	2	PTHR235 (PANTHER);EF-hand domain	PTHR235:SF18 (PANTHER);EF-hand domain				
comp21435_c0_seq2	1	IPR1982;Lambda repressor-like, DNA-binding domain					
comp21441_c0_seq1	3	IPR4888;Glycoside hydrolase, family 63	PTHR1412:SF6 (PANTHER);Glycoside hydrolase, family 63	PTHR1412 (PANTHER);Glycoside hydrolase, family 63			
comp21441_c2_seq1	3	IPR3342;Glycosyltransferase, family 39	IPR274;Dolichyl-phosphate-mannose-protein mannosyltransferase 1/5	IPR275;Glycosyltransferase 39 like			

comp21442_c2_seq1	1	PTHR11384:SF13 (PANTHER);ABC transporter type 1, transmembrane domain			
comp21444_c0_seq1	2	PTHR2391 (PANTHER);GNAT domain	PTHR2391:SF71 (PANTHER); GNAT domain		
comp21450_c0_seq1	1	PTHR1171:SF156 (PANTHER);Nucleotide-binding alpha-beta plait domain			
comp21477_c0_seq1	4	IPR158;Calreticulin/calnexin	IPR1332;Concanavalin A-like lectin/glucanase domain	PTHR1173:SF8 (PANTHER);Concanavalin A-like lectin/glucanase domain	IPR18124; Calreticulin/calnexin, conserved site
comp21485_c0_seq1	1	PTHR118:SF695 (PANTHER);S-adenosyl-L-methionine-dependent methyltransferase			
comp21485_c1_seq1	1	PTHR118:SF766 (PANTHER);null			
comp21499_c0_seq1	4	IPR13783;Immunoglobulin-like fold	IPR1579;MHC class I, alpha chain, C-terminal	PTHR16675 (PANTHER);MHC class I, alpha chain, C-terminal	PTHR16675:SF149 (PANTHER);MHC class I, alpha chain, C-terminal
comp21506_c2_seq1	1	IPR21983;PRP8 domain IV core			
comp21506_c5_seq1	1	IPR12592;PROCN domain			
comp21512_c0_seq1	2	PTHR1489:SF2 (PANTHER);null	PTHR1489 (PANTHER); null		
comp21566_c0_seq1	2	PTHR15889:SF1 (PANTHER);null	PTHR15889 (PANTHER); null		
comp21578_c0_seq1	2	PTHR2458:SF22 (PANTHER);Protein kinase domain	IPR17441;Protein kinase, ATP binding site		
comp21600_c0_seq1	1	IPR4273;Dynein heavy chain domain			
comp21607_c0_seq1	3	IPR524;Snf7 family	PTHR1476 (PANTHER); Snf7 family	PTHR1476:SF4 (PANTHER);Snf7 family	
comp21610_c0_seq1	1	SSF144217 (SUPERFAMILY);null			
comp21614_c1_seq1	1	PTHR1124:SF3 (PANTHER);null			
comp21632_c0_seq1	3	IPR16181;Acyl-CoA N-acyltransferase	PTHR1545:SF22 (PANTHER); Acyl-CoA N-acyltransferase	PTHR1545 (PANTHER);Acyl-CoA N-acyltransferase	

comp21635_c0_seq1	5	IPR192;Aminotransferase class V domain	IPR15421;Pyridoxal phosphate-dependent transferase, major region, subdomain 1	IPR15422;Pyridoxal phosphate-dependent transferase, major region, subdomain 2	IPR1111;Kynureninase	IPR15424;Pyridoxal phosphate-dependent transferase	
comp21635_c1_seq1	1	PTHR1484:SF (PANTHER);Kynureninase					
comp21646_c1_seq1	6	IPR763;Catalase-peroxidase haem	IPR216;Haem peroxidase, plant/fungal/bacterial	G3DSA:1.1.52.1 (GENE3D);Haem peroxidase, plant/fungal/bacterial	PTHR3555 (PANTHER);Haem peroxidase, plant/fungal/bacterial	PTHR3555:SF (PANTHER);Haem peroxidase, plant/fungal/bacterial	IPR1255;Haem peroxidase
comp21660_c0_seq1	3	IPR175;Sodium:neurotransmitter symporter	PTHR11616:SF17 (PANTHER);Sodium:neurotransmitter symporter	SSF1617 (SUPERFAMILY);Sodium:neurotransmitter symporter			
comp21668_c0_seq1	4	IPR825;P-type ATPase, A domain	IPR414;Cation-transporting P-type ATPase, N-terminal	PTHR2493:SF255 (PANTHER);Cation-transporting P-type ATPase, N-terminal	SSF81653 (SUPERFAMILY);Cation-transporting P-type ATPase, N-terminal		
comp21669_c1_seq1	2	PTHR23111:SF26 (PANTHER);Nucleotide-binding alpha-beta plait domain	PTHR23111 (PANTHER);Nucleotide-binding alpha-beta plait domain				
comp21682_c1_seq1	2	IPR27512;Eukaryotic translation initiation factor 3 subunit A	PTHR145 (PANTHER);Eukaryotic translation initiation factor 3 subunit A				
comp21683_c0_seq1	4	IPR2222;Ribosomal protein S19/S15	IPR23575;Ribosomal protein S19, superfamily	PTHR1188:SF2 (PANTHER);Ribosomal protein S19/S15	IPR2934;Ribosomal protein S19 conserved site		
comp21685_c0_seq1	4	IPR9145;U2 auxiliary factor small subunit	IPR571;Zinc finger, CCCH-type	PTHR1262:SF5 (PANTHER);Zinc finger, CCCH-type	SSF9229 (SUPERFAMILY);Zinc finger, CCCH-type		
comp21686_c2_seq1	2	PTHR12563:SF1 (PANTHER);null	IPR22284;Glycerol-3-phosphate O-acyltransferase/Dihydroxyacetone phosphate acyltransferase				
comp21717_c0_seq1	3	PTHR2473:SF313 (PANTHER);Small GTPase superfamily	PTHR2473 (PANTHER);Small GTPase superfamily	PS51419 (PROSITE_PROFILES);Small GTPase superfamily			
comp21739_c2_seq1	2	IPR6762;Gtr1/RagA G protein	PTHR11259:SF2 (PANTHER);				

			Gtr1/RagA G protein		
comp21803_c0_seq1	1	IPR6811;RNA polymerase II subunit A			
comp21829_c0_seq1	3	IPR12571;Mitochondrial distribution and morphology protein family 31/32, fungi	PTHR3168 (PANTHER); Mitochondrial distribution and morphology protein family 31/32, fungi	PTHR3168:SF (PANTHER);Mitochondrial distribution and morphology protein family 31/32, fungi	
comp21835_c0_seq1	1	IPR8278;4'-phosphopantetheinyl transferase superfamily			
comp21844_c0_seq1	3	IPR197;ATP-dependent Clp protease proteolytic subunit	IPR23562;Clp protease proteolytic subunit /Translocation-enhancing protein TepA	IPR18215;ClpP, active site	
comp21846_c0_seq1	1	PTHR13387 (PANTHER);null			
comp21847_c1_seq1	1	IPR21771;Triacylglycerol lipase			
comp21849_c0_seq1	1	PTHR2473:SF38 (PANTHER);Small GTP-binding protein domain			
comp21850_c1_seq1	4	IPR2898;Glycosyltransferase subfamily 4, N-terminal domain	PTHR12526 (PANTHER); Glycosyltransferase subfamily 4, N-terminal domain	IPR2754;Alpha-1,3/1,6-mannosyltransferase ALG2	SSF53756 (SUPERFAMILY);Alpha-1,3/1,6-mannosyltransferase ALG2
comp21885_c3_seq1	2	PTHR2275:SF3 (PANTHER);null	IPR254;Inorganic polyphosphate /ATP-NAD kinase		
comp21899_c1_seq1	3	IPR3111;Peptidase S16, lon N-terminal	PTHR146:SF23 (PANTHER); Peptidase S16, lon N-terminal	IPR2765;Lon protease	
comp21902_c0_seq1	1	PTHR1933:SF165 (PANTHER);HTH CenpB-type DNA-binding domain			
comp21903_c1_seq1	1	PTHR19446:SF34 (PANTHER);Reverse transcriptase domain			
comp21914_c0_seq1	3	IPR15374;Chs5p-Arf1p binding	PTHR31975 (PANTHER); Chs5p-Arf1p binding	PTHR31975:SF1 (PANTHER);Chs5p-Arf1p binding	
comp21932_c1_seq1	3	IPR1544;Vps5 C-terminal	PTHR1555:SF84 (PANTHER); Vps5 C-terminal	PTHR1555 (PANTHER);Vps5 C-terminal	

comp21934_c0_seq1	3	IPR1122;Arrestin C-terminal-like domain	PTHR11188 (PANTHER); Arrestin C-terminal-like domain	PTHR11188:SF46 (PANTHER);Arrestin C-terminal-like domain		
comp21950_c0_seq1	3	IPR764;CP2 transcription factor	PTHR1137:SF14 (PANTHER); CP2 transcription factor	PTHR1137 (PANTHER);CP2 transcription factor		
comp21966_c0_seq1	3	IPR4839;Aminotransferase, class I/classII	PTHR11751:SF3 (PANTHER); Pyridoxal phosphate-dependent transferase, major region, subdomain 2	PTHR11751 (PANTHER);Pyridoxal phosphate-dependent transferase, major region, subdomain 2		
comp21985_c1_seq1	2	PTHR31313:SF7 (PANTHER);Transcription factor domain, fungi	PTHR31313 (PANTHER); Transcription factor domain, fungi			
comp21988_c1_seq1	5	IPR6553;Leucine-rich repeat, cysteine-containing subtype	PF13516 (PFAM);Leucine-rich repeat, cysteine-containing subtype	PTHR23125 (PANTHER);Leucine-rich repeat, cysteine-containing subtype	PTHR23125:SF231 (PANTHER);Leucine-rich repeat, cysteine-containing subtype	SSF5247 (SUPERFAMILY);Leucine-rich repeat, cysteine-containing subtype
comp21990_c1_seq1	2	IPR2849;Small GTPase superfamily, Ras type	PTHR247:SF (PANTHER); Small GTPase superfamily, Ras type			
comp21993_c0_seq1	2	PTHR23244 (PANTHER);PAS domain	PTHR23244:SF6 (PANTHER); PAS domain			
comp22038_c5_seq1	4	IPR198;Rho GTPase-activating protein domain	PTHR24215:SF1 (PANTHER); Rho GTPase-activating protein domain	PTHR24215 (PANTHER);Rho GTPase-activating protein domain	IPR8936;Rho GTPase activation protein	
comp22070_c0_seq1	2	IPR15916;Galactose oxidase, beta-propeller	IPR1143;Galactose oxidase/kelch, beta-propeller			
comp22072_c0_seq1	2	PF1346 (PFAM);null	PTHR12126:SF1 (PANTHER); NAD(P)-binding domain			
comp22077_c0_seq1	2	IPR8429;Cleft lip and palate transmembrane 1	PTHR21347:SF (PANTHER); Cleft lip and palate transmembrane 1			
comp22097_c0_seq1	1	IPR27156;AP-2 complex subunit sigma				

comp22101_c1_seq1	1	IPR27121;Vacuolar protein sorting-associated protein 33			
comp22134_c1_seq1	2	IPR12312;Haemerythrin/HHE cation-binding motif	G3DSA:1.2.12.52 (GENE3D);Haemerythrin/HHE cation-binding motif		
comp22135_c0_seq1	1	IPR333;Sarcoplasmic/endoplasmic reticulum calcium ATPase 3			
comp22136_c0_seq1	4	IPR3829;Pirin, N-terminal domain	IPR8778;Pirin, C-terminal domain	PTHR1393:SF8 (PANTHER);Pirin, C-terminal domain	IPR1293;Pirin
comp22145_c1_seq1	2	PTHR11662 (PANTHER);Major facilitator superfamily	PTHR11662:SF172 (PANTHER); Major facilitator superfamily		
comp22146_c1_seq1	2	PTHR11139 (PANTHER);null	PTHR11139:SF1 (PANTHER); null		
comp22172_c0_seq1	2	IPR26827;Proteasome component ECM29/Translational activator GCN1	IPR24372;Proteasome stabiliser ECM29		
comp22213_c0_seq1	1	IPR25476;Helitron helicase-like domain			
comp22244_c0_seq1	1	PTHR23244:SF271 (PANTHER);Kelch-type beta propeller			
comp22272_c0_seq2	1	PTHR24343:SF98 (PANTHER);Protein kinase domain			
comp22353_c1_seq1	2	PTHR2455:SF1 (PANTHER);Protein kinase domain	IPR3527;Mitogen-activated protein (MAP) kinase, conserved site		
comp22356_c0_seq1	3	IPR2454;Gamma tubulin	IPR18316;Tubulin/FtsZ, 2-layer sandwich domain	IPR828;Tubulin/FtsZ, C-terminal	
comp22356_c0_seq2	1	IPR17975;Tubulin, conserved site			
comp22356_c1_seq1	1	IPR23123;Tubulin, C-terminal			
comp22384_c0_seq1	3	IPR2891;Adenylylsulphate kinase	PTHR1155 (PANTHER); Adenylylsulphate kinase	PTHR1155:SF2 (PANTHER);Adenylylsulphate kinase	
comp22406_c0_seq1	2	IPR27299;GIY-YIG domain	IPR1896;Nuclease-associated modular DNA-binding 1		

comp22430_c0_seq1	1	PTHR22781 (PANTHER);Armadillo-like helical					
comp22440_c1_seq2	2	IPR1683;Phox homologous domain	PTHR1555:SF133 (PANTHER); Phox homologous domain				
comp22447_c1_seq1	2	IPR22816;Condensin complex subunit 2/barren	PTHR1318 (PANTHER); Condensin complex subunit 2/barren				
comp22460_c0_seq1	1	PTHR1548 (PANTHER);Nucleotide-binding alpha-beta plait domain					
comp22517_c0_seq1	6	IPR6649;Ribonucleoprotein LSM domain, eukaryotic/archaea-type	G3DSA:2.3.3.1 (GENE3D);Ribonucleoprotein LSM domain, eukaryotic/archaea-type	IPR1163;Ribonucleoprotein LSM domain	PTHR1553 (PANTHER);Ribonucleoprotein LSM domain	PTHR1553:SF2 (PANTHER);Ribonucleoprotein LSM domain	IPR192; Like-Sm (LSM) domain
comp22587_c0_seq1	1	IPR29498;Prion-inhibition and propagation, HeLo domain					
comp22604_c0_seq1	1	PTHR22847:SF399 (PANTHER);null					
comp22650_c3_seq1	1	IPR12476;GLE1-like					
comp22677_c0_seq1	2	IPR3788;Putative S-adenosyl-L-methionine-dependent methyltransferase MidA	PTHR1249:SF6 (PANTHER); Putative S-adenosyl-L-methionine-dependent methyltransferase MidA				
comp22751_c0_seq1	3	IPR225;Armadillo	PTHR23315:SF7 (PANTHER); Armadillo-like helical	PTHR23315 (PANTHER);Armadillo-like helical			
comp22754_c0_seq1	1	PTHR23244:SF283 (PANTHER);null					
comp22762_c0_seq1	5	G3DSA:3.3.42.6 (GENE3D);null	IPR514;eRF1 domain 1/Pelota-like	IPR2449;Peptide Chain Release Factor eRF1/aRF1, N-terminal	PTHR1113:SF7 (PANTHER);Peptide Chain Release Factor eRF1/aRF1, N-terminal	IPR443;Peptide chain release factor eRF1/aRF1	
comp22772_c2_seq1	3	PF773 (PFAM);null	PTHR23355 (PANTHER); null	PTHR23355:SF35 (PANTHER);null			

comp22772_c1_seq1	1	PTHR32514:SF19 (PANTHER);Zn(2)-C6 fungal-type DNA-binding domain				
comp22774_c1_seq1	3	IPR4871;Cleavage/polyadenylation specificity factor, A subunit, C-terminal	PTHR1644 (PANTHER);Cleavage/polyadenylation specificity factor, A subunit, C-terminal	PTHR1644:SF1 (PANTHER);Cleavage/polyadenylation specificity factor, A subunit, C-terminal		
comp22798_c0_seq2	5	IPR12385;Prephenate dehydrogenase, fungal	IPR399;Prephenate dehydrogenase	G3DSA:1.1.366.1 (GENE3D);Prephenate dehydrogenase	PTHR21363 (PANTHER);Prephenate dehydrogenase	PTHR21363:SF (PANTHER);Prephenate dehydrogenase
comp22829_c0_seq1	3	PTHR11922 (PANTHER);Rossmann-like alpha/beta/alpha sandwich fold	PTHR11922:SF2 (PANTHER);Rossmann-like alpha/beta/alpha sandwich fold	IPR25777;GMP synthetase ATP pyrophosphatase domain		
comp22840_c0_seq1	1	PF5729 (PFAM);null				
comp22908_c2_seq1	3	IPR858;PPPDE putative peptidase domain	PTHR12378 (PANTHER);PPPDE putative peptidase domain	PTHR12378:SF7 (PANTHER);PPPDE putative peptidase domain		
comp22919_c2_seq1	3	IPR6594;LIS1 homology motif	PTHR22846:SF2 (PANTHER);LIS1 homology motif	PTHR22846 (PANTHER);LIS1 homology motif		
comp22928_c1_seq1	3	IPR1356;Homeobox domain	PTHR24325:SF15 (PANTHER);Homeodomain-like	PTHR24325 (PANTHER);Homeodomain-like		
comp22934_c0_seq2	3	IPR19133;Mitochondrial inner membrane protein Mitofilin	PTHR15415:SF7 (PANTHER);Mitochondrial inner membrane protein Mitofilin	PTHR15415 (PANTHER);Mitochondrial inner membrane protein Mitofilin		
comp23041_c3_seq1	3	IPR734;Ribosome biogenesis protein BMS1/TSR1, C-terminal	PTHR12858:SF1 (PANTHER);Ribosome biogenesis protein BMS1/TSR1, C-terminal	PTHR12858 (PANTHER);Ribosome biogenesis protein BMS1/TSR1, C-terminal		
comp23123_c0_seq1	1	G3DSA:2.6.12.62 (GENE3D);null				
comp23131_c0_seq1	1	IPR6722;Sedlin				

comp23139_c0_seq1	3	IPR426;Proteasome alpha-subunit, N-terminal domain	PTHR11599:SF14 (PANTHER); Nucleophile aminohydrolases, N-terminal	IPR23332;Proteasome A-type subunit
comp23153_c0_seq1	1	PTHR19877 (PANTHER);WD4/YVTN repeat-like-containing domain		
comp23172_c0_seq1	2	PTHR13923 (PANTHER);WD4/YVTN repeat-like-containing domain	PTHR13923:SF11 (PANTHER); WD4/YVTN repeat-like-containing domain	
comp23230_c1_seq1	3	IPR118;Citron-like	PTHR12673 (PANTHER); Citron-like	PTHR12673:SF63 (PANTHER);Citron-like
comp23230_c2_seq1	1	IPR219;Dbl homology (DH) domain		
comp23253_c0_seq1	3	PF1387 (PFAM);P-loop containing nucleoside triphosphate hydrolase	PTHR1887:SF34 (PANTHER); P-loop containing nucleoside triphosphate hydrolase	PTHR1887 (PANTHER);P-loop containing nucleoside triphosphate hydrolase
comp23253_c6_seq1	1	IPR18999;RNA helicase UPF1, UPF2-interacting domain		
comp23268_c0_seq1	2	IPR2636;Calcium/calmodulin-dependent/calcium-dependent protein kinase	PTHR24347:SF134 (PANTHER); Calcium/calmodulin-dependent/calcium-dependent protein kinase	
comp23274_c2_seq1	2	IPR2554;Protein phosphatase 2A, regulatory B subunit, B56	PTHR1257:SF14 (PANTHER); Protein phosphatase 2A, regulatory B subunit, B56	
comp23283_c1_seq1	1	PTHR11669:SF9 (PANTHER);null		
comp23316_c0_seq1	3	IPR8968;Clathrin adaptor, mu subunit, C-terminal	PTHR11998:SF11 (PANTHER); Clathrin adaptor, mu subunit, C-terminal	IPR28565;Mu homology domain
comp23316_c3_seq1	1	IPR1392;Clathrin adaptor, mu subunit		
comp23341_c1_seq1	3	IPR1549;Domain of unknown function DUF19	PTHR1856:SF (PANTHER); Domain of unknown	IPR1555;Coronin

			function DUF19			
comp23441_c1_seq1	2	G3DSA:1.2.16.1 (GENE3D);null	IPR2322;Type IV secretion system, VirB5-domain			
comp23453_c1_seq1	3	IPR184;Isocitrate and isopropylmalate dehydrogenases family	PTHR11835:SF42 (PANTHER);Isocitrate and isopropylmalate dehydrogenases family	IPR19818;Isocitrate/isopropylmalate dehydrogenase, conserved site		
comp23467_c3_seq1	5	IPR644;CBS domain	G3DSA:3.1.58.1 (GENE3D);CBS domain	PTHR1378:SF23 (PANTHER);CBS domain	PTHR1378 (PANTHER);CBS domain	SSF54631 (SUPERFAMILY);CBS domain
comp23509_c0_seq1	2	PTHR1163 (PANTHER);Aldehyde dehydrogenase N-terminal domain	PTHR1163:SF8 (PANTHER); Aldehyde dehydrogenase N-terminal domain			
comp23510_c2_seq1	3	IPR13922;Cyclin PHO8-like	PTHR15615 (PANTHER); Cyclin PHO8-like	PTHR15615:SF9 (PANTHER);Cyclin PHO8-like		
comp23516_c2_seq1	4	IPR2876;Transcriptional regulator TACO1-like	IPR17856;Integrase, N-terminal zinc-binding domain-like	PTHR12532:SF (PANTHER);Integrase, N-terminal zinc-binding domain-like	IPR2972;YebC-like	
comp23534_c2_seq3	2	IPR241;Ran GTPase	PTHR2473:SF132 (PANTHER); P-loop containing nucleoside triphosphate hydrolase			
comp23558_c0_seq2	1	IPR2562;3'-5' exonuclease domain				
comp23578_c1_seq1	1	PTHR11134:SF3 (PANTHER);Armadillo-like helical				
comp23583_c0_seq1	1	IPR13218;Kinetochore-associated protein Dsn1/Mis13				
comp23608_c2_seq1	1	PTHR23115:SF99 (PANTHER);Elongation factor, GTP-binding domain				
comp23618_c1_seq1	1	PTHR1193:SF2 (PANTHER);TIP49, C-terminal				
comp23638_c0_seq1	2	PTHR18937:SF148 (PANTHER);P-loop containing nucleoside triphosphate hydrolase	PTHR18937 (PANTHER); P-loop containing nucleoside triphosphate hydrolase			

comp23695_c0_seq1	5	IPR1199;Tetratricopeptide-like helical domain	IPR24983;CHAT domain	PTHR19959:SF121 (PANTHER);Tetratricopeptide-like helical domain	PTHR19959 (PANTHER);Tetratricopeptide-like helical domain	SSF48452 (SUPERFAMILY);Tetratricopeptide-like helical domain
comp23716_c0_seq1	1	PTHR12272 (PANTHER);Zinc finger, CCCH-type				
comp23769_c1_seq1	1	PTHR11668:SF233 (PANTHER);Metallo-dependent phosphatase-like				
comp23779_c0_seq1	1	PTHR22847:SF371 (PANTHER);WD4/YVTN repeat-like-containing domain				
comp23794_c0_seq1	1	IPR35;Amphiphysin				
comp23850_c1_seq1	2	IPR13865;Protein of unknown function DUF1754, eukaryotic	PTHR13282:SF6 (PANTHER); Protein of unknown function DUF1754, eukaryotic			
comp23857_c0_seq1	5	IPR15;SANT/Myb domain	PTHR1641:SF485 (PANTHER); Homeodomain-like	PTHR1641 (PANTHER);Homeodomain-like	IPR1793;Myb domain	IPR17877;Myb-like domain
comp23922_c0_seq1	4	G3DSA:3.3.3.3 (GENE3D);Heat shock protein 7kD, peptide-binding domain	G3DSA:3.9.64.1 (GENE3D);Heat shock protein 7kD, peptide-binding domain	PTHR19375:SF78 (PANTHER);Heat shock protein 7kD, C-terminal domain	IPR18181; Heat shock protein 7, conserved site	
comp23973_c0_seq1	2	IPR23398;Translation Initiation factor eIF- 4e-like domain	IPR14;Translation Initiation factor eIF- 4e			
comp23977_c0_seq1	4	IPR897;Signal recognition particle, SRP54 subunit, GTPase domain	IPR4125;Signal recognition particle, SRP54 subunit, M-domain	PTHR11564:SF5 (PANTHER);Signal recognition particle, SRP54 subunit, M-domain	PTHR11564 (PANTHER);Signal recognition particle, SRP54 subunit, M-domain	
comp24012_c0_seq1	3	IPR1975;Ribosomal protein L4e	G3DSA:3.1.2.9 (GENE3D);Ribosomal protein L4e	PTHR1666 (PANTHER);Ribosomal protein L4e		
comp24031_c0_seq1	2	PTHR22847 (PANTHER);WD4 repeat	PTHR22847:SF353 (PANTHER); WD4 repeat			

comp24069_c0_seq1	1	PTHR2431:SF75 (PANTHER);P-loop containing nucleoside triphosphate hydrolase				
comp24135_c2_seq1	1	IPR2749;GroEL-like apical domain				
comp24148_c0_seq1	3	IPR375;Dynamin central domain	PTHR11566:SF21 (PANTHER);Dynamin central domain	IPR22812;Dynamin superfamily		
comp24155_c1_seq1	1	IPR3445;Cation transporter				
comp24204_c0_seq2	2	PF13358 (PFAM);null	PTHR2322 (PANTHER);null			
comp24285_c4_seq1	1	IPR1584;Integrase, catalytic core				
comp24322_c0_seq1	1	PTHR22847:SF321 (PANTHER);WD4/YVTN repeat-like-containing domain				
comp24335_c1_seq1	1	IPR22124;Protein of unknown function DUF3659				
comp24423_c0_seq1	2	PTHR1199:SF127 (PANTHER);Protein kinase domain	PTHR1199 (PANTHER);Protein kinase domain			
comp24476_c1_seq1	1	IPR1229;Jacalin-like lectin domain				
comp24480_c1_seq1	2	PTHR23323 (PANTHER);null	PTHR23323:SF26 (PANTHER);null			
comp24488_c0_seq1	1	PTHR19852 (PANTHER);WD4/YVTN repeat-like-containing domain				
comp24491_c0_seq1	1	IPR18982;RQC domain				
comp24491_c2_seq1	2	IPR4589;DNA helicase, ATP-dependent, RecQ type	IPR2464;DNA/RNA helicase, ATP-dependent, DEAH-box type, conserved site			
comp24511_c0_seq1	5	IPR6411;Fructose-bisphosphate aldolase, class II, yeast/E. coli subtype	IPR771;Ketose-bisphosphate aldolase, class-II	PTHR3559:SF (PANTHER);Ketose-bisphosphate aldolase, class-II	PTHR3559 (PANTHER);Ketose-bisphosphate aldolase, class-II	SSF51569 (SUPERFAMILY);Ketose-bisphosphate aldolase, class-II
comp24530_c0_seq1	1	PTHR11599:SF13 (PANTHER);Proteasome alpha-subunit, N-				

			terminal domain				
comp24748_c1_seq1	1		IPR8266;Tyrosine-protein kinase, active site				
comp24809_c0_seq1	2		PR76 (PRINTS);null	PTHR11811:SF25 (PANTHER);NAD(P)-binding domain			
comp24881_c0_seq2	1		IPR7274;Ctr copper transporter				
comp24920_c1_seq1	2		PTHR1566:SF59 (PANTHER);null	PTHR1566 (PANTHER);null			
comp24946_c2_seq1	5		IPR2453;Beta tubulin	IPR217;Tubulin	IPR38;Tubulin/FtsZ, GTPase domain	PTHR11588:SF47 (PANTHER);Tubulin/FtsZ, GTPase domain	IPR13838;Beta tubulin, autoregulation binding site
comp24957_c0_seq1	1		IPR1646;Coatomer beta subunit (COPB1)				
comp24971_c4_seq1	4		IPR38;14-3-3 protein	IPR2341;14-3-3 domain	G3DSA:1.2.19.2 (GENE3D);14-3-3 domain	PTHR1886:SF18 (PANTHER);14-3-3 domain	
comp24971_c4_seq2	1		IPR2349;14-3-3 protein, conserved site				
comp24972_c1_seq1	4		IPR555;JAB1/MPN/MOV34 metalloenzyme domain	IPR24969;Rpn11/EIF3F C-terminal domain	PTHR154:SF7 (PANTHER);Rpn11/EIF3F C-terminal domain	PTHR154 (PANTHER);Rpn11/EIF3F C-terminal domain	
comp25002_c0_seq1	1		PTHR23272:SF3 (PANTHER);HAT dimerisation domain, C-terminal				
comp25136_c0_seq1	3		G3DSA:2.4.1.17 (GENE3D);null	IPR41;ATPase, F1 complex alpha/beta subunit, N-terminal domain	PTHR15184:SF32 (PANTHER);ATPase, F1 complex alpha/beta subunit, N-terminal domain		
comp25247_c1_seq1	1		PTHR24361:SF129 (PANTHER);Protein kinase domain				
comp25315_c0_seq1	1		IPR2736;Actin-related protein 2				
comp25441_c0_seq1	2		IPR27141;U6 snRNA-associated Sm-like protein LSm4/Small nuclear ribonucleoprotein Sm D1/D3	PTHR23338:SF16 (PANTHER);U6 snRNA-associated Sm-like protein LSm4/Small nuclear ribonucleoprot			

			ein Sm D1/D3				
comp25467_c0_seq2	2	IPR125;Bromo adjacent homology (BAH) domain	PTHR1255 (PANTHER); Bromo adjacent homology (BAH) domain				
comp25541_c0_seq1	2	IPR1382;Glycoside hydrolase, family 47	PTHR11742:SF7 (PANTHER); Glycoside hydrolase, family 47				
comp25543_c0_seq1	1	PTHR11588:SF54 (PANTHER);Tubulin					
comp25686_c0_seq1	3	IPR18289;MULE transposase domain	PTHR31669:SF2 (PANTHER); MULE transposase domain	PTHR31669 (PANTHER);MULE transposase domain			
comp25740_c0_seq1	1	PTHR2435:SF49 (PANTHER);Cytochrome P45					
comp25947_c0_seq1	6	IPR1529;DNA-directed RNA polymerase, M/15kDa subunit	IPR1222;Zinc finger, TFIIS-type	G3DSA:2.2.25.1 (GENE3D);Zinc finger, TFIIS-type	PTHR11239 (PANTHER);DNA-directed RNA polymerase, M/15kDa subunit	PTHR11239:SF1 (PANTHER);DNA-directed RNA polymerase, M/15kDa subunit	SSF57783 (SUPERFAMILY);Zinc finger, TFIIS-type
comp25948_c0_seq1	4	PF13489 (PFAM);null	IPR2963;S-adenosyl-L-methionine-dependent methyltransferase	PTHR118:SF724 (PANTHER);S-adenosyl-L-methionine-dependent methyltransferase	PTHR118 (PANTHER);S-adenosyl-L-methionine-dependent methyltransferase		
comp25960_c0_seq1	1	IPR18283;Ribosomal protein S8e, conserved site					
comp26027_c1_seq1	4	IPR2975;Fungal G-protein, alpha subunit	IPR119;Guanine nucleotide binding protein (G-protein), alpha subunit	IPR1125;G protein alpha subunit, helical insertion	PTHR1218:SF176 (PANTHER);Guanine nucleotide binding protein (G-protein), alpha subunit		
comp26032_c0_seq1	1	IPR25213;Kinetochore Sim4 complex subunit Fta2					
comp26044_c3_seq1	3	PF13424 (PFAM);Tetratricopeptide-like helical domain	IPR1326;Tetratricopeptide repeat-containing domain	IPR19734;Tetratricopeptide repeat			

comp26102_c1_seq1	5	IPR1425;Archaeal/bacterial/fungal rhodopsin	IPR2973;Archaeal/bacterial/fungal rhodopsin-like	G3DSA:1.2.17.1 (GENE3D);Archaeal/bacterial/fungal rhodopsin-like	IPR18229; Rhodopsin, retinal binding site	SSF81321 (SUPERFAMILY);Rhodopsin, retinal binding site
comp26106_c0_seq1	5	IPR24948;Major latex protein domain	IPR916;Bet v I domain	IPR23393;START-like domain	PTHR3197 (PANTHER);START-like domain	SSF55961 (SUPERFAMILY);START-like domain
comp26107_c0_seq7	1	IPR2221;Transcription activator, osmotic-stress induced				
comp26108_c0_seq1	3	IPR167;Alcohol dehydrogenase, iron-type	G3DSA:3.4.5.197 (GENE3D);Alcohol dehydrogenase, iron-type	SSF56796 (SUPERFAMILY); Alcohol dehydrogenase, iron-type		
comp26121_c1_seq1	5	G3DSA:3.3.7.27 (GENE3D);Reverse transcriptase domain	IPR13242;Retroviral aspartyl protease	G3DSA:3.1.1.1 (GENE3D);Ribonuclease H-like domain	PTHR24559 (PANTHER);Ribonuclease H-like domain	PTHR24559:SF173 (PANTHER);Ribonuclease H-like domain
comp26123_c0_seq5	1	PTHR1933:SF17 (PANTHER);DDE superfamily endonuclease, CENP-B-like				
comp26131_c0_seq1	1	G3DSA:1.1.42.1 (GENE3D);Haem peroxidase, plant/fungal/bacterial				
comp26139_c0_seq1	1	PTHR11751:SF26 (PANTHER);Pyridoxal phosphate-dependent transferase, major region, subdomain 1				
comp26174_c2_seq1	1	PTHR11695:SF294 (PANTHER);Alcohol dehydrogenase superfamily, zinc-type				
comp26178_c0_seq1	1	IPR1387;Zinc finger C2H2-type/integrase DNA-binding domain				
comp26188_c0_seq1	1	IPR1945;Malate dehydrogenase, type 2				
comp26340_c1_seq1	1	IPR24929;Nucleolar GTP-binding protein 2				
comp26404_c0_seq1	3	IPR54;Ribosomal protein L31e	IPR23621;Ribosomal protein L31e domain	IPR252;Ribosomal protein L31e, conserved site		
comp26441_c0_seq1	3	G3DSA:3.3.7.1 (GENE3D);null	IPR6121;Heavy metal-associated domain, HMA	PTHR22814 (PANTHER);Heavy metal-associated domain, HMA		
comp26467_c0_se	3	IPR941;Enolase	IPR281;Enolase, C-terminal	IPR289;Enolase, conserved site		

q1						
comp26557_c0_seq1	1	IPR4977;Ribosomal protein S25				
comp26577_c0_seq1	2	IPR425;Cytochrome b-c1 complex subunit 8	IPR211;Cytochrome b-c1 complex subunit 8, plants			
comp26583_c0_seq1	3	IPR477;Reverse transcriptase domain	PTHR19446:SF326 (PANTHER);Reverse transcriptase domain	PTHR19446 (PANTHER);Reverse transcriptase domain		
comp26586_c0_seq1	1	PTHR23115:SF124 (PANTHER);Translation elongation factor EFTu/EF1A, C-terminal				
comp26671_c0_seq1	1	PTHR2493:SF257 (PANTHER);P-type ATPase, transmembrane domain				
comp26673_c0_seq1	4	IPR814;TATA-box binding protein	IPR12295;Beta2-adaptin/TBP, C-terminal domain	IPR3491;TATA-box binding protein, conserved site	SSF55945 (SUPERFAMILY);TATA-box binding protein, conserved site	
comp26677_c0_seq1	5	IPR1464;Annexin	IPR1852;Annexin repeat	PTHR152 (PANTHER);Annexin repeat	PTHR152:SF98 (PANTHER);Annexin repeat	SSF47874 (SUPERFAMILY);Annexin repeat
comp26693_c0_seq1	3	IPR15972;Ribosomal protein L19/L19e, domain 1	IPR23638;Ribosomal protein L19/L19e conserved site	IPR27547;Ribosomal protein L19/L19e		
comp26715_c0_seq1	1	PTHR19446:SF244 (PANTHER);null				
comp26717_c0_seq1	1	PTHR1126:SF186 (PANTHER);Glutathione S-transferase, C-terminal-like				
comp26763_c0_seq1	1	IPR29768;Fructose-bisphosphate aldolase class-I active site				
comp26774_c0_seq1	5	IPR16162;Aldehyde dehydrogenase N-terminal domain	IPR1559;Aldehyde dehydrogenase domain	PTHR11699 (PANTHER);Aldehyde dehydrogenase domain	IPR15657;Aminobutyraldehyde dehydrogenase	IPR16161;Aldehyde/histidinol dehydrogenase
comp26881_c0_seq1	2	IPR1921;Ribosomal protein L7A/L8	PTHR2315 (PANTHER);5S ribosomal protein L3e-like			
comp26883_c0_se	4	G3DSA:3.3.3.1 (GENE3D);null	IPR2263;S-adenosylmethi	IPR2133;S-adenosylmethionine	IPR22636;S-	

q1			onine synthetase, C-terminal	synthetase	adenosylmethionine synthetase superfamily
comp26898_c0_seq1	4	IPR6534;P-type ATPase, subfamily IIIA	IPR23299;P-type ATPase, cytoplasmic domain N	PTHR2493:SF271 (PANTHER);P-type ATPase, transmembrane domain	IPR1833;P-type ATPase, phosphorylation site
comp26911_c0_seq1	1	PTHR1164:SF (PANTHER);Profilin			
comp26934_c0_seq1	3	IPR1569;Ribosomal protein L37e	IPR11331;Ribosomal protein L37ae/L37e	IPR18267;Ribosomal protein L37e, conserved site	
comp26937_c0_seq1	4	IPR6652;Kelch repeat type 1	PF13415 (PFAM);Jacalin-like lectin domain	PF13418 (PFAM);Jacalin-like lectin domain	SSF117281 (SUPERFAMILY);Jacalin-like lectin domain
comp26956_c0_seq1	3	IPR5519;Acid phosphatase (Class B)	PTHR31284 (PANTHER); Acid phosphatase (Class B)	PTHR31284:SF6 (PANTHER);Acid phosphatase (Class B)	
comp27009_c0_seq1	2	PTHR23274 (PANTHER);null	PTHR23274:SF9 (PANTHER); null		
comp27046_c0_seq1	2	IPR2935;O-methyltransferase, family 3	PTHR159:SF15 (PANTHER); O-methyltransferase, family 3		
comp27062_c0_seq1	1	PTHR1171:SF191 (PANTHER);Cyclophilin-type peptidyl-prolyl cis-trans isomerase domain			
comp27176_c0_seq1	1	PTHR1342 (PANTHER);Ubiquitin-like			
comp27235_c0_seq1	1	IPR19651;Glutamate dehydrogenase, NAD-specific			
comp27246_c0_seq1	1	PTHR19375:SF164 (PANTHER);Heat shock protein 7kD, peptide-binding domain			
comp27295_c0_seq1	3	IPR19791;Haem peroxidase, animal	PTHR1193 (PANTHER); Haem peroxidase, animal	PTHR1193:SF11 (PANTHER);Haem peroxidase, animal	
comp27296_c0_seq1	3	IPR23441;Ribosomal protein L24e domain	IPR988;Ribosomal protein L24e-related	PTHR1792:SF1 (PANTHER);Ribosomal protein L24e-related	
comp27320_c1_seq1	3	PR1434 (PRINTS);null	IPR1934;NADH dehydrogenase subunit 5, C-terminal	PTHR22773:SF5 (PANTHER);NADH:quinone oxidoreductase/Mrp antiporter,	

			membrane subunit			
comp27367_c0_seq1	2	PF13465 (PFAM);Zinc finger, C2H2-like	PTHR2449 (PANTHER); Zinc finger C2H2-type/integrase DNA-binding domain			
comp27416_c0_seq1	1	PTHR1126:SF39 (PANTHER);Glutathione S-transferase, C-terminal-like				
comp27453_c0_seq1	3	IPR13189;Glycosyl hydrolase family 32, C-terminal	PTHR31953 (PANTHER); Glycosyl hydrolase family 32, C-terminal	PTHR31953:SF1 (PANTHER);Glycosyl hydrolase family 32, C-terminal		
comp27478_c0_seq1	2	IPR2492;Transposase, Tc1-like	PTHR2322:SF19 (PANTHER); Transposase, Tc1-like			
comp27499_c0_seq1	5	G3DSA:3.2.2.21 (GENE3D);null	IPR2629;Cobalamin-independent methionine synthase MetE, C-terminal/archaeal	PTHR3519:SF (PANTHER);Cobalamin-independent methionine synthase MetE, C-terminal/archaeal	PTHR3519 (PANTHER);Cobalamin-independent methionine synthase MetE, C-terminal/archaeal	SSF51726 (SUPERFAMILY);Cobalamin-independent methionine synthase MetE, C-terminal/archaeal
comp27512_c0_seq1	2	IPR14726;Ribosomal protein L2, domain 3	IPR22671;Ribosomal protein L2, conserved site			
comp27513_c0_seq1	2	PF1341 (PFAM);Glutathione S-transferase, C-terminal-like	PTHR1126:SF174 (PANTHER); Glutathione S-transferase, C-terminal-like			
comp27520_c0_seq1	1	PTHR19139:SF5 (PANTHER);Major intrinsic protein				
comp27542_c2_seq1	1	PTHR1933:SF229 (PANTHER);null				
comp27660_c0_seq1	2	IPR2546;ATPase, F1 complex, delta/epsilon subunit, N-terminal	IPR1469;ATPase, F1 complex, delta/epsilon subunit			
comp27666_c0_seq1	1	IPR13215;Cobalamin-independent methionine synthase MetE, N-terminal				
comp27725_c0_seq1	2	IPR4;Actin family	PTHR11937:SF188 (PANTHER); Actin family			

comp27743_c0_seq1	3	IPR43;Adenosylhomocysteinase	G3DSA:3.4.5.148 (GENE3D);Adenosylhomocysteinase	IPR15878;S-adenosyl-L-homocysteine hydrolase, NAD binding domain			
comp27756_c0_seq1	1	IPR445;Glutathione S-transferase, N-terminal					
comp27791_c0_seq1	1	IPR796;Aspartate/other aminotransferase					
comp27801_c0_seq1	3	IPR6114;6-phosphogluconate dehydrogenase, C-terminal	PTHR11811 (PANTHER);6-phosphogluconate dehydrogenase, C-terminal	PTHR11811:SF27 (PANTHER);6-phosphogluconate dehydrogenase, C-terminal			
comp27809_c0_seq1	1	IPR179;Ribosomal protein L1P					
comp27854_c0_seq1	3	IPR938;GOLD	IPR1572;TMP21-related	PTHR22811:SF58 (PANTHER);TMP21-related			
comp27878_c0_seq1	2	IPR652;Triosephosphate isomerase	PTHR21139:SF1 (PANTHER);Aldolase-type TIM barrel				
comp27891_c0_seq1	4	IPR21135;Phosphoenolpyruvate carboxylase	PTHR3523:SF2 (PANTHER);Phosphoenolpyruvate carboxylase	PTHR3523 (PANTHER);Phosphoenolpyruvate carboxylase	IPR15813;Pyruvate/Phosphoenolpyruvate kinase-like domain		
comp27915_c0_seq1	2	IPR35;GIY-YIG nuclease superfamily	IPR635;Intron endonuclease, group I				
comp27915_c1_seq2	2	IPR27387;Cytochrome b/b6-like domain	IPR5798;Cytochrome b/b6, C-terminal				
comp27915_c1_seq9	3	IPR568;ATPase, F complex, subunit A	PTHR1141:SF (PANTHER);ATPase, F complex, subunit A	IPR2311;ATPase, F complex, subunit A, active site			
comp27915_c1_seq12	4	IPR298;Cytochrome c oxidase subunit III domain	IPR13833;Cytochrome c oxidase, subunit III, 4-helical bundle	PTHR1143:SF3 (PANTHER);Cytochrome c oxidase, subunit III, 4-helical bundle	IPR24791;Cytochrome c oxidase subunit III		
comp27924_c0_seq1	3	IPR23798;Ribosomal protein S7 domain	IPR235;Ribosomal protein S5/S7	IPR266;Ribosomal protein S7, conserved site			
comp27937_c0_seq1	6	IPR2942;RNA-binding S4 domain	IPR1912;Ribosomal protein S4/S9, N-terminal	IPR571;Ribosomal protein S4/S9, eukaryotic/archaeal	IPR2281;Ribosomal protein S4/S9	IPR1879;Ribosomal protein S4, conserved site	SSF55174 (SUPERFAMILY);Ribosomal protein S4, conserved site
comp27943_c0_seq1	2	PTHR32183:SF4 (PANTHER);TPMT family	PTHR32183 (PANTHER);TPMT family				
comp27953_c0_seq1	4	IPR2484;Isopropylmalate dehydrogenase-	IPR479;Isocitrate dehydrogenas	PTHR11822:SF5 (PANTHER);Isocitrate dehydrogenase	SSF53659 (SUPERFAMILY);Isoc		

		like domain	e NADP-dependent	NADP-dependent	itrate dehydrogenase NADP-dependent
comp27968_c0_seq1	4	IPR823;Plant peroxidase	PTHR31388:SF7 (PANTHER); Haem peroxidase, plant/fungal/bacterial	PTHR31388 (PANTHER);Haem peroxidase, plant/fungal/bacterial	IPR19793; Peroxidases heam-ligand binding site
comp27984_c0_seq3	3	IPR166;Sterile alpha motif domain	IPR13761;Sterile alpha motif/pointed domain	IPR21129;Sterile alpha motif, type 1	
comp28026_c0_seq1	2	PTHR2411 (PANTHER);RNA recognition motif domain	PTHR2411:SF258 (PANTHER); RNA recognition motif domain		
comp28045_c0_seq1	4	IPR27923;Hydrophobic seed protein	PTHR31731:SF (PANTHER); Hydrophobic seed protein	PTHR31731 (PANTHER);Hydrophobic seed protein	PS51257 (PROSITE_PROFILES);Hydrophobic seed protein
comp28053_c0_seq1	3	IPR27246;Eukaryotic porin/Tom4	PTHR11743 (PANTHER); Eukaryotic porin/Tom4	PTHR11743:SF29 (PANTHER);Eukaryotic porin/Tom4	
comp28054_c0_seq1	3	IPR2784;Ribosomal protein L14	PTHR11127:SF2 (PANTHER); Ribosomal protein L14	PTHR11127 (PANTHER);Ribosomal protein L14	
comp28087_c0_seq1	4	G3DSA:2.4.5.1 (GENE3D);null	IPR293;Glycine cleavage system H-protein	PTHR11715:SF3 (PANTHER);Glycine cleavage system H-protein	IPR1153;Single hybrid motif
comp28093_c0_seq1	1	PTHR11699:SF144 (PANTHER);Aldehyde dehydrogenase N-terminal domain			
comp28111_c0_seq9	1	IPR25332;Protein of unknown function DUF4238			
comp28164_c0_seq1	3	IPR582;Acyl-CoA-binding protein, ACBP	IPR14352;FERM/acyl-CoA-binding protein, 3-helical bundle	PTHR2331 (PANTHER);Acyl-CoA-binding protein, ACBP	
comp28213_c0_seq1	3	IPR227;Plant ascorbate peroxidase	PTHR31356:SF5 (PANTHER); Haem peroxidase, plant/fungal/bacterial	PTHR31356 (PANTHER);Haem peroxidase, plant/fungal/bacterial	
comp28227_c0_seq1	1	PTHR19139:SF52 (PANTHER);Major intrinsic protein			
comp28240_c0_seq1	1	PTHR23413:SF1 (PANTHER);Ribosomal protein L32e			

comp28252_c0_seq1	1	PTHR129 (PANTHER);Isopenicillin N synthase-like			
comp28255_c0_seq1	4	IPR1585;Transaldolase	IPR473;Transaldolase type 1	PTHR1683:SF6 (PANTHER);Transaldolase	IPR18225;Transaldolase, active site
comp28265_c0_seq1	1	PTHR11599:SF44 (PANTHER);Nucleophile aminohydrolases, N-terminal			
comp28340_c0_seq1	1	PTHR2489:SF86 (PANTHER);Mitochondrial substrate/solute carrier			
comp28360_c0_seq1	3	IPR2489;Glutamate synthase, alpha subunit, C-terminal	PTHR11938:SF1 (PANTHER);Glutamate synthase, alpha subunit, C-terminal	PTHR11938 (PANTHER);Glutamate synthase, alpha subunit, C-terminal	
comp28371_c0_seq1	3	IPR194;ATPase, F1/V1/A1 complex, alpha/beta subunit, nucleotide-binding domain	PTHR15184 (PANTHER);ATPase, F1/V1/A1 complex, alpha/beta subunit, nucleotide-binding domain	PTHR15184:SF7 (PANTHER);ATPase, F1/V1/A1 complex, alpha/beta subunit, nucleotide-binding domain	
comp28372_c0_seq1	4	IPR2842;ATPase, V1/A1 complex, subunit E	PTHR11583:SF1 (PANTHER);ATPase, V1/A1 complex, subunit E	PTHR11583 (PANTHER);ATPase, V1/A1 complex, subunit E	SSF16527 (SUPERFAMILY);ATPase, V1/A1 complex, subunit E
comp28396_c0_seq1	3	IPR439;Ribosomal protein L15e	IPR24794;Ribosomal protein L15e core domain	IPR2925;Ribosomal protein L15e, conserved site	
comp28402_c0_seq1	4	IPR241;Cytochrome P45, E-class, group I	PTHR24298:SF61 (PANTHER);Cytochrome P45	PTHR24298 (PANTHER);Cytochrome P45	IPR17972;Cytochrome P45, conserved site
comp28405_c0_seq1	1	PTHR11711:SF118 (PANTHER);P-loop containing nucleoside triphosphate hydrolase			
comp28488_c0_seq1	1	PTHR1886:SF2 (PANTHER);14-3-3 domain			
comp28515_c0_seq1	1	PTHR2456:SF176 (PANTHER);null			
comp28524_c0_seq1	3	IPR24286;Domain of unknown function DUF37	PTHR11772:SF8 (PANTHER);Domain of unknown function DUF37	PTHR11772 (PANTHER);Domain of unknown function DUF37	

comp28561_c0_seq1	1	IPR5874;Eukaryotic translation initiation factor SUI1			
comp28562_c0_seq1	4	IPR1794;ADF/Cofilin/Destrin	IPR218;Actin-depolymerising factor homology domain	IPR296;ADF-H/Gelsolin-like domain	SSF55753 (SUPERFAMILY);Actin-depolymerising factor homology domain
comp28593_c0_seq1	4	IPR1321;Proteinase inhibitor I29, cathepsin propeptide	G3DSA:3.9.7.1 (GENE3D);Proteinase inhibitor I29, cathepsin propeptide	PTHR12411:SF289 (PANTHER);Proteinase inhibitor I29, cathepsin propeptide	IPR13128; Peptidase C1A
comp28679_c0_seq1	1	PTHR24322:SF289 (PANTHER);Short-chain dehydrogenase/reductase SDR			
comp28680_c0_seq1	1	PTHR2493:SF264 (PANTHER);P-type ATPase, cytoplasmic domain N			
comp28845_c0_seq1	4	G3DSA:4.1.26.3 (GENE3D);null	IPR7493;Protein of unknown function DUF538	PTHR31676 (PANTHER);Protein of unknown function DUF538	PTHR31676:SF5 (PANTHER);Protein of unknown function DUF538
comp28874_c0_seq1	3	PF571 (PFAM);null	PTHR1291:SF2 (PANTHER); null	IPR7763;NADH dehydrogenase [ubiquinone] 1 alpha subcomplex subunit 12	
comp28910_c0_seq1	3	PF13967 (PFAM);null	PTHR1318 (PANTHER); null	PTHR1318:SF32 (PANTHER);null	
comp28934_c0_seq1	3	IPR2495;Glycosyl transferase, family 8	PTHR32116:SF9 (PANTHER); Nucleotide-diphospho-sugar transferases	IPR29993;Plant galacturonosyltransferase GAUT	
comp29033_c0_seq1	4	IPR12675;Beta-grasp domain	IPR141;2Fe-2S ferredoxin-type domain	PTHR1937:SF67 (PANTHER);2Fe-2S ferredoxin-type domain	IPR658;2Fe-2S ferredoxin, iron-sulphur binding site
comp29077_c0_seq1	2	PTHR1179:SF58 (PANTHER);null	PTHR1179 (PANTHER); null		
comp29097_c0_seq1	2	PTHR31235:SF11 (PANTHER);Haem peroxidase, plant/fungal/bacterial	PTHR31235 (PANTHER); Haem peroxidase, plant/fungal/bacterial		
comp29099_c0_seq1	4	IPR1576;Phosphoglycerate kinase	IPR1591;Phosphoglycerate kinase, C-	IPR15824;Phosphoglycerate kinase, N-terminal	PTHR1146: SF8 (PANTHE

			terminal		R);Phosphoglycerate kinase		
comp29105_c0_seq1	1	IPR393;Ubiquitin interacting motif					
comp29107_c0_seq1	4	G3DSA:3.8.1.1 (GENE3D);null	PF1458 (PFAM);null	PTHR1552 (PANTHER);null	SSF5258 (SUPERFAMILY);null		
comp29118_c0_seq1	6	IPR1218;NADH dehydrogenase, subunit C	IPR1268;NADH:ubiquinone oxidoreductase, 3kDa subunit	PTHR1884 (PANTHER);NADH:ubiquinone oxidoreductase, 3kDa subunit	PTHR1884:SF6 (PANTHER);NADH:ubiquinone oxidoreductase, 3kDa subunit	IPR2396;NADH:ubiquinone oxidoreductase, 3kDa subunit, conserved site	SSF143243 (SUPERFAMILY);NADH dehydrogenase, subunit C
comp29152_c0_seq1	3	IPR3689;Zinc/iron permease	PTHR114:SF23 (PANTHER);Zinc/iron permease	PTHR114 (PANTHER);Zinc/iron permease			
comp29155_c0_seq1	1	IPR2673;Ribosomal protein L29e					
comp29185_c0_seq1	4	IPR1854;Ribosomal protein L29	G3DSA:1.1.24.1 (GENE3D);Ribosomal protein L29	PTHR13872 (PANTHER);Ribosomal protein L29	IPR18254;Ribosomal protein L29, conserved site		
comp29191_c0_seq1	3	IPR1327;Pyridine nucleotide-disulphide oxidoreductase, NAD-binding domain	PTHR22912 (PANTHER);Pyridine nucleotide-disulphide oxidoreductase, NAD-binding domain	PTHR22912:SF97 (PANTHER);Pyridine nucleotide-disulphide oxidoreductase, NAD-binding domain			
comp29197_c0_seq1	1	PTHR22838 (PANTHER);null					
comp29211_c0_seq1	1	IPR5745;Ribosomal protein L14, bacterial-type					
comp29213_c0_seq1	2	IPR1667;S-adenosylmethionine decarboxylase, core	IPR1985;S-adenosylmethionine decarboxylase				
comp29220_c0_seq1	2	PTHR2377 (PANTHER);ATPase, AAA-type, core	PTHR2377:SF63 (PANTHER);ATPase, AAA-type, core				
comp29231_c0_seq1	2	IPR793;ATPase, F1/V1/A1 complex, alpha/beta subunit, C-terminal	IPR2434;ATPase, F1 complex beta subunit/V1 complex, C-terminal				
comp29251_c0_seq1	1	IPR7232;DNA repair protein Rad52/59/22					
comp29252_c0_seq1	3	IPR8177;Gamma Purothionin	IPR3614;Knottin, scorpion toxin-like	IPR8176;Gamma thionin			

comp29274_c0_seq1	2	IPR298;Rab5-interacting protein family	IPR1742;Rab5-interacting protein					
comp29334_c0_seq1	3	IPR21149;Oligosaccharyl transferase complex, subunit OST3/OST6	IPR6844;Magnesium transporter protein 1	PTHR12692:SF (PANTHER);Magnesium transporter protein 1				
comp29356_c0_seq1	1	PTHR2489:SF92 (PANTHER);Mitochondrial carrier domain						
comp29390_c0_seq1	2	PTHR32494 (PANTHER);null	PTHR32494:SF5 (PANTHER);null					
comp29397_c0_seq1	2	IPR4154;Anticodon-binding	PTHR11451 (PANTHER);Anticodon-binding					
comp29442_c0_seq1	1	PTHR19139:SF3 (PANTHER);Major intrinsic protein						
comp29443_c0_seq1	7	IPR8145;Guanylate kinase/L-type calcium channel beta subunit	IPR17665;Guanylate kinase	G3DSA:3.3.63.1 (GENE3D);Guanylate kinase	PTHR23117:SF13 (PANTHER);P-loop containing nucleoside triphosphate hydrolase	PTHR23117 (PANTHER);P-loop containing nucleoside triphosphate hydrolase	IPR259;Guanylate kinase, conserved site	IPR8144;Guanylate kinase-like
comp29460_c0_seq1	3	IPR645;Cupin 1	PTHR31189:SF1 (PANTHER);RmlC-like jelly roll fold	PTHR31189 (PANTHER);RmlC-like jelly roll fold				
comp29462_c0_seq1	4	G3DSA:1.1.287.99 (GENE3D);null	IPR19831;Manganese/iron superoxide dismutase, N-terminal	IPR1189;Manganese/iron superoxide dismutase	PTHR1144:SF17 (PANTHER);Manganese/iron superoxide dismutase			
comp29470_c0_seq1	1	IPR3666;Photosystem I PsaF, reaction centre subunit III						
comp29483_c0_seq1	3	IPR31;Carbon-nitrogen hydrolase	PTHR2388:SF13 (PANTHER);Carbon-nitrogen hydrolase	PTHR2388 (PANTHER);Carbon-nitrogen hydrolase				
comp29486_c0_seq1	4	IPR185;Adenosine kinase	IPR11611;Carbohydrate kinase PfkB	IPR2956;Ribokinase-like	PTHR1584 (PANTHER);Ribokinase-like			
comp29496_c0_seq1	1	PTHR11588:SF72 (PANTHER);Tubulin						
comp29523_c0_seq1	1	PTHR31189:SF2 (PANTHER);RmlC-like jelly roll fold						
comp29567_c0_seq1	2	IPR22618;Defensin-like protein, Arabidopsis	IPR2191;Low-molecular-weight cysteine-rich family					
comp29581_c0_se	1	G3DSA:3.3.143.1 (GENE3D);null						

q1						
comp29586_c0_seq1	1	PTHR2473:SF394 (PANTHER);P-loop containing nucleoside triphosphate hydrolase				
comp29602_c0_seq1	5	IPR16635;Adaptor protein complex, sigma subunit	G3DSA:3.3.45.6 (GENE3D);Adaptor protein complex, sigma subunit	IPR22775;AP complex, mu/sigma subunit	PTHR11753:SF5 (PANTHER);AP complex, mu/sigma subunit	IPR1112;Longin-like domain
comp29605_c0_seq1	2	IPR163;Ribosomal protein L22/L17	IPR5721;Ribosomal protein L22/L17, eukaryotic/archaeal			
comp29610_c0_seq1	2	IPR27486;Ribosomal protein S1 domain	IPR1848;Ribosomal protein S1			
comp29622_c0_seq1	2	PTHR31265 (PANTHER);null	PTHR31265:SF3 (PANTHER);null			
comp29629_c0_seq1	5	IPR1465;Malate synthase	G3DSA:1.2.122.12 (GENE3D);Malate synthase	PTHR21631 (PANTHER);Malate synthase	PTHR21631:SF16 (PANTHER);Malate synthase	IPR1176;Malate synthase-like
comp29634_c0_seq1	3	IPR559;Formate-tetrahydrofolate ligase, FTHFS	G3DSA:3.3.151.1 (GENE3D);Formate-tetrahydrofolate ligase, FTHFS	PTHR125 (PANTHER);Formate-tetrahydrofolate ligase, FTHFS		
comp29637_c0_seq1	4	IPR6636;Heat shock chaperonin-binding	IPR1536;XPC-binding domain	PTHR1621 (PANTHER);XPC-binding domain	PTHR1621:SF5 (PANTHER);XPC-binding domain	
comp29653_c0_seq1	1	IPR8892;Cold acclimation WCOR413				
comp29701_c0_seq1	5	IPR775;Vesicle transport v-SNARE, N-terminal	G3DSA:1.2.58.4 (GENE3D);Vesicle transport v-SNARE, N-terminal	PTHR2123 (PANTHER);Vesicle transport v-SNARE, N-terminal	PTHR2123:SF26 (PANTHER);Vesicle transport v-SNARE, N-terminal	IPR1989;t-SNARE
comp29707_c0_seq1	5	IPR1865;Ribosomal protein S2	G3DSA:3.4.5.149 (GENE3D);Ribosomal protein S2	IPR577;Ribosomal protein S2, eukaryotic/archaeal	IPR1813;Ribosomal protein S2, conserved site	IPR23591;Ribosomal protein S2, flavodoxin-like domain
comp29736_c0_seq1	1	PTHR31642:SF1 (PANTHER);Transferase				
comp29740_c0_seq1	1	IPR676;Endosulphine				
comp29741_c0_seq1	1	IPR25124;Domain of unknown function DUF45				

comp29744_c0_seq1	3	IPR782;FAS1 domain	PTHR32382 (PANTHER); FAS1 domain	PTHR32382:SF5 (PANTHER);FAS1 domain	
comp29787_c0_seq1	4	IPR8147;Glutamine synthetase, beta-Grasp	PTHR2852:SF5 (PANTHER); Glutamine synthetase, beta-Grasp	PTHR2852 (PANTHER);Glutamine synthetase, beta-Grasp	IPR2732;Glutamine synthetase, N-terminal conserved site
comp29791_c0_seq1	2	IPR136;Glycoside hydrolase, family 1	PTHR1353:SF37 (PANTHER); Glycoside hydrolase, family 1		
comp29809_c0_seq1	2	IPR668;Peptidase C1A, papain C-terminal	PTHR12411:SF296 (PANTHER); Peptidase C1A, papain C-terminal		
comp29814_c0_seq1	4	IPR5846;Alpha-D-phosphohexomutase, alpha/beta/alpha domain III	IPR1655;Alpha-D-phosphohexomutase, alpha/beta/alpha I/II/III	PTHR22573 (PANTHER);Alpha-D-phosphohexomutase, alpha/beta/alpha I/II/III	PTHR22573:SF36 (PANTHER);Alpha-D-phosphohexomutase, alpha/beta/alpha I/II/III
comp29881_c0_seq1	3	IPR8384;Actin-related protein 2/3 complex subunit 4	G3DSA:3.3.146.2 (GENE3D);Actin-related protein 2/3 complex subunit 4	SSF69645 (SUPERFAMILY); Actin-related protein 2/3 complex subunit 4	
comp29910_c0_seq1	2	IPR11344;Single-stranded DNA-binding protein	IPR424;Primosome PriB/single-strand DNA-binding		
comp29943_c0_seq1	1	PTHR1124 (PANTHER);WD 4 repeat			
comp29948_c0_seq1	3	IPR18859;BAR domain-containing family	PTHR1661:SF118 (PANTHER); BAR domain-containing family	PTHR1661 (PANTHER);BAR domain-containing family	
comp30035_c0_seq1	1	IPR2829;LAMTOR1/MEH1			
comp30324_c0_seq1	1	IPR18793;Cytochrome c oxidase assembly protein PET191			
comp30359_c0_seq1	4	IPR1915;Peptidase M48	G3DSA:3.3.21.1 (GENE3D);Peptidase M48	IPR2757;CAAX prenyl protease 1	PTHR112 (PANTHER);CAAX prenyl protease 1
comp30384_c0_seq1	1	PTHR118:SF711 (PANTHER);S-adenosyl-L-methionine-dependent methyltransferase			

comp30450_c0_seq1	4	G3DSA:3.4.5.8 (GENE3D);null	IPR13112;FAD-binding 8	IPR17927;Ferredoxin reductase-type FAD-binding domain	SSF52343 (SUPERFAMILY);Ferredoxin reductase-type FAD-binding domain
comp30590_c0_seq1	1	IPR13958;DASH complex subunit Dad1			
comp30637_c0_seq1	1	IPR13869;Protein of unknown function DUF1757			
comp30660_c0_seq1	1	IPR9571;Actin cortical patch SUR7/pH-response regulator PalI			
comp30774_c0_seq1	2	IPR2674;Ribosomal protein L37ae	PTHR11517 (PANTHER); Ribosomal protein L37ae		
comp30809_c0_seq1	1	PTHR11695:SF44 (PANTHER);Alcohol dehydrogenase superfamily, zinc-type			
comp30812_c0_seq1	1	IPR9653;Protein of unknown function DUF1242			
comp30844_c0_seq1	2	IPR17384;NADH dehydrogenase [ubiquinone] (complex I), alpha subcomplex subunit 1	PTHR1798:SF2 (PANTHER); NADH dehydrogenase [ubiquinone] (complex I), alpha subcomplex subunit 1		
comp30847_c0_seq1	1	IPR6413;P-type ATPase, subfamily IIA, PMR1-type			
comp30992_c0_seq1	3	IPR2579;Peptide methionine sulphoxide reductase MrsB	PTHR1173:SF25 (PANTHER); Peptide methionine sulphoxide reductase MrsB	IPR28427;Peptide methionine sulfoxide reductase	
comp31045_c0_seq1	2	IPR16482;Protein transport protein SecG/Sec61-beta/Sbh	IPR3671;Protein transport Sec61-beta/Sbh		
comp31052_c0_seq1	1	IPR6913;Glutathione-dependent formaldehyde-activating enzyme/centromere protein V			
comp31135_c0_seq1	1	PTHR11695:SF384 (PANTHER);Alcohol dehydrogenase superfamily, zinc-type			

comp31196_c0_seq1	2	IPR4923;Iron permease FTR1/Fip1/EfeU	PTHR31632:SF2 (PANTHER);Iron permease FTR1/Fip1/EfeU			
comp31221_c0_seq1	1	IPR2699;ATPase, V1 complex, subunit D				
comp31253_c0_seq1	5	IPR938;CAP Gly-rich domain	IPR626;Ubiquitin-like	PTHR18916 (PANTHER);CAP Gly-rich domain	PTHR18916:SF33 (PANTHER);CAP Gly-rich domain	IPR2971;Ubiquitin-related domain
comp31347_c0_seq1	2	IPR1117;Protein phosphatase inhibitor	PTHR2835:SF (PANTHER); Protein phosphatase inhibitor			
comp31378_c0_seq1	3	IPR2843;Polyketide synthase, enoylreductase domain	IPR13149;Alcohol dehydrogenase, C-terminal	PTHR11695:SF411 (PANTHER);Alcohol dehydrogenase superfamily, zinc-type		
comp31451_c0_seq1	2	IPR243;Peptidase T1A, proteasome beta-subunit	PTHR11599:SF3 (PANTHER); Proteasome, subunit alpha/beta			
comp31471_c0_seq1	2	PTHR22779:SF6 (PANTHER);null	PTHR22779 (PANTHER); null			
comp31543_c0_seq1	2	PTHR24115:SF382 (PANTHER);Kinesin motor domain	IPR19821;Kinesin motor domain, conserved site			
comp31573_c0_seq1	3	IPR811;Complex 1 LYR protein	PTHR13166:SF6 (PANTHER); Complex 1 LYR protein	PTHR13166 (PANTHER);Complex 1 LYR protein		
comp31643_c0_seq1	5	IPR11765;Peptidase M16, N-terminal	IPR11237;Peptidase M16 domain	PTHR11851 (PANTHER);Peptidase M16 domain	PTHR11851:SF68 (PANTHER);Peptidase M16 domain	IPR11249;Metalloenzyme, LuxS/M16 peptidase-like
comp31690_c0_seq1	1	PTHR1222:SF (PANTHER);Cytochrome b-c1 complex subunit 7				
comp31694_c0_seq1	1	PTHR21535:SF31 (PANTHER);Mg2+ transporter protein, CorA-like/Zinc transport protein ZntB				
comp31708_c0_seq1	5	IPR2499;Cysteinyl-tRNA synthetase/mycothiol ligase	IPR1583;Cysteine-tRNA ligase	G3DSA:1.2.12.64 (GENE3D);Cysteinyl-tRNA synthetase/mycothiol ligase	PTHR189: SF3 (PANTHER);Rossmann-like alpha/beta/alpha sandwich fold	IPR98;Aminoacyl-tRNA synthetase, class 1a, anticodon-binding

comp31726_c0_seq1	1	PTHR23249:SF15 (PANTHER);Sybindin-like protein					
comp31750_c0_seq1	6	IPR73;Proliferating cell nuclear antigen, PCNA	IPR22648;Proliferating cell nuclear antigen, PCNA, N-terminal	IPR22649;Proliferating cell nuclear antigen, PCNA, C-terminal	G3DSA:3.7.1.1 (GENE3D);Proliferating cell nuclear antigen, PCNA, C-terminal	IPR22659;Proliferating cell nuclear antigen, PCNA, conserved site	SSF55979 (SUPERFAMILY);Proliferating cell nuclear antigen, PCNA, conserved site
comp31769_c0_seq1	3	IPR1461;Aspartic peptidase	IPR2119;Aspartic peptidase domain	PTHR13683:SF252 (PANTHER);Aspartic peptidase			
comp31789_c0_seq1	1	IPR11516;Shugoshin, N-terminal					
comp31799_c0_seq1	6	IPR1828;Catalase, mono-functional, haem-containing	IPR11614;Catalase core domain	PTHR11465:SF7 (PANTHER);Catalase core domain	IPR2478;Catalase active site	IPR2226;Catalase haem-binding site	IPR2835;Catalase-like domain
comp31813_c0_seq1	2	PTHR12914:SF2 (PANTHER);GINS complex	IPR5339;GINS complex, subunit Psf1				
comp31849_c0_seq1	2	IPR7262;Vacuolar protein sorting 55	PTHR125:SF (PANTHER);Vacuolar protein sorting 55				
comp31885_c0_seq1	1	PF13857 (PFAM);Ankyrin repeat-containing domain					
comp31929_c0_seq1	1	IPR21858;Protein of unknown function DUF3468					
comp31963_c0_seq1	3	IPR757;Glycoside hydrolase, family 16	PTHR1963 (PANTHER);Concanavalin A-like lectin/glucanase domain	PTHR1963:SF3 (PANTHER);Concanavalin A-like lectin/glucanase domain			
comp32009_c0_seq1	2	PTHR15481 (PANTHER);RNA recognition motif domain	PTHR15481:SF (PANTHER);RNA recognition motif domain				
comp32049_c0_seq1	3	IPR6439;HAD hydrolase, subfamily IA	PTHR1891:SF1 (PANTHER);HAD hydrolase, subfamily IA	PTHR1891 (PANTHER);HAD hydrolase, subfamily IA			
comp32061_c0_seq1	1	IPR1845;Reactive oxygen species modulator 1					
comp32062_c0_seq1	6	IPR364;Zinc finger, U1-type	IPR1385;Zinc finger, U1-C type	IPR1734;U1 small nuclear ribonucleoprotein C	PTHR31148:SF1 (PANTHER);U1 small nuclear ribonucleoprotein C	IPR69;Zinc finger, C2H2-type matrin	SSF57667 (SUPERFAMILY);Zinc finger, C2H2-type matrin

comp32089_c0_seq1	1	PF13637 (PFAM);null				
comp32176_c0_seq1	2	IPR531;Mob1/phocein	PTHR22599:SF2 (PANTHER); Mob1/phocein			
comp32279_c0_seq1	5	IPR1588;Zinc finger, C2H2-like	IPR22755;Zinc finger, double-stranded RNA binding	PF12874 (PFAM);Zinc finger, double-stranded RNA binding	PF13912 (PFAM);Zinc finger, double-stranded RNA binding	IPR787;Zinc finger, C2H2
comp32420_c0_seq1	1	IPR18811;Uncharacterised protein family FLILHELTA				
comp32448_c0_seq1	2	PTHR22847:SF346 (PANTHER);WD4 repeat	SSF63829 (SUPERFAMILY);WD4-repeat-containing domain			
comp32520_c0_seq1	1	PTHR243:SF444 (PANTHER);null				
comp32545_c0_seq1	1	IPR1785;Alkaline-phosphatase-like, core domain				
comp32553_c0_seq1	2	PTHR1261:SF (PANTHER);Clathrin/coatomer adaptor, adaptin-like, N-terminal	IPR1716;Coatomer gamma subunit			
comp32591_c0_seq1	3	IPR3123;VPS9 domain	PTHR2311 (PANTHER); VPS9 domain	SSF19993 (SUPERFAMILY); VPS9 domain		
comp32643_c0_seq1	1	IPR2838;Mitochondrial biogenesis protein AIM24				
comp32681_c0_seq1	3	IPR2741;TCP-1-like chaperonin intermediate domain	PTHR11353:SF58 (PANTHER); TCP-1-like chaperonin intermediate domain	SSF54849 (SUPERFAMILY); Chaperonin TCP-1, conserved site		
comp32822_c0_seq1	3	IPR12292;Globin, structural domain	IPR971;Globin	IPR95;Globin-like		
comp32853_c0_seq1	1	PTHR23115:SF96 (PANTHER);Elongation factor, GTP-binding domain				
comp32899_c0_seq1	2	IPR58;Zinc finger, AN1-type	SSF11831 (SUPERFAMILY);Protein kinase-like domain			
comp32995_c0_seq1	1	PTHR24361:SF1 (PANTHER);Protein kinase domain				
comp33177_c0_seq1	3	IPR5123;Oxoglutarate/iron-dependent dioxygenase	PTHR1869 (PANTHER); Oxoglutarate/iron-dependent dioxygenase	PTHR1869:SF47 (PANTHER);Oxoglutarate/iron-dependent dioxygenase		

comp33213_c0_seq1	1	PTHR22883:SF4 (PANTHER);null					
comp33333_c0_seq1	1	PTHR24423:SF395 (PANTHER);Signal transduction response regulator, receiver domain					
comp33478_c0_seq1	1	PTHR11695:SF388 (PANTHER);Alcohol dehydrogenase superfamily, zinc-type					
comp33584_c0_seq1	6	IPR222;Hydroxymethylglutaryl-CoA reductase, class I/II	IPR2374;Hydroxymethylglutaryl-CoA reductase, class I/II, catalytic domain	IPR923;Hydroxymethylglutaryl-CoA reductase, class I/II, NAD/NADP-binding domain	PTHR1572:SF2 (PANTHER);Hydroxymethylglutaryl-CoA reductase, class I/II, NAD/NADP-binding domain	IPR2376;Hydroxymethylglutaryl-CoA reductase, class I/II, conserved site	IPR929; Hydroxymethylglutaryl-CoA reductase, class I/II, substrate-binding domain
comp33605_c0_seq1	4	IPR13342;Mandelate racemase/muconate lactonizing enzyme, C-terminal	IPR2965;Enolase C-terminal domain-like	IPR1354;Mandelate racemase/muconate lactonizing enzyme/methylaspartate ammonia-lyase	PTHR13794:SF5 (PANTHER);Mandelate racemase/muconate lactonizing enzyme/methylaspartate ammonia-lyase		
comp33713_c0_seq1	3	IPR24956;tRNAHis guanylyltransferase catalytic domain	PTHR12729:SF1 (PANTHER);tRNAHis guanylyltransferase catalytic domain	IPR7537;tRNAHis guanylyltransferase Thg1			
comp33719_c0_seq1	1	IPR9453;IMP-specific 5-nucleotidase					
comp33755_c0_seq1	2	PTHR31642 (PANTHER);Chloramphenicol acetyltransferase-like domain	PTHR31642:SF13 (PANTHER); Chloramphenicol acetyltransferase-like domain				
comp33773_c0_seq1	1	PF12774 (PFAM);P-loop containing nucleoside triphosphate hydrolase					
comp33918_c0_seq1	1	PTHR11851:SF85 (PANTHER);Peptidase M16 domain					
comp33949_c0_seq1	1	PTHR24322:SF78 (PANTHER);NAD(P)-binding domain					

comp34032_c0_seq1	2	IPR6935;Helicase/UvrB domain	PTHR2431:SF164 (PANTHER);P-loop containing nucleoside triphosphate hydrolase	
comp34108_c0_seq1	3	IPR7128;Nnf1	PTHR15459:SF2 (PANTHER);Nnf1	PTHR15459 (PANTHER);Nnf1
comp34147_c0_seq1	1	PTHR24347:SF141 (PANTHER);Calcium/calmodulin-dependent/calcium-dependent protein kinase		
comp34191_c0_seq1	2	PTHR31263 (PANTHER);Zn(2)-C6 fungal-type DNA-binding domain	PTHR31263:SF1 (PANTHER);Zn(2)-C6 fungal-type DNA-binding domain	
comp34494_c0_seq1	1	PTHR24115:SF36 (PANTHER);Kinesin motor domain		
comp34527_c0_seq1	3	IPR18556;Domain of unknown function DUF213	PTHR13357 (PANTHER);Domain of unknown function DUF213	IPR3125;Protein Ldb17
comp34533_c0_seq1	1	PTHR2463:SF251 (PANTHER);General substrate transporter		
comp34560_c0_seq1	2	PTHR2298:SF58 (PANTHER);null	PTHR2298 (PANTHER);null	
comp34650_c0_seq1	2	IPR13748;Replication factor C, C-terminal domain	PTHR11669:SF1 (PANTHER);Replication factor C, C-terminal domain	
comp34701_c0_seq1	1	PTHR24322:SF313 (PANTHER);NAD(P)-binding domain		
comp34768_c0_seq1	2	IPR7225;Exocyst complex subunit Sec15-like	PTHR1272:SF (PANTHER);Exocyst complex subunit Sec15-like	
comp34987_c0_seq1	1	PTHR31263:SF4 (PANTHER);Zn(2)-C6 fungal-type DNA-binding domain		
comp35000_c0_seq1	1	IPR12885;F-box associated domain, type 2		

comp35093_c0_seq1	3	IPR2737;RING-type zinc-finger, LisH dimerisation motif	IPR27711;Rmd5	PTHR1217 (PANTHER);Rmd5
comp35235_c0_seq1	1	PTHR2456:SF92 (PANTHER);Protein kinase domain		
comp35363_c0_seq1	3	IPR4299;Membrane bound O-acyl transferase, MBOAT	IPR14371;Sterol O-acyltransferase, ACAT/DAG/ARE types	PTHR148:SF9 (PANTHER);Sterol O-acyltransferase, ACAT/DAG/ARE types
comp35445_c0_seq1	1	IPR1533;Transcriptional coactivator/pterin dehydratase		
comp35447_c0_seq1	2	PTHR32514:SF12 (PANTHER);null	PTHR32514 (PANTHER);null	
comp35520_c0_seq1	1	IPR19433;GPI-Mannosyltransferase II co-activator, Pga1		
comp35557_c0_seq1	3	IPR1394;Alpha/beta hydrolase fold-3	PTHR2324:SF24 (PANTHER);Alpha/beta hydrolase fold-3	PTHR2324 (PANTHER);Alpha/beta hydrolase fold-3
comp35568_c0_seq1	3	IPR13786;Acyl-CoA dehydrogenase/oxidase, N-terminal	PTHR199 (PANTHER);Acyl-CoA dehydrogenase/oxidase, N-terminal	PTHR199:SF241 (PANTHER);Acyl-CoA dehydrogenase/oxidase, N-terminal
comp35617_c0_seq1	1	PTHR2473:SF45 (PANTHER);P-loop containing nucleoside triphosphate hydrolase		
comp35681_c0_seq1	3	IPR374;Phosphatidate cytidylyltransferase	PTHR13773:SF8 (PANTHER);Phosphatidate cytidylyltransferase	PTHR13773 (PANTHER);Phosphatidate cytidylyltransferase
comp35741_c0_seq1	1	PTHR22844:SF126 (PANTHER);F-box domain		
comp35757_c0_seq1	1	PTHR2278:SF4 (PANTHER);Armadillo-like helical		
comp35819_c0_seq1	1	IPR756;Domain of unknown function DUF521		
comp35869_c0_seq1	1	PTHR11353:SF54 (PANTHER);Chaperonin Cpn6/TCP-1 family		
comp35963_c0_seq1	3	IPR519;Anp1	PTHR24319:SF25 (PANTHER);Anp1	PTHR24319 (PANTHER);Anp1
comp36015_c0_seq1	1	IPR1285;DNA helicase Pif1 like		

comp36049_c0_seq1	5	G3DSA:3.1.45.5 (GENE3D);null	IPR9783;Protein of unknown function DUF1348	PTHR31757 (PANTHER);Protein of unknown function DUF1348	PTHR31757:SF (PANTHER);Protein of unknown function DUF1348	SSF54427 (SUPERFAMILY);Protein of unknown function DUF1348
comp36080_c0_seq1	1	IPR27244;Vacuolar membrane-associated protein Iml1				
comp36097_c0_seq1	2	PTHR31987 (PANTHER);null	PTHR31987:SF8 (PANTHER);null			
comp36440_c0_seq1	2	IPR7259;Gamma-tubulin complex component protein	IPR15697;Gamma-tubulin complex component/Spindle pole body component			
comp36568_c0_seq1	4	G3DSA:1.1.8.27 (GENE3D);null	IPR195;Rab-GTPase-TBC domain	PTHR22957 (PANTHER);Rab-GTPase-TBC domain	PTHR22957:SF26 (PANTHER);Rab-GTPase-TBC domain	
comp36580_c0_seq1	1	PTHR12526:SF311 (PANTHER);Zn(2)-C6 fungal-type DNA-binding domain				
comp36600_c0_seq1	1	PTHR11785:SF263 (PANTHER);Amino acid/polyamine transporter I				
comp36603_c0_seq1	2	IPR2459;AMP-binding	PTHR2496 (PANTHER);AMP-dependent synthetase/ligase			
comp36637_c0_seq1	2	PTHR31986:SF7 (PANTHER);null	PTHR31986 (PANTHER);null			
comp36751_c0_seq1	4	IPR722;RNA polymerase, alpha subunit	G3DSA:2.4.4.2 (GENE3D);RNA polymerase, alpha subunit	PTHR19376 (PANTHER);RNA polymerase, alpha subunit	IPR157;DNA-directed RNA polymerase III subunit RPC1	
comp36761_c0_seq1	1	PTHR24322:SF228 (PANTHER);Short-chain dehydrogenase/reductase SDR				
comp36762_c0_seq1	3	IPR15797;NUDIX hydrolase domain-like	PTHR13622:SF5 (PANTHER);NUDIX hydrolase domain-like	PTHR13622 (PANTHER);NUDIX hydrolase domain-like		
comp36787_c0_seq1	5	IPR2478;PUA domain	IPR5155;Ribosome biogenesis	IPR15947;PUA-like domain	PTHR23415 (PANTHE	PTHR23415:SF4 (PANTHER)

			factor NIP7-like		R);PUA-like domain	;PUA-like domain
comp36793_c0_seq1	1	IPR12722;T-complex protein 1, zeta subunit				
comp36922_c0_seq1	1	PTHR11662:SF21 (PANTHER);General substrate transporter				
comp37014_c0_seq1	1	PTHR24356:SF141 (PANTHER);null				
comp37214_c0_seq1	1	IPR211;N-glycosylation protein EOS1				
comp37420_c0_seq1	1	IPR25496;Protein of unknown function DUF4387				
comp37485_c0_seq1	1	IPR3395;RecF/RecN/SMC, N-terminal				
comp37549_c0_seq1	1	PTHR2456:SF39 (PANTHER);Protein kinase domain				
comp37650_c0_seq1	1	G3DSA:3.9.17.1 (GENE3D);Adenylosuccinate synthetase				
comp37719_c0_seq1	3	IPR4274;NLI interacting factor	PTHR1221 (PANTHER);NLI interacting factor	PTHR1221:SF51 (PANTHER);NLI interacting factor		
comp37978_c0_seq1	2	IPR2772;Glycoside hydrolase family 3 C-terminal domain	PTHR362:SF14 (PANTHER);Glycoside hydrolase family 3 C-terminal domain			

Supplementary Table 4 List of Differentially Expressed Genes (DEGs).

Transcript ID	Wormbase Gene ID	Gene Name/Transcript Name	Chromosome	qstart	qend	sstart	send	B1	B2	B3	B4	B5	B6	B7	P-Value	FDR
TCONS_00018298	WBGene00000137	amx-1	III	28	2502	1	2475	30.56441048	53.89648382	27.37787198	34.12991325	26.09444952	37.92088881	8.479869648	0.001111343	0.03593279
TCONS_00015999	WBGene00000389	cdc-25.4	II	1	837	1	837	16.52914139	16.29490347	9.906294263	7.071344663	23.36298663	0.7485821	30.01106254	8.10E-06	0.000365092
TCONS_00012637	WBGene00000399	cdh-7	II	111	4205	1	4095	1.886945886	4.424261746	1.392843131	0.653369962	0.589319529	1.09192241	0.3072389	0.000941467	0.031339626
TCONS_00014062	WBGene00000606	col-17	II	1	887	4867574	4866688	10.22865746	10.62395825	2.829809105	4.528851023	2.662867472	6.07347747	2.31986308	0.000141073	0.00553588
TCONS_00042184	WBGene00000702	col-128	IV	24	1307	1	1284	0.737104484	1.671023897	0.374600601	0.228789038	0.184559475	0.348660939	0.140166772	0.000770881	0.026363351
TCONS_00016025	WBGene00001531	gcy-4	II	1	3411	1	3411	0.091475771	35.42915058	0.203337254	0.160301303	0.103209328	0.17067007	0.089980403	1.41E-08	8.39E-07
TCONS_00016028	WBGene00001532	gcy-5	II	1	3369	1	3369	0.136788165	35.40380346	0.260943789	0.307838093	0.318387887	0.348583274	0.325939248	0.000141611	0.005551683
TCONS_00001805	WBGene00001614	glr-3	I	1	2511	1	2511	0.038312112	0.118057998	0.145511844	8.36719237	0.065276796	0.175524714	0.126150095	1.85E-05	0.000802331
TCONS_00030051	WBGene00001796	gtl-2	IV	357	4565	1	4221	1.361645056	2.438120424	1.702019286	1.469099302	1.377443523	104.4434778	0.886846684	3.24E-05	0.00135928
TCONS_00030049	WBGene00001796	gtl-2	IV	106	4322	1	4221	1.368645338	2.429692162	1.713229341	1.475481559	1.363580438	108.4077851	0.893855023	3.10E-05	0.00130636
TCONS_00030050	WBGene00001796	gtl-2	IV	106	3954	1	3849	1.368645338	2.438120424	1.706347682	1.475481559	1.363580438	104.4434778	0.893855023	3.00E-05	0.001267159
TCONS_00030052	WBGene00001796	gtl-2	IV	13	4233	1	4221	1.361645056	2.438120424	1.702019286	1.469099302	1.363580438	104.4434778	0.879838345	3.00E-05	0.001268688
TCONS_00009644	WBGene00001806	gur-5	II	14	1240	1	1227	1.595096254	0.025284787	0.453411368	0.0659121	0.042653807	0.006607179	0.086459066	5.11E-05	0.002112583
TCONS_00017001	WBGene00001884	his-10	II	1	312	1	312	6.031251611	2.846198033	5.845678495	5.964126086	47.66590455	12.89502713	18.96724748	4.94E-05	0.0020475
TCONS_00012398	WBGene00001887	his-13	II	1	411	1	411	4.284894519	1.926582563	2.493665568	3.995740277	24.13075566	4.412871692	7.798756053	0.000668527	0.023179264
TCONS_00016999	WBGene00001888	his-14	II	1	312	1	312	6.037022033	2.850996376	5.851283523	6.534236472	51.7336554	13.37255957	18.91463343	6.55E-05	0.002682138
TCONS_00012402	WBGene00001900	his-26	II	1	312	1	312	5.965317248	2.825711589	5.845678496	5.939455604	23.88195058	12.89502713	19.09585958	0.000725271	0.024962411
TCONS_00041355	WBGene00002013	hsp-12.6	IV	32	364	1	333	13.95162912	1.745102333	5.325967183	8.801957129	18.49558392	3.814424507	51.20102941	2.74E-08	1.58E-06
TCONS_00041354	WBGene00002013	hsp-12.6	IV	231	657	9446639	9446213	14.0950387	1.789705141	5.388899119	8.943484104	18.60313262	3.866723647	51.43586025	3.44E-08	1.98E-06
TCONS_00049727	WBGene00002018	hsp-16.41	V	39	470	1	432	145.6460985	4.066037303	7.581789833	28.39938233	42.95668601	32.35748313	170.7619064	0.000357275	0.013026451
TCONS_00006504	WBGene00002026	hsp-70	I	31	1962	1	1932	125.2180176	3.295419761	5.437812245	21.72910609	33.26396031	24.76152487	111.1406019	0.001190635	0.038290064
TCONS_00025336	WBGene00002140	inx-18	IV	84	1322	1	1239	3.445933802	17.73591853	2.584318878	0.908287792	2.944817084	2.867830749	0.484806368	0.000902999	0.030418199
TCONS_00025337	WBGene00002140	inx-18	IV	84	1394	1	1311	3.445933802	17.73591853	2.584318878	0.908287792	2.944817084	2.867830749	0.47779803	0.000853003	0.028947246
TCONS_00025335	WBGene00002140	inx-18	IV	84	1322	1	1239	3.434392957	15.27125108	2.559345505	0.810091967	2.867887469	2.8341589	0.426380656	0.001356673	0.043291529
TCONS_00041506	WBGene00002206	kin-24	IV	97	1647	1	1551	5.334657073	9.855506327	2.825322587	3.108430006	4.567556847	27.32835052	3.555689676	0.000222233	0.0084391
TCONS_00063699	WBGene00002233	kqt-1	X	170	2248	1	2079	93.90383588	77.14050436	239.8301437	88.67615492	227.4640767	69.02489033	182.0471772	2.95E-05	0.001249717
TCONS_00063697	WBGene00002233	kqt-1	X	1	2223	1	2223	105.7428539	90.57158702	240.30803	100.5976108	227.5464914	87.62403906	190.3920585	0.000397359	0.014360746
TCONS_00063698	WBGene00002233	kqt-1	X	1	1604	1	1604	105.9566451	90.85956568	240.6267992	100.9730781	227.8597761	87.97986546	190.6796064	0.000411219	0.014809651
TCONS_00018291	WBGene00003033	lin-48	III	1	843	1	843	6.011456867	28.0472025	24.42126111	16.35025604	15.02143968	22.9886849	0.400706305	0.000282915	0.010529749
TCONS_00042097	WBGene00003287	mir-59	IV	1	100	11309253	11309154	0	0	0	0	0	3.97752169	0	3.00E-20	4.65E-18
TCONS_00025255	WBGene00003362	mir-269	IV	1	94	273900	273993	0	0.783781823	0	0	0	0	0	1.38E-12	1.16E-10
TCONS_00062692	WBGene00003364	mir-271	X	1	94	104	11	0.005770422	0.134852198	0.357846271	0.038665983	0.318850952	0	1.458833165	3.91E-13	3.43E-11

TCONS_00026921	WBGene00003445	msp-52	IV	1	234	5265592	5265825	1.22229206	10.05449651	0.575883094	7.154292632	0.502102392	0.30543513	0.184610142	2.11E-13	1.90E-11
TCONS_00026760	WBGene00003446	msp-53	IV	1	463	5064637	5065099	16.19933656	22.56482808	11.4322857	18.13980155	7.859585902	6.120349036	2.726793081	0.001363876	0.043420416
TCONS_00038797	WBGene00003446	msp-53	IV	1	513	5065153	5064641	16.19933656	22.56482808	11.4322857	18.13980155	7.859585902	6.120349036	2.726793081	0.001363876	0.043420416
TCONS_00018244	WBGene00003605	nhr-6	III	4	1825	39	1860	0.980884484	15.05252105	13.76768642	11.7265033	1.2375858	11.93736115	8.241364899	0.001298976	0.041547048
TCONS_00018243	WBGene00003605	nhr-6	III	1	1858	2	1860	1.492371219	17.14330679	14.97482212	13.35306949	1.254723704	13.83255329	8.883807378	0.000499008	0.01775383
TCONS_00070391	WBGene00003616	nhr-17	X	3	1193	1	1191	36.41425395	3.191632143	3.1223283	8.26743882	14.33770409	3.978812697	49.70106282	0.000368758	0.013374042
TCONS_00070389	WBGene00003616	nhr-17	X	1	2967	12670734	12667768	38.22891783	3.970303999	3.786834079	9.691893784	16.50865163	5.389928243	51.89108786	0.000929208	0.031122799
TCONS_00049439	WBGene00003689	nhr-99	V	1	1257	1	1257	1.206216592	1.497535657	0.79509918	1.037163915	0.419305787	0.974109771	0.345767594	0.000766056	0.026256398
TCONS_00005071	WBGene00003906	paf-1	I	80	1234	1	1155	0.763968985	1.17117027	1.130403835	1.031785322	0.862079534	9.919689517	0.307307571	0.000445938	0.016004007
TCONS_00042598	WBGene00003995	pgp-1	IV	3	3968	1	3966	1.977191798	3.486745976	1.652571041	4.81684821	1.715943389	3.470074564	1.951226501	0.001359023	0.043332932
TCONS_00067259	WBGene00004351	rgs-8.1	X	1	690	1	690	1.790637972	8.045985222	1.74374699	4.984295944	1.754997734	5.411871538	5.661492543	0.00089439	0.030227158
TCONS_00026918	WBGene00004844	sls-2.12	IV	1	110	1	110	0.453782512	9.521661987	0.176588747	6.805699025	0.122175593	0.072756632	0.028033354	5.71E-25	1.43E-22
TCONS_00026920	WBGene00004851	sls-2.19	IV	1	115	5264907	5265021	0.432781667	9.521661987	0.169707088	6.805699025	0.122175593	0.078169566	0.028033354	2.79E-25	7.18E-23
TCONS_00038858	WBGene00004929	soc-2	IV	196	1872	1	1677	10.94021459	80.07199935	11.79365728	32.72882358	12.32623929	10.04410042	8.694317703	8.27E-05	0.003364599
TCONS_00038856	WBGene00004929	soc-2	IV	291	1970	1	1680	10.37093634	74.87092042	14.16874177	28.98428384	14.64703133	11.86256488	10.35126266	0.001117963	0.036093986
TCONS_00038855	WBGene00004929	soc-2	IV	88	1767	1	1680	10.27785184	74.80103282	14.04437341	28.74739546	14.52119902	11.73758677	10.22624056	0.001075762	0.034923253
TCONS_00038857	WBGene00004929	soc-2	IV	218	1897	1	1680	10.32893465	82.24921761	14.06374175	29.40390936	14.53833692	11.74841264	10.2332489	0.000686103	0.023713482
TCONS_00014598	WBGene00004996	spp-11	II	5	1189	1	1185	1.792153472	0.2663861	1.852576903	2.03681063	3.816510953	0.729163526	1.499784459	4.35E-05	0.001818128
TCONS_00014596	WBGene00004996	spp-11	II	289	1210	264	1185	1.896778823	0.286872545	1.986878692	2.159676937	3.970752088	0.752009506	1.569867844	4.32E-05	0.00180445
TCONS_00014593	WBGene00004996	spp-11	II	238	734	6405754	6405258	1.148605604	0.112091232	1.093721904	1.098183525	2.271889341	0.327009202	0.89706734	2.41E-06	0.000111257
TCONS_00014595	WBGene00004996	spp-11	II	430	1068	6406287	6405649	1.567579115	0.220552544	1.535862464	1.72945864	2.928996677	0.557221546	1.296542639	4.64E-05	0.001925667
TCONS_00014594	WBGene00004996	spp-11	II	289	1873	6406842	6405258	1.774370096	0.268785272	1.872224876	2.046627066	3.509857046	0.750179307	1.513801136	8.23E-05	0.003350353
TCONS_00014597	WBGene00004996	spp-11	II	374	1466	6406741	6405649	1.689515732	0.248298827	1.737923087	1.923760759	3.370925453	0.73274626	1.450726088	8.39E-05	0.003403548
TCONS_00011417	WBGene00005071	srb-6	II	1	1014	1	1014	18.0152582	17.6579143	10.33114537	7.326830801	26.44833911	0.021651736	34.43094608	7.28E-07	3.73E-05
TCONS_00019503	WBGene00005072	srb-7	III	1	1047	1	1047	0.052312675	0.048232728	0.062931936	18.94804852	0.030619084	0.053493386	0.051417373	6.79E-07	3.49E-05
TCONS_00015997	WBGene00005079	srd-1	II	1	1116	1	1116	23.17401567	22.70186425	13.27344368	9.432116998	34.01308127	0.024040226	44.29174327	7.02E-07	3.60E-05
TCONS_00015998	WBGene00005080	srd-2	II	1	1089	1	1089	20.58886651	20.12990391	11.79821538	8.407521731	30.22103611	0.010825868	39.3917027	7.05E-07	3.61E-05
TCONS_00001531	WBGene00005171	srg-14	I	1	981	1	981	0.011540844	0	0	0.01914677	0.020794627	3.794350881	0	3.60E-14	3.38E-12
TCONS_00054709	WBGene00005200	srg-43	V	1	892	1	900	2.202599323	0	1.986246204	0.082969338	0.048138893	0.066071789	2.530010231	1.05E-15	1.16E-13
TCONS_00001524	WBGene00005272	srh-49	I	1	1038	1	1038	10.75631289	6.047178215	4.982321156	3.140070343	0.025897808	6.739035361	0.019862682	3.63E-19	5.38E-17
TCONS_00001526	WBGene00005273	srh-50	I	1	1023	1	1023	10.75631289	6.061635569	5.027161377	3.120923573	0	6.725185049	0.007008339	7.86E-21	1.26E-18
TCONS_00001525	WBGene00005274	srh-51	I	1	1047	1	1047	10.75631289	6.041149124	4.992254579	3.126447285	0.046310531	6.71857787	0	9.01E-19	1.30E-16
TCONS_00060914	WBGene00005331	srh-112	V	1	1011	1	1011	1.979919341	1.651472321	0.013763318	1.65032738	2.166966175	1.180019613	0.102869082	3.92E-09	2.45E-07
TCONS_00037440	WBGene00005487	srh-282	IV	1	807	124	930	0.046163378	2.363403649	0.086908305	0	0.04120735	0.021651736	0.005846005	1.86E-05	0.000803211
TCONS_00008936	WBGene00005543	sri-31	II	1	975	1	975	1.787128922	0.030145455	0.062433434	0.101630005	0.108312508	0.060100564	1.021064444	0.00015438	0.006023614
TCONS_00013649	WBGene00005544	sri-32	II	1	966	1	966	0	0	0.082300281	0.331422707	0.006931542	1.818915796	0.021025016	2.61E-09	1.66E-07

TCONS_00013650	WBGene00005545	sri-33	II	1	975	1	975	0	0	0.041289954	0.331422707	0	1.832130154	0	1.86E-14	1.77E-12
TCONS_00013651	WBGene00005546	sri-34	II	1	969	1	969	0.007000282	0	0.022420111	0.343328676	0	1.818915796	0	1.08E-16	1.27E-14
TCONS_00060003	WBGene00005706	sru-43	V	1	441	14435743	14435303	0.017311267	0.18062343	0.02930177	0.027618559	0.05468853	4.646676957	0.04905837	5.00E-09	3.11E-07
TCONS_00039675	WBGene00005728	srv-17	IV	1	942	1	942	0	5.663792331	0	9.284130606	0	10.03894976	3.78821108	1.53E-28	4.47E-26
TCONS_00039679	WBGene00005729	srv-18	IV	316	462	5871289	5871143	0	2.831896166	0.005605028	4.642065303	0	5.019474882	1.89410554	1.85E-24	4.56E-22
TCONS_00039680	WBGene00005730	srv-19	IV	1	972	1	972	0	5.663792331	0.044840221	9.290512863	0.044864074	10.0443627	3.809236096	5.59E-25	1.41E-22
TCONS_00039683	WBGene00005731	srv-20	IV	31	216	5875569	5875384	0.005770422	4.247844249	0.016815083	6.963097955	0.076246967	7.529212323	2.854012653	1.18E-23	2.65E-21
TCONS_00039685	WBGene00005732	srv-21	IV	1	954	1	954	0	4.964246552	0.006881659	8.136378794	0.069315424	8.784081044	3.314684695	3.74E-25	9.50E-23
TCONS_00039687	WBGene00005733	srv-22	IV	1	945	1	945	0	5.663792331	0	9.284130606	0.013863085	10.04977563	3.78821108	5.70E-28	1.63E-25
TCONS_00039691	WBGene00005734	srv-23	IV	1	201	5882335	5882135	0	6.607789208	2.25718417	7.665146202	2.081186033	8.784081044	4.604218996	4.37E-05	0.001823489
TCONS_00061476	WBGene00005832	srw-85	V	1	948	349	1296	0.209629576	0.957207738	0.106276651	0.878579702	1.205195615	1.870098356	0.587641115	0.000531828	0.018791555
TCONS_00061477	WBGene00005832	srw-85	V	1	624	1	624	0.174628168	0.766738324	0.081303277	0.684650025	0.908065421	1.454016056	0.457969683	0.000527841	0.018682773
TCONS_00052805	WBGene00005967	srx-76	V	1	966	1	966	0.058934082	0.097571554	0.168710084	2.113978186	0.13200005	0.162868647	0.059622381	0.000246115	0.009294578
TCONS_00053950	WBGene00005969	srx-78	V	1	975	1	975	0	4.660829106	0	0.022094847	0	0	0	2.93E-22	6.02E-20
TCONS_00053951	WBGene00005970	srx-79	V	249	481	15098244	15098476	0.017311267	4.660829106	0	0	0.006931542	0.005412934	0	1.77E-18	2.48E-16
TCONS_00056039	WBGene00006136	str-76	V	1	954	1	954	12.27491794	26.95632804	11.44879785	25.91389572	0	23.10228776	13.75036032	7.28E-09	4.42E-07
TCONS_00008372	WBGene00006195	str-148	II	1	993	1	993	2.443284783	0.567404375	6.316366832	0.482134897	0.675309792	0.714521931	28.90582548	2.27E-13	2.03E-11
TCONS_00049922	WBGene00006283	str-257	V	1	1059	1	1059	42.9377118	94.11115679	39.89353503	90.45291813	0.029172627	80.78413394	48.10991976	6.02E-09	3.71E-07
TCONS_00049920	WBGene00006284	str-258	V	1	1059	1	1059	43.18129939	94.60996664	40.0189004	91.48899618	0.056134988	81.33021897	48.87266633	6.65E-09	4.09E-07
TCONS_00004071	WBGene00006399	tag-4	I	1	723	1	723	0.005770422	7.712296366	10.67028075	1.063128298	0.015309542	0.592335274	0.046768038	1.52E-07	8.38E-06
TCONS_00055612	WBGene00006419	oac-39	V	42	2186	1	2145	3.286325833	0.726622236	2.933593246	3.386963908	5.884354227	1.876254193	4.015812349	0.000327299	0.012061731
TCONS_00038841	WBGene00006428	tkr-3	IV	1	1161	1	1161	14.44452128	59.77705382	0.343025196	47.85366583	0.335826534	0.235625278	0.425218322	4.42E-21	7.29E-19
TCONS_00038839	WBGene00006428	tkr-3	IV	1	1203	1	1188	14.44452128	59.77705382	0.355511883	47.85918955	0.335826534	0.235625278	0.425218322	5.26E-21	8.52E-19
TCONS_00038840	WBGene00006428	tkr-3	IV	1	1188	1	1188	14.44452128	59.77705382	0.349906855	47.85366583	0.335826534	0.235625278	0.425218322	4.87E-21	7.95E-19
TCONS_00030089	WBGene00006459	tag-89	IV	1	1194	1	1194	0.745241391	0.679433282	0.44463307	0.678898989	0.728633026	24.70877121	0.56080443	9.67E-05	0.003887341
TCONS_00005620	WBGene00006625	try-7	I	27	1169	1	1143	0.881650681	0.997993653	0.876341705	2.184782776	0.633419794	0.584766843	0.165875459	0.000900483	0.030383179
TCONS_00005524	WBGene00006689	twk-37	I	32	1180	1	1149	23.79489221	81.25873439	23.00104864	6.876638647	48.52574494	12.55546881	0.070117722	0.000558114	0.019636057
TCONS_00018264	WBGene00006822	unc-93	III	1	2118	1	2118	0.913720261	0.893956736	19.38625851	1.50321378	1.923044692	1.095582809	0.986160094	0.001068142	0.034703219
TCONS_00018265	WBGene00006822	unc-93	III	1	2106	13	2118	0.913720261	0.893956736	19.38625851	1.496831523	1.923044692	1.095582809	0.979151755	0.001081193	0.035071889
TCONS_00062711	WBGene00006952	wrt-6	X	52	1623	211	1782	2.429552044	4.193052997	5.025143232	1.169360556	1.5487414	0.54624035	10.68840933	0.000246833	0.009313121
TCONS_00062710	WBGene00006952	wrt-6	X	4	1785	1	1782	2.589800772	4.556823677	5.600369703	1.276514273	1.617293016	0.591296357	11.86342195	0.000260019	0.009756999
TCONS_00005640	WBGene00007057	ant-1.2	I	1	1390	6731110	6729721	0.08447549	0.037342969	0.07569825	1.369880523	0.951368515	0.019821537	1.944291909	0.000354209	0.012937569
TCONS_00053952	WBGene00007230	C01G10.1	V	171	3812	1	3642	0.992332094	9.79912482	0.937279632	0.66132419	0.336972248	0.739120447	0.164713125	2.05E-08	1.20E-06
TCONS_00053947	WBGene00007231	C01G10.4	V	1	219	1	219	0.375077445	1.639149097	0.499187841	0.243643083	0.07144453	0.119084548	0.012854343	7.18E-06	0.000324841
TCONS_00060261	WBGene00007232	C01G10.5	V	1	228	1	228	0.017311267	1.349752732	0.044840221	0.336946419	0.188516942	0.148537708	0.028033354	2.23E-08	1.30E-06
TCONS_00053946	WBGene00007233	C01G10.6	V	93	395	15088034	15088336	0.248885906	1.639024448	0.258390527	1.017190057	0.58260757	0.694297432	0.357459604	0.000132949	0.005261056

TCONS_00060259	WBGene00007240	C01G10.15	V	1	303	1	303	0.196573231	1.531793883	0.225258863	0.39266964	0.269785929	0.495446111	0.177567468	4.94E-05	0.002047199
TCONS_00053949	WBGene00007241	C01G10.16	V	180	416	1	237	0.070853801	2.754935705	0.137852056	0.141526975	0.157134049	0.156261468	0.071280055	5.59E-05	0.002305812
TCONS_00022406	WBGene00007271	C03C10.5	III	280	609	4089940	4089611	9.796786165	1.701792598	2.634630142	2.710072246	3.111183564	1.649376949	13.60262299	0.000999503	0.032683837
TCONS_00006668	WBGene00007275	C03D6.1	I	1	789	1	789	0.285117174	0.291608563	3.035152847	0.226585845	0.269485185	0.105792526	0.217327167	0.000131182	0.005207306
TCONS_00016037	WBGene00007416	ceh-57	II	83	1114	1	1032	23.28195172	0.040972889	13.53410785	9.865883137	34.42149805	0.434398935	0.344639596	6.93E-08	3.91E-06
TCONS_00054915	WBGene00007439	C08E8.3	V	1	237	1	237	0.03131183	0.006029091	0.022420111	1.166216613	0.490067669	3.507688184	0.287341882	1.18E-05	0.000529363
TCONS_00042051	WBGene00007446	mboa-4	IV	32	1567	1	1536	2.538339082	2.596793107	2.382622772	2.024387101	1.495418167	13.59818082	0.919426368	0.000631742	0.02196372
TCONS_00042050	WBGene00007446	mboa-4	IV	32	1654	1	1623	2.538339082	2.596793107	2.382622772	2.024387101	1.505624528	13.59818082	0.919426368	0.000656455	0.022784424
TCONS_00042052	WBGene00007446	mboa-4	IV	32	1522	1	1491	2.472025843	2.570277572	2.300820993	1.97038097	1.447279274	13.55909604	0.898401352	0.000510835	0.01814324
TCONS_00022423	WBGene00007629	C16C10.9	III	15	839	1	825	0.077475208	0.266199126	0.180917143	0.038293541	0.241377111	0.106986771	22.36671118	3.64E-09	2.28E-07
TCONS_00060978	WBGene00007673	C18D4.3	V	1	372	1	372	1.931768353	0.022885616	2.921884688	1.470651984	3.462782706	0.069096233	2.03942653	8.21E-18	1.10E-15
TCONS_00060980	WBGene00007675	C18D4.6	V	19	981	1	963	6.798612655	2.48741267	9.934514963	6.028933027	10.79684606	3.559225326	7.937515067	0.000189306	0.007235473
TCONS_00060981	WBGene00007675	C18D4.6	V	19	915	1	897	6.838154626	2.465757802	9.988790107	5.93379894	10.68708709	3.556200881	7.943361072	0.000170373	0.006597579
TCONS_00060979	WBGene00007675	C18D4.6	V	36	217	17535934	17535753	1.987484965	0.198897676	3.126000072	1.726832257	3.681918732	0.170747734	2.270873381	1.41E-12	1.18E-10
TCONS_00060982	WBGene00007675	C18D4.6	V	1	391	1	395	3.9305204	1.503814046	5.661636354	3.618050511	6.357966641	2.450831122	4.664959225	0.000668957	0.023179264
TCONS_00054705	WBGene00007676	srz-57	V	1	978	1	981	2.983308292	0.012058182	6.605797645	3.231371395	6.914017653	0	4.835753628	6.37E-25	1.59E-22
TCONS_00060986	WBGene00007677	C18D4.8	V	1	2024	1	2028	6.529670122	1.551922124	12.45517205	7.463153679	13.17161062	2.078440614	9.705867313	6.21E-08	3.52E-06
TCONS_00060246	WBGene00007715	C25D7.2	V	84	663	15032468	15033037	1.536168111	3.637332856	1.22796294	1.035219502	0.75925211	2.270063444	0.498823045	0.000139881	0.005494337
TCONS_00055195	WBGene00007723	C25F9.2	V	1480	6035	1	4572	0.687350701	2.692105883	0.316276689	2.784506881	3.455559882	5.659895063	1.392540725	4.38E-05	0.00182427
TCONS_00055196	WBGene00007723	C25F9.2	V	1	4572	1	4572	0.638727604	2.633045722	0.281369892	2.71478816	3.380076724	5.380408022	1.357499032	3.76E-05	0.001576165
TCONS_00054332	WBGene00007829	oac-5	V	45	2174	1	2130	0.488783921	1.161324286	0.249733734	0.908773896	0.609951835	0.887735819	0.177636139	1.44E-05	0.000639133
TCONS_00069978	WBGene00007938	C34F6.1	X	8	3139	1	3132	1.523688988	2.591994765	0.617951178	0.708461962	0.485045649	0.40024646	0.176405135	0.000709603	0.024443612
TCONS_00018300	WBGene00007963	cyp-25A1	III	50	1558	1	1509	26.59300126	45.53955758	22.55528705	30.54722037	19.50038074	33.03555768	10.30776664	0.001542784	0.04847875
TCONS_00022335	WBGene00007970	C36A4.10	III	1	114	1	114	12.50008319	23.42781405	10.08755426	14.34414292	10.08005207	15.54477833	0.530549414	6.46E-07	3.33E-05
TCONS_00022376	WBGene00007981	glb-11	III	389	1078	76	765	13.38892062	11.2002593	1.165031005	12.30385449	7.224667365	12.52096303	8.925891746	0.000122503	0.004891107
TCONS_00022378	WBGene00007981	glb-11	III	13	777	1	765	16.65699691	13.92638986	1.351054674	15.32071754	8.821351975	15.49681501	11.01095832	8.36E-05	0.003398042
TCONS_00022377	WBGene00007981	glb-11	III	104	621	3990837	3990320	13.58748146	11.42911546	1.381633075	12.76352209	7.590048431	13.01617615	9.344170401	0.000220343	0.008375064
TCONS_00054710	WBGene00008014	fbxa-172	V	1	1671	1	1671	5.120736941	1.054093722	4.634007176	1.245461535	0.774862396	0.994008972	4.630221477	0.000852667	0.028947246
TCONS_00060988	WBGene00008015	fbxa-174	V	1	1218	1	1218	1.820049487	0.08674412	1.67224315	0.111446442	0.034275808	0.196462831	2.052280873	4.62E-08	2.63E-06
TCONS_00060987	WBGene00008016	C38D9.8	V	1	1035	1	1035	1.393510351	0.289147067	1.540652746	0.166311118	0.093002966	0.114229905	1.616601547	3.96E-06	0.000180546
TCONS_00054708	WBGene00008017	fbxa-176	V	47	1078	1	1032	2.073854831	0.398714478	1.935740202	0.209269826	0.50027403	0.182054229	2.260206363	1.29E-09	8.49E-08
TCONS_00030691	WBGene00008026	scl-4	IV	183	533	13062511	13062861	0.028001127	0.008428262	0	0.972173274	0.006931542	0	0	1.59E-12	1.33E-10
TCONS_00022268	WBGene00008180	C48D5.3	III	40	537	1	498	0.364579992	4.908786126	4.706727718	3.823716704	0.144798582	3.411134506	2.497327542	0.000435647	0.015661981
TCONS_00059605	WBGene00008220	C50B6.7	V	1	2142	1	2142	9.728624271	33.86426298	9.165763157	14.95313237	1.666496461	10.86562377	14.82810563	6.48E-05	0.00266045
TCONS_00053760	WBGene00008593	clec-227	V	15	1160	1	1146	4.492449191	3.483240705	1.456176199	2.488492552	0.871221342	0.944889603	0.420637658	0.000253592	0.00953319
TCONS_00071267	WBGene00008621	F09C8.1	X	52	1176	1	1125	1.381975446	5.643742811	0.246681969	1.604761581	0.374742457	0.899585962	0.672084533	2.59E-08	1.50E-06

TCONS_00004046	WBGene00009022	F21F12.1	I	158	736	1	579	0.061772676	8.271755332	10.46029319	20.49724536	0.190345304	0.422301158	0.060784715	1.47E-08	8.73E-07
TCONS_00004047	WBGene00009022	F21F12.1	I	15	593	1	579	0.061772676	8.271755332	10.46029319	20.49724536	0.190345304	0.422301158	0.060784715	1.47E-08	8.73E-07
TCONS_00042140	WBGene00009036	F22B3.5	IV	285	899	1	615	0.025541408	0	0.034906797	0	0.015309542	19.8269496	0	5.61E-09	3.46E-07
TCONS_00042141	WBGene00009036	F22B3.5	IV	285	869	1	585	0.025541408	0	0.034906797	0	0.015309542	19.8269496	0	5.61E-09	3.46E-07
TCONS_00042142	WBGene00009038	F22B3.7	IV	1	1140	1	1140	0	0	0	0	0	27.75015133	0	3.61E-30	1.27E-27
TCONS_00010857	WBGene00009042	F22B5.4	II	4	498	1	495	11.89810468	2.439912094	1.497004267	10.18703268	22.14503131	1.336621021	14.9298073	0.001412302	0.044849159
TCONS_00005956	WBGene00009042	F22B5.4	I	13	693	1	681	83.49117095	12.67124729	12.08549572	18.73519643	40.49253238	8.762647658	57.2249829	0.000862352	0.029240398
TCONS_00005959	WBGene00009142	F26A3.4	I	57	737	1	681	83.49117095	12.69653207	12.08549572	18.73519643	40.49253238	8.762647658	57.2308289	0.000870812	0.029454494
TCONS_00005957	WBGene00009142	F26A3.4	I	1	932	7658548	7657617	74.02712918	11.30382781	10.96346905	17.0662713	36.6587212	7.680279208	50.64102335	0.000792971	0.027066201
TCONS_00005958	WBGene00009142	F26A3.4	I	1	986	7659587	7658602	84.05355403	12.94470625	12.3201895	19.09360648	41.20372675	8.965795333	57.77067699	0.000919258	0.030864888
TCONS_00041515	WBGene00009324	F32B6.10	IV	1	1689	1	1689	0.983437437	0.60370357	0.886117007	0.459667608	14.49003619	13.78731585	9.946029129	0.001162954	0.037458404
TCONS_00060573	WBGene00009486	F36G9.7	V	1	669	1	669	0.820635755	0.623083916	1.448674532	0.375726063	1.261817568	0.414810391	0.835429311	0.000346206	0.012690303
TCONS_00065531	WBGene00009555	C02B4.t2	X	1	2551	12663626	12666176	30.36510791	2.415733405	2.400933361	6.683572557	12.27384218	3.220909629	40.61214427	0.000231343	0.008776972
TCONS_00059943	WBGene00009600	F40G12.5	V	106	900	1	795	2.970741873	0.269891371	1.711393455	6.115924144	15.75013943	6.788169371	12.61769271	1.41E-05	0.000627415
TCONS_00059944	WBGene00009600	F40G12.5	V	76	870	1	795	2.970741873	0.269891371	1.704511796	6.115924144	15.75013943	6.788169371	12.61769271	1.43E-05	0.000636899
TCONS_00059942	WBGene00009600	F40G12.5	V	11	805	1	795	2.964971451	0.269891371	1.692025109	6.115924144	15.73627635	6.774955013	12.6060007	1.48E-05	0.000650724
TCONS_00065461	WBGene00009635	F42F12.3	X	1	732	1	732	11.23771766	33.59421873	6.103485631	124.3950798	5.881427951	5.536602012	7.034342264	1.20E-07	6.65E-06
TCONS_00070319	WBGene00009636	F42F12.4	X	3	539	1	537	11.28482525	40.69728234	6.746665673	154.024823	6.207210446	5.470544865	6.299835059	1.65E-08	9.75E-07
TCONS_00070320	WBGene00009636	F42F12.4	X	33	569	1	537	11.28482525	40.69728234	6.746665673	154.024823	6.207210446	5.470544865	6.299835059	1.65E-08	9.75E-07
TCONS_00054269	WBGene00009706	F44G3.2	V	88	1206	1	1119	0.393904212	1.566379478	1.403335809	1.061979516	0.502866201	1.182189753	0.077091725	0.000144101	0.005638578
TCONS_00054270	WBGene00009706	F44G3.2	V	107	1225	1	1119	0.393904212	1.566379478	1.403335809	1.061979516	0.502866201	1.182189753	0.077091725	0.000144101	0.005638578
TCONS_00054271	WBGene00009706	F44G3.2	V	42	1160	1	1119	0.38813379	1.557951215	1.39645415	1.056455804	0.490831478	1.182189753	0.077091725	0.000129749	0.005160378
TCONS_00015484	WBGene00009778	F46C5.1	II	35	400	1	366	15.92530593	37.12294858	41.29613686	40.85891449	16.37502837	42.69598338	8.089437442	0.001202214	0.038602122
TCONS_00030681	WBGene00009892	scl-11	IV	1	624	1	624	4.678886027	1.090642215	3.924807641	2.702945106	8.913261514	1.431820672	1.400505385	0.00110914	0.035893569
TCONS_00053207	WBGene00010062	lipl-1	V	1	1218	1	1218	16.40373315	45.71768802	9.861369977	32.02502887	1.850674032	15.37482633	5.368564114	1.24E-11	9.71E-10
TCONS_00042131	WBGene00010352	fbxb-76	IV	1	531	1	531	0.139626759	0.146972705	0.185525167	0.142871623	0.133064603	8.187008259	0.187003481	0.000513197	0.01821143
TCONS_00041507	WBGene00010634	K07F5.6	IV	7	1419	1	1413	8.652405787	7.918345629	6.882304848	1.745398554	6.419968618	1.405950247	6.114861262	0.000557955	0.019636057
TCONS_00042653	WBGene00010657	K08D8.3	IV	31	1470	1	1440	2.515164159	1.958083415	4.756588751	1.804959853	6.237838992	1.246509006	2.626562762	0.000102716	0.004125081
TCONS_00042652	WBGene00010657	K08D8.3	IV	41	1465	1	1440	2.515164159	1.958083415	4.756588751	1.804959853	6.237838992	1.25192194	2.620716757	0.000106533	0.004274214
TCONS_00003408	WBGene00010769	asah-1	I	28	1209	1	1182	36.26551044	39.32599613	17.80821647	46.53466591	8.611247721	21.85303568	21.24329538	0.001031848	0.033656888
TCONS_00003409	WBGene00010769	asah-1	I	28	1209	1	1182	36.26551044	39.32599613	17.80821647	46.54104817	8.604316179	21.85303568	21.24329538	0.001028343	0.033569159
TCONS_00020370	WBGene00010781	K11H3.4	III	7	1101	1	1095	2.350561335	2.43075168	16.1598781	2.083203517	1.747001639	1.744265943	8.017741478	2.86E-05	0.001212785
TCONS_00020374	WBGene00010782	K11H3.5	III	1	324	1	324	0.176802245	0.171151393	4.837138859	0.212590344	0.275652919	0.229654054	1.950172249	5.30E-11	3.97E-09
TCONS_00020375	WBGene00010782	K11H3.5	III	1	406	1	406	0.199883934	0.189238666	6.428966711	0.212590344	0.306272003	0.25369428	2.589711435	1.59E-12	1.33E-10
TCONS_00011444	WBGene00010831	M02G9.2	II	32	1228	1	1197	3.055677594	7.937664302	0.455624251	0.55025019	0.559383093	0.210390808	0.119176092	0.000136864	0.005391277
TCONS_00011443	WBGene00010832	M02G9.3	II	1	885	1	885	0.089016052	12.70083312	0.030578401	0.124955839	0.04958535	0.081116346	0.05493871	4.83E-10	3.28E-08

TCONS_00015341	WBGene00010877	lact-4	II	6	1295	1	1290	27.09769615	5.600230358	8.70745166	9.68823228	17.56393603	8.104891192	42.29199254	0.000375838	0.013606857
TCONS_00049312	WBGene00010966	MTCE.34	MtDNA	1	336	11356	11691	125.1421468	170.8479357	80.02278086	203.1128792	136.1699243	107.4203285	18.98303438	0.000135036	0.005324335
TCONS_00011416	WBGene00011041	R05H5.7	II	1	612	1	612	13.0443141	12.14316517	8.651206643	11.64511602	15.30596654	0.043861763	19.72740604	0.000730218	0.025111703
TCONS_00022331	WBGene00011260	R13G10.4	III	1979	4147	1	2169	57.26296943	104.2426905	51.63379266	68.33370499	50.93876765	73.65000979	11.95163601	0.000245577	0.009282767
TCONS_00022334	WBGene00011260	R13G10.4	III	1	2169	1	2169	32.13786513	60.65668307	27.09453304	37.58028366	25.94918787	40.28011766	1.632089578	9.46E-07	4.83E-05
TCONS_00022332	WBGene00011260	R13G10.4	III	2611	4779	1	2169	57.85572013	105.137878	52.58000872	69.42289916	52.06721988	74.61647347	13.16233763	0.000367899	0.013366453
TCONS_00022333	WBGene00011260	R13G10.4	III	2556	4724	1	2169	57.71108069	104.8366728	52.13942442	69.09382477	51.71493809	74.23063561	12.7439903	0.00031824	0.011741322
TCONS_00015859	WBGene00011678	T10B9.9	II	8	382	1	375	0.493989	0.192868586	0.695643436	0.817105446	1.140625367	0.337912735	0.881206693	0.001452067	0.045908521
TCONS_00042259	WBGene00011725	lips-5	IV	107	1096	1	990	0.672306746	0.997806679	0.307060642	0.481276352	0.202380027	0.778360558	0.3377686	0.000249089	0.009389651
TCONS_00042261	WBGene00011725	lips-5	IV	107	1129	1	1023	0.69453745	0.941145689	0.300178983	0.508036366	0.212586388	0.76753469	0.32607659	0.00026177	0.009787004
TCONS_00042262	WBGene00011725	lips-5	IV	107	1138	1	1032	0.69453745	0.941145689	0.300178983	0.508036366	0.212586388	0.76753469	0.32607659	0.00026177	0.009787004
TCONS_00029509	WBGene00011744	T13F2.4	IV	1	864	1	864	0.698040561	2.736303255	0.475989601	0.188778407	10.52356573	21.11991892	0.231275174	0.000263938	0.009850158
TCONS_00024172	WBGene00012060	T26G10.3	III	1	276	1	276	0	0.006029091	1.720743488	0	0.025897808	0.012020113	0.019862682	8.31E-13	7.09E-11
TCONS_00022291	WBGene00012084	npr-15	III	583	1115	506	1038	0.500045063	0.198835352	9.612245418	0.443955018	0.544455456	0.257277014	0.592496463	8.86E-07	4.53E-05
TCONS_00022290	WBGene00012084	npr-15	III	17	1054	1	1038	0.493044781	0.192806261	8.480029833	0.443955018	0.527317552	0.239843967	0.592496463	8.29E-07	4.24E-05
TCONS_00007455	WBGene00012213	W02D9.7	I	1	398	12559672	12559275	29.56935734	30.5919499	26.49400448	59.77927024	28.76440042	42.08627107	7.879001576	0.000814729	0.027785838
TCONS_00041492	WBGene00012340	dct-15	IV	27	1658	1	1632	1.396553231	4.377602225	0.700689209	0.403458284	21.26354635	38.04911034	0.670544508	0.00019689	0.00751138
TCONS_00041491	WBGene00012340	dct-15	IV	27	1706	1	1680	1.291549005	3.912754721	0.661454015	0.403458284	18.94436308	33.85408648	0.635502815	0.000309649	0.011452292
TCONS_00041490	WBGene00012340	dct-15	IV	1	1689	1	1689	1.291549005	3.912754721	0.667059043	0.409840541	18.94436308	33.86730084	0.642511154	0.000329647	0.012137392
TCONS_00022374	WBGene00012380	Y1A5A.2	III	1	252	1	252	6.178572863	11.61448935	0	7.044969258	4.845873636	5.193242606	3.392035878	1.77E-06	8.85E-05
TCONS_00070913	WBGene00012445	Y16B4A.2	X	28	6513	1	6486	9.490743562	31.69400805	8.765277068	11.90009172	4.548605019	7.665511568	4.921678551	0.00026096	0.009774489
TCONS_00070912	WBGene00012445	Y16B4A.2	X	2810	6435	2861	6486	9.396429197	31.36155125	8.677870261	11.8371277	4.491706223	7.578982288	4.871423512	0.000253563	0.00953319
TCONS_00012130	WBGene00012587	Y38E10A.9	II	5	238	1	234	0.30290055	1.992669009	0.517218803	0.794297173	0.274507205	0.129910416	0.123928434	0.00101958	0.033309505
TCONS_00012133	WBGene00012593	nspe-7	II	21	245	1	225	0.065083379	0.214585778	0.319766203	4.263156717	1.424331642	3.406177369	0.231275174	4.45E-05	0.001852263
TCONS_00061794	WBGene00012615	dct-16	V	46	377	20498253	20497922	24.62282398	49.42213861	7.034503611	16.04660502	4.886245478	7.99393782	10.00428436	0.000182623	0.006990947
TCONS_00061786	WBGene00012615	dct-16	V	1	396	20498317	20497922	17.25066627	34.67228777	4.889249392	11.02653538	3.40417007	5.544004929	6.864332503	0.00018195	0.006973729
TCONS_00061790	WBGene00012615	dct-16	V	57	377	20498242	20497922	24.6285944	49.47037134	7.040108639	16.06955841	4.886245478	8.000544999	10.01129269	0.000180964	0.006942413
TCONS_00061788	WBGene00012615	dct-16	V	37	357	20498242	20497922	24.88864227	50.10090207	7.112973998	16.21832619	4.932556009	8.081739009	10.10246977	0.000178476	0.006859729
TCONS_00061792	WBGene00012615	dct-16	V	65	384	20498241	20497922	32.75989488	66.07687057	9.327263683	21.49211413	6.515630478	10.66158232	13.3826914	0.000173997	0.006693825
TCONS_00061795	WBGene00012615	dct-16	V	63	383	20498242	20497922	25.14168987	50.69162833	7.142275768	16.31947009	4.983587816	8.154495641	10.14455413	0.000172654	0.006654617
TCONS_00061791	WBGene00012615	dct-16	V	63	383	20498242	20497922	32.73558334	65.94546132	9.326765181	21.47468445	6.505424117	10.66277656	13.35462371	0.000172505	0.006654617
TCONS_00061793	WBGene00012615	dct-16	V	63	382	20498241	20497922	25.12891916	50.67957015	7.142275768	16.29565815	4.963175093	8.142475528	10.14455413	0.000171716	0.00663088
TCONS_00061785	WBGene00012615	dct-16	V	79	406	20498242	20497915	25.12891916	50.67957015	7.142275768	16.29565815	4.963175093	8.149082707	10.14455413	0.000170967	0.006608175
TCONS_00061796	WBGene00012615	dct-16	V	78	397	20498241	20497922	25.12891916	50.67957015	7.142275768	16.29565815	4.963175093	8.149082707	10.14455413	0.000170967	0.006608175
TCONS_00061789	WBGene00012615	dct-16	V	63	381	20498240	20497922	32.73558334	65.94546132	9.299238544	21.46916074	6.498492574	10.65616938	13.34761537	0.00016991	0.006585861
TCONS_00061787	WBGene00012615	dct-16	V	41	358	20498239	20497922	33.00017177	66.64950956	9.350960425	21.56944609	6.534214839	10.72231884	13.41892976	0.000166586	0.006463752

TCONS_00061403	WBGene00012664	Y39B6A.1	V	594	1703	493	1590	5.592245229	33.34756857	1.638248468	2.302144661	1.015857603	2.94587427	1.787440436	5.33E-07	2.78E-05
TCONS_00061404	WBGene00012664	Y39B6A.1	V	11	2218	1	2208	5.823726635	34.57656825	1.648181892	2.478158555	1.038098687	3.02762657	1.856395824	3.53E-07	1.89E-05
TCONS_00036268	WBGene00013102	Y51H4A.6	IV	1	231	1	231	0	0.036174546	0.975274811	0	0.006931542	1.266626558	2.806082282	6.19E-07	3.20E-05
TCONS_00032873	WBGene00013338	lgc-8	IV	1	1317	1	1317	0.089016053	0.038573717	0.209221909	0.397334807	0.493724392	0.158091667	0.670544508	0.000529815	0.018736515
TCONS_00046524	WBGene00013636	Y105C5A.12	IV	1	231	1	231	0.738520812	1.088679316	0.507066504	1.950211246	1.421357566	0.906270805	0.213771495	0.001438539	0.045515757
TCONS_00036789	WBGene00013772	clec-192	IV	73	477	211	615	0	0	1.012734871	0.023811937	0.022241085	0.022845981	4.2231401	7.97E-23	1.70E-20
TCONS_00048637	WBGene00013773	Y116A8A.2	IV	1	510	1	510	0.118247039	0.118120323	0.172758853	0.107526159	0.064894892	0.034866094	2.857499655	0.000148335	0.005798736
TCONS_00048626	WBGene00013774	clec-193	IV	21	635	1	615	0.023081689	0.024116364	0.080525148	0.052289041	0.027344265	0.069732188	4.28857415	2.38E-07	1.28E-05
TCONS_00054945	WBGene00013947	ZK228.4	V	1	1113	18462542	18463654	0.794808706	3.428791913	1.076128692	2.021666347	0.995306458	2.618942732	0.984860418	0.000130301	0.005177307
TCONS_00054946	WBGene00013947	ZK228.4	V	66	976	1	930	0.74372589	3.169727976	0.978788461	1.760966025	1.000791543	2.341921845	0.938126716	0.000365974	0.013320021
TCONS_00054948	WBGene00013947	ZK228.4	V	1	930	1	930	0.74372589	3.190214421	0.973183434	1.772013449	1.000791543	2.360549137	0.945135054	0.00033337	0.012238158
TCONS_00054944	WBGene00013947	ZK228.4	V	274	1203	1	930	0.74372589	3.190214421	0.98567012	1.778395706	1.000791543	2.417066967	0.945135054	0.000307874	0.011417393
TCONS_00054947	WBGene00013947	ZK228.4	V	12	896	1	885	0.730955187	3.149241533	0.966301775	1.749060056	0.986928458	2.323294553	0.938126716	0.000357037	0.013026451
TCONS_00054943	WBGene00013947	ZK228.4	V	274	1174	1	885	0.730955187	3.163698886	0.978788461	1.760966025	0.986928458	2.391832496	0.938126716	0.000308844	0.011432785
TCONS_00054949	WBGene00013947	ZK228.4	V	1	813	1	813	0.704183919	3.081753108	0.941328401	1.680572322	0.943892746	2.246396896	0.887871676	0.000308839	0.011432785
TCONS_00020142	WBGene00013988	ZK512.10	III	15	284	1	270	4.077893313	3.008874584	2.60380873	2.861964675	1.896578759	1.895410491	0.213771495	0.001527707	0.048078398
TCONS_00042640	WBGene00014132	ZK896.1	IV	16	1455	1	1440	0.156180276	1.234904125	0.325930486	1.166526141	0.513153724	0.708628371	0.453320348	0.00075686	0.026006154
TCONS_00007167	WBGene00014183	ZK1025.3	I	17	1033	1	1017	3.245268361	5.183366283	0.999153811	1.035446825	0.858503971	1.001252105	0.537317404	0.001434209	0.045413662
TCONS_00007168	WBGene00014183	ZK1025.3	I	17	1033	1	1017	3.245268361	5.183366283	0.999153811	1.035446825	0.858503971	1.001252105	0.537317404	0.001434209	0.045413662
TCONS_00060985	WBGene00014263	C18D4.t1	X	1	73	91	8	1.188706979	0	2.653840365	1.292548558	2.753359428	0	1.934301451	1.47E-20	2.35E-18
TCONS_00070318	WBGene00014373	F42F12.t1	X	1	84	1359034	1359117	3.218776795	15.40160082	1.62801128	60.70560085	1.389802889	1.763869129	1.859029512	3.29E-11	2.51E-09
TCONS_00049306	WBGene00014468	MTCE.28	MtDNA	1	59	9478	9536	0	0.012058182	0.031855032	0.006382257	2.155370617	0.08414079	0.007008339	8.58E-05	0.003477077
TCONS_00048286	WBGene00014584	Y51H4A.t6	IV	1	82	16577306	16577387	0	0	0.487637405	0	0	0.633313279	1.403041141	2.07E-06	9.58E-05
TCONS_00011437	WBGene00014636	ZK970.t2	X	1	72	470248	470177	0	2.514130923	0	0	0	0	0	3.85E-18	5.22E-16
TCONS_00016026	WBGene00014637	ZK970.t3	II	1	72	82	11	0	2.514130923	0	0	0	0	0	3.85E-18	5.22E-16
TCONS_00054704	WBGene00014679	srz-25	V	1	918	1	918	2.377413958	0	5.280154094	2.585097116	5.506718855	0.046250252	3.868602902	3.81E-22	7.80E-20
TCONS_00029495	WBGene00014689	C28D4.11	IV	1	1111	9748815	9749925	0.005770422	0	0	0.01914677	0.006931542	1.85122343	0.021025016	1.68E-12	1.40E-10
TCONS_00053206	WBGene00014770	F54F3.2	V	71	1200	76	1202	0.171789573	0.907183341	0.119261839	0.827294324	0.160490029	0.591776984	0.116851424	2.74E-10	1.92E-08
TCONS_00030085	WBGene00014828	T01G1.t1	IV	1	73	11362125	11362197	0	0.008428262	0	0.038293541	0.013863085	4.017164764	0	2.64E-17	3.46E-15
TCONS_00042128	WBGene00014829	T01G1.t2	IV	1	73	11362125	11362197	0	0.008428262	0	0.038293541	0.013863085	4.017164764	0	2.64E-17	3.46E-15
TCONS_00004022	WBGene00014921	Y54E5B.5	I	1	1460	14824493	14825952	0.011540844	0.012058182	1.126610557	0	0.020412723	0.037254584	0.014016677	3.83E-07	2.05E-05
TCONS_00026802	WBGene00014997	AC7.3	IV	1	711	1	711	10.3620874	38.93287055	1.337230603	30.66824256	1.445971239	1.005145496	0.603982459	5.92E-16	6.66E-14
TCONS_00013304	WBGene00015012	bath-20	II	261	1076	1	816	1.047570659	0.330119956	1.584508444	0.285370805	1.622291136	1.18583551	0.591196787	0.001285937	0.041161975
TCONS_00013611	WBGene00015198	clec-2	II	1	1236	1	1236	1.14500332	0.368880646	0.018091714	0.335715432	0.046310531	1.201671349	0.49759204	1.24E-08	7.39E-07
TCONS_00049921	WBGene00015269	C01B4.2	V	67	276	2408819	2409029	12.27491794	26.88890194	11.38941618	25.9385662	0	23.07585904	13.75036032	7.16E-09	4.37E-07
TCONS_00038812	WBGene00015327	snpn-1	IV	326	733	5090697	5090290	9.271955652	26.1731826	3.333763091	19.859531	3.921605904	4.53234456	5.296710505	6.75E-06	0.000305656

TCONS_00050434	WBGene00015339	C02E7.6	V	36	248	1	213	1.527366688	3.33703164	3.796244865	5.297890116	2.259424915	1.792006455	0.217327168	0.000771098	0.026363351
TCONS_00056634	WBGene00015340	C02E7.7	V	30	230	1	201	1.257858812	2.785128393	2.319107445	4.592376231	1.930611084	1.937675046	0.37506629	0.000563536	0.019793005
TCONS_00056261	WBGene00015439	C04E12.10	V	1	2538	1	2538	11.59524391	1.890361437	15.02181447	23.81926017	8.310455828	0.556274936	11.99729516	1.75E-06	8.75E-05
TCONS_00056695	WBGene00015449	ugt-63	V	24	1544	1	1521	0.262035485	3.191632143	0.640152414	1.72919986	0.877007171	1.904435443	0.32607659	2.21E-07	1.21E-05
TCONS_00038794	WBGene00015630	C09B9.7	IV	269	3010	1	2742	2.07292843	0.862518208	0.648249952	3.713102393	1.324779037	0.473639046	0.078288394	0.000257213	0.009660519
TCONS_00038793	WBGene00015630	C09B9.7	IV	1	2742	1	2742	1.573827585	0.828805159	0.598303205	2.589472046	0.95003658	0.377478144	0.066596385	0.00038941	0.014085842
TCONS_00038791	WBGene00015630	C09B9.7	IV	360	815	5056047	5055592	17.00664221	24.06476291	12.08616482	19.61270748	8.528045315	6.775886982	3.058647005	0.001406988	0.044723758
TCONS_00001528	WBGene00015685	C10G11.1	I	137	576	214	653	3.940178747	2.343898655	1.901830409	0.289922309	0.102827423	2.922280932	0.111005419	2.64E-06	0.000121923
TCONS_00001527	WBGene00015685	C10G11.1	I	27	722	1	696	14.92314684	8.550170082	7.017675224	4.606303773	0.142206411	11.91487866	0.17643947	4.15E-16	4.69E-14
TCONS_00005532	WBGene00015690	C10G11.10	I	26	709	1	684	25.4432513	14.64914862	11.9434476	7.568384352	0.203143836	15.88797222	0.046733702	9.84E-19	1.41E-16
TCONS_00027884	WBGene00015701	C11D2.3	IV	383	738	11	366	0.891390384	0.648368703	0.911747004	0.894437411	6.166213215	1.186486106	1.668499619	0.00106224	0.034538739
TCONS_00027883	WBGene00015701	C11D2.3	IV	82	594	6151944	6152457	1.024016861	0.749570177	1.094718908	1.00883193	6.559783516	1.341553329	2.078504594	0.000636488	0.022110049
TCONS_00067699	WBGene00015715	C12D12.4	X	1	369	1	369	0	0.269704397	0.72945586	0.006382257	0	0.013214358	2.244865825	8.76E-10	5.82E-08
TCONS_00008624	WBGene00015827	math-13	II	1	828	1	828	1.454531216	0.597986102	0.022420111	0.627840935	0.116690508	1.499397362	0.462825032	1.95E-10	1.39E-08
TCONS_00013253	WBGene00015832	math-16	II	141	875	1	735	1.484613186	0.708846586	0.183470406	0.622317223	0.253411834	1.603995643	0.534105087	2.10E-05	0.000902175
TCONS_00013255	WBGene00015833	math-17	II	1	654	1	654	1.446301075	0.652185596	0.054275143	0.678898989	0.20931157	1.561250461	0.462825032	2.22E-08	1.30E-06
TCONS_00013254	WBGene00015833	math-17	II	225	720	159	654	1.452071497	0.660613858	0.054275143	0.691663503	0.202380027	1.566663395	0.46983337	1.58E-08	9.34E-07
TCONS_00013256	WBGene00015834	math-5	II	1	849	1	849	1.454531216	0.713707253	0.033630166	0.846813536	0.447876927	1.54023468	0.529421416	6.54E-07	3.37E-05
TCONS_00008623	WBGene00015840	math-11	II	1	495	1	495	1.475153186	0.517208749	0.45185511	0.668596449	0.199487113	1.558861972	0.455816693	0.000917519	0.030856827
TCONS_00013684	WBGene00015913	C17F4.7	II	31	591	1	561	187.5048298	41.87081768	70.40991049	7.439980087	26.5666105	8.538314232	33.9189113	1.88E-05	0.000810275
TCONS_00025330	WBGene00015994	C18H7.4	IV	98	1270	1	1173	2.777292878	14.72972325	2.581193881	2.313823307	1.948780179	1.845203826	0.920588701	0.000706705	0.024379323
TCONS_00025331	WBGene00015994	C18H7.4	IV	88	1260	1	1173	2.777292878	14.72972325	2.581193881	2.313823307	1.948780179	1.845203826	0.920588701	0.000706705	0.024379323
TCONS_00025329	WBGene00015995	C18H7.5	IV	1	330	1	330	0	4.883563663	0	0.006382257	0.005103181	0.026428716	0	7.73E-20	1.18E-17
TCONS_00037535	WBGene00015996	C18H7.6	IV	829	1641	1	813	3.499476336	19.69697779	4.330412736	0.251742431	2.374163077	4.904521173	0.239514518	0.001539807	0.048422159
TCONS_00037538	WBGene00015998	srt-59	IV	1	933	355	1287	0.007000282	12.2511128	0.013763318	0	0	0	0.04905837	1.46E-10	1.05E-08
TCONS_00037539	WBGene00015999	prmt-4	IV	1	435	1	435	0.028852111	4.920906633	0.018091714	0.027618559	0.034275808	0.02706467	0.032717025	1.24E-07	6.87E-06
TCONS_00014703	WBGene00016003	C18H9.5	II	1	1395	1	1395	1.422560809	0.531416803	1.799359517	2.336890361	6.060454542	6.159152446	7.875774033	0.001413112	0.044849159
TCONS_00023122	WBGene00016016	C23G10.10	III	477	2097	6215430	6213810	40.88559954	35.04115267	32.00136152	47.7176139	9.36682867	26.08283778	11.51805504	0.000825596	0.028086645
TCONS_00023124	WBGene00016017	C23G10.11	III	62	327	6215430	6215165	39.3733641	33.46751336	31.08251399	46.52552728	8.554716391	24.85495607	10.87903089	0.000673765	0.023318124
TCONS_00005191	WBGene00016042	C24A11.5	I	1	390	1	390	0.627752103	0.271309092	1.033464737	0.7426901	0.655742039	0.022845981	0.629588139	3.16E-05	0.001331832
TCONS_00026849	WBGene00016053	C24D10.1	IV	38	1486	1	1449	3.34838415	40.79275917	3.466690879	5.399142635	2.166550908	1.826654198	1.123899191	1.53E-06	7.72E-05
TCONS_00026840	WBGene00016056	C24D10.5	IV	55	732	1	678	0.494839984	22.92414157	0.463782541	2.435105476	0.502484297	0.352243673	0.680117863	4.65E-07	2.45E-05
TCONS_00038914	WBGene00016058	nspd-3	IV	1	332	5163074	5162743	11.407	15.90439749	7.888996941	7.193804992	4.455668777	3.832852545	1.080755497	0.000989961	0.03252254
TCONS_00038915	WBGene00016058	nspd-3	IV	1	331	5163074	5162744	11.407	15.90439749	7.888996941	7.193804992	4.455668777	3.832852545	1.080755497	0.000989961	0.03252254
TCONS_00001135	WBGene00016259	C30F8.3	I	1	1002	1	1002	0.404972947	0.312157332	0.411562159	0.228789038	0.121793689	4.051263127	0.113330087	0.000132803	0.005261056
TCONS_00071499	WBGene00016271	irld-23	X	353	1471	1	1119	0.048623097	0.079546606	0.036183429	0.133055186	0.061620073	1.977473448	0.100475744	3.66E-07	1.96E-05

TCONS_00071498	WBGene00016271	irld-23	X	334	1127	326	1119	0.041622815	0.037342969	0.022420111	0.096106294	0.070379977	2.868777359	0.079450728	1.28E-06	6.47E-05
TCONS_00008924	WBGene00016571	C41H7.1	II	1	753	1	753	1.949458495	0.713707253	0.220431964	0.653256301	0.181967304	2.122195429	0.906469017	2.56E-05	0.00108899
TCONS_00013610	WBGene00016577	clec-3	II	1	1230	1	1230	1.198545855	1.564291929	0.133025157	0.440293514	0.070379977	1.398847799	0.584085442	2.18E-09	1.40E-07
TCONS_00013609	WBGene00016577	clec-3	II	484	2016	3018308	3016776	1.290021626	1.664324979	0.217878702	0.556777565	0.101380966	1.541258955	0.638989817	2.10E-08	1.23E-06
TCONS_00001129	WBGene00016727	C46H11.1	I	1	198	1	198	0.011540844	0	0.013763318	0	0	2.680596578	0.014016677	2.56E-12	2.13E-10
TCONS_00005090	WBGene00016731	C46H11.7	I	128	847	1	720	0.63191379	0.671067345	0.791767788	0.615934967	0.471865213	6.713232414	0.150730784	0.000552362	0.019466969
TCONS_00001609	WBGene00016943	acdh-1	I	16	978	1	964	11.07039323	6.637592204	19.59068766	6.469182918	22.69262316	2.722594403	10.79007667	5.10E-07	2.67E-05
TCONS_00001610	WBGene00016943	acdh-1	I	16	980	1	964	11.07039323	6.637592204	19.59068766	6.469182918	22.69262316	2.722594403	10.79007667	5.10E-07	2.67E-05
TCONS_00001613	WBGene00016943	acdh-1	I	1	948	17	964	11.07039323	6.637592204	19.59068766	6.469182918	22.69262316	2.722594403	10.79007667	5.10E-07	2.67E-05
TCONS_00001612	WBGene00016943	acdh-1	I	14	974	1	964	11.02508084	6.625534022	19.52953086	6.457276949	22.62917473	2.717181469	10.7421463	5.26E-07	2.75E-05
TCONS_00001607	WBGene00016943	acdh-1	I	332	1614	1	1284	11.93747206	7.055811676	20.74899513	6.994907744	24.13774943	2.853063109	11.42137651	5.44E-07	2.83E-05
TCONS_00001608	WBGene00016943	acdh-1	I	332	1616	1	1284	11.93747206	7.055811676	20.74899513	6.994907744	24.13774943	2.853063109	11.42137651	5.44E-07	2.83E-05
TCONS_00001611	WBGene00016943	acdh-1	I	16	1299	1	1284	11.93747206	7.047383414	20.7171401	6.994907744	24.12388635	2.853063109	11.40735983	5.52E-07	2.87E-05
TCONS_00001614	WBGene00016943	acdh-1	I	36	794	1	759	9.671386219	5.577407067	16.62851003	5.391863253	19.12289325	2.339145035	9.095044309	5.09E-07	2.67E-05
TCONS_00001615	WBGene00016943	acdh-1	I	36	794	1	759	10.53269463	5.978770015	17.42987086	5.724258167	20.20714014	2.469613741	9.700635465	5.71E-07	2.96E-05
TCONS_00057500	WBGene00017205	F07C4.12	V	1	1089	1	1089	3.517230017	3.070318173	1.66521585	1.149127918	0.314349259	0.822222321	0.358621938	0.000369437	0.013386888
TCONS_00062247	WBGene00017222	F07G6.2	X	1	729	1	729	0	0.034943798	0	0.028477104	0.630770362	0.006607179	0.030392358	0.000132447	0.005252455
TCONS_00013421	WBGene00017528	F16G10.14	II	1	663	1	663	0.849481927	0	0.005605028	0.016571136	0.325782495	0.018627292	0	0.000486494	0.017338548
TCONS_00051433	WBGene00017678	asp-12	V	3	1190	1	1188	2.126931196	8.12201016	3.753994523	16.10145041	2.131267809	12.89506532	3.058549073	3.14E-08	1.81E-06
TCONS_00051432	WBGene00017678	asp-12	V	49	1236	1	1188	2.305342175	9.219039669	4.718837408	20.6515499	2.32305957	16.17100698	3.24900522	8.57E-09	5.15E-07
TCONS_00051431	WBGene00017678	asp-12	V	89	1276	1	1188	2.305342175	9.22506876	4.71323238	20.6515499	2.298990124	16.16559405	3.261859564	8.21E-09	4.94E-07
TCONS_00051430	WBGene00017678	asp-12	V	101	1288	1	1188	2.298341894	9.078158379	4.689535638	20.61202537	2.271645859	16.16917678	3.17307583	6.98E-09	4.26E-07
TCONS_00051429	WBGene00017678	asp-12	V	164	1351	1	1188	2.298341894	9.134757045	4.689535638	20.61202537	2.271645859	16.17458971	3.180084168	6.71E-09	4.11E-07
TCONS_00057685	WBGene00017678	asp-12	V	1	1503	8272147	8270645	1.695472622	6.42225853	2.817954906	10.05920385	1.758654457	7.783658415	2.665807428	1.37E-06	6.91E-05
TCONS_00068607	WBGene00017691	ilys-5	X	64	483	1	420	24.94398775	63.20778367	5.852803166	7.082542249	0.727406152	1.226051515	18.59271771	0.000471044	0.016846264
TCONS_00068608	WBGene00017691	ilys-5	X	57	645	6536112	6535524	9.163210188	24.83843436	2.346220469	2.779645849	0.299722365	0.45859449	7.398270987	0.000943135	0.031339626
TCONS_00001541	WBGene00017815	F26B1.1	I	1	534	1	534	3.205178864	0.501271349	0.383755896	0.367254275	0.372995257	5.847508643	0.181088806	0.000292331	0.010870369
TCONS_00026932	WBGene00018118	F36H12.2	IV	127	1185	1	1059	5.283923437	58.6625137	2.871877189	41.7146046	1.860579649	1.695224238	2.05577295	6.05E-12	4.95E-10
TCONS_00026930	WBGene00018118	F36H12.2	IV	239	1273	25	1059	5.272382592	58.6625137	2.871877189	41.7146046	1.860579649	1.695224238	2.05577295	6.05E-12	4.95E-10
TCONS_00026929	WBGene00018119	F36H12.3	IV	1	1008	1	1008	6.454001995	43.07968521	5.101705324	29.94440187	3.500032589	2.829474226	1.628671248	6.50E-10	4.36E-08
TCONS_00026924	WBGene00018120	F36H12.4	IV	55	261	1	207	2.759019575	30.04549386	1.738518958	21.44742409	1.543093994	1.109496143	0.462687689	6.28E-15	6.03E-13
TCONS_00026925	WBGene00018120	F36H12.4	IV	1	251	5269113	5269363	2.395482975	20.49971551	1.551996787	14.73476962	1.370951146	1.031326577	0.427645996	2.87E-13	2.54E-11
TCONS_00026922	WBGene00018121	F36H12.5	IV	1	594	1	594	3.378168601	32.08477516	2.900619704	22.30021598	1.553844581	1.638706409	0.669382174	1.04E-13	9.44E-12
TCONS_00026914	WBGene00018122	ttbk-2	IV	42	977	1	936	19.62145882	60.7820895	5.230365469	51.11262938	3.524703522	2.922858319	1.357430361	2.44E-13	2.17E-11
TCONS_00026915	WBGene00018122	ttbk-2	IV	42	977	1	936	19.47804925	60.68334952	5.12004005	49.88587926	3.449602269	2.840470064	1.324713336	2.25E-13	2.01E-11
TCONS_00026913	WBGene00018123	F36H12.9	IV	1	1143	1	1143	26.27665003	31.7783353	4.391301567	27.62924104	4.249460242	3.750260581	2.057204893	7.26E-09	4.42E-07

TCONS_00038985	WBGene00018124	F36H12.10	IV	1	1197	1	1197	30.20548034	35.51147953	7.927149417	30.1512483	6.18224823	5.482506411	2.843438492	5.87E-07	3.04E-05
TCONS_00038986	WBGene00018125	rmd-4	IV	1	648	1	648	37.48412121	49.9190873	3.197309172	41.39568763	2.328162751	1.854432486	0.850505315	6.16E-15	5.92E-13
TCONS_00038987	WBGene00018126	F36H12.13	IV	1	224	5263282	5263059	1.631706397	28.79876511	0.742538418	20.70530229	0.597315624	0.508582804	0.179892136	2.13E-21	4.20E-19
TCONS_00038989	WBGene00018127	F36H12.14	IV	34	852	1	819	4.155473633	49.17942543	2.353102128	35.66808461	2.195341631	2.044210475	0.656493495	2.28E-17	3.01E-15
TCONS_00038988	WBGene00018127	F36H12.14	IV	262	1080	1	819	5.033807671	68.2287785	2.703726359	49.29053008	2.455002359	2.195136672	0.719568543	1.39E-18	1.96E-16
TCONS_00038992	WBGene00018128	F36H12.15	IV	167	515	5268211	5267862	1.163544446	28.79039917	0.632431873	20.17196422	0.588254977	0.367924184	0.198592484	1.30E-19	1.96E-17
TCONS_00038996	WBGene00018129	F36H12.16	IV	1	372	1	372	0.827630098	19.16027587	0.418662693	13.4534136	0.390596226	0.2489173	0.163550791	9.50E-19	1.36E-16
TCONS_00038991	WBGene00018130	F36H12.17	IV	267	826	1	560	8.098647746	105.1224143	2.028168646	74.26117299	1.499619116	1.06174099	0.740730901	2.74E-19	4.09E-17
TCONS_00038997	WBGene00018130	F36H12.17	IV	267	1031	1	765	5.67431266	47.56573235	0.743656927	33.8784649	0.38000796	0.299386242	0.237189849	2.99E-21	4.94E-19
TCONS_00056120	WBGene00018138	folt-2	V	123	1397	1	1275	1.014656033	2.303642173	1.828102032	1.643995871	3.864731007	0.308862537	2.532207707	0.000999634	0.032683837
TCONS_00023740	WBGene00018410	F44B9.9	III	1	735	1	735	0.106706194	0.118120323	0.364886052	0.142871623	2.568394149	0.155067223	0.143688109	7.99E-06	0.000360639
TCONS_00008364	WBGene00018449	F45D11.2	II	1	389	1007792	1008180	0.219182811	0.036174546	0.40717301	0.348593607	0.79117923	0.961439062	13.37042584	6.93E-11	5.13E-09
TCONS_00008361	WBGene00018452	F45D11.5	II	1	441	1001735	1002175	0.736725609	0.415695652	3.202306673	4.976361006	6.359694917	1.288773562	7.401757989	4.03E-07	2.14E-05
TCONS_00008360	WBGene00018454	fbxb-28	II	1	279	993033	993311	5.338253417	6.910474503	2.238083795	4.449922753	0.582769891	7.962912282	4.251158228	1.11E-06	5.64E-05
TCONS_00008358	WBGene00018455	fbxc-42	II	52	996	1	945	8.264353353	10.74843438	3.601722525	6.753690133	1.034303542	12.0390171	6.609824687	3.04E-06	0.000139377
TCONS_00008359	WBGene00018455	fbxc-42	II	52	996	1	945	10.94024429	14.20305626	4.730448596	9.000130863	1.341721258	16.04300125	8.761693655	2.33E-06	0.000107907
TCONS_00008357	WBGene00018456	fbxc-39	II	159	1289	1	1131	12.30642397	16.250096	8.224386482	11.06993865	3.487396378	19.09235722	11.47457814	0.001377208	0.043810965
TCONS_00051499	WBGene00018477	otpl-4	V	1	1677	1	1677	0.06262366	1.989069926	0.128696761	0.022094847	0.0824147	0.066071789	0.007008339	0.000113228	0.004533981
TCONS_00068067	WBGene00018879	F55D10.4	X	1	924	1	924	0.21095267	1.25509469	0.30372925	0.467394513	0.410627044	0.961220711	0.255821527	0.000281008	0.01046825
TCONS_00062220	WBGene00019146	H02F09.3	X	1	3828	1	3828	0.116166195	2.501184453	0.111103549	0.548160658	0.496316563	1.687112159	1.453531454	0.001039925	0.033893471
TCONS_00062219	WBGene00019146	H02F09.3	X	10	3791	1	3782	0.133477462	2.610876513	0.111103549	0.570255505	0.501419744	1.770694658	1.548195528	0.000898023	0.03032503
TCONS_00062218	WBGene00019146	H02F09.3	X	10	3791	1	3782	0.121936617	2.555446271	0.111103549	0.577496307	0.501419744	1.769500414	1.509632499	0.000767702	0.026290914
TCONS_00022039	WBGene00019150	H04J21.1	III	1	2307	1	2297	0.983816312	0.794048335	0.639872787	0.670654524	0.257832367	0.46098298	0.100475744	0.001492003	0.047062681
TCONS_00022040	WBGene00019150	H04J21.1	III	1	2297	1	2297	0.983816312	0.794048335	0.639872787	0.670654524	0.257832367	0.46098298	0.100475744	0.001492003	0.047062681
TCONS_00027581	WBGene00019152	nspb-6	IV	23	277	1	255	0.728681935	1.673968245	0.279254378	2.41112913	0.194684676	2.536802111	0.965753118	1.62E-10	1.16E-08
TCONS_00027569	WBGene00019154	glf-1	IV	2	1432	1	1431	4.264750598	11.34498702	5.624553291	11.30757696	3.352341092	10.1640832	4.783429496	0.00027408	0.010219423
TCONS_00056379	WBGene00019179	scl-13	V	17	643	1	627	10.22686819	2.136369998	7.000544721	2.839403014	7.862526616	0.716429794	1.282525962	0.000166603	0.006463752
TCONS_00050433	WBGene00019232	ugt-13	V	17	1615	1	1599	0.763024767	1.038654918	0.802479341	2.028452503	0.877689819	1.48627531	0.185841147	0.000124447	0.004963876
TCONS_00050431	WBGene00019234	ugt-8	V	27	1622	1	1596	0.999804485	2.182078905	0.732106492	1.264608305	0.63814107	1.990003471	0.281667555	2.39E-06	0.000110242
TCONS_00055705	WBGene00019243	fbxb-72	V	1	1041	1	1041	12.8929541	0.389242441	0.627884602	5.930231809	0.863525991	3.149380981	0.747979588	0.000155752	0.00607137
TCONS_00039789	WBGene00019297	K02B2.6	IV	1	432	1	432	0	0.03371305	0.006881659	0	0.005103181	3.441145956	0	7.50E-16	8.39E-14
TCONS_00021646	WBGene00019331	dos-3	III	86	951	1	866	0.041622815	0.057829413	0.023696742	0.131338096	0.070379977	0.564076359	0.074767057	0.000160512	0.006239213
TCONS_00057851	WBGene00019442	K06C4.1	V	100	882	16	798	0.586694631	1.218966725	0.202838752	0.702452146	5.532917407	0.568047414	5.47628347	0.000263908	0.009850158
TCONS_00068571	WBGene00019619	asp-14	X	1	1233	1	1233	17.06682396	43.20789029	8.635352772	19.60811234	4.751457574	10.35698239	2.31579745	1.49E-09	9.69E-08
TCONS_00013031	WBGene00019702	fbxb-39	II	1	813	1	813	0.716581687	0.383088701	1.616047232	0.122493866	0.200250922	0.11303566	7.24200322	1.20E-10	8.77E-09
TCONS_00013032	WBGene00019703	fbxb-41	II	1	894	1	894	1.641178276	1.006234941	2.613146282	1.253333558	1.68325246	1.782156481	8.593180625	0.000195108	0.007450312

TCONS_00013033	WBGene00019705	fbxb-45	II	1	1008	1	1008	2.489622751	1.78901957	4.011497071	1.792536324	3.030377643	1.679233071	10.28571664	3.89E-05	0.001627167
TCONS_00049426	WBGene00019744	M02H5.8	V	1	378	1	378	5.538952701	18.29730891	3.07120229	4.797776521	3.241632097	2.731560788	1.839882802	0.000902702	0.030418199
TCONS_00028661	WBGene00019899	R05G6.5	IV	1	567	1	567	0.316807879	0.345870382	0.277260371	1.717085859	0.39340798	0.307187665	0.11688576	0.000910692	0.030652276
TCONS_00050193	WBGene00019964	R08E5.4	V	16	402	1	387	0.386525055	0.107292889	0.483929017	0.513818858	0.950661967	0.413057856	1.135556865	0.000173379	0.006676304
TCONS_00056378	WBGene00019968	R08F11.4	V	24	1088	1	1065	0.562004207	5.099582256	0.535152395	1.17036422	0.861697629	1.319343303	0.942094415	0.000519251	0.01841036
TCONS_00055654	WBGene00020016	lipl-3	V	6	1220	1	1215	28.76197393	12.01036838	16.05053556	28.41935114	3.122836383	9.032983702	3.365782898	0.000333573	0.012238158
TCONS_00055653	WBGene00020016	lipl-3	V	78	1292	1	1215	28.76197393	12.01639747	16.05053556	28.41935114	3.122836383	9.032983702	3.365782898	0.000332706	0.01222815
TCONS_00038798	WBGene00020071	R13H9.5	IV	54	989	1	936	6.785189313	5.039400251	4.315068199	14.5834912	2.879167845	2.487357445	1.286150306	0.001416735	0.044929483
TCONS_00013334	WBGene00020076	R52.4	II	1	507	1	507	0.553960534	0.133808424	0	0.231995895	0.124767764	0.129910416	0	6.40E-07	3.30E-05
TCONS_00013335	WBGene00020077	R52.5	II	1	552	1	552	0.565501378	0.151895697	0.011210055	0.231995895	0.124767764	0.129910416	0.014016677	1.07E-05	0.000480724
TCONS_00010381	WBGene00020319	npr-31	II	13	1239	1	1227	1.387460227	0.275920462	1.409341969	0.286974233	1.645238768	1.144920527	0.816557285	0.000231757	0.008784567
TCONS_00008962	WBGene00020406	T10D4.1	II	1	1134	1	1134	0.005770422	0	0.013763318	0.469515502	0.022241085	2.562720917	0.01169201	2.22E-17	2.94E-15
TCONS_00013647	WBGene00020410	T10D4.11	II	536	881	392	737	0.014000563	0.008428262	0	0.397707249	0	2.189306134	0	6.87E-26	1.81E-23
TCONS_00063605	WBGene00020420	T10E10.3	X	28	1209	1	1182	88.96489955	30.28759759	11.24418314	5.732173815	3.521657749	0.433685317	0.141363442	0.00087062	0.029454494
TCONS_00050095	WBGene00020619	T20D4.13	V	429	1109	1	681	4.289341848	0.525699335	1.921211235	0.50150899	3.61443167	0.277098551	0.217430174	7.84E-05	0.003195788
TCONS_00025254	WBGene00020650	T21D12.11	IV	1	2130	1	2130	0.072083661	4.037029435	0.272433472	0.201542921	0.103891976	0.14838238	0.072442389	2.00E-06	9.58E-05
TCONS_00056332	WBGene00020700	T22F3.11	V	37	1160	1	1124	145.1589501	129.9816414	121.581968	96.69373728	11.0479331	71.26730218	26.42314819	4.62E-05	0.001922008
TCONS_00056331	WBGene00020700	T22F3.11	V	37	1398	1	1362	180.5399932	154.9537451	144.1654491	115.8645356	13.37928951	86.91436224	29.72650334	2.81E-05	0.001195878
TCONS_00001678	WBGene00020711	rmd-5	I	1	771	1	771	0.037082252	0.057829413	0.12742013	6.68160173	4.386520649	0.194710296	9.275216205	0.000182739	0.006990947
TCONS_00001676	WBGene00020711	rmd-5	I	1	1329	6708991	6710319	0.06262366	0.112028907	0.412340289	6.809619308	4.541143688	0.552925193	9.55442175	5.45E-05	0.002248986
TCONS_00001677	WBGene00020711	rmd-5	I	1	1329	6708991	6710319	0.068394082	0.112028907	0.412340289	6.81514302	4.541143688	0.558338127	9.55442175	5.62E-05	0.002314861
TCONS_00057701	WBGene00020946	W02D7.11	V	1	252	1	252	6.047793249	0.167583798	1.201494061	2.877645808	3.778855266	1.821784912	5.980883848	2.98E-05	0.001262392
TCONS_00026866	WBGene00020992	W03F8.2	IV	1	1347	1	1347	0.498057454	0.854089946	0.765238156	4.266181955	0.401785979	0.382177459	0.265360546	0.000994505	0.032590524
TCONS_00026867	WBGene00020992	W03F8.2	IV	710	1494	5191384	5192168	0.498057454	0.854089946	0.765238156	4.266181955	0.401785979	0.388784638	0.265360546	0.000918339	0.030859193
TCONS_00038942	WBGene00020997	gpa-18	IV	29	985	1	966	0.095544225	7.736225756	0.272652347	4.736865529	0.247926749	0.214609497	0.268710205	2.59E-12	2.14E-10
TCONS_00038943	WBGene00020997	gpa-18	IV	29	994	1	966	0.095544225	7.736225756	0.272652347	4.736865529	0.247926749	0.214609497	0.268710205	2.59E-12	2.14E-10
TCONS_00005072	WBGene00021017	W03G9.7	I	58	1704	1	1647	0.206790982	0.325321612	0.106775153	0.487658609	0.448559575	11.33843651	0.462584683	1.33E-06	6.73E-05
TCONS_00004181	WBGene00021028	W04C9.6	I	1	1980	1	1980	0.377350696	0.861349785	0.337420169	0.449851172	0.21441475	0.523472034	0.160098125	0.000361632	0.013173642
TCONS_00038384	WBGene00021283	Y24D9A.5	IV	1	849	1	849	0.927627591	0.051800323	1.068809284	0.543526948	1.086481062	1.25375214	1.027253805	0.000936014	0.031325265
TCONS_00013653	WBGene00021297	fbxc-55	II	74	1130	41	1098	0.848252068	0.226581635	0.227033996	0.47772851	0.381836322	1.21879374	0	0.000350781	0.012846588
TCONS_00055949	WBGene00021491	comt-4	V	1	684	1	684	2.194474294	0.086806445	0.253782503	0.864356878	2.143684437	0.030647405	0.197498821	5.13E-05	0.002120333
TCONS_00049927	WBGene00021568	srt-2	V	1	1041	1	1041	0	0	0	0.022094847	0	0.026428716	7.428838907	1.24E-12	1.05E-10
TCONS_00049926	WBGene00021569	Y45G12C.6	V	1	825	1	825	1.927321024	0	0.099394992	0.033142271	0.04120735	0.030647405	11.14325836	1.90E-16	2.20E-14
TCONS_00056041	WBGene00021573	Y45G12C.10	V	1	1020	1	1020	13.5711821	8.106820003	0.041289954	0	0.005103181	8.718529351	0.079450728	2.77E-32	1.10E-29
TCONS_00056042	WBGene00021574	Y45G12C.11	V	1	2340	1	2340	0.089394928	0.17348824	0.347353592	0.31593744	0.382218226	0.284822311	9.620330232	3.63E-06	0.000166045
TCONS_00049723	WBGene00021599	srt-42	V	1084	3156	1805465	1807537	228.829975	6.883009007	11.68119222	46.25161196	69.13893025	60.02721008	272.248262	0.001112097	0.03593279

TCONS_00049724	WBGene00021599	srt-42	V	1084	3159	1805465	1807541	228.9177612	7.026414117	11.81521438	46.37828489	69.27420512	60.3950566	272.3674724	0.001194701	0.038390857
TCONS_00049725	WBGene00021599	srt-42	V	1084	3159	1805465	1807541	228.9667632	7.149270458	11.83202946	46.44039036	69.33437873	60.70511338	272.4761532	0.001319494	0.0421552
TCONS_00049726	WBGene00021599	srt-42	V	1084	3159	1805465	1807541	228.9667632	7.149270458	11.83202946	46.44039036	69.33437873	60.71172056	272.4761532	0.001320038	0.0421552
TCONS_00055902	WBGene00021601	Y46H3A.5	V	1	1089	1	1089	1.062993489	0.032544626	0.248457103	0.915042491	1.353755981	0.840368986	4.044841434	1.58E-05	0.00068559
TCONS_00055903	WBGene00021601	Y46H3A.5	V	777	1708	1812566	1811635	0.999518844	0.034943798	0.232918651	0.869994251	1.223584293	0.862579012	3.721192518	5.66E-05	0.002331455
TCONS_00038975	WBGene00021995	Y59E9AL.5	IV	1	300	1	300	14.51756877	19.90682624	8.034994806	16.45321645	9.070037557	12.81334804	0.907190064	4.34E-07	2.29E-05
TCONS_00038978	WBGene00021996	Y59E9AL.6	IV	62	706	1	645	32.48204346	37.12148427	19.01694504	31.12768908	19.33924142	24.16515997	3.621575164	7.81E-05	0.003186732
TCONS_00026911	WBGene00021997	Y59E9AR.1	IV	1	393	1	393	19.2793257	20.62322724	8.111933071	16.08487927	6.117549022	5.542762304	3.314365525	0.000921654	0.03092009
TCONS_00026909	WBGene00021998	Y59E9AR.2	IV	550	1701	1	1152	24.58203433	73.67220526	3.017887535	50.0735525	2.646412216	2.080829104	1.210289586	3.03E-15	2.95E-13
TCONS_00038984	WBGene00022003	Y59E9AR.8	IV	1	1152	1	1152	23.42217946	69.72174937	2.909835751	49.21566009	2.460869349	2.034578852	1.196272909	2.61E-15	2.54E-13
TCONS_00017675	WBGene00022481	srz-9	III	1	846	1	846	0.625105916	0.083114201	0.065485198	1.268724454	0.062001977	1.033792731	0.084100063	5.33E-11	3.99E-09
TCONS_00057809	WBGene00022535	ZC178.1	V	1	3238	8702401	8699164	0.345566757	1.177012387	0.791147781	1.620815156	0.539051531	1.264097382	0.404261977	0.000559458	0.019666536
TCONS_00057808	WBGene00022535	ZC178.1	V	1	3130	8702293	8699164	0.344336897	1.185440649	0.794977675	1.633579669	0.540879893	1.268316071	0.403099644	0.000525165	0.018604027
TCONS_00023818	WBGene00022582	ZC262.5	III	1	165	1	165	67.9298309	25.758862	22.46380652	83.18682352	30.44534253	58.43424465	13.53698681	0.000483005	0.017229116
TCONS_00063156	WBGene00022616	hsd-3	X	205	1284	1	1080	0.025541408	0.024116364	0.061156802	0.011047424	0.025897808	0.726789678	0	0.00145966	0.046113168
TCONS_00014439	WBGene00022652	ZK84.5	II	1	269	6016524	6016256	0.755074328	0.69211471	0.446687831	0.605146323	0.2723781	0.325970285	0.007008339	6.53E-05	0.002676564
TCONS_00005468	WBGene00022754	nspd-1	I	1	342	6097837	6097496	11.23993746	9.743852017	6.047970473	6.859755902	4.454742646	4.146802718	0.78756761	0.00087005	0.029454494
TCONS_00067688	WBGene00022824	ZK813.5	X	36	1451	1	1416	0.11455746	1.741207371	4.251309037	0.440520838	0.439035862	0.407489594	12.73816967	3.15E-06	0.000143895
TCONS_00067700	WBGene00022922	C12D12.7	X	1	896	1	896	0.023081689	0.14691038	0.357846271	0.060388388	0.030619084	0.006607179	1.169166615	5.28E-06	0.000239847
TCONS_00026917	WBGene00023030	F36H12.18	IV	29	138	5261183	5261291	0.963567278	19.42906115	0.909692243	14.05941847	0.653531773	0.514709357	0.140166772	3.26E-19	4.84E-17
TCONS_00025362	WBGene00023038	F41A4.t1	X	1	72	1298578	1298507	3.066123387	2.447810923	0	0	0	0	0	5.22E-18	7.03E-16
TCONS_00026846	WBGene00023243	C24D10.3	IV	1	110	5162223	5162332	0	29.79150841	0	2.552902718	0.018966266	0	0	2.16E-41	2.12E-38
TCONS_00013035	WBGene00023315	K02E7.8	II	3	531	456	984	2.573608314	5.468773873	11.32647011	4.61731974	9.493210189	0.046886207	11.04852173	7.75E-09	4.68E-07
TCONS_00040002	WBGene00023326	M03D4.5	IV	1	1032	6142454	6141423	0.007000282	0.016856525	0.006881659	0.011047424	0.804058923	0.005412934	0	4.96E-07	2.61E-05
TCONS_00007954	WBGene00043308	F39B2.9	I	15	425	1	411	0.150788728	0.254203269	0.411562159	1.53016966	0.298657812	1.157265939	0.237155514	0.000477593	0.017050845
TCONS_00020211	WBGene00043993	T26G10.6	III	147	335	9413719	9413907	0	0	1.71513846	0.025529027	0.006931542	0.017433047	0.007008339	1.52E-06	7.68E-05
TCONS_00054667	WBGene00044095	srh-121	V	1	586	1	579	5.380516992	3.421532074	2.831328747	0	3.725126228	0.357253644	0.577145774	0.000133989	0.005288139
TCONS_00065459	WBGene00044140	F42F12.11	X	1	549	1	549	8.046941988	38.50400205	4.082514888	151.7640021	3.496748308	4.382608152	4.647573779	3.44E-11	2.61E-09
TCONS_00018341	WBGene00044230	C36E8.6	III	1	276	1	276	6.207424974	5.124727301	0	5.353854906	2.661712296	5.220307276	3.443453252	2.10E-07	1.16E-05
TCONS_00003383	WBGene00044242	C54C8.12	I	1	298	12456918	12457215	0.218425061	0.301454547	0.375597605	2.971725485	1.120269905	4.989700879	0.727807887	0.000405518	0.014617111
TCONS_00038976	WBGene00044346	Y59E9AL.8	IV	1	906	1	906	14.65473581	19.84536691	8.051809889	16.41369192	9.105759822	12.94015635	0.962128774	5.62E-07	2.92E-05
TCONS_00013803	WBGene00044379	F40H7.12	II	1	744	1	744	0.102165632	0.065089253	0.172260351	0.178589529	0.377415789	0.344519913	1.168205219	0.00046804	0.016753399
TCONS_00070321	WBGene00044398	nspc-11	X	1	219	12353768	12353550	1.639470368	11.2024715	1.408101954	30.40128285	1.26539313	21.71265716	15.12023807	3.19E-11	2.44E-09
TCONS_00025333	WBGene00044457	C18H7.11	IV	15	871	1	872	0.136788165	15.33277274	0.568782561	0.72577798	0.495853498	0.717546375	0.685895197	0.000133101	0.005261056
TCONS_00025359	WBGene00044458	K11H12.10	IV	1	456	676075	676530	6.132246774	4.924536552	0.011210055	0	0.055070435	0.064319254	0.01169201	5.44E-08	3.09E-06
TCONS_00038898	WBGene00044496	C24D10.9	IV	1	294	5152485	5152192	0.026771267	7.475868747	0.024973373	0.74071423	0.034275808	0.049274696	0.06075038	1.82E-16	2.12E-14

TCONS_00038983	WBGene00044497	Y59E9AR.9	IV	1	270	1	270	2.319709736	7.821427505	0.064986696	1.703878865	0.010206361	0.013214358	0.014016677	7.34E-27	2.06E-24
TCONS_00026910	WBGene00044498	Y59E9AR.10	IV	1	270	1	270	5.859158579	19.15627201	0.026250005	13.51111269	0.010206361	0.013214358	0.014016677	9.69E-43	9.97E-40
TCONS_00067211	WBGene00044560	C36C9.6	X	1	390	1	390	0.422849557	0.543101038	0.466895059	0.160301303	0.677381636	0.076339366	0.561863757	0.000260745	0.009774489
TCONS_00062693	WBGene00044564	ZK813.6	X	754	1803	3427179	3428228	0.887514337	1.724849443	3.811916478	1.496976641	1.987094614	1.479978787	11.39658473	7.31E-05	0.002983955
TCONS_00062695	WBGene00044564	ZK813.6	X	1	756	1	756	0.054393519	1.044185412	2.498978488	0.566335222	0.313967354	0.394430564	8.760007329	0.000211928	0.008070112
TCONS_00053948	WBGene00044658	C01G10.17	V	32	259	1	228	0.115408445	1.361810914	0.107273655	0.17209361	0.122476337	0.313314218	0.012854343	7.92E-09	4.77E-07
TCONS_00013648	WBGene00044889	T10D4.14	II	1	360	1	360	0	0	0.005605028	0.198853624	0	1.091349478	0	2.99E-17	3.91E-15
TCONS_00054712	WBGene00044960	fbxa-173	V	1	653	17562221	17562874	0.910024743	0.096465455	0.633611135	0.195160664	0.096277785	0.070926432	0.657621494	0.000153253	0.005985315
TCONS_00054711	WBGene00044960	fbxa-173	V	86	604	17562355	17562874	1.400889508	0.156756365	0.972587562	0.318026972	0.158279763	0.112399705	1.001030085	0.000127401	0.005071903
TCONS_00042139	WBGene00045122	F22B3.10	IV	1	966	1	966	0.03131183	0.024116364	0	0.016571135	0.012034723	35.69500479	0	1.36E-26	3.79E-24
TCONS_00037442	WBGene00045177	T21D12.14	IV	187	294	287561	287454	1.626792899	4.053512013	1.007810603	1.065786138	1.136968643	1.032132502	0.28163322	0.00076579	0.026256398
TCONS_00029527	WBGene00045180	K07F5.17	IV	1	396	1	396	4.937075161	4.300860227	3.521793248	0.112191326	4.108619127	0.170034116	4.38021162	3.18E-12	2.63E-10
TCONS_00029528	WBGene00045180	K07F5.17	IV	51	282	9842808	9843039	2.509119973	2.17752986	1.818861848	0.084572767	2.066153335	0.094330704	2.193609979	1.84E-10	1.31E-08
TCONS_00071359	WBGene00045272	F59C12.4	X	1	273	16602717	16602445	0.201113794	0.606289716	0.392631563	0.875549421	0.628918101	3.30731732	0.584154113	0.001504059	0.047406645
TCONS_00051145	WBGene00045275	F18E3.12	V	1	404	7414941	7415344	6.376136033	0.886883871	2.600720348	0.381622217	10.62977303	0.53262303	1.14448292	3.38E-09	2.13E-07
TCONS_00051146	WBGene00045276	F18E3.13	V	1	499	7416116	7416614	19.64992612	3.802175024	8.73779953	6.053572053	36.04411481	4.353795402	3.67248258	0.001587253	0.04983805
TCONS_00048128	WBGene00045281	Y51H4A.33	IV	1	140	16534576	16534437	0	0	0.487637405	0	0	0.633313279	1.403041141	2.07E-06	9.58E-05
TCONS_00013654	WBGene00045303	Y27F2A.10	II	1	765	1	765	0.565501378	0	0	0.132569083	0	0.727566318	0	1.62E-16	1.90E-14
TCONS_00038822	WBGene00045532	21ur-116		NA	NA	NA	NA	2.145466383	8.042104699	0.961754503	6.66221547	0.787279024	0.590815731	0.682476867	2.27E-07	1.23E-05
TCONS_00048630	WBGene00045546	21ur-3313		NA	NA	NA	NA	0.005770422	0	0	0.011047424	0.012034723	0.005412934	1.415895484	3.01E-11	2.32E-09
TCONS_00036804	WBGene00045581	21ur-3683		NA	NA	NA	NA	0	0.006029091	0	0.011047424	0	0.005412934	1.408887146	3.40E-09	2.13E-07
TCONS_00039006	WBGene00045595	21ur-676		NA	NA	NA	NA	1.292574579	9.431225623	0.068816591	6.740759131	0	0	0	6.27E-38	3.27E-35
TCONS_00039712	WBGene00045649	21ur-198		NA	NA	NA	NA	0.291645347	1.177806863	0.345797333	1.498176171	0.385793789	1.636487889	1.028725159	9.64E-05	0.003880812
TCONS_00027924	WBGene00045688	21ur-4041		NA	NA	NA	NA	0	0.825985459	0.728653594	0	0.556246697	0	0.007008339	0.000583541	0.02037395
TCONS_00026771	WBGene00045728	21ur-2397		NA	NA	NA	NA	0.4154704	0.332332153	0.005605028	1.902297134	0.006931542	0.019821537	0.017538014	4.87E-17	5.86E-15
TCONS_00036412	WBGene00045747	21ur-4706		NA	NA	NA	NA	0	0	0.494519064	0	0	0.633313279	1.403041141	1.95E-06	9.49E-05
TCONS_00038998	WBGene00045748	21ur-4832		NA	NA	NA	NA	1.313575424	9.45651041	0.089461568	6.762853979	0.03282935	0.02706467	0.021025016	3.21E-30	1.14E-27
TCONS_00036366	WBGene00045750	21ur-3704		NA	NA	NA	NA	0	0	0.494519064	0	0	0.633313279	1.410049479	1.86E-06	9.22E-05
TCONS_00026769	WBGene00045755	21ur-506		NA	NA	NA	NA	0.4154704	0.321504719	0.026250005	1.940590674	0.025897808	0.030647405	0.01169201	5.80E-15	5.59E-13
TCONS_00048225	WBGene00045765	21ur-4567		NA	NA	NA	NA	0	0	0.487637405	0	0	0.633313279	1.403041141	2.07E-06	9.58E-05
TCONS_00036800	WBGene00045796	21ur-3106		NA	NA	NA	NA	0	0	0	0.011047424	0.012034723	0.005412934	1.422903823	2.88E-11	2.23E-09
TCONS_00026878	WBGene00045812	21ur-3808		NA	NA	NA	NA	0	0	0	0.63822568	0	0	0.007008339	1.44E-09	9.42E-08
TCONS_00026842	WBGene00045824	21ur-4351		NA	NA	NA	NA	0	7.442155697	0	0.63822568	0	0	0	1.02E-33	4.08E-31
TCONS_00027582	WBGene00045855	21ur-4682		NA	NA	NA	NA	0	0.707974041	0	1.160516326	0	1.254868721	0.473526385	3.51E-17	4.24E-15
TCONS_00039678	WBGene00045872	21ur-906		NA	NA	NA	NA	0	0.707974041	0	1.160516326	0	1.254868721	0.473526385	3.51E-17	4.24E-15
TCONS_00026833	WBGene00045876	21ur-4409		NA	NA	NA	NA	0.044082534	7.448184788	0.005605028	0.662037617	0	0.010825868	0.007008339	2.71E-30	9.78E-28

TCONS_00039994	WBGene00045928	21ur-5209	NA	NA	NA	NA	0	0	0	0.011047424	0.776332753	0.006607179	0	7.37E-09	4.46E-07
TCONS_00036418	WBGene00045978	21ur-463	NA	NA	NA	NA	0	0	0.487637405	0	0	0	1.403041141	7.09E-24	1.59E-21
TCONS_00048622	WBGene00045996	21ur-2495	NA	NA	NA	NA	0	0	0	0	0	0	1.403041141	1.86E-15	1.81E-13
TCONS_00038927	WBGene00046014	21ur-2448	NA	NA	NA	NA	0.425029575	8.004761729	0.649307709	1.089907603	0.575155701	0.528249013	0.693006543	0.000823401	0.028035134
TCONS_00036342	WBGene00046033	21ur-4741	NA	NA	NA	NA	0	0.006029091	0	0	0	0	1.403041141	6.02E-13	5.19E-11
TCONS_00026871	WBGene00046036	21ur-4419	NA	NA	NA	NA	0	0	0	0.63822568	0	0	0	9.75E-12	7.70E-10
TCONS_00048628	WBGene00046106	21ur-480	NA	NA	NA	NA	0	0	0.006881659	0	0	0.012020113	1.421741489	2.38E-13	2.12E-11
TCONS_00048235	WBGene00046116	21ur-2746	NA	NA	NA	NA	0	0	0	0	0	0.006607179	1.403041141	6.51E-14	5.95E-12
TCONS_00036807	WBGene00046136	21ur-888	NA	NA	NA	NA	0	0	0	0.016571135	0	0.005412934	1.408887146	2.46E-09	1.57E-07
TCONS_00036427	WBGene00046165	21ur-410	NA	NA	NA	NA	0	0	0	0	0	0	1.403041141	1.86E-15	1.81E-13
TCONS_00039710	WBGene00046192	21ur-3682	NA	NA	NA	NA	0	0.707974041	0.006881659	1.160516326	0	1.254868721	0.473526385	1.01E-16	1.20E-14
TCONS_00027822	WBGene00046212	21ur-2987	NA	NA	NA	NA	0.005770422	0.910392732	0.773493815	0.797782099	0.006931542	1.57813986	0.652359194	1.29E-05	0.00057414
TCONS_00038831	WBGene00046213	21ur-4622	NA	NA	NA	NA	1.604177379	7.425299173	0	5.925276404	0	0	0	1.58E-44	2.44E-41
TCONS_00036819	WBGene00046236	21ur-4426	NA	NA	NA	NA	0.005770422	0.008428262	0	0.011047424	0	0.005412934	1.408887146	3.44E-09	2.15E-07
TCONS_00027931	WBGene00046257	21ur-4577	NA	NA	NA	NA	0.005770422	0.008428262	0.74802194	0.01742968	0.031000989	0	0.005846005	2.74E-08	1.59E-06
TCONS_00027949	WBGene00046267	21ur-297	NA	NA	NA	NA	0	0.825985459	0.728653594	0	0.556246697	0	0	0.000952431	0.031339626
TCONS_00038800	WBGene00046270	21ur-2058	NA	NA	NA	NA	0.409699978	0.307047366	0.005605028	1.883150363	0	0.019821537	0	2.52E-22	5.21E-20
TCONS_00039785	WBGene00046294	21ur-2380	NA	NA	NA	NA	0	0	0	0.005523712	0.010206361	0.864346189	0	2.31E-09	1.49E-07
TCONS_00026788	WBGene00046318	21ur-4870	NA	NA	NA	NA	2.157007227	8.050532961	1.00354296	6.693268209	0.811730375	0.614855957	0.726885901	3.91E-07	2.08E-05
TCONS_00048606	WBGene00046334	21ur-4629	NA	NA	NA	NA	0	0.025284787	0.487637405	0.006382257	0	0	1.403041141	1.19E-17	1.59E-15
TCONS_00038947	WBGene00046404	21ur-4955	NA	NA	NA	NA	0	0.026515535	0.011210055	0.663754707	0.032447446	0.005412934	0.01169201	9.72E-06	0.000436242
TCONS_00040050	WBGene00046405	21ur-1198	NA	NA	NA	NA	0	0.825985459	0.728653594	0	0.556246697	0	0.007008339	0.000583541	0.02037395
TCONS_00026820	WBGene00046424	21ur-1084	NA	NA	NA	NA	1.775494844	7.454213879	0	5.948229797	0.005103181	0.006607179	0	4.60E-43	4.98E-40
TCONS_00027583	WBGene00046425	21ur-3843	NA	NA	NA	NA	0	0.707974041	0	1.160516326	0	1.254868721	0.473526385	3.51E-17	4.24E-15
TCONS_00048604	WBGene00046427	21ur-5254	NA	NA	NA	NA	0	0	0.487637405	0.006382257	0	0	1.403041141	2.82E-21	4.67E-19
TCONS_00026785	WBGene00046436	21ur-4588	NA	NA	NA	NA	2.164007509	8.050532961	1.00354296	6.693268209	0.818661917	0.614855957	0.726885901	4.09E-07	2.16E-05
TCONS_00038893	WBGene00046437	21ur-1146	NA	NA	NA	NA	0.007000282	7.467440485	0.005605028	0.680325842	0.017137904	0	0.040887698	2.84E-18	3.90E-16
TCONS_00038883	WBGene00046478	21ur-905	NA	NA	NA	NA	0.005770422	7.442155697	0.005605028	0.63822568	0	0	0.007008339	1.47E-29	4.54E-27
TCONS_00039775	WBGene00046482	21ur-97	NA	NA	NA	NA	0	0.008428262	0	0	0.010206361	0.858933255	0	2.46E-09	1.57E-07
TCONS_00038919	WBGene00046509	21ur-718	NA	NA	NA	NA	0.021000845	7.442155697	0.005605028	0.650131648	0	0.039643073	0.007008339	2.98E-31	1.13E-28
TCONS_00036766	WBGene00046516	21ur-212	NA	NA	NA	NA	0	0	0.487637405	0.006382257	0	0	1.403041141	2.82E-21	4.67E-19
TCONS_00038835	WBGene00046522	21ur-5324	NA	NA	NA	NA	1.604177379	7.425299173	0	5.918894148	0	0	0	1.60E-44	2.44E-41
TCONS_00038850	WBGene00046547	21ur-1688	NA	NA	NA	NA	1.769724421	7.442155697	0	5.936323828	0	0	0	1.50E-44	2.44E-41
TCONS_00039895	WBGene00046559	21ur-4068	NA	NA	NA	NA	0.966038875	0.83201455	0	0.72671873	0.776332753	1.406833835	0.507405744	1.49E-05	0.000650724
TCONS_00036416	WBGene00046598	21ur-2555	NA	NA	NA	NA	0	0	0.487637405	0	0	0	1.403041141	7.09E-24	1.59E-21
TCONS_00038994	WBGene00046604	21ur-2996	NA	NA	NA	NA	0.3635366	9.545778351	0.186522171	6.707130757	0.172142847	0.078169566	0.035041693	7.54E-23	1.62E-20

TCONS_00038948	WBGene00046611	21ur-2081	NA	NA	NA	NA	0.007000282	0.006029091	0	0.63822568	0.025515904	0.012020113	0.019862682	4.72E-06	0.000214771
TCONS_00036810	WBGene00046614	21ur-4150	NA	NA	NA	NA	0	0	0	0.016571135	0	0.005412934	1.408887146	2.46E-09	1.57E-07
TCONS_00038913	WBGene00046656	21ur-1556	NA	NA	NA	NA	0	7.442155697	0	0.63822568	0.005103181	0	0	2.72E-31	1.04E-28
TCONS_00026805	WBGene00046675	21ur-3769	NA	NA	NA	NA	1.781265266	7.442155697	0.012486687	5.946512707	0.010206361	0.022845981	0	9.96E-41	8.54E-38
TCONS_00048127	WBGene00046688	21ur-5193	NA	NA	NA	NA	0	0	0.487637405	0	0	0.633313279	1.403041141	2.07E-06	9.58E-05
TCONS_00048359	WBGene00046698	21ur-328	NA	NA	NA	NA	0	0	0.494519064	0	0	0.633313279	1.410049479	1.86E-06	9.22E-05
TCONS_00039673	WBGene00046707	21ur-1948	NA	NA	NA	NA	0.12647718	0.767034203	0.034906797	1.20470602	0.088964338	1.283127635	0.520260087	4.53E-12	3.73E-10
TCONS_00036811	WBGene00046741	21ur-1887	NA	NA	NA	NA	0	0	0	0.016571135	0	0.005412934	1.408887146	2.46E-09	1.57E-07
TCONS_00027555	WBGene00046794	21ur-4270	NA	NA	NA	NA	0	0.707974041	0	1.160516326	0.005103181	1.254868721	0.480534724	8.61E-17	1.02E-14
TCONS_00038950	WBGene00046802	21ur-4520	NA	NA	NA	NA	0.007000282	0.006029091	0	0.63822568	0.025515904	0.012020113	0.026871021	1.84E-05	0.000798853
TCONS_00036765	WBGene00046827	21ur-3452	NA	NA	NA	NA	0	0	0.487637405	0.006382257	0	0	1.403041141	2.82E-21	4.67E-19
TCONS_00039704	WBGene00046866	21ur-1235	NA	NA	NA	NA	0.184181402	0.974235492	0.362612416	1.340336841	0.191791761	1.292123304	0.50624341	1.61E-06	8.07E-05
TCONS_00039780	WBGene00046895	21ur-4302	NA	NA	NA	NA	0	0	0.005605028	0	0	0.858933255	0.007008339	1.22E-09	8.05E-08
TCONS_00036420	WBGene00046903	21ur-1292	NA	NA	NA	NA	0	0	0.487637405	0	0	0	1.403041141	7.09E-24	1.59E-21
TCONS_00027952	WBGene00046951	21ur-762	NA	NA	NA	NA	0	0.825985459	0.728653594	0	0.556246697	0	0	0.000952431	0.031339626
TCONS_00048648	WBGene00047046	21ur-775	NA	NA	NA	NA	0	0	0	0.011047424	0	0.018627292	1.41473315	1.64E-09	1.06E-07
TCONS_00048612	WBGene00047071	21ur-3973	NA	NA	NA	NA	0	0	0.487637405	0.006382257	0	0	1.403041141	2.82E-21	4.67E-19
TCONS_00048282	WBGene00047091	21ur-1352	NA	NA	NA	NA	0	0	0.487637405	0	0	0.633313279	1.403041141	2.07E-06	9.58E-05
TCONS_00048615	WBGene00047092	21ur-2909	NA	NA	NA	NA	0	0	0.487637405	0.006382257	0	0	1.403041141	2.82E-21	4.67E-19
TCONS_00048601	WBGene00047136	21ur-3656	NA	NA	NA	NA	0	0	0.487637405	0.006382257	0	0	1.403041141	2.82E-21	4.67E-19
TCONS_00048254	WBGene00047178	21ur-702	NA	NA	NA	NA	0	0	0	0	0	0	1.403041141	1.86E-15	1.81E-13
TCONS_00039005	WBGene00047179	21ur-689	NA	NA	NA	NA	1.292574579	9.431225623	0.068816591	6.740759131	0	0	0	6.27E-38	3.27E-35
TCONS_00026792	WBGene00047187	21ur-5430	NA	NA	NA	NA	2.145466383	8.042104699	0.968636162	6.656691758	0.784004205	0.592009976	0.682476867	2.29E-07	1.24E-05
TCONS_00027778	WBGene00047191	21ur-615	NA	NA	NA	NA	0.959038594	0.83201455	0	0.72671873	0.776332753	1.413441014	0.507405744	1.44E-05	0.000639133
TCONS_00038859	WBGene00047233	21ur-4118	NA	NA	NA	NA	2.091072864	7.827830544	0.342745569	6.279393724	0.462040755	0.453739846	0.23137818	2.42E-14	2.29E-12
TCONS_00048258	WBGene00047290	21ur-993	NA	NA	NA	NA	0	0	0	0	0	0	1.403041141	1.86E-15	1.81E-13
TCONS_00048620	WBGene00047297	21ur-3462	NA	NA	NA	NA	0	0	0	0	0	0	1.403041141	1.86E-15	1.81E-13
TCONS_00038956	WBGene00047312	21ur-4445	NA	NA	NA	NA	0	0	0	0.63822568	0	0	0	9.75E-12	7.70E-10
TCONS_00038805	WBGene00047378	21ur-2481	NA	NA	NA	NA	1.616948083	7.433727435	0.011210055	5.937182373	0.006931542	0.012020113	0	4.56E-41	4.26E-38
TCONS_00036417	WBGene00047383	21ur-4493	NA	NA	NA	NA	0	0	0.487637405	0	0	0	1.403041141	7.09E-24	1.59E-21
TCONS_00048124	WBGene00047390	21ur-2959	NA	NA	NA	NA	0	0	0.487637405	0.022094847	0	0.633313279	1.403041141	8.70E-05	0.003512404
TCONS_00048366	WBGene00047403	21ur-4111	NA	NA	NA	NA	0	0	0	0	0	0	1.403041141	1.86E-15	1.81E-13
TCONS_00027560	WBGene00047433	21ur-3567	NA	NA	NA	NA	0	0.707974041	0	1.160516326	0.013863085	1.254868721	0.473526385	1.83E-16	2.12E-14
TCONS_00036783	WBGene00047439	21ur-387	NA	NA	NA	NA	0	0	0.487637405	0.006382257	0	0	1.403041141	2.82E-21	4.67E-19
TCONS_00038834	WBGene00047444	21ur-3953	NA	NA	NA	NA	1.604177379	7.425299173	0	5.918894148	0	0.013214358	0	4.51E-45	2.44E-41
TCONS_00036414	WBGene00047495	21ur-78	NA	NA	NA	NA	0	0	0.494519064	0	0	0.633313279	1.403041141	1.95E-06	9.49E-05

TCONS_00027573	WBGene00047536	21ur-2797	NA	NA	NA	NA	0	0.707974041	0	1.160516326	0	1.260281655	0.473526385	3.32E-17	4.24E-15
TCONS_00039674	WBGene00047555	21ur-3585	NA	NA	NA	NA	0.152869572	0.857408242	0.154667139	1.253188439	0.164447496	1.285516125	0.516773086	2.02E-08	1.19E-06
TCONS_00036786	WBGene00047593	21ur-176	NA	NA	NA	NA	0	0.006029091	0.487637405	0	0	0	1.403041141	6.59E-22	1.34E-19
TCONS_00027956	WBGene00047630	21ur-1741	NA	NA	NA	NA	0	0.825985459	0.728653594	0	0.556246697	0	0	0.000952431	0.031339626
TCONS_00038958	WBGene00047645	21ur-3986	NA	NA	NA	NA	0	0	0	0.63822568	0	0	0	9.75E-12	7.70E-10
TCONS_00036801	WBGene00047656	21ur-477	NA	NA	NA	NA	0	0	0	0.011047424	0.010206361	0.016238802	1.422903823	3.05E-10	2.09E-08
TCONS_00027959	WBGene00047672	21ur-820	NA	NA	NA	NA	0	0.825985459	0.728653594	0	0.556246697	0	0	0.000952431	0.031339626
TCONS_00048126	WBGene00047684	21ur-1565	NA	NA	NA	NA	0	0	0.487637405	0	0	0.633313279	1.403041141	2.07E-06	9.58E-05
TCONS_00039982	WBGene00047687	21ur-4368	NA	NA	NA	NA	0.104625351	0.091604788	0.169208587	0.13010711	0.870782177	0.060100565	0.068955388	0.001279963	0.041002632
TCONS_00038825	WBGene00047694	21ur-12	NA	NA	NA	NA	2.145466383	8.042104699	0.961754503	6.656691758	0.777072662	0.590815731	0.682476867	2.13E-07	1.17E-05
TCONS_00048592	WBGene00047703	21ur-4890	NA	NA	NA	NA	0	0	0.487637405	0.006382257	0	0	1.403041141	2.82E-21	4.67E-19
TCONS_00036806	WBGene00047748	21ur-4298	NA	NA	NA	NA	0	0	0.006881659	0.011047424	0	0.005412934	1.408887146	7.32E-11	5.40E-09
TCONS_00036411	WBGene00047798	21ur-366	NA	NA	NA	NA	0	0	0.494519064	0	0	0.633313279	1.410049479	1.86E-06	9.22E-05
TCONS_00038896	WBGene00047834	21ur-2878	NA	NA	NA	NA	0.007000282	7.45058396	0.011210055	0.685849554	0.017137904	0.005412934	0.035041693	1.51E-19	2.26E-17
TCONS_00036805	WBGene00047843	21ur-2912	NA	NA	NA	NA	0.007000282	0	0	0.01742968	0	0.005412934	1.408887146	2.50E-09	1.59E-07
TCONS_00036415	WBGene00047861	21ur-887	NA	NA	NA	NA	0	0	0.487637405	0	0	0	1.403041141	7.09E-24	1.59E-21
TCONS_00026824	WBGene00047873	21ur-2664	NA	NA	NA	NA	1.769724421	7.442155697	0	5.936323828	0	0	0	1.50E-44	2.44E-41
TCONS_00026838	WBGene00047957	21ur-4816	NA	NA	NA	NA	1.769724421	7.442155697	0.005605028	5.929941571	0	0	0	8.27E-43	8.73E-40
TCONS_00036822	WBGene00047968	21ur-4586	NA	NA	NA	NA	0.03254169	0.006029091	0.069315093	0.049340964	0.039378988	0.016238802	1.462629187	1.57E-08	9.30E-07
TCONS_00026943	WBGene00048033	21ur-2425	NA	NA	NA	NA	1.292574579	9.431225623	0.068816591	6.740759131	0.006931542	0	0	3.10E-37	1.56E-34
TCONS_00048244	WBGene00048065	21ur-576	NA	NA	NA	NA	0	0	0	0	0	0	1.403041141	1.86E-15	1.81E-13
TCONS_00027869	WBGene00048075	21ur-4552	NA	NA	NA	NA	0	0	0	0.011047424	0.776332753	0	0	3.01E-10	2.06E-08
TCONS_00039892	WBGene00048109	21ur-2480	NA	NA	NA	NA	0.959038594	0.83201455	0	0.72671873	0.783264295	1.406833835	0.507405744	1.58E-05	0.00068559
TCONS_00048310	WBGene00048117	21ur-636	NA	NA	NA	NA	0	0	0.494519064	0	0	0.633313279	1.403041141	1.95E-06	9.49E-05
TCONS_00038836	WBGene00048129	21ur-3719	NA	NA	NA	NA	1.604177379	7.425299173	0	5.918894148	0	0	0	1.60E-44	2.44E-41
TCONS_00036422	WBGene00048137	21ur-3782	NA	NA	NA	NA	0	0	0.487637405	0.005523712	0	0	1.403041141	2.80E-21	4.67E-19
TCONS_00027668	WBGene00048151	21ur-3568	NA	NA	NA	NA	0	0	0	0	0	0.858933255	0	9.33E-13	7.88E-11
TCONS_00048228	WBGene00048153	21ur-3355	NA	NA	NA	NA	0	0	0.487637405	0	0	0.633313279	1.403041141	2.07E-06	9.58E-05
TCONS_00026806	WBGene00048175	21ur-3367	NA	NA	NA	NA	1.781265266	7.442155697	0.012486687	5.946512707	0.010206361	0.022845981	0	9.96E-41	8.54E-38
TCONS_00048231	WBGene00048198	21ur-5350	NA	NA	NA	NA	0	0	0.487637405	0	0	0.633313279	1.403041141	2.07E-06	9.58E-05
TCONS_00039902	WBGene00048205	21ur-2322	NA	NA	NA	NA	0.959038594	0.840442812	0.006881659	0.721195018	0.776332753	1.406833835	0.507405744	2.04E-05	0.000878052
TCONS_00039681	WBGene00048236	21ur-4460	NA	NA	NA	NA	0	0.707974041	0.005605028	1.160516326	0.006931542	1.254868721	0.473526385	2.06E-16	2.35E-14
TCONS_00026875	WBGene00048253	21ur-4001	NA	NA	NA	NA	0	0	0	0.643749391	0	0	0	8.90E-12	7.27E-10
TCONS_00027946	WBGene00048299	21ur-2429	NA	NA	NA	NA	0	0.825985459	0.728653594	0	0.556246697	0	0	0.000952431	0.031339626
TCONS_00026778	WBGene00048377	21ur-2121	NA	NA	NA	NA	2.042543001	7.980770015	0.621781072	5.709655779	0.813176832	0.615491911	0.529455751	9.10E-09	5.45E-07
TCONS_00036773	WBGene00048402	21ur-207	NA	NA	NA	NA	0.005770422	0	0.487637405	0.006382257	0	0	1.403041141	2.82E-21	4.67E-19

TCONS_00048239	WBGene00048452	21ur-244	NA	NA	NA	NA	0	0	0	0	0	0	1.403041141	1.86E-15	1.81E-13
TCONS_00036459	WBGene00048486	21ur-2235	NA	NA	NA	NA	0.069245067	0.048232728	0.487637405	0.027618559	0.129407879	0.801114233	1.403041141	1.44E-05	0.000639133
TCONS_00039773	WBGene00048518	21ur-1221	NA	NA	NA	NA	0	0	0	0.006382257	0	0.858933255	0	4.67E-13	4.07E-11
TCONS_00027661	WBGene00048626	21ur-3140	NA	NA	NA	NA	0	0	0	0	0.005103181	0.858933255	0	1.62E-10	1.16E-08
TCONS_00039896	WBGene00048633	21ur-4200	NA	NA	NA	NA	0.973039157	0.83201455	0	0.72671873	0.776332753	1.406833835	0.507405744	1.49E-05	0.000650724
TCONS_00036812	WBGene00048767	21ur-1088	NA	NA	NA	NA	0	0	0.006881659	0.011047424	0.005103181	0.005412934	1.408887146	3.52E-11	2.68E-09
TCONS_00038828	WBGene00048786	21ur-62	NA	NA	NA	NA	2.145466383	8.042104699	0.961754503	6.663074015	0.784004205	0.590815731	0.682476867	2.22E-07	1.21E-05
TCONS_00026869	WBGene00048817	21ur-2580	NA	NA	NA	NA	0	0	0	0.63822568	0	0	0	9.75E-12	7.70E-10
TCONS_00038907	WBGene00048836	21ur-3831	NA	NA	NA	NA	0	7.442155697	0	0.643749391	0	0	0	9.32E-34	4.08E-31
TCONS_00048361	WBGene00048852	21ur-173	NA	NA	NA	NA	0.007000282	0	0.494519064	0	0	0.633313279	1.403041141	1.95E-06	9.49E-05
TCONS_00036365	WBGene00048859	21ur-3536	NA	NA	NA	NA	0	0	0.494519064	0	0	0.633313279	1.410049479	1.86E-06	9.22E-05
TCONS_00039778	WBGene00048874	21ur-1829	NA	NA	NA	NA	0.007000282	0	0	0	0	0.865540434	0.007008339	2.82E-11	2.19E-09
TCONS_00038918	WBGene00048897	21ur-270	NA	NA	NA	NA	0	7.442155697	0	0.63822568	0	0	0	1.02E-33	4.08E-31
TCONS_00027784	WBGene00048900	21ur-5295	NA	NA	NA	NA	0	0.83201455	0.013763318	0.721195018	0.769401211	1.406833835	0.507405744	2.62E-05	0.001116901
TCONS_00036338	WBGene00048909	21ur-2628	NA	NA	NA	NA	0	0	0	0	0	0	1.403041141	1.86E-15	1.81E-13
TCONS_00048367	WBGene00048922	21ur-730	NA	NA	NA	NA	0	0	0	0	0	0	1.403041141	1.86E-15	1.81E-13
TCONS_00038951	WBGene00048934	21ur-3507	NA	NA	NA	NA	0	0.038573717	0.022420111	0.63822568	0.013863085	0	0.007008339	2.23E-06	0.000103108
TCONS_00036797	WBGene00048941	21ur-2738	NA	NA	NA	NA	0	0	0.487637405	0.011047424	0.005103181	0.010825868	1.415895484	2.43E-18	3.37E-16
TCONS_00027548	WBGene00049016	21ur-1205	NA	NA	NA	NA	0.152869572	0.857408242	0.154667139	1.253188439	0.164447496	1.285516125	0.516773086	2.02E-08	1.19E-06
TCONS_00036368	WBGene00049148	21ur-4978	NA	NA	NA	NA	0	0	0.494519064	0.006382257	0	0.633313279	1.403041141	6.25E-06	0.000283195
TCONS_00038818	WBGene00049252	21ur-5230	NA	NA	NA	NA	2.505033703	8.59966309	1.3300327	7.242773515	1.06530453	0.734979422	1.202633947	2.46E-05	0.0010519
TCONS_00027928	WBGene00049265	21ur-3166	NA	NA	NA	NA	0	0.825985459	0.728653594	0	0.556246697	0	0.007008339	0.000583541	0.02037395
TCONS_00038817	WBGene00049272	21ur-3444	NA	NA	NA	NA	2.512033984	8.59966309	1.3300327	7.236391258	1.06530453	0.734979422	1.202633947	2.47E-05	0.001054236
TCONS_00026765	WBGene00049274	21ur-4280	NA	NA	NA	NA	0.409699978	0.307047366	0.005605028	1.883150363	0	0.019821537	0	2.52E-22	5.21E-20
TCONS_00036340	WBGene00049284	21ur-2399	NA	NA	NA	NA	0	0	0	0	0	0	1.403041141	1.86E-15	1.81E-13
TCONS_00048362	WBGene00049294	21ur-1471	NA	NA	NA	NA	0.007000282	0	0.494519064	0	0	0.633313279	1.403041141	1.95E-06	9.49E-05
TCONS_00048123	WBGene00049301	21ur-4871	NA	NA	NA	NA	0	0	0.487637405	0.022094847	0	0.633313279	1.403041141	8.70E-05	0.003512404
TCONS_00026825	WBGene00049302	21ur-4695	NA	NA	NA	NA	1.769724421	7.442155697	0	5.936323828	0	0	0	1.50E-44	2.44E-41
TCONS_00048363	WBGene00049313	21ur-2039	NA	NA	NA	NA	0.007000282	0	0.494519064	0	0	0.633313279	1.403041141	1.95E-06	9.49E-05
TCONS_00048642	WBGene00049339	21ur-1616	NA	NA	NA	NA	0	0	0	0.011047424	0	0.005412934	1.408887146	1.35E-10	9.77E-09
TCONS_00048607	WBGene00049385	21ur-18	NA	NA	NA	NA	0	0	0.487637405	0.006382257	0	0	1.403041141	2.82E-21	4.67E-19
TCONS_00039002	WBGene00049391	21ur-200	NA	NA	NA	NA	1.292574579	9.431225623	0.068816591	6.740759131	0	0	0	6.27E-38	3.27E-35
TCONS_00036794	WBGene00049400	21ur-3793	NA	NA	NA	NA	0	0	0	0.011047424	0.005103181	0.005412934	1.415895484	6.23E-11	4.65E-09
TCONS_00036816	WBGene00049432	21ur-2457	NA	NA	NA	NA	0	0	0	0.011047424	0	0.005412934	1.408887146	1.35E-10	9.77E-09
TCONS_00026791	WBGene00049456	21ur-950	NA	NA	NA	NA	2.145466383	8.042104699	0.967359531	6.656691758	0.784004205	0.585402797	0.682476867	2.44E-07	1.32E-05
TCONS_00048247	WBGene00049459	21ur-4290	NA	NA	NA	NA	0	0	0	0	0	0	1.403041141	1.86E-15	1.81E-13

TCONS_00039003	WBGene00049475	21ur-5352	NA	NA	NA	NA	1.292574579	9.431225623	0.068816591	6.740759131	0	0	0	6.27E-38	3.27E-35
TCONS_00027868	WBGene00049490	21ur-759	NA	NA	NA	NA	0	0	0	0.011047424	0.783264295	0.005412934	0	6.72E-09	4.12E-07
TCONS_00038920	WBGene00049539	21ur-1167	NA	NA	NA	NA	0.007000282	7.459012222	0.005605028	0.63822568	0.006931542	0.018627292	0.019862682	6.98E-25	1.73E-22
TCONS_00039897	WBGene00049551	21ur-4137	NA	NA	NA	NA	0.959038594	0.838043641	0	0.72671873	0.776332753	1.406833835	0.507405744	1.46E-05	0.000645429
TCONS_00036775	WBGene00049559	21ur-4476	NA	NA	NA	NA	0	0	0.487637405	0.006382257	0.006931542	0	1.403041141	1.36E-21	2.71E-19
TCONS_00026931	WBGene00049589	21ur-4486	NA	NA	NA	NA	0.357766178	9.567433219	0.194680462	6.706272212	0.136038678	0.088995434	0.058425712	4.95E-21	8.05E-19
TCONS_00026766	WBGene00049598	21ur-4762	NA	NA	NA	NA	0.409699978	0.307047366	0.005605028	1.883150363	0	0.019821537	0	2.52E-22	5.21E-20
TCONS_00039007	WBGene00049602	21ur-662	NA	NA	NA	NA	1.292574579	9.431225623	0.068816591	6.740759131	0	0	0	6.27E-38	3.27E-35
TCONS_00027781	WBGene00049633	21ur-2732	NA	NA	NA	NA	0.959038594	0.83201455	0	0.72671873	0.776332753	1.406833835	0.507405744	1.49E-05	0.000650724
TCONS_00048218	WBGene00049665	21ur-1415	NA	NA	NA	NA	0	0	0.487637405	0	0	0.633313279	1.403041141	2.07E-06	9.58E-05
TCONS_00038862	WBGene00049689	21ur-2269	NA	NA	NA	NA	0.292875206	8.133709486	0.598363958	1.156678247	0.564868178	0.50850514	0.533933409	0.00012196	0.004874146
TCONS_00039782	WBGene00049711	21ur-4293	NA	NA	NA	NA	0	0	0	0	0	0.858933255	0.014016677	2.61E-10	1.84E-08
TCONS_00038945	WBGene00049741	21ur-2673	NA	NA	NA	NA	0	0.020486444	0.011210055	0.663754707	0.025515904	0.005412934	0.01169201	8.73E-06	0.000392898
TCONS_00026794	WBGene00049742	21ur-2943	NA	NA	NA	NA	1.604177379	7.425299173	0	5.918894148	0	0	0	1.60E-44	2.44E-41
TCONS_00036791	WBGene00049762	21ur-1295	NA	NA	NA	NA	0	0.006029091	0	0.011047424	0.005103181	0.005412934	1.415895484	1.59E-09	1.03E-07
TCONS_00048608	WBGene00049773	21ur-691	NA	NA	NA	NA	0	0	0.487637405	0.006382257	0	0.005412934	1.403041141	2.68E-20	4.16E-18
TCONS_00026826	WBGene00049793	21ur-1052	NA	NA	NA	NA	1.769724421	7.442155697	0	5.936323828	0	0.013214358	0	4.24E-45	2.44E-41
TCONS_00048587	WBGene00049825	21ur-1827	NA	NA	NA	NA	0	0	0.487637405	0.006382257	0	0	1.403041141	2.82E-21	4.67E-19
TCONS_00026854	WBGene00049891	21ur-4194	NA	NA	NA	NA	0.007000282	7.442155697	0	0.650990193	0	0.012020113	0.012854343	1.11E-28	3.29E-26
TCONS_00038877	WBGene00049913	21ur-3864	NA	NA	NA	NA	0.044082534	7.448184788	0.005605028	0.656513905	0.013863085	0.010825868	0.012854343	2.98E-25	7.61E-23
TCONS_00027779	WBGene00049915	21ur-1804	NA	NA	NA	NA	0.959038594	0.83201455	0	0.72671873	0.776332753	1.406833835	0.507405744	1.49E-05	0.000650724
TCONS_00048641	WBGene00049921	21ur-706	NA	NA	NA	NA	0.007000282	0	0.006881659	0.011047424	0	0.005412934	1.408887146	7.32E-11	5.40E-09
TCONS_00048249	WBGene00049931	21ur-2723	NA	NA	NA	NA	0	0	0	0	0	0	1.403041141	1.86E-15	1.81E-13
TCONS_00026941	WBGene00049971	21ur-2585	NA	NA	NA	NA	1.292574579	9.431225623	0.068816591	6.740759131	0	0	0	6.27E-38	3.27E-35
TCONS_00026945	WBGene00049973	21ur-5123	NA	NA	NA	NA	1.292574579	9.431225623	0.068816591	6.740759131	0	0	0	6.27E-38	3.27E-35
TCONS_00036426	WBGene00049983	21ur-1759	NA	NA	NA	NA	0	0	0	0	0	0	1.403041141	1.86E-15	1.81E-13
TCONS_00048617	WBGene00050006	21ur-3099	NA	NA	NA	NA	0	0	0.487637405	0	0	0	1.403041141	7.09E-24	1.59E-21
TCONS_00036799	WBGene00050050	21ur-3277	NA	NA	NA	NA	0	0	0.011210055	0.011047424	0.012034723	0.005412934	1.415895484	9.30E-12	7.57E-10
TCONS_00038906	WBGene00050073	21ur-1305	NA	NA	NA	NA	0	7.442155697	0	0.644607936	0	0	0	9.21E-34	4.08E-31
TCONS_00026796	WBGene00050099	21ur-3428	NA	NA	NA	NA	1.604177379	7.425299173	0	5.918894148	0	0.005412934	0	8.42E-45	2.44E-41
TCONS_00048122	WBGene00050128	21ur-3486	NA	NA	NA	NA	0	0	0.487637405	0.022094847	0	0.633313279	1.403041141	8.70E-05	0.003512404
TCONS_00048241	WBGene00050129	21ur-3149	NA	NA	NA	NA	0	0	0	0	0.006931542	0	1.403041141	9.35E-16	1.04E-13
TCONS_00036269	WBGene00050195	21ur-633	NA	NA	NA	NA	0	0	0.487637405	0	0	0.633313279	1.403041141	2.07E-06	9.58E-05
TCONS_00038897	WBGene00050225	21ur-3717	NA	NA	NA	NA	0	7.45058396	0.005605028	0.680325842	0.017137904	0.005412934	0.028033354	4.44E-21	7.29E-19
TCONS_00026827	WBGene00050269	21ur-2318	NA	NA	NA	NA	0.315012677	7.831460463	0.022420111	6.279393724	0.630445719	0.005412934	0.018700348	1.47E-17	1.95E-15
TCONS_00036778	WBGene00050337	21ur-4675	NA	NA	NA	NA	0	0	0.494519064	0.006382257	0	0	1.403041141	2.49E-21	4.67E-19

TCONS_00026873	WBGene00050373	21ur-1826		NA	NA	NA	NA	0	0	0	0.63822568	0	0	0	9.75E-12	7.70E-10
TCONS_00048603	WBGene00050410	21ur-4262		NA	NA	NA	NA	0	0	0.487637405	0.006382257	0	0	1.403041141	2.82E-21	4.67E-19
TCONS_00038944	WBGene00050417	21ur-4263		NA	NA	NA	NA	0	0.020486444	0.018091714	0.669278418	0.032447446	0.012020113	0.01169201	2.92E-05	0.00123778
TCONS_00036821	WBGene00050434	21ur-1389		NA	NA	NA	NA	0	0	0	0.022953392	0	0.017433047	1.422903823	1.33E-07	7.37E-06
TCONS_00027663	WBGene00050451	21ur-4913		NA	NA	NA	NA	0	0	0	0	0.013863085	0.858933255	0.007008339	2.59E-08	1.50E-06
TCONS_00026807	WBGene00050493	21ur-3365		NA	NA	NA	NA	1.781265266	7.442155697	0.012486687	5.946512707	0.010206361	0.022845981	0	9.96E-41	8.54E-38
TCONS_00027552	WBGene00050503	21ur-1203		NA	NA	NA	NA	0	0.707974041	0	1.160516326	0	1.254868721	0.473526385	3.51E-17	4.24E-15
TCONS_00039786	WBGene00050516	21ur-141		NA	NA	NA	NA	0	0.006029091	0	0	0	0.858933255	0	4.43E-13	3.87E-11
TCONS_00048651	WBGene00050525	21ur-917		NA	NA	NA	NA	0	0	0	0.011047424	0.006931542	0.005412934	1.408887146	6.29E-11	4.68E-09
TCONS_00026938	WBGene00050526	21ur-4025		NA	NA	NA	NA	1.292574579	9.431225623	0.068816591	6.740759131	0	0	0	6.27E-38	3.27E-35
TCONS_00040052	WBGene00050588	21ur-2511		NA	NA	NA	NA	0	0.825985459	0.728653594	0	0.556246697	0	0	0.000952431	0.031339626
TCONS_00027580	WBGene00050609	21ur-3134		NA	NA	NA	NA	0	0.707974041	0	1.160516326	0	1.254868721	0.473526385	3.51E-17	4.24E-15
TCONS_00038871	WBGene00050631	21ur-3835		NA	NA	NA	NA	0.044082534	7.448184788	0.019368346	0.656513905	0	0.017433047	0.007008339	4.95E-29	1.50E-26
TCONS_00048644	WBGene00050713	21ur-4883		NA	NA	NA	NA	0	0	0	0.011047424	0	0.005412934	1.408887146	1.35E-10	9.77E-09
TCONS_00036430	WBGene00050719	21ur-3198		NA	NA	NA	NA	0	0	0	0	0	0	1.403041141	1.86E-15	1.81E-13
TCONS_00027660	WBGene00050729	21ur-2478		NA	NA	NA	NA	0	0	0.006881659	0.005523712	0.006931542	0.858933255	0	2.82E-09	1.79E-07
TCONS_00048284	WBGene00050753	21ur-652		NA	NA	NA	NA	0	0	0.487637405	0	0	0.633313279	1.403041141	2.07E-06	9.58E-05
TCONS_00038909	WBGene00050761	21ur-1700		NA	NA	NA	NA	0	7.442155697	0	0.63822568	0	0	0	1.02E-33	4.08E-31
TCONS_00048643	WBGene00050789	21ur-4526		NA	NA	NA	NA	0	0	0	0.011047424	0	0.005412934	1.41473315	1.30E-10	9.49E-09
TCONS_00027854	WBGene00050796	21ur-386		NA	NA	NA	NA	0.098854928	0.066257676	0.116210075	0.073152902	0.894851623	0.036060339	0.078288394	1.92E-05	0.000829151
TCONS_00048246	WBGene00050801	21ur-3051		NA	NA	NA	NA	0	0	0	0	0	0	1.403041141	1.86E-15	1.81E-13
TCONS_00038808	WBGene00050809	21ur-778		NA	NA	NA	NA	1.64494921	7.425299173	0	5.937182373	0.012034723	0.005412934	0.005846005	4.09E-39	3.12E-36
TCONS_00026870	WBGene00050810	21ur-3177		NA	NA	NA	NA	0	0	0	0.63822568	0	0	0.007008339	1.44E-09	9.42E-08
TCONS_00048125	WBGene00050818	21ur-1551		NA	NA	NA	NA	0	0	0.487637405	0.022094847	0	0.633313279	1.403041141	8.70E-05	0.003512404
TCONS_00048281	WBGene00050827	21ur-3483		NA	NA	NA	NA	0	0	0.487637405	0	0	0.633313279	1.403041141	2.07E-06	9.58E-05
TCONS_00027664	WBGene00050829	21ur-5405		NA	NA	NA	NA	0	0	0	0	0	0.865540434	0.007008339	2.82E-11	2.19E-09
TCONS_00039011	WBGene00050840	21ur-1597		NA	NA	NA	NA	1.292574579	9.431225623	0.068816591	6.740759131	0	0	0	6.27E-38	3.27E-35
TCONS_00021710	WBGene00050914	T12B5.15	III	49	302	954548	954295	8.848995042	6.221585579	6.977869945	7.200736265	3.873037308	5.82369568	0.697690214	0.001517794	0.047802964
TCONS_00054703	WBGene00077438	C18D4.10	V	200	766	17533078	17533644	5.64498844	0.185608747	8.812767921	4.411842289	10.34675886	0.212701633	6.167372296	1.06E-18	1.50E-16
TCONS_00060260	WBGene00077583	C01G10.18	V	1	201	1	201	0	1.349752732	0.005605028	0.038665983	0.035722265	0.037890538	0.007008339	1.33E-08	7.93E-07
TCONS_00060262	WBGene00077584	C01G10.19	V	198	432	15104958	15104724	0	1.33769455	0	0.012764514	0	0	0	1.61E-15	1.77E-13
TCONS_00007584	WBGene00077766	F17B5.9	I	508	1020	13183786	13183274	0.83889124	0.084344949	0.655192364	0.54879188	0.92986734	0.513995739	0.929818701	0.000352922	0.012902034
TCONS_00036802	WBGene00164993	21ur-13666		NA	NA	NA	NA	0	0	0	0.011047424	0	0.005412934	1.408887146	1.35E-10	9.77E-09
TCONS_00048591	WBGene00165005	21ur-12650		NA	NA	NA	NA	0	0	0.487637405	0.006382257	0	0	1.403041141	2.82E-21	4.67E-19
TCONS_00036353	WBGene00165012	21ur-13773		NA	NA	NA	NA	0	0	0.487637405	0	0	0.633313279	1.403041141	2.07E-06	9.58E-05
TCONS_00038955	WBGene00165017	21ur-5533		NA	NA	NA	NA	0	0	0	0.63822568	0	0	0	9.75E-12	7.70E-10

TCONS_00039009	WBGene00165047	21ur-12645	NA	NA	NA	NA	1.292574579	9.431225623	0.068816591	6.740759131	0	0	0	6.27E-38	3.27E-35
TCONS_00039706	WBGene00165048	21ur-7066	NA	NA	NA	NA	0.184181402	0.974235492	0.362612416	1.340336841	0.191791761	1.292123304	0.50624341	1.61E-06	8.07E-05
TCONS_00048287	WBGene00165088	21ur-7598	NA	NA	NA	NA	0	0	0.487637405	0	0	0.633313279	1.403041141	2.07E-06	9.58E-05
TCONS_00027669	WBGene00165093	21ur-13001	NA	NA	NA	NA	0	0	0	0	0	0.858933255	0	9.33E-13	7.88E-11
TCONS_00038902	WBGene00165094	21ur-7868	NA	NA	NA	NA	0	7.442155697	0	0.63822568	0	0	0	1.02E-33	4.08E-31
TCONS_00041484	WBGene00165136	21ur-8989	NA	NA	NA	NA	0.105004226	0.464847505	0.039235194	0.006382257	2.074230591	4.195023855	0.035041693	0.000235514	0.008910553
TCONS_00048131	WBGene00165167	21ur-11339	NA	NA	NA	NA	0	0	0.487637405	0	0	0.633313279	1.403041141	2.07E-06	9.58E-05
TCONS_00048288	WBGene00165239	21ur-6114	NA	NA	NA	NA	0	0	0.494519064	0	0	0.633313279	1.403041141	1.95E-06	9.49E-05
TCONS_00039997	WBGene00165240	21ur-10729	NA	NA	NA	NA	0	0	0	0.011047424	0.776332753	0	0	3.01E-10	2.06E-08
TCONS_00027550	WBGene00165279	21ur-15533	NA	NA	NA	NA	0	0.707974041	0	1.160516326	0	1.254868721	0.473526385	3.51E-17	4.24E-15
TCONS_00048234	WBGene00165281	21ur-14014	NA	NA	NA	NA	0	0	0	0	0	0.006607179	1.403041141	6.51E-14	5.95E-12
TCONS_00036428	WBGene00165290	21ur-6328	NA	NA	NA	NA	0	0	0	0	0	0	1.403041141	1.86E-15	1.81E-13
TCONS_00038820	WBGene00165345	21ur-10576	NA	NA	NA	NA	2.164007509	8.050532961	1.00354296	6.693268209	0.818661917	0.614855957	0.726885901	4.09E-07	2.16E-05
TCONS_00036782	WBGene00165349	21ur-6541	NA	NA	NA	NA	0	0	0.487637405	0.006382257	0	0	1.403041141	2.82E-21	4.67E-19
TCONS_00038804	WBGene00165379	21ur-6198	NA	NA	NA	NA	1.604177379	7.425299173	0	5.937182373	0.020794627	0.013214358	0	8.29E-41	7.58E-38
TCONS_00026790	WBGene00165410	21ur-6371	NA	NA	NA	NA	2.145466383	8.042104699	0.967359531	6.656691758	0.777072662	0.585402797	0.682476867	2.33E-07	1.26E-05
TCONS_00039008	WBGene00165419	21ur-13968	NA	NA	NA	NA	1.292574579	9.431225623	0.068816591	6.740759131	0	0	0	6.27E-38	3.27E-35
TCONS_00026880	WBGene00165426	21ur-8529	NA	NA	NA	NA	0	0	0	0.63822568	0	0	0	9.75E-12	7.70E-10
TCONS_00027564	WBGene00165432	21ur-14368	NA	NA	NA	NA	0	0.707974041	0	1.160516326	0	1.254868721	0.473526385	3.51E-17	4.24E-15
TCONS_00026928	WBGene00165461	21ur-13676	NA	NA	NA	NA	0.357766178	9.567433219	0.194680462	6.706272212	0.14297022	0.0835825	0.051417373	2.34E-21	4.60E-19
TCONS_00027556	WBGene00165470	21ur-8410	NA	NA	NA	NA	0	0.707974041	0.005605028	1.160516326	0.006931542	1.254868721	0.473526385	2.06E-16	2.35E-14
TCONS_00039903	WBGene00165478	21ur-14751	NA	NA	NA	NA	0.959038594	0.840442812	0.006881659	0.721195018	0.776332753	1.406833835	0.507405744	2.04E-05	0.000878052
TCONS_00039686	WBGene00165512	21ur-13930	NA	NA	NA	NA	0	0.707974041	0	1.166898583	0.013863085	1.254868721	0.473526385	1.77E-16	2.07E-14
TCONS_00039708	WBGene00165532	21ur-12754	NA	NA	NA	NA	0.184181402	0.974235492	0.362612416	1.340336841	0.191791761	1.292123304	0.50624341	1.61E-06	8.07E-05
TCONS_00040008	WBGene00165534	21ur-9814	NA	NA	NA	NA	0.042852675	0.057829414	0.044341719	0.04676533	0.894851623	0.057712075	0.065434051	0.001090916	0.03535943
TCONS_00026823	WBGene00165544	21ur-6716	NA	NA	NA	NA	1.776724703	7.448184788	0	5.955470598	0	0	0	1.43E-44	2.44E-41
TCONS_00040065	WBGene00165550	21ur-6243	NA	NA	NA	NA	0	0.825985459	0.728653594	0	0.556246697	0	0	0.000952431	0.031339626
TCONS_00048623	WBGene00165615	21ur-14142	NA	NA	NA	NA	0	0	0	0	0	0	1.403041141	1.86E-15	1.81E-13
TCONS_00048599	WBGene00165619	21ur-11569	NA	NA	NA	NA	0	0.008428262	0.487637405	0.006382257	0	0	1.403041141	7.84E-20	1.19E-17
TCONS_00027870	WBGene00165620	21ur-12244	NA	NA	NA	NA	0.007000282	0	0	0.011047424	0.776332753	0	0	3.01E-10	2.06E-08
TCONS_00038977	WBGene00165673	21ur-13900	NA	NA	NA	NA	7.240243258	9.899766676	3.979399027	8.17898435	4.521155694	6.385937383	0.446586693	5.18E-07	2.71E-05
TCONS_00026937	WBGene00165711	21ur-10009	NA	NA	NA	NA	1.292574579	9.431225623	0.068816591	6.740759131	0	0	0	6.27E-38	3.27E-35
TCONS_00038999	WBGene00165732	21ur-11759	NA	NA	NA	NA	1.292574579	9.431225623	0.068816591	6.740759131	0	0	0	6.27E-38	3.27E-35
TCONS_00038809	WBGene00165734	21ur-9336	NA	NA	NA	NA	1.604177379	7.465041313	0.013763318	5.938040918	0.006931542	0.010825868	0.007008339	1.78E-38	1.33E-35
TCONS_00048638	WBGene00165742	21ur-9764	NA	NA	NA	NA	0.007000282	0.014457353	0.006881659	0.016571135	0.010206361	0.005412934	1.429912161	5.33E-08	3.03E-06
TCONS_00039771	WBGene00165752	21ur-11026	NA	NA	NA	NA	0	0	0	0	0	0.864346189	0	8.71E-13	7.41E-11

TCONS_00048365	WBGene00165757	21ur-11592	NA	NA	NA	NA	0	0	0	0	0	0	1.403041141	1.86E-15	1.81E-13
TCONS_00026793	WBGene00165762	21ur-12642	NA	NA	NA	NA	1.604177379	7.425299173	0	5.925276404	0	0	0	1.58E-44	2.44E-41
TCONS_00036429	WBGene00165796	21ur-9301	NA	NA	NA	NA	0	0	0	0	0	0	1.403041141	1.86E-15	1.81E-13
TCONS_00048259	WBGene00165825	21ur-7013	NA	NA	NA	NA	0	0	0	0	0	0	1.403041141	1.86E-15	1.81E-13
TCONS_00036780	WBGene00165869	21ur-7974	NA	NA	NA	NA	0	0	0.487637405	0.006382257	0	0	1.403041141	2.82E-21	4.67E-19
TCONS_00038929	WBGene00165885	21ur-11795	NA	NA	NA	NA	0.450949857	7.836071832	0.426602109	1.013806624	0.453743917	0.569086331	0.725654896	0.000113743	0.004550165
TCONS_00039690	WBGene00165925	21ur-7880	NA	NA	NA	NA	0	0.707974041	0	1.160516326	0	1.254868721	0.473526385	3.51E-17	4.24E-15
TCONS_00036790	WBGene00165957	21ur-12152	NA	NA	NA	NA	0	0	0.487637405	0	0	0	1.403041141	7.09E-24	1.59E-21
TCONS_00036820	WBGene00165961	21ur-14182	NA	NA	NA	NA	0.005770422	0.008428262	0	0.011047424	0	0.005412934	1.408887146	3.44E-09	2.15E-07
TCONS_00027667	WBGene00165987	21ur-7003	NA	NA	NA	NA	0	0	0	0	0	0.858933255	0	9.33E-13	7.88E-11
TCONS_00027957	WBGene00166008	21ur-5469	NA	NA	NA	NA	0	0.825985459	0.728653594	0	0.556246697	0	0	0.000952431	0.031339626
TCONS_00038903	WBGene00166010	21ur-11707	NA	NA	NA	NA	0	7.442155697	0	0.63822568	0.006931542	0	0	3.50E-31	1.30E-28
TCONS_00027874	WBGene00166040	21ur-10778	NA	NA	NA	NA	0	0	0	0.011047424	0.776332753	0	0	3.01E-10	2.06E-08
TCONS_00027923	WBGene00166136	21ur-7448	NA	NA	NA	NA	0	0.825985459	0.728653594	0	0.556246697	0	0.007008339	0.000583541	0.02037395
TCONS_00026836	WBGene00166185	21ur-6527	NA	NA	NA	NA	0	7.442155697	0.005605028	0.644607936	0	0	0.007008339	1.33E-29	4.53E-27
TCONS_00036777	WBGene00166199	21ur-13249	NA	NA	NA	NA	0	0	0.494519064	0.006382257	0	0	1.403041141	2.49E-21	4.67E-19
TCONS_00036776	WBGene00166218	21ur-15470	NA	NA	NA	NA	0	0	0.487637405	0.006382257	0.006931542	0	1.403041141	1.36E-21	2.71E-19
TCONS_00026942	WBGene00166255	21ur-14617	NA	NA	NA	NA	1.292574579	9.431225623	0.068816591	6.740759131	0.006931542	0	0	3.10E-37	1.56E-34
TCONS_00040005	WBGene00166267	21ur-7970	NA	NA	NA	NA	0.058083098	0.030145455	0.065485198	0.063336465	0.886091719	0.066707744	0.056066709	0.000351378	0.012857016
TCONS_00026832	WBGene00166273	21ur-8507	NA	NA	NA	NA	0.044082534	7.448184788	0.005605028	0.656513905	0.005103181	0.017433047	0.012854343	6.27E-27	1.77E-24
TCONS_00026787	WBGene00166316	21ur-9085	NA	NA	NA	NA	2.157007227	8.050532961	1.00354296	6.693268209	0.811730375	0.614855957	0.726885901	3.91E-07	2.08E-05
TCONS_00039901	WBGene00166323	21ur-6917	NA	NA	NA	NA	0.959038594	0.83201455	0	0.72671873	0.776332753	1.406833835	0.507405744	1.49E-05	0.000650724
TCONS_00048237	WBGene00166334	21ur-14186	NA	NA	NA	NA	0	0	0	0	0	0.006607179	1.403041141	6.51E-14	5.95E-12
TCONS_00038849	WBGene00166350	21ur-6908	NA	NA	NA	NA	1.775494844	7.454213879	0	5.948229797	0.005103181	0.006607179	0	4.60E-43	4.98E-40
TCONS_00048253	WBGene00166443	21ur-8924	NA	NA	NA	NA	0	0	0	0	0	0	1.403041141	1.86E-15	1.81E-13
TCONS_00036370	WBGene00166455	21ur-6786	NA	NA	NA	NA	0	0	0.494519064	0	0	0.633313279	1.403041141	1.95E-06	9.49E-05
TCONS_00026837	WBGene00166490	21ur-13578	NA	NA	NA	NA	0	7.442155697	0.005605028	0.63822568	0	0	0.007008339	1.47E-29	4.54E-27
TCONS_00048596	WBGene00166565	21ur-7863	NA	NA	NA	NA	0	0.016856525	0.487637405	0.006382257	0	0	1.403041141	1.18E-18	1.67E-16
TCONS_00048219	WBGene00166606	21ur-14577	NA	NA	NA	NA	0	0	0.487637405	0	0	0.633313279	1.403041141	2.07E-06	9.58E-05
TCONS_00026819	WBGene00166608	21ur-8383	NA	NA	NA	NA	1.775494844	7.454213879	0	5.948229797	0.005103181	0.006607179	0	4.60E-43	4.98E-40
TCONS_00048250	WBGene00166676	21ur-10141	NA	NA	NA	NA	0	0	0	0	0	0	1.403041141	1.86E-15	1.81E-13
TCONS_00048132	WBGene00166703	21ur-11117	NA	NA	NA	NA	0	0	0.487637405	0	0	0.633313279	1.403041141	2.07E-06	9.58E-05
TCONS_00026858	WBGene00166830	21ur-11605	NA	NA	NA	NA	0.26440197	7.899868012	0.518835814	1.097520846	0.365080322	0.382735749	0.571540119	6.44E-05	0.002644051
TCONS_00027947	WBGene00166861	21ur-9170	NA	NA	NA	NA	0	0.825985459	0.728653594	0	0.556246697	0	0	0.000952431	0.031339626
TCONS_00038868	WBGene00166862	21ur-7559	NA	NA	NA	NA	0.044082534	7.456613051	0.005605028	0.656513905	0.005103181	0.042667518	0.021025016	3.84E-26	1.03E-23
TCONS_00038995	WBGene00166873	21ur-9262	NA	NA	NA	NA	0.3635366	9.545778351	0.186522171	6.707130757	0.172142847	0.078169566	0.035041693	7.54E-23	1.62E-20

TCONS_00048625	WBGene00166912	21ur-11540	NA	NA	NA	NA	0	0	0	0	0	0	1.403041141	1.86E-15	1.81E-13
TCONS_00048130	WBGene00166914	21ur-6572	NA	NA	NA	NA	0	0	0.487637405	0	0	0.633313279	1.403041141	2.07E-06	9.58E-05
TCONS_00038954	WBGene00166921	21ur-6508	NA	NA	NA	NA	0	0	0	0.63822568	0	0.005412934	0	5.09E-12	4.18E-10
TCONS_00048222	WBGene00166973	21ur-10982	NA	NA	NA	NA	0	0	0.487637405	0	0	0.633313279	1.403041141	2.07E-06	9.58E-05
TCONS_00048251	WBGene00166981	21ur-6655	NA	NA	NA	NA	0	0	0	0	0	0	1.403041141	1.86E-15	1.81E-13
TCONS_00026763	WBGene00166998	21ur-11788	NA	NA	NA	NA	0.409699978	0.307047366	0.005605028	1.883150363	0	0.019821537	0	2.52E-22	5.21E-20
TCONS_00038851	WBGene00167004	21ur-11381	NA	NA	NA	NA	1.769724421	7.442155697	0	5.936323828	0	0	0	1.50E-44	2.44E-41
TCONS_00036772	WBGene00167020	21ur-10655	NA	NA	NA	NA	0	0	0.487637405	0.006382257	0	0	1.403041141	2.82E-21	4.67E-19
TCONS_00048611	WBGene00167076	21ur-10620	NA	NA	NA	NA	0	0	0.487637405	0.006382257	0	0.005412934	1.403041141	2.68E-20	4.16E-18
TCONS_00027553	WBGene00167098	21ur-13890	NA	NA	NA	NA	0	0.707974041	0	1.160516326	0	1.254868721	0.473526385	3.51E-17	4.24E-15
TCONS_00039709	WBGene00167102	21ur-13457	NA	NA	NA	NA	0.184181402	0.974235492	0.362612416	1.340336841	0.191791761	1.292123304	0.50624341	1.61E-06	8.07E-05
TCONS_00026949	WBGene00167105	21ur-14515	NA	NA	NA	NA	1.516297953	9.789091862	0.082579909	7.153547748	0.331706746	0.397377344	0.63793049	1.86E-13	1.68E-11
TCONS_00027566	WBGene00167113	21ur-14847	NA	NA	NA	NA	0	0.707974041	0	1.160516326	0	1.254868721	0.473526385	3.51E-17	4.24E-15
TCONS_00039906	WBGene00167138	21ur-10204	NA	NA	NA	NA	0	0.83201455	0	0.721195018	0.776332753	1.412246769	0.507405744	1.52E-05	0.000663247
TCONS_00039013	WBGene00167143	21ur-6283	NA	NA	NA	NA	1.292574579	9.431225623	0.068816591	6.740759131	0.005103181	0	0.007008339	2.97E-35	1.34E-32
TCONS_00027880	WBGene00167146	21ur-9792	NA	NA	NA	NA	0	0	0	0.011047424	0.776332753	0.013214358	0	9.93E-08	5.55E-06
TCONS_00027823	WBGene00167178	21ur-10477	NA	NA	NA	NA	0.005770422	0.910392732	0.773493815	0.797782099	0	1.57813986	0.652359194	9.42E-06	0.000423292
TCONS_00048260	WBGene00167245	21ur-7441	NA	NA	NA	NA	0	0	0	0	0	0	1.403041141	1.86E-15	1.81E-13
TCONS_00036419	WBGene00167254	21ur-8818	NA	NA	NA	NA	0	0	0.487637405	0	0	0	1.403041141	7.09E-24	1.59E-21
TCONS_00038806	WBGene00167266	21ur-14120	NA	NA	NA	NA	1.616948083	7.433727435	0.011210055	5.937182373	0.006931542	0.012020113	0	4.56E-41	4.26E-38
TCONS_00039701	WBGene00167341	21ur-8828	NA	NA	NA	NA	0.076624224	0.83569105	0.119261839	1.2081402	0.086071424	1.293953503	0.499235072	2.23E-10	1.58E-08
TCONS_00048600	WBGene00167349	21ur-11983	NA	NA	NA	NA	0	0	0.487637405	0.006382257	0	0.005412934	1.403041141	2.68E-20	4.16E-18
TCONS_00038853	WBGene00167410	21ur-8151	NA	NA	NA	NA	1.769724421	7.442155697	0	5.936323828	0	0	0	1.50E-44	2.44E-41
TCONS_00038814	WBGene00167414	21ur-10249	NA	NA	NA	NA	2.334188347	8.339867002	1.234686477	6.192762882	0.915321607	0.899119977	0.908009042	1.12E-06	5.68E-05
TCONS_00036424	WBGene00167418	21ur-13549	NA	NA	NA	NA	0	0	0.507005751	0	0	0	1.403041141	4.97E-24	1.22E-21
TCONS_00048245	WBGene00167419	21ur-12005	NA	NA	NA	NA	0	0	0	0	0	0	1.403041141	1.86E-15	1.81E-13
TCONS_00036369	WBGene00167472	21ur-11211	NA	NA	NA	NA	0	0	0.494519064	0	0	0.633313279	1.403041141	1.95E-06	9.49E-05
TCONS_00038889	WBGene00167475	21ur-10768	NA	NA	NA	NA	0	7.442155697	0.005605028	0.643749391	0	0	0.007008339	1.34E-29	4.53E-27
TCONS_00038957	WBGene00167482	21ur-13589	NA	NA	NA	NA	0	0	0	0.63822568	0	0	0	9.75E-12	7.70E-10
TCONS_00038832	WBGene00167516	21ur-10277	NA	NA	NA	NA	1.604177379	7.425299173	0	5.925276404	0	0	0	1.58E-44	2.44E-41
TCONS_00048311	WBGene00167553	21ur-9801	NA	NA	NA	NA	0	0	0.494519064	0	0	0.633313279	1.403041141	1.95E-06	9.49E-05
TCONS_00039898	WBGene00167555	21ur-6463	NA	NA	NA	NA	0.959038594	0.83201455	0	0.733100986	0.776332753	1.406833835	0.507405744	1.41E-05	0.000627733
TCONS_00038891	WBGene00167562	21ur-15634	NA	NA	NA	NA	0.007000282	7.45058396	0.005605028	0.686708099	0.022241085	0	0.033879359	8.59E-19	1.24E-16
TCONS_00039900	WBGene00167574	21ur-5652	NA	NA	NA	NA	0.959038594	0.83201455	0	0.72671873	0.776332753	1.406833835	0.507405744	1.49E-05	0.000650724
TCONS_00048236	WBGene00167667	21ur-11558	NA	NA	NA	NA	0	0	0	0	0	0.006607179	1.403041141	6.51E-14	5.95E-12
TCONS_00016027	WBGene00167689	21ur-14772	NA	NA	NA	NA	0	2.514130923	0	0	0	0	0	3.85E-18	5.22E-16

TCONS_00048613	WBGene00167733	21ur-14616	NA	NA	NA	NA	0	0	0.487637405	0.006382257	0	0	1.403041141	2.82E-21	4.67E-19
TCONS_00048588	WBGene00167736	21ur-8356	NA	NA	NA	NA	0	0	0.487637405	0.006382257	0	0	1.403041141	2.82E-21	4.67E-19
TCONS_00038899	WBGene00167797	21ur-7754	NA	NA	NA	NA	0	7.442155697	0	0.63822568	0	0	0	1.02E-33	4.08E-31
TCONS_00038874	WBGene00167808	21ur-12793	NA	NA	NA	NA	0.044082534	7.448184788	0.005605028	0.656513905	0.006931542	0.010825868	0.012854343	1.55E-26	4.19E-24
TCONS_00039983	WBGene00167815	21ur-8729	NA	NA	NA	NA	0.104625351	0.091604788	0.169208587	0.13010711	0.870782177	0.060100565	0.068955388	0.001279963	0.041002632
TCONS_00027951	WBGene00167821	21ur-14735	NA	NA	NA	NA	0	0.825985459	0.728653594	0	0.556246697	0	0	0.000952431	0.031339626
TCONS_00027563	WBGene00167833	21ur-12541	NA	NA	NA	NA	0	0.707974041	0	1.160516326	0.006931542	1.254868721	0.473526385	8.24E-17	9.86E-15
TCONS_00036425	WBGene00167856	21ur-14602	NA	NA	NA	NA	0	0	0.005605028	0	0	0	1.403041141	1.00E-15	1.11E-13
TCONS_00039682	WBGene00167866	21ur-12094	NA	NA	NA	NA	0	0.707974041	0.005605028	1.160516326	0.006931542	1.254868721	0.473526385	2.06E-16	2.35E-14
TCONS_00026839	WBGene00167919	21ur-11328	NA	NA	NA	NA	0.021000845	7.456613051	0.012486687	0.680325842	0.017137904	0	0.033879359	3.75E-19	5.53E-17
TCONS_00026828	WBGene00167927	21ur-7462	NA	NA	NA	NA	0.292496331	7.834965734	0.340192306	1.051014297	0.366827523	0.317377579	0.334178591	3.03E-06	0.000139164
TCONS_00036781	WBGene00167945	21ur-8711	NA	NA	NA	NA	0	0	0.487637405	0.006382257	0	0	1.403041141	2.82E-21	4.67E-19
TCONS_00040066	WBGene00167952	21ur-5803	NA	NA	NA	NA	0	0.825985459	0.728653594	0	0.556246697	0	0	0.000952431	0.031339626
TCONS_00026926	WBGene00167988	21ur-14716	NA	NA	NA	NA	0.3635366	9.545778351	0.186522171	6.707130757	0.172142847	0.078169566	0.035041693	7.54E-23	1.62E-20
TCONS_00036354	WBGene00168061	21ur-5810	NA	NA	NA	NA	0	0	0.487637405	0	0	0.633313279	1.403041141	2.07E-06	9.58E-05
TCONS_00048593	WBGene00168086	21ur-15609	NA	NA	NA	NA	0	0	0.487637405	0.006382257	0	0	1.403041141	2.82E-21	4.67E-19
TCONS_00038926	WBGene00168088	21ur-8128	NA	NA	NA	NA	0.372716899	7.972217105	0.631215995	1.035415368	0.61125987	0.594320802	0.618170815	0.000449225	0.016093917
TCONS_00038959	WBGene00168101	21ur-9304	NA	NA	NA	NA	0	0	0	0.63822568	0	0	0	9.75E-12	7.70E-10
TCONS_00026935	WBGene00168105	21ur-12108	NA	NA	NA	NA	1.292574579	9.431225623	0.068816591	6.740759131	0	0	0	6.27E-38	3.27E-35
TCONS_00038875	WBGene00168146	21ur-15178	NA	NA	NA	NA	0.044082534	7.448184788	0.005605028	0.656513905	0.006931542	0.010825868	0.012854343	1.55E-26	4.19E-24
TCONS_00048257	WBGene00168149	21ur-9125	NA	NA	NA	NA	0.007000282	0	0	0	0	0	1.403041141	1.86E-15	1.81E-13
TCONS_00048229	WBGene00168172	21ur-7866	NA	NA	NA	NA	0	0	0.487637405	0	0	0.633313279	1.403041141	2.07E-06	9.58E-05
TCONS_00026813	WBGene00168177	21ur-10908	NA	NA	NA	NA	1.775494844	7.442155697	0.019368346	5.949088342	0.034275808	0	0	2.93E-36	1.36E-33
TCONS_00026857	WBGene00168213	21ur-8656	NA	NA	NA	NA	0.26440197	7.899868012	0.518835814	1.097520846	0.365080322	0.382735749	0.571540119	6.44E-05	0.002644051
TCONS_00048369	WBGene00168250	21ur-14974	NA	NA	NA	NA	0	0	0	0	0	0	1.403041141	1.86E-15	1.81E-13
TCONS_00011453	WBGene00168263	21ur-12159	NA	NA	NA	NA	2.573608314	0	1.472675038	1.046690114	3.776353718	0	0	4.46E-11	3.37E-09
TCONS_00026772	WBGene00168271	21ur-15226	NA	NA	NA	NA	1.604177379	7.425299173	0	5.925276404	0.006931542	0.012020113	0.007008339	2.45E-40	2.02E-37
TCONS_00048589	WBGene00168273	21ur-9944	NA	NA	NA	NA	0	0	0.487637405	0.006382257	0	0	1.403041141	2.82E-21	4.67E-19
TCONS_00027579	WBGene00168278	21ur-9090	NA	NA	NA	NA	0	0.707974041	0	1.160516326	0	1.254868721	0.473526385	3.51E-17	4.24E-15
TCONS_00026801	WBGene00168295	21ur-6891	NA	NA	NA	NA	1.611177661	7.425299173	0.005605028	5.918894148	0	0.005412934	0.005846005	3.21E-40	2.54E-37
TCONS_00048368	WBGene00168310	21ur-9009	NA	NA	NA	NA	0	0.008428262	0	0	0	0	1.403041141	6.10E-13	5.24E-11
TCONS_00027882	WBGene00168458	21ur-11979	NA	NA	NA	NA	0	0	0	0.011047424	0.776332753	0	0	3.01E-10	2.06E-08
TCONS_00036351	WBGene00168479	21ur-13776	NA	NA	NA	NA	0	0	0.487637405	0	0	0.633313279	1.403041141	2.07E-06	9.58E-05
TCONS_00027662	WBGene00168528	21ur-14516	NA	NA	NA	NA	0	0	0	0	0.013863085	0.858933255	0.007008339	2.59E-08	1.50E-06
TCONS_00048636	WBGene00168533	21ur-15636	NA	NA	NA	NA	0	0	0	0.016571135	0.012034723	0.005412934	1.408887146	5.46E-10	3.67E-08
TCONS_00036815	WBGene00168537	21ur-13278	NA	NA	NA	NA	0	0	0	0.011047424	0	0.005412934	1.41473315	1.30E-10	9.49E-09

TCONS_00026872	WBGene00168567	21ur-14805	NA	NA	NA	NA	0	0	0	0.63822568	0	0	0	9.75E-12	7.70E-10
TCONS_00036767	WBGene00168592	21ur-7951	NA	NA	NA	NA	0	0	0.487637405	0.006382257	0	0	1.403041141	2.82E-21	4.67E-19
TCONS_00041483	WBGene00168622	21ur-9485	NA	NA	NA	NA	0.105004226	0.464847505	0.039235194	0	2.074230591	4.195023855	0.035041693	0.000249947	0.009413383
TCONS_00026767	WBGene00168630	21ur-5783	NA	NA	NA	NA	0.409699978	0.307047366	0.005605028	1.883150363	0	0.019821537	0	2.52E-22	5.21E-20
TCONS_00026874	WBGene00168640	21ur-8920	NA	NA	NA	NA	0	0	0	0.63822568	0	0	0	9.75E-12	7.70E-10
TCONS_00048590	WBGene00168644	21ur-7873	NA	NA	NA	NA	0	0	0.487637405	0.006382257	0	0	1.403041141	2.82E-21	4.67E-19
TCONS_00036795	WBGene00168664	21ur-13569	NA	NA	NA	NA	0	0	0.487637405	0.011047424	0.005103181	0.005412934	1.415895484	5.50E-19	8.00E-17
TCONS_00038925	WBGene00168695	21ur-10369	NA	NA	NA	NA	0.372716899	7.972217105	0.631215995	1.035415368	0.61125987	0.594320802	0.618170815	0.000449225	0.016093917
TCONS_00048252	WBGene00168725	21ur-8607	NA	NA	NA	NA	0	0	0	0	0	0	1.403041141	1.86E-15	1.81E-13
TCONS_00039711	WBGene00168749	21ur-15369	NA	NA	NA	NA	0	0.707974041	0.006881659	1.160516326	0	1.254868721	0.473526385	1.01E-16	1.20E-14
TCONS_00026853	WBGene00168766	21ur-14622	NA	NA	NA	NA	0.007000282	7.442155697	0	0.650990193	0	0.012020113	0.012854343	1.11E-28	3.29E-26
TCONS_00011422	WBGene00168785	21ur-15355	NA	NA	NA	NA	2.759398451	2.640679508	1.600095168	1.258908017	4.062976807	0.208560608	5.188613458	3.51E-05	0.001472297
TCONS_00039012	WBGene00168790	21ur-10105	NA	NA	NA	NA	1.292574579	9.431225623	0.068816591	6.746282843	0	0	0	6.22E-38	3.27E-35
TCONS_00039893	WBGene00168797	21ur-12881	NA	NA	NA	NA	0.959038594	0.83201455	0	0.72671873	0.783264295	1.406833835	0.507405744	1.58E-05	0.00068559
TCONS_00048639	WBGene00168804	21ur-8132	NA	NA	NA	NA	0	0.006029091	0	0.011047424	0	0.005412934	1.408887146	3.40E-09	2.13E-07
TCONS_00026818	WBGene00168839	21ur-12498	NA	NA	NA	NA	1.775494844	7.454213879	0	5.948229797	0.005103181	0.006607179	0	4.60E-43	4.98E-40
TCONS_00027875	WBGene00168857	21ur-13537	NA	NA	NA	NA	0	0	0	0.011047424	0.776332753	0	0	3.01E-10	2.06E-08
TCONS_00048632	WBGene00168859	21ur-14350	NA	NA	NA	NA	0	0	0.487637405	0.011047424	0.005103181	0.010825868	1.415895484	2.43E-18	3.37E-16
TCONS_00026811	WBGene00168874	21ur-6061	NA	NA	NA	NA	1.775494844	7.442155697	0.019368346	5.949088342	0.034275808	0	0	2.93E-36	1.36E-33
TCONS_00048624	WBGene00168914	21ur-10781	NA	NA	NA	NA	0	0	0	0	0	0	1.403041141	1.86E-15	1.81E-13
TCONS_00039899	WBGene00168926	21ur-10884	NA	NA	NA	NA	0.959038594	0.83201455	0	0.733100986	0.776332753	1.406833835	0.507405744	1.41E-05	0.000627733
TCONS_00048621	WBGene00168928	21ur-15183	NA	NA	NA	NA	0	0	0	0	0	0	1.403041141	1.86E-15	1.81E-13
TCONS_00027572	WBGene00168942	21ur-9365	NA	NA	NA	NA	0.214170139	1.022343571	0.253563628	1.302529404	0.15134822	1.329377888	0.552977113	7.08E-08	3.98E-06
TCONS_00026768	WBGene00168970	21ur-13854	NA	NA	NA	NA	0.4154704	0.313076457	0.02930177	1.901438589	0.006931542	0.019821537	0.017538014	7.59E-14	6.93E-12
TCONS_00039001	WBGene00168978	21ur-10235	NA	NA	NA	NA	1.292574579	9.431225623	0.068816591	6.740759131	0	0	0	6.27E-38	3.27E-35
TCONS_00038803	WBGene00168985	21ur-9076	NA	NA	NA	NA	0.4154704	0.319105548	0.012486687	1.88953262	0	0.026428716	0.005846005	1.69E-19	2.53E-17
TCONS_00039689	WBGene00169095	21ur-8839	NA	NA	NA	NA	0	0.707974041	0	1.160516326	0	1.254868721	0.473526385	3.51E-17	4.24E-15
TCONS_00039703	WBGene00169147	21ur-8665	NA	NA	NA	NA	0.118247039	0.876663939	0.220930466	1.226800867	0.176482219	1.289734814	0.547131108	3.04E-07	1.63E-05
TCONS_00038802	WBGene00169153	21ur-9197	NA	NA	NA	NA	0.409699978	0.307047366	0.024973373	1.88953262	0.018966266	0.026428716	0.005846005	5.96E-16	6.69E-14
TCONS_00027551	WBGene00169172	21ur-8145	NA	NA	NA	NA	0	0.707974041	0	1.160516326	0	1.254868721	0.473526385	3.51E-17	4.24E-15
TCONS_00048598	WBGene00169211	21ur-5605	NA	NA	NA	NA	0	0.008428262	0.487637405	0.006382257	0	0	1.403041141	7.84E-20	1.19E-17
TCONS_00026912	WBGene00169316	21ur-6779	NA	NA	NA	NA	6.899629782	9.519138167	0	7.875209579	0	0.006607179	0	1.63E-47	6.70E-43
TCONS_00038824	WBGene00169383	21ur-9225	NA	NA	NA	NA	2.145466383	8.042104699	0.961754503	6.656691758	0.777072662	0.590815731	0.682476867	2.13E-07	1.17E-05
TCONS_00026822	WBGene00169384	21ur-7803	NA	NA	NA	NA	1.769724421	7.448184788	0	5.936323828	0	0	0	1.49E-44	2.44E-41
TCONS_00039996	WBGene00169402	21ur-10077	NA	NA	NA	NA	0	0	0	0.011047424	0.776332753	0	0	3.01E-10	2.06E-08
TCONS_00048629	WBGene00169425	21ur-6215	NA	NA	NA	NA	0	0.006029091	0	0.01742968	0.005103181	0.005412934	1.415895484	1.97E-08	1.16E-06

TCONS_00048360	WBGene00169430	21ur-11866	NA	NA	NA	NA	0	0	0.494519064	0	0	0.633313279	1.403041141	1.95E-06	9.49E-05
TCONS_00039904	WBGene00169476	21ur-7233	NA	NA	NA	NA	0	0.840442812	0	0.721195018	0.769401211	1.406833835	0.507405744	1.44E-05	0.000637516
TCONS_00038827	WBGene00169481	21ur-9442	NA	NA	NA	NA	2.145466383	8.042104699	0.961754503	6.656691758	0.777072662	0.590815731	0.688322871	2.26E-07	1.23E-05
TCONS_00038816	WBGene00169496	21ur-5726	NA	NA	NA	NA	2.334188347	7.863568817	0.47642735	6.82546485	0	0.502145595	0.438450356	1.62E-15	1.77E-13
TCONS_00038880	WBGene00169502	21ur-9353	NA	NA	NA	NA	0	7.442155697	0.005605028	0.63822568	0	0	0.007008339	1.47E-29	4.54E-27
TCONS_00026852	WBGene00169554	21ur-6661	NA	NA	NA	NA	0.014000563	7.456613051	0.018091714	0.644607936	0	0.006607179	0.007008339	1.73E-28	5.03E-26
TCONS_00036413	WBGene00169563	21ur-10474	NA	NA	NA	NA	0	0	0.494519064	0	0	0.633313279	1.403041141	1.95E-06	9.49E-05
TCONS_00038892	WBGene00169576	21ur-11239	NA	NA	NA	NA	0.007000282	7.45058396	0.031855032	0.680325842	0.017137904	0	0.033879359	1.98E-18	2.77E-16
TCONS_00039705	WBGene00169600	21ur-10911	NA	NA	NA	NA	0.184181402	0.974235492	0.362612416	1.340336841	0.191791761	1.292123304	0.50624341	1.61E-06	8.07E-05
TCONS_00027958	WBGene00169677	21ur-8677	NA	NA	NA	NA	0	0.825985459	0.728653594	0	0.556246697	0	0	0.000952431	0.031339626
TCONS_00036779	WBGene00169706	21ur-15120	NA	NA	NA	NA	0	0	0.487637405	0.006382257	0	0	1.403041141	2.82E-21	4.67E-19
TCONS_00036337	WBGene00169725	21ur-14965	NA	NA	NA	NA	0	0	0	0	0	0	1.403041141	1.86E-15	1.81E-13
TCONS_00026946	WBGene00169738	21ur-13631	NA	NA	NA	NA	1.292574579	9.431225623	0.068816591	6.740759131	0	0	0	6.27E-38	3.27E-35
TCONS_00027558	WBGene00169746	21ur-15389	NA	NA	NA	NA	0	0.707974041	0.005605028	1.160516326	0.006931542	1.254868721	0.473526385	2.06E-16	2.35E-14
TCONS_00038949	WBGene00169750	21ur-11097	NA	NA	NA	NA	0.007000282	0.006029091	0	0.63822568	0.025515904	0.012020113	0.026871021	1.84E-05	0.000798853
TCONS_00048283	WBGene00169755	21ur-6641	NA	NA	NA	NA	0	0	0.487637405	0	0	0.633313279	1.403041141	2.07E-06	9.58E-05
TCONS_00038829	WBGene00169768	21ur-6396	NA	NA	NA	NA	2.145466383	8.042104699	0.968636162	6.656691758	0.777072662	0.592009976	0.689485205	2.34E-07	1.27E-05
TCONS_00048289	WBGene00169790	21ur-15367	NA	NA	NA	NA	0	0	0.494519064	0	0	0.633313279	1.403041141	1.95E-06	9.49E-05
TCONS_00048233	WBGene00169792	21ur-10983	NA	NA	NA	NA	0	0	0	0	0	0	1.403041141	1.86E-15	1.81E-13
TCONS_00039995	WBGene00169838	21ur-11646	NA	NA	NA	NA	0	0	0	0.011047424	0.776332753	0	0	3.01E-10	2.06E-08
TCONS_00038928	WBGene00169845	21ur-9967	NA	NA	NA	NA	0.425029575	8.004761729	0.649307709	1.089907603	0.575155701	0.528249013	0.685998204	0.000783387	0.026761282
TCONS_00039999	WBGene00169857	21ur-7948	NA	NA	NA	NA	0	0	0.011210055	0.011047424	0.776332753	0	0	9.23E-11	6.77E-09
TCONS_00048364	WBGene00169895	21ur-11511	NA	NA	NA	NA	0	0	0.487637405	0	0	0	1.403041141	7.09E-24	1.59E-21
TCONS_00038826	WBGene00169910	21ur-13961	NA	NA	NA	NA	2.145466383	8.042104699	0.961754503	6.656691758	0.777072662	0.590815731	0.688322871	2.26E-07	1.23E-05
TCONS_00048312	WBGene00169918	21ur-15197	NA	NA	NA	NA	0	0	0.494519064	0	0	0.633313279	1.403041141	1.95E-06	9.49E-05
TCONS_00048609	WBGene00169935	21ur-11515	NA	NA	NA	NA	0	0	0.487637405	0.006382257	0	0.005412934	1.403041141	2.68E-20	4.16E-18
TCONS_00042089	WBGene00169991	21ur-12986	NA	NA	NA	NA	0	0.068656847	0	0.031052739	0	3.964307333	0	5.30E-24	1.29E-21
TCONS_00036792	WBGene00169994	21ur-14286	NA	NA	NA	NA	0	0.006029091	0	0.011047424	0.005103181	0.005412934	1.415895484	1.59E-09	1.03E-07
TCONS_00026810	WBGene00170013	21ur-11818	NA	NA	NA	NA	1.775494844	7.442155697	0.019368346	5.949088342	0.034275808	0.010825868	0	8.53E-37	4.13E-34
TCONS_00027886	WBGene00170017	21ur-14534	NA	NA	NA	NA	0.024311548	0.008428262	0.058105037	0.076100978	0.888301985	0.070926432	0.126150095	3.04E-06	0.000139164
TCONS_00026844	WBGene00170022	21ur-9926	NA	NA	NA	NA	0.005770422	7.442155697	0.013763318	0.650990193	0.010206361	0.010825868	0	1.01E-28	3.04E-26
TCONS_00038890	WBGene00170028	21ur-7064	NA	NA	NA	NA	0	7.442155697	0.005605028	0.63822568	0	0	0.007008339	1.47E-29	4.54E-27
TCONS_00038852	WBGene00170034	21ur-6981	NA	NA	NA	NA	1.769724421	7.442155697	0	5.936323828	0	0	0	1.50E-44	2.44E-41
TCONS_00048614	WBGene00170046	21ur-9499	NA	NA	NA	NA	0	0	0.487637405	0.006382257	0	0	1.403041141	2.82E-21	4.67E-19
TCONS_00027549	WBGene00170072	21ur-6340	NA	NA	NA	NA	0	0.707974041	0	1.160516326	0	1.254868721	0.473526385	3.51E-17	4.24E-15
TCONS_00039700	WBGene00170074	21ur-9034	NA	NA	NA	NA	0	0.707974041	0	1.160516326	0.006931542	1.254868721	0.473526385	8.24E-17	9.86E-15

TCONS_00026927	WBGene00170114	21ur-8549	NA	NA	NA	NA	0.36476646	9.567433219	0.194680462	6.712654469	0.136038678	0.0835825	0.051417373	1.70E-21	3.35E-19
TCONS_00038905	WBGene00170120	21ur-9241	NA	NA	NA	NA	0	7.442155697	0	0.63822568	0	0	0	1.02E-33	4.08E-31
TCONS_00027871	WBGene00170123	21ur-14521	NA	NA	NA	NA	0	0	0.011210055	0.011047424	0.776332753	0	0	9.23E-11	6.77E-09
TCONS_00039905	WBGene00170193	21ur-7138	NA	NA	NA	NA	0	0.83201455	0	0.721195018	0.776332753	1.412246769	0.507405744	1.52E-05	0.000663247
TCONS_00026933	WBGene00170215	21ur-10656	NA	NA	NA	NA	1.305345283	9.448082148	0.082579909	6.759047357	0.031000989	0.017433047	0.025708687	1.31E-28	3.85E-26
TCONS_00038833	WBGene00170249	21ur-12352	NA	NA	NA	NA	1.604177379	7.425299173	0	5.925276404	0	0	0	1.58E-44	2.44E-41
TCONS_00002573	WBGene00170280	21ur-14451	NA	NA	NA	NA	0	0.008428263	0.597962825	0.022094847	0	0.018627292	0.014016677	0.000539764	0.019039285
TCONS_00026797	WBGene00170294	21ur-8385	NA	NA	NA	NA	1.604177379	7.425299173	0	5.918894148	0	0	0	1.60E-44	2.44E-41
TCONS_00026795	WBGene00170303	21ur-11778	NA	NA	NA	NA	1.604177379	7.425299173	0	5.925276404	0	0	0	1.58E-44	2.44E-41
TCONS_00038843	WBGene00170308	21ur-10286	NA	NA	NA	NA	1.775494844	7.442155697	0.019368346	5.949088342	0.034275808	0.010825868	0	8.53E-37	4.13E-34
TCONS_00036423	WBGene00170314	21ur-13635	NA	NA	NA	NA	0	0	0.493242433	0	0	0	1.403041141	6.37E-24	1.54E-21
TCONS_00026936	WBGene00170383	21ur-8488	NA	NA	NA	NA	1.292574579	9.431225623	0.068816591	6.740759131	0	0	0	6.27E-38	3.27E-35
TCONS_00026855	WBGene00170397	21ur-15259	NA	NA	NA	NA	0.007000282	7.454213879	0	0.63822568	0	0.019821537	0.007008339	2.36E-31	9.24E-29
TCONS_00027571	WBGene00170427	21ur-13737	NA	NA	NA	NA	0.214170139	1.022343571	0.253563628	1.302529404	0.15134822	1.329377888	0.552977113	7.08E-08	3.98E-06
TCONS_00036764	WBGene00170438	21ur-7356	NA	NA	NA	NA	0	0	0.494519064	0.006382257	0	0	1.403041141	2.49E-21	4.67E-19
TCONS_00027574	WBGene00170474	21ur-9873	NA	NA	NA	NA	0	0.707974041	0	1.160516326	0	1.254868721	0.473526385	3.51E-17	4.24E-15
TCONS_00039684	WBGene00170564	21ur-9588	NA	NA	NA	NA	0	0.707974041	0	1.160516326	0.013863085	1.254868721	0.473526385	1.83E-16	2.12E-14
TCONS_00027867	WBGene00170579	21ur-11984	NA	NA	NA	NA	0	0	0	0.011047424	0.776332753	0.005412934	0	7.31E-09	4.44E-07
TCONS_00048227	WBGene00170581	21ur-15697	NA	NA	NA	NA	0	0	0.487637405	0	0	0.633313279	1.403041141	2.07E-06	9.58E-05
TCONS_00030381	WBGene00170609	21ur-8543	NA	NA	NA	NA	1.710230936	0.860306011	0.840158276	1.317755891	2.598354484	0.343806294	2.49196731	0.001173065	0.037754543
TCONS_00026923	WBGene00170617	21ur-14345	NA	NA	NA	NA	0.3635366	9.545778351	0.186522171	6.707130757	0.172142847	0.078169566	0.035041693	7.54E-23	1.62E-20
TCONS_00036334	WBGene00170624	21ur-8149	NA	NA	NA	NA	0	0	0.487637405	0	0	0.633313279	1.403041141	2.07E-06	9.58E-05
TCONS_00038846	WBGene00170720	21ur-15554	NA	NA	NA	NA	1.775494844	7.483128586	0.027526636	5.942706085	0.039378988	0	0.056066709	5.20E-23	1.14E-20
TCONS_00036341	WBGene00170735	21ur-10794	NA	NA	NA	NA	0	0	0	0	0	0	1.403041141	1.86E-15	1.81E-13
TCONS_00038838	WBGene00170802	21ur-7067	NA	NA	NA	NA	1.604177379	7.425299173	0	5.918894148	0	0	0	1.60E-44	2.44E-41
TCONS_00038916	WBGene00170806	21ur-13327	NA	NA	NA	NA	1.077751802	8.596344792	1.117698274	1.482690904	0.866500066	0.575771174	0.467302689	0.000443125	0.015916942
TCONS_00038819	WBGene00170812	21ur-13307	NA	NA	NA	NA	2.157007227	8.050532961	1.014753015	6.699650466	0.816833555	0.614855957	0.726885901	4.27E-07	2.26E-05
TCONS_00038886	WBGene00170813	21ur-6233	NA	NA	NA	NA	0	7.442155697	0.005605028	0.63822568	0	0	0.012854343	2.83E-27	8.04E-25
TCONS_00038821	WBGene00170837	21ur-6379	NA	NA	NA	NA	2.157007227	8.050532961	1.00354296	6.693268209	0.811730375	0.614855957	0.726885901	3.91E-07	2.08E-05
TCONS_00038873	WBGene00170852	21ur-7327	NA	NA	NA	NA	0.044082534	7.448184788	0.005605028	0.656513905	0.006931542	0.010825868	0.012854343	1.55E-26	4.19E-24
TCONS_00026877	WBGene00170853	21ur-5495	NA	NA	NA	NA	0	0	0	0.63822568	0	0	0	9.75E-12	7.70E-10
TCONS_00026786	WBGene00170872	21ur-10399	NA	NA	NA	NA	2.157007227	8.050532961	1.00354296	6.693268209	0.818661917	0.620268891	0.726885901	3.86E-07	2.06E-05
TCONS_00026764	WBGene00170876	21ur-13153	NA	NA	NA	NA	0.409699978	0.307047366	0.005605028	1.88953262	0	0.019821537	0	2.43E-22	5.16E-20
TCONS_00038901	WBGene00170888	21ur-14373	NA	NA	NA	NA	0	7.442155697	0	0.63822568	0	0	0	1.02E-33	4.08E-31
TCONS_00026898	WBGene00170906	21ur-13395	NA	NA	NA	NA	7.272784948	9.903396595	4.000044004	8.191748863	4.526258874	6.407589119	0.47229538	6.37E-07	3.29E-05
TCONS_00038904	WBGene00170936	21ur-15216	NA	NA	NA	NA	0	7.442155697	0	0.63822568	0.006931542	0	0	3.50E-31	1.30E-28

TCONS_00039698	WBGene00170956	21ur-10904	NA	NA	NA	NA	0.024311548	0.765803455	1.181590597	1.214522457	0.056898797	1.285516125	0.487543062	0.00040432	0.014586715
TCONS_00038952	WBGene00170959	21ur-9901	NA	NA	NA	NA	0	0	0	0.63822568	0	0	0	9.75E-12	7.70E-10
TCONS_00038887	WBGene00170969	21ur-9366	NA	NA	NA	NA	0.012770704	7.442155697	0.005605028	0.63822568	0	0	0.007008339	1.47E-29	4.54E-27
TCONS_00039779	WBGene00170975	21ur-14741	NA	NA	NA	NA	0	0	0.005605028	0	0	0.858933255	0.007008339	1.22E-09	8.05E-08
TCONS_00038923	WBGene00170980	21ur-7956	NA	NA	NA	NA	0.007000282	7.45058396	0	0.63822568	0.006931542	0.013214358	0.007008339	3.37E-29	1.03E-26
TCONS_00038807	WBGene00171029	21ur-15620	NA	NA	NA	NA	1.64494921	7.425299173	0	5.937182373	0.012034723	0.005412934	0.005846005	4.09E-39	3.12E-36
TCONS_00039676	WBGene00171042	21ur-9065	NA	NA	NA	NA	0	0.707974041	0	1.160516326	0	1.254868721	0.473526385	3.51E-17	4.24E-15
TCONS_00036774	WBGene00171062	21ur-14433	NA	NA	NA	NA	0	0	0.487637405	0.006382257	0.006931542	0	1.403041141	1.36E-21	2.71E-19
TCONS_00027561	WBGene00171106	21ur-12712	NA	NA	NA	NA	0	0.707974041	0	1.160516326	0	1.254868721	0.473526385	3.51E-17	4.24E-15
TCONS_00026816	WBGene00171119	21ur-7187	NA	NA	NA	NA	1.775494844	7.454213879	0	5.948229797	0.005103181	0.006607179	0	4.60E-43	4.98E-40
TCONS_00027852	WBGene00171185	21ur-11548	NA	NA	NA	NA	0.098854928	0.066257676	0.110605047	0.073152902	0.894851623	0.036060339	0.078288394	2.41E-05	0.001033078
TCONS_00039004	WBGene00171193	21ur-9938	NA	NA	NA	NA	1.292574579	9.431225623	0.068816591	6.740759131	0	0	0	6.27E-38	3.27E-35
TCONS_00038895	WBGene00171205	21ur-9051	NA	NA	NA	NA	0.007000282	7.45058396	0.012486687	0.680325842	0.017137904	0	0.033879359	3.77E-19	5.54E-17
TCONS_00036336	WBGene00171207	21ur-6088	NA	NA	NA	NA	0	0	0	0	0	0	1.403041141	1.86E-15	1.81E-13
TCONS_00048634	WBGene00171208	21ur-13135	NA	NA	NA	NA	0	0	0	0.011047424	0.012034723	0.005412934	1.422903823	2.88E-11	2.23E-09
TCONS_00038921	WBGene00171221	21ur-8114	NA	NA	NA	NA	0.012770704	7.442155697	0.016815083	0.65737245	0.006931542	0.013214358	0.012854343	1.30E-25	3.40E-23
TCONS_00027872	WBGene00171243	21ur-8913	NA	NA	NA	NA	0	0	0.011210055	0.011047424	0.776332753	0	0	9.23E-11	6.77E-09
TCONS_00038854	WBGene00171248	21ur-12865	NA	NA	NA	NA	1.769724421	7.442155697	0	5.936323828	0	0	0	1.50E-44	2.44E-41
TCONS_00039015	WBGene00171289	21ur-15218	NA	NA	NA	NA	1.292574579	9.431225623	0.07569825	0.095733852	0	0	0	5.86E-21	9.45E-19
TCONS_00039010	WBGene00171329	21ur-10189	NA	NA	NA	NA	1.292574579	9.431225623	0.068816591	6.740759131	0	0	0.005846005	8.44E-36	3.86E-33
TCONS_00039774	WBGene00171364	21ur-6358	NA	NA	NA	NA	0	0	0.005605028	0	0.006931542	0.858933255	0	5.64E-09	3.48E-07
TCONS_00026781	WBGene00171382	21ur-14319	NA	NA	NA	NA	2.334188347	8.33746783	1.247173164	5.695805397	0.922253149	0.899119977	0.915017381	2.06E-06	9.58E-05
TCONS_00026804	WBGene00171384	21ur-10378	NA	NA	NA	NA	1.775494844	7.448184788	0	5.929941571	0	0	0	1.51E-44	2.44E-41
TCONS_00038870	WBGene00171386	21ur-15344	NA	NA	NA	NA	0.044082534	7.448184788	0.005605028	0.656513905	0	0.010825868	0.007008339	2.95E-30	1.06E-27
TCONS_00039777	WBGene00171403	21ur-10906	NA	NA	NA	NA	0	0	0	0	0	0.865540434	0.007008339	2.82E-11	2.19E-09
TCONS_00027577	WBGene00171480	21ur-15019	NA	NA	NA	NA	0	0.707974041	0	1.160516326	0	1.254868721	0.473526385	3.51E-17	4.24E-15
TCONS_00038872	WBGene00171487	21ur-9624	NA	NA	NA	NA	0.044082534	7.448184788	0.026250005	0.656513905	0	0.017433047	0.007008339	1.97E-28	5.67E-26
TCONS_00027955	WBGene00171494	21ur-6155	NA	NA	NA	NA	0	0.825985459	0.728653594	0	0.556246697	0	0	0.000952431	0.031339626
TCONS_00048153	WBGene00171501	21ur-7433	NA	NA	NA	NA	0	0	0	0	0	0	1.408887146	1.77E-15	1.81E-13
TCONS_00048220	WBGene00171527	21ur-9687	NA	NA	NA	NA	0	0	0.487637405	0	0	0.633313279	1.403041141	2.07E-06	9.58E-05
TCONS_00038876	WBGene00171549	21ur-10611	NA	NA	NA	NA	0.044082534	7.448184788	0.005605028	0.656513905	0.006931542	0.010825868	0.012854343	1.55E-26	4.19E-24
TCONS_00038842	WBGene00171555	21ur-7123	NA	NA	NA	NA	1.775494844	7.442155697	0.019368346	5.949088342	0.034275808	0.010825868	0	8.53E-37	4.13E-34
TCONS_00038879	WBGene00171565	21ur-14043	NA	NA	NA	NA	0	7.442155697	0.005605028	0.63822568	0	0	0.007008339	1.47E-29	4.54E-27
TCONS_00027570	WBGene00171603	21ur-10386	NA	NA	NA	NA	0.214170139	1.022343571	0.253563628	1.302529404	0.15134822	1.329377888	0.552977113	7.08E-08	3.98E-06
TCONS_00036787	WBGene00171618	21ur-11770	NA	NA	NA	NA	0	0	0.487637405	0	0	0	1.403041141	7.09E-24	1.59E-21
TCONS_00039699	WBGene00171637	21ur-11318	NA	NA	NA	NA	0.024311548	0.765803455	1.181590597	1.214522457	0.056898797	1.285516125	0.487543062	0.00040432	0.014586715

TCONS_00006675	WBGene00171663	21ur-15317	NA	NA	NA	NA	0	0	0.57171282	0	0	0	0.01169201	3.28E-11	2.50E-09
TCONS_00038884	WBGene00171680	21ur-6916	NA	NA	NA	NA	0.005770422	7.442155697	0.005605028	0.63822568	0	0	0.007008339	1.47E-29	4.54E-27
TCONS_00048256	WBGene00171736	21ur-7350	NA	NA	NA	NA	0	0	0	0	0	0	1.403041141	1.86E-15	1.81E-13
TCONS_00027887	WBGene00171744	21ur-10452	NA	NA	NA	NA	0.024311548	0.008428262	0.058105037	0.076100978	0.888301985	0.070926432	0.126150095	3.04E-06	0.000139164
TCONS_00038993	WBGene00171753	21ur-15272	NA	NA	NA	NA	0.36476646	9.560235705	0.180917143	6.707130757	0.165211305	0.0835825	0.04905837	1.08E-21	2.20E-19
TCONS_00026814	WBGene00171760	21ur-7471	NA	NA	NA	NA	1.775494844	7.454213879	0	5.948229797	0.005103181	0.006607179	0	4.60E-43	4.98E-40
TCONS_00036788	WBGene00171787	21ur-8876	NA	NA	NA	NA	0	0	0.487637405	0	0	0	1.403041141	7.09E-24	1.59E-21
TCONS_00026773	WBGene00171799	21ur-6616	NA	NA	NA	NA	1.604177379	7.425299173	0	5.925276404	0.006931542	0.012020113	0.007008339	2.45E-40	2.02E-37
TCONS_00039894	WBGene00171806	21ur-9606	NA	NA	NA	NA	0.959038594	0.83201455	0	0.72671873	0.783264295	1.406833835	0.507405744	1.58E-05	0.00068559
TCONS_00036421	WBGene00171827	21ur-13518	NA	NA	NA	NA	0	0	0.487637405	0	0	0	1.403041141	7.09E-24	1.59E-21
TCONS_00026859	WBGene00171834	21ur-11876	NA	NA	NA	NA	0.425029575	8.004761729	0.649307709	1.089907603	0.575155701	0.522836079	0.685998204	0.000821845	0.028005321
TCONS_00048285	WBGene00171891	21ur-6108	NA	NA	NA	NA	0	0	0.487637405	0	0	0.633313279	1.403041141	2.07E-06	9.58E-05
TCONS_00048616	WBGene00171901	21ur-5740	NA	NA	NA	NA	0	0	0.487637405	0.006382257	0	0	1.403041141	2.82E-21	4.67E-19
TCONS_00026876	WBGene00171912	21ur-11856	NA	NA	NA	NA	0	0	0	0.63822568	0	0	0	9.75E-12	7.70E-10
TCONS_00026803	WBGene00171933	21ur-6591	NA	NA	NA	NA	2.109707224	7.778429393	0.238742553	6.124616132	0.170997134	0.215245451	0.014016677	3.08E-26	8.29E-24
TCONS_00036793	WBGene00171973	21ur-10788	NA	NA	NA	NA	0	0	0	0.011047424	0.005103181	0.005412934	1.415895484	6.23E-11	4.65E-09
TCONS_00043265	WBGene00172007	21ur-10500	NA	NA	NA	NA	0.729346452	0.498934502	0.972903806	0.31567866	1.057064953	0.08891777	0.2057725	0.000595431	0.020771476
TCONS_00027567	WBGene00172049	21ur-12897	NA	NA	NA	NA	0	0.707974041	0	1.160516326	0	1.254868721	0.473526385	3.51E-17	4.24E-15
TCONS_00048221	WBGene00172055	21ur-5995	NA	NA	NA	NA	0	0	0.487637405	0	0	0.633313279	1.403041141	2.07E-06	9.58E-05
TCONS_00039908	WBGene00172092	21ur-12794	NA	NA	NA	NA	0	0.83201455	0	0.721195018	0.769401211	1.406833835	0.507405744	1.47E-05	0.000649439
TCONS_00005530	WBGene00172093	21ur-15496	NA	NA	NA	NA	3.585437631	2.013716375	1.658479832	1.040307858	0	2.237323564	0	1.27E-15	1.40E-13
TCONS_00040012	WBGene00172103	21ur-7664	NA	NA	NA	NA	0.017311267	0.006029091	0.033131664	0.033142271	0.843437911	0.036060339	0.103962745	4.52E-08	2.57E-06
TCONS_00036355	WBGene00172155	21ur-11861	NA	NA	NA	NA	0	0	0.487637405	0	0	0.633313279	1.403041141	2.07E-06	9.58E-05
TCONS_00026835	WBGene00172176	21ur-7010	NA	NA	NA	NA	0	7.442155697	0.005605028	0.63822568	0	0	0.007008339	1.47E-29	4.54E-27
TCONS_00026809	WBGene00172181	21ur-11568	NA	NA	NA	NA	1.775494844	7.442155697	0.019368346	5.949088342	0.034275808	0	0	2.93E-36	1.36E-33
TCONS_00026817	WBGene00172200	21ur-8774	NA	NA	NA	NA	1.775494844	7.454213879	0	5.948229797	0.005103181	0.006607179	0	4.60E-43	4.98E-40
TCONS_00027873	WBGene00172206	21ur-6053	NA	NA	NA	NA	0	0	0	0.011047424	0.776332753	0	0	3.01E-10	2.06E-08
TCONS_00027782	WBGene00172223	21ur-11665	NA	NA	NA	NA	0.959038594	0.83201455	0	0.72671873	0.776332753	1.406833835	0.507405744	1.49E-05	0.000650724
TCONS_00048602	WBGene00172251	21ur-12049	NA	NA	NA	NA	0	0	0.487637405	0.006382257	0	0	1.403041141	2.82E-21	4.67E-19
TCONS_00040051	WBGene00172263	21ur-10883	NA	NA	NA	NA	0	0.825985459	0.728653594	0	0.556246697	0	0.014016677	0.000366871	0.013340885
TCONS_00042088	WBGene00172298	21ur-12489	NA	NA	NA	NA	0.056002254	0.075854362	0.016815083	0.054006131	0.051795616	4.001561916	0	2.61E-10	1.84E-08
TCONS_00027568	WBGene00172363	21ur-11186	NA	NA	NA	NA	0	0.707974041	0	1.160516326	0	1.254868721	0.473526385	3.51E-17	4.24E-15
TCONS_00026944	WBGene00172376	21ur-7558	NA	NA	NA	NA	1.292574579	9.431225623	0.068816591	6.740759131	0	0	0	6.27E-38	3.27E-35
TCONS_00026843	WBGene00172394	21ur-6975	NA	NA	NA	NA	0.005770422	7.442155697	0	0.63822568	0.010206361	0.005412934	0	7.87E-30	2.74E-27
TCONS_00026815	WBGene00172403	21ur-9368	NA	NA	NA	NA	1.775494844	7.454213879	0	5.948229797	0.005103181	0.006607179	0	4.60E-43	4.98E-40
TCONS_00027554	WBGene00172445	21ur-15606	NA	NA	NA	NA	0	0.707974041	0	1.160516326	0.005103181	1.254868721	0.47937239	8.38E-17	1.00E-14

TCONS_00048597	WBGene00172448	21ur-14553	NA	NA	NA	NA	0	0.016856525	0.487637405	0.006382257	0	0	1.403041141	1.18E-18	1.67E-16
TCONS_00038815	WBGene00172509	21ur-7042	NA	NA	NA	NA	2.334188347	7.855140555	1.252778192	6.82546485	0.478276428	0.956196098	0.438450356	6.32E-11	4.70E-09
TCONS_00048646	WBGene00172549	21ur-6153	NA	NA	NA	NA	0	0	0	0.011047424	0.005103181	0.005412934	1.408887146	6.52E-11	4.83E-09
TCONS_00048224	WBGene00172559	21ur-12843	NA	NA	NA	NA	0	0	0.487637405	0	0	0.633313279	1.403041141	2.07E-06	9.58E-05
TCONS_00027780	WBGene00172591	21ur-7625	NA	NA	NA	NA	0.959038594	0.83201455	0	0.72671873	0.776332753	1.406833835	0.507405744	1.49E-05	0.000650724
TCONS_00038885	WBGene00172593	21ur-7649	NA	NA	NA	NA	0	7.442155697	0.005605028	0.63822568	0	0	0.007008339	1.47E-29	4.54E-27
TCONS_00027585	WBGene00172612	21ur-12254	NA	NA	NA	NA	0.335349006	1.05255135	0.251010365	1.508737491	0.245033834	1.500125622	0.564634787	1.14E-07	6.33E-06
TCONS_00027559	WBGene00172643	21ur-6477	NA	NA	NA	NA	0	0.707974041	0.005605028	1.160516326	0.013863085	1.254868721	0.473526385	4.43E-16	5.00E-14
TCONS_00026831	WBGene00172652	21ur-13358	NA	NA	NA	NA	0.044082534	7.462642142	0.005605028	0.668419873	0	0.036060339	0.007008339	2.40E-31	9.24E-29
TCONS_00026779	WBGene00172659	21ur-12091	NA	NA	NA	NA	2.334188347	8.33746783	1.247173164	5.695805397	0.922253149	0.899119977	0.915017381	2.06E-06	9.58E-05
TCONS_00039787	WBGene00172692	21ur-9340	NA	NA	NA	NA	0	0	0.006881659	0	0	0.858933255	0	1.53E-10	1.10E-08
TCONS_00048242	WBGene00172694	21ur-11380	NA	NA	NA	NA	0	0	0	0	0	0	1.403041141	1.86E-15	1.81E-13
TCONS_00039950	WBGene00172732	21ur-6093	NA	NA	NA	NA	0.021000845	0.960962307	0.839757143	0.913893708	0.075865062	1.609981509	0.692084557	0.000139724	0.005493423
TCONS_00026868	WBGene00172741	21ur-7386	NA	NA	NA	NA	0	0	0.036183429	0.644607936	0.012034723	0.010825868	0	4.66E-05	0.001935336
TCONS_00027925	WBGene00172753	21ur-12625	NA	NA	NA	NA	0	0.825985459	0.728653594	0	0.556246697	0	0.007008339	0.000583541	0.02037395
TCONS_00027926	WBGene00172795	21ur-7360	NA	NA	NA	NA	0	0.825985459	0.728653594	0	0.556246697	0	0.007008339	0.000583541	0.02037395
TCONS_00048633	WBGene00172856	21ur-7506	NA	NA	NA	NA	0	0	0.487637405	0.011047424	0.005103181	0.005412934	1.415895484	5.50E-19	8.00E-17
TCONS_00038894	WBGene00172879	21ur-14180	NA	NA	NA	NA	0.007000282	7.467440485	0.005605028	0.680325842	0.017137904	0	0.040887698	2.84E-18	3.90E-16
TCONS_00036339	WBGene00172904	21ur-5658	NA	NA	NA	NA	0	0	0	0	0	0	1.403041141	1.86E-15	1.81E-13
TCONS_00038888	WBGene00172942	21ur-12973	NA	NA	NA	NA	0	7.442155697	0.005605028	0.643749391	0	0	0.007008339	1.34E-29	4.53E-27
TCONS_00036808	WBGene00172956	21ur-9288	NA	NA	NA	NA	0	0	0	0.016571135	0	0.005412934	1.408887146	2.46E-09	1.57E-07
TCONS_00036814	WBGene00172965	21ur-13695	NA	NA	NA	NA	0	0	0	0.011047424	0	0.012020113	1.41473315	5.09E-10	3.43E-08
TCONS_00026939	WBGene00172974	21ur-14090	NA	NA	NA	NA	1.292574579	9.431225623	0.068816591	6.740759131	0	0	0	6.27E-38	3.27E-35
TCONS_00048240	WBGene00172981	21ur-10268	NA	NA	NA	NA	0	0	0	0	0.006931542	0	1.403041141	9.35E-16	1.04E-13
TCONS_00036770	WBGene00172996	21ur-9010	NA	NA	NA	NA	0	0	0.487637405	0.006382257	0	0	1.403041141	2.82E-21	4.67E-19
TCONS_00036302	WBGene00173035	21ur-6687	NA	NA	NA	NA	0	0	0.005605028	0	0	0	1.403041141	1.00E-15	1.11E-13
TCONS_00026782	WBGene00173082	21ur-6694	NA	NA	NA	NA	2.334188347	8.33746783	1.247173164	5.695805397	0.922253149	0.899119977	0.915017381	2.06E-06	9.58E-05
TCONS_00005772	WBGene00173107	21ur-14256	NA	NA	NA	NA	0	0	0	0.552371179	0.006931542	0	0	1.12E-09	7.40E-08
TCONS_00036768	WBGene00173129	21ur-7425	NA	NA	NA	NA	0	0	0.487637405	0.006382257	0	0	1.403041141	2.82E-21	4.67E-19
TCONS_00027927	WBGene00173131	21ur-9597	NA	NA	NA	NA	0	0.825985459	0.728653594	0	0.556246697	0	0.007008339	0.000583541	0.02037395
TCONS_00027953	WBGene00173140	21ur-12027	NA	NA	NA	NA	0	0.825985459	0.728653594	0	0.556246697	0	0	0.000952431	0.031339626
TCONS_00039707	WBGene00173141	21ur-6391	NA	NA	NA	NA	0.184181402	0.974235492	0.362612416	1.340336841	0.191791761	1.292123304	0.50624341	1.61E-06	8.07E-05
TCONS_00027557	WBGene00173149	21ur-12435	NA	NA	NA	NA	0	0.707974041	0.005605028	1.160516326	0.006931542	1.254868721	0.473526385	2.06E-16	2.35E-14
TCONS_00040064	WBGene00173152	21ur-15131	NA	NA	NA	NA	0	0.825985459	0.728653594	0	0.556246697	0	0	0.000952431	0.031339626
TCONS_00027565	WBGene00173161	21ur-14234	NA	NA	NA	NA	0	0.707974041	0	1.160516326	0	1.254868721	0.473526385	3.51E-17	4.24E-15
TCONS_00048226	WBGene00173196	21ur-14634	NA	NA	NA	NA	0	0	0.487637405	0	0	0.633313279	1.403041141	2.07E-06	9.58E-05

TCONS_00026934	WBGene00173202	21ur-6810	NA	NA	NA	NA	1.292574579	9.431225623	0.068816591	6.740759131	0	0	0	6.27E-38	3.27E-35
TCONS_00039951	WBGene00173213	21ur-8177	NA	NA	NA	NA	0.021000845	0.960962307	0.839757143	0.913893708	0.075865062	1.609981509	0.692084557	0.000139724	0.005493423
TCONS_00048635	WBGene00173215	21ur-6126	NA	NA	NA	NA	0	0	0	0.011047424	0.012034723	0.005412934	1.408887146	3.15E-11	2.42E-09
TCONS_00018345	WBGene00173248	21ur-14745	NA	NA	NA	NA	3.089286432	2.562363651	0.034408295	2.693312368	1.347994052	2.603228482	1.710034616	1.10E-06	5.61E-05
TCONS_00039702	WBGene00173249	21ur-15611	NA	NA	NA	NA	0.076624224	0.832061131	0.119261839	1.2081402	0.086071424	1.293953503	0.499235072	2.31E-10	1.64E-08
TCONS_00038990	WBGene00173270	21ur-7377	NA	NA	NA	NA	0.432781667	9.521661987	0.169707088	6.805699025	0.122175593	0.0835825	0.028033354	1.88E-25	4.89E-23
TCONS_00048238	WBGene00173291	21ur-12325	NA	NA	NA	NA	0	0	0	0.006382257	0	0	1.403041141	5.71E-13	4.95E-11
TCONS_00038847	WBGene00173293	21ur-6196	NA	NA	NA	NA	1.775494844	7.454213879	0	5.948229797	0.005103181	0.019821537	0	1.37E-43	1.95E-40
TCONS_00001635	WBGene00173303	21ur-14797	NA	NA	NA	NA	0.007000282	0.028914707	0.013763318	0.046765329	0.017137904	1.087999735	0.021025016	8.69E-09	5.21E-07
TCONS_00026780	WBGene00173335	21ur-10421	NA	NA	NA	NA	2.334188347	8.33746783	1.247173164	5.695805397	0.922253149	0.899119977	0.915017381	2.06E-06	9.58E-05
TCONS_00040000	WBGene00173353	21ur-10885	NA	NA	NA	NA	0	0	0	0.011047424	0.776332753	0	0	3.01E-10	2.06E-08
TCONS_00026808	WBGene00173357	21ur-13117	NA	NA	NA	NA	1.817496534	7.442155697	0.013763318	5.949088342	0.005103181	0.017433047	0.042050032	2.41E-29	7.40E-27
TCONS_00048248	WBGene00173398	21ur-10752	NA	NA	NA	NA	0	0	0	0	0	0	1.403041141	1.86E-15	1.81E-13
TCONS_00039781	WBGene00173420	21ur-9898	NA	NA	NA	NA	0	0	0	0	0	0.858933255	0.014016677	2.61E-10	1.84E-08
TCONS_00038837	WBGene00173427	21ur-11743	NA	NA	NA	NA	1.604177379	7.425299173	0	5.918894148	0	0.005412934	0	8.42E-45	2.44E-41
TCONS_00048645	WBGene00173433	21ur-9173	NA	NA	NA	NA	0	0	0	0.011047424	0	0.005412934	1.408887146	1.35E-10	9.77E-09
TCONS_00036798	WBGene00173435	21ur-11464	NA	NA	NA	NA	0	0	0.011210055	0.011047424	0.012034723	0.005412934	1.415895484	9.30E-12	7.57E-10
TCONS_00048223	WBGene00173439	21ur-7098	NA	NA	NA	NA	0	0	0.487637405	0	0	0.633313279	1.403041141	2.07E-06	9.58E-05
TCONS_00036367	WBGene00173450	21ur-8635	NA	NA	NA	NA	0	0	0.494519064	0	0	0.633313279	1.410049479	1.86E-06	9.22E-05
TCONS_00026881	WBGene00173472	21ur-11947	NA	NA	NA	NA	0	0	0	0.63822568	0	0	0	9.75E-12	7.70E-10
TCONS_00027562	WBGene00173494	21ur-9409	NA	NA	NA	NA	0	0.707974041	0	1.160516326	0	1.254868721	0.473526385	3.51E-17	4.24E-15
TCONS_00027948	WBGene00173501	21ur-11329	NA	NA	NA	NA	0	0.825985459	0.728653594	0	0.556246697	0	0	0.000952431	0.031339626
TCONS_00027888	WBGene00173506	21ur-9879	NA	NA	NA	NA	0.024311548	0.008428262	0.058105037	0.076100978	0.888301985	0.070926432	0.126150095	3.04E-06	0.000139164
TCONS_00039688	WBGene00173522	21ur-14977	NA	NA	NA	NA	0	0.707974041	0	1.160516326	0	1.254868721	0.473526385	3.51E-17	4.24E-15
TCONS_00048129	WBGene00173581	21ur-8562	NA	NA	NA	NA	0	0	0.487637405	0	0	0.633313279	1.403041141	2.07E-06	9.58E-05
TCONS_00036809	WBGene00173582	21ur-9971	NA	NA	NA	NA	0	0	0	0.016571135	0	0.005412934	1.408887146	2.46E-09	1.57E-07
TCONS_00038882	WBGene00173598	21ur-11159	NA	NA	NA	NA	0.005770422	7.442155697	0.005605028	0.63822568	0	0	0.007008339	1.47E-29	4.54E-27
TCONS_00048243	WBGene00173627	21ur-5838	NA	NA	NA	NA	0	0	0	0	0	0	1.403041141	1.86E-15	1.81E-13
TCONS_00038861	WBGene00173664	21ur-14289	NA	NA	NA	NA	0.292496331	7.834965734	0.340192306	1.051014297	0.366827523	0.317377579	0.334178591	3.03E-06	0.000139164
TCONS_00039776	WBGene00173682	21ur-12107	NA	NA	NA	NA	0	0	0	0	0	0.865540434	0.007008339	2.82E-11	2.19E-09
TCONS_00040001	WBGene00173751	21ur-13565	NA	NA	NA	NA	0	0	0	0.011047424	0.776332753	0	0	3.01E-10	2.06E-08
TCONS_00036771	WBGene00173753	21ur-10125	NA	NA	NA	NA	0	0	0.487637405	0.006382257	0	0	1.403041141	2.82E-21	4.67E-19
TCONS_00038844	WBGene00173783	21ur-10559	NA	NA	NA	NA	1.775494844	7.483128586	0.027526636	5.942706085	0.039378988	0	0.056066709	5.20E-23	1.14E-20
TCONS_00036785	WBGene00173800	21ur-9323	NA	NA	NA	NA	0	0	0.487637405	0	0	0	1.403041141	7.09E-24	1.59E-21
TCONS_00030054	WBGene00173853	21ur-10898	NA	NA	NA	NA	0.019770986	0.051800322	0.072366857	0.012764514	0.012034723	3.989541803	0.007008339	3.78E-07	2.02E-05
TCONS_00039772	WBGene00173891	21ur-10426	NA	NA	NA	NA	0	0	0	0	0	0.870953368	0.005846005	2.28E-11	1.78E-09

TCONS_00038845	WBGene00173918	21ur-13901	NA	NA	NA	NA	1.775494844	7.483128586	0.027526636	5.942706085	0.039378988	0	0.056066709	5.20E-23	1.14E-20
TCONS_00026812	WBGene00173942	21ur-9153	NA	NA	NA	NA	1.775494844	7.442155697	0.019368346	5.949088342	0.034275808	0	0	2.93E-36	1.36E-33
TCONS_00048619	WBGene00173990	21ur-7326	NA	NA	NA	NA	0	0	0	0	0	0	1.403041141	1.86E-15	1.81E-13
TCONS_00048640	WBGene00174013	21ur-7522	NA	NA	NA	NA	0	0.006029091	0	0.01742968	0	0.005412934	1.408887146	4.24E-08	2.42E-06
TCONS_00027877	WBGene00174020	21ur-10486	NA	NA	NA	NA	0	0	0	0.011047424	0.776332753	0	0	3.01E-10	2.06E-08
TCONS_00036784	WBGene00174037	21ur-10514	NA	NA	NA	NA	0	0	0.487637405	0.006382257	0	0	1.403041141	2.82E-21	4.67E-19
TCONS_00027576	WBGene00174040	21ur-6448	NA	NA	NA	NA	0	0.707974041	0.006881659	1.160516326	0	1.254868721	0.473526385	1.01E-16	1.20E-14
TCONS_00048261	WBGene00174059	21ur-11302	NA	NA	NA	NA	0	0	0	0	0	0	1.403041141	1.86E-15	1.81E-13
TCONS_00026947	WBGene00174072	21ur-6090	NA	NA	NA	NA	1.292574579	9.431225623	0.068816591	6.740759131	0.005103181	0	0	2.47E-37	1.27E-34
TCONS_00038830	WBGene00174087	21ur-10658	NA	NA	NA	NA	1.604177379	7.425299173	0	5.918894148	0	0	0	1.60E-44	2.44E-41
TCONS_00038946	WBGene00174106	21ur-12146	NA	NA	NA	NA	0	0.020486444	0.011210055	0.670136963	0.025515904	0.005412934	0.01169201	8.18E-06	0.000368458
TCONS_00039998	WBGene00174135	21ur-7688	NA	NA	NA	NA	0	0	0	0.011047424	0.776332753	0	0	3.01E-10	2.06E-08
TCONS_00038881	WBGene00174181	21ur-7898	NA	NA	NA	NA	0	7.442155697	0.005605028	0.63822568	0	0	0.007008339	1.47E-29	4.54E-27
TCONS_00036335	WBGene00174216	21ur-11098	NA	NA	NA	NA	0.007000282	0	0.487637405	0	0	0.633313279	1.403041141	2.07E-06	9.58E-05
TCONS_00048647	WBGene00174234	21ur-8235	NA	NA	NA	NA	0	0	0	0.011047424	0	0.018627292	1.41473315	1.64E-09	1.06E-07
TCONS_00048631	WBGene00174252	21ur-13570	NA	NA	NA	NA	0.005770422	0	0	0.011047424	0.012034723	0.005412934	1.415895484	3.01E-11	2.32E-09
TCONS_00026940	WBGene00174266	21ur-13697	NA	NA	NA	NA	1.292574579	9.431225623	0.068816591	6.740759131	0	0	0	6.27E-38	3.27E-35
TCONS_00048610	WBGene00174288	21ur-6933	NA	NA	NA	NA	0	0	0.487637405	0.006382257	0	0.005412934	1.403041141	2.68E-20	4.16E-18
TCONS_00026800	WBGene00174289	21ur-11345	NA	NA	NA	NA	1.611177661	7.425299173	0.005605028	5.918894148	0	0.005412934	0.005846005	3.21E-40	2.54E-37
TCONS_00027954	WBGene00174313	21ur-6734	NA	NA	NA	NA	0	0.825985459	0.728653594	0	0.556246697	0	0	0.000952431	0.031339626
TCONS_00026841	WBGene00174337	21ur-8067	NA	NA	NA	NA	0	7.442155697	0	0.63822568	0	0	0	1.02E-33	4.08E-31
TCONS_00048370	WBGene00174351	21ur-11068	NA	NA	NA	NA	0	0	0	0	0	0	1.408887146	1.77E-15	1.81E-13
TCONS_00048232	WBGene00174362	21ur-8240	NA	NA	NA	NA	0	0	0.487637405	0	0	0.633313279	1.403041141	2.07E-06	9.58E-05
TCONS_00026830	WBGene00174401	21ur-5852	NA	NA	NA	NA	0.044082534	7.462642142	0.005605028	0.668419873	0	0.036060339	0.007008339	2.40E-31	9.24E-29
TCONS_00039907	WBGene00174405	21ur-5836	NA	NA	NA	NA	0	0.838043641	0	0.727577275	0.776332753	1.406833835	0.507405744	1.45E-05	0.000641695
TCONS_00039783	WBGene00174409	21ur-5929	NA	NA	NA	NA	0	0	0	0	0	0.858933255	0.007008339	3.05E-11	2.34E-09
TCONS_00048594	WBGene00174418	21ur-8074	NA	NA	NA	NA	0	0	0.487637405	0.006382257	0	0	1.403041141	2.82E-21	4.67E-19
TCONS_00026798	WBGene00174429	21ur-8306	NA	NA	NA	NA	1.604177379	7.425299173	0	5.918894148	0	0	0	1.60E-44	2.44E-41
TCONS_00039677	WBGene00174451	21ur-11361	NA	NA	NA	NA	0	0.707974041	0	1.160516326	0	1.254868721	0.473526385	3.51E-17	4.24E-15
TCONS_00036350	WBGene00174487	21ur-14606	NA	NA	NA	NA	0	0	0.487637405	0	0	0.633313279	1.403041141	2.07E-06	9.58E-05
TCONS_00026770	WBGene00174496	21ur-15374	NA	NA	NA	NA	0.4154704	0.321504719	0.026250005	1.940590674	0.025897808	0.030647405	0.01169201	5.80E-15	5.59E-13
TCONS_00026821	WBGene00174508	21ur-9238	NA	NA	NA	NA	1.775494844	7.454213879	0	5.948229797	0.005103181	0.019821537	0	1.37E-43	1.95E-40
TCONS_00048595	WBGene00174514	21ur-10236	NA	NA	NA	NA	0	0.016856525	0.487637405	0.006382257	0	0	1.403041141	1.18E-18	1.67E-16
TCONS_00027853	WBGene00174528	21ur-14171	NA	NA	NA	NA	0.098854928	0.066257676	0.116210075	0.073152902	0.894851623	0.036060339	0.078288394	1.92E-05	0.000829151
TCONS_00026834	WBGene00174541	21ur-13011	NA	NA	NA	NA	0.044082534	7.448184788	0.005605028	0.662037617	0	0.017433047	0.007008339	1.46E-30	5.35E-28
TCONS_00038869	WBGene00174554	21ur-10994	NA	NA	NA	NA	0.044082534	7.448184788	0.005605028	0.656513905	0	0.017433047	0.007008339	1.59E-30	5.79E-28

TCONS_00036803	WBGene00174584	21ur-11191		NA	NA	NA	NA	0	0.006029091	0	0.011047424	0	0.005412934	1.408887146	3.40E-09	2.13E-07
TCONS_00048230	WBGene00174600	21ur-9372		NA	NA	NA	NA	0	0	0.487637405	0	0	0.633313279	1.403041141	2.07E-06	9.58E-05
TCONS_00048255	WBGene00174605	21ur-14613		NA	NA	NA	NA	0	0	0	0	0	0	1.403041141	1.86E-15	1.81E-13
TCONS_00036813	WBGene00174617	21ur-6191		NA	NA	NA	NA	0	0	0	0.011047424	0	0.012020113	1.41473315	5.09E-10	3.43E-08
TCONS_00026783	WBGene00174620	21ur-10796		NA	NA	NA	NA	2.505033703	8.59966309	1.3300327	7.236391258	1.06530453	0.734979422	1.202633947	2.47E-05	0.001054236
TCONS_00027881	WBGene00174634	21ur-12951		NA	NA	NA	NA	0	0	0	0.011047424	0.776332753	0.006607179	0	7.37E-09	4.46E-07
TCONS_00026829	WBGene00174636	21ur-7532		NA	NA	NA	NA	0.462962811	8.204640856	0.822625816	1.267638586	0.708220303	0.610559604	0.518754398	0.0002048	0.007805905
TCONS_00027950	WBGene00174655	21ur-8972		NA	NA	NA	NA	0	0.825985459	0.728653594	0	0.556246697	0	0	0.000952431	0.031339626
TCONS_00030053	WBGene00174696	21ur-11320		NA	NA	NA	NA	0.021000845	0.068656847	0	0.036576451	0	3.964307333	0.014016677	1.15E-21	2.33E-19
TCONS_00048650	WBGene00174701	21ur-11333		NA	NA	NA	NA	0	0	0	0.011047424	0.013863085	0.005412934	1.408887146	3.02E-11	2.32E-09
TCONS_00048605	WBGene00174711	21ur-10557		NA	NA	NA	NA	0	0	0.487637405	0.006382257	0	0	1.403041141	2.82E-21	4.67E-19
TCONS_00036796	WBGene00174731	21ur-7335		NA	NA	NA	NA	0	0	0.487637405	0.011047424	0.005103181	0.005412934	1.415895484	5.50E-19	8.00E-17
TCONS_00036769	WBGene00174736	21ur-7230		NA	NA	NA	NA	0	0.006029091	0.487637405	0.006382257	0	0	1.403041141	6.17E-20	9.44E-18
TCONS_00036352	WBGene00174738	21ur-5602		NA	NA	NA	NA	0	0	0.487637405	0	0	0.633313279	1.403041141	2.07E-06	9.58E-05
TCONS_00027578	WBGene00174758	21ur-12565		NA	NA	NA	NA	0	0.707974041	0	1.160516326	0	1.254868721	0.473526385	3.51E-17	4.24E-15
TCONS_00030055	WBGene00174764	21ur-15560		NA	NA	NA	NA	0.019770986	0.051800322	0.072366857	0.012764514	0.005103181	3.989541803	0.007008339	6.44E-08	3.64E-06
TCONS_00038878	WBGene00174797	21ur-9509		NA	NA	NA	NA	0	7.448184788	0.005605028	0.643749391	0	0	0.007008339	1.33E-29	4.53E-27
TCONS_00036817	WBGene00174798	21ur-12234		NA	NA	NA	NA	0	0.006029091	0	0.011047424	0	0.005412934	1.408887146	3.40E-09	2.13E-07
TCONS_00038953	WBGene00174808	21ur-10784		NA	NA	NA	NA	0	0	0	0.63822568	0	0.006607179	0.007008339	7.21E-10	4.83E-08
TCONS_00038922	WBGene00174833	21ur-11453		NA	NA	NA	NA	0.007000282	7.442155697	0	0.644607936	0.012034723	0.019821537	0.012854343	4.60E-26	1.22E-23
TCONS_00038823	WBGene00174847	21ur-11489		NA	NA	NA	NA	2.145466383	8.04813379	0.961754503	6.656691758	0.784004205	0.590815731	0.682476867	2.22E-07	1.21E-05
TCONS_00038900	WBGene00174857	21ur-10727		NA	NA	NA	NA	0	7.442155697	0	0.63822568	0	0	0	1.02E-33	4.08E-31
TCONS_00038848	WBGene00174865	21ur-6813		NA	NA	NA	NA	1.775494844	7.454213879	0	5.948229797	0.005103181	0.006607179	0	4.60E-43	4.98E-40
TCONS_00038924	WBGene00174913	21ur-14781		NA	NA	NA	NA	0.286632675	7.724042925	0.316775191	0.935502452	0.265527718	0.309420827	0.382177634	2.01E-06	9.58E-05
TCONS_00038860	WBGene00174933	21ur-9616		NA	NA	NA	NA	0.325323662	7.831460463	0.018091714	6.279393724	0.630445719	0.213492916	0.019862682	1.95E-23	4.34E-21
TCONS_00039790	WBGene00174944	21ur-10061		NA	NA	NA	NA	0	0.825985459	1.128592085	0.702048247	0.006931542	1.406833835	0	4.45E-11	3.37E-09
TCONS_00026879	WBGene00174953	21ur-13173		NA	NA	NA	NA	0	0	0	0.63822568	0	0	0.007008339	1.44E-09	9.42E-08
TCONS_00027665	WBGene00174955	21ur-12284		NA	NA	NA	NA	0	0	0	0	0.005103181	0.865540434	0.012854343	5.45E-09	3.37E-07
TCONS_00036431	WBGene00174956	21ur-9057		NA	NA	NA	NA	0	0.008428262	0	0	0	0	1.403041141	6.10E-13	5.24E-11
TCONS_00048649	WBGene00174997	21ur-10744		NA	NA	NA	NA	0	0.006029091	0	0.011047424	0	0.005412934	1.408887146	3.40E-09	2.13E-07
TCONS_00048627	WBGene00175011	21ur-9147		NA	NA	NA	NA	0	0	0.006881659	0	0	0.012020113	1.421741489	2.38E-13	2.12E-11
TCONS_00022408	WBGene00185075	C03C10.9	III	1	135	1	135	2.490299145	0.429109231	0.660736639	0.769854013	0.922935797	0.442991642	3.407980422	0.001259985	0.040425586
TCONS_00053107	WBGene00189948	F56H9.8	V	1	435	1	435	0.068772958	0.036174546	0.019368346	0.016571135	0.049967254	1.133691697	0.01169201	2.10E-05	0.000902175
TCONS_00053106	WBGene00189948	F56H9.8	V	61	346	150	435	0.045312394	0.036174546	0.005605028	0.011047424	0.018966266	1.133691697	0.014016677	4.85E-09	3.02E-07
TCONS_00017880	WBGene00189950	C39B5.14	III	90	1079	1	990	0.835673771	0.122918666	0	0.16668356	0.013863085	0.119642839	0.129705767	0.000158425	0.006163884
TCONS_00017881	WBGene00189950	C39B5.14	III	88	1077	1	990	0.835673771	0.122918666	0	0.16668356	0.013863085	0.119642839	0.129705767	0.000158425	0.006163884

TCONS_00037534	WBGene00189987	C18H7.12	IV	640	1543	126	1029	0	17.13467646	0	0.054006131	0.080204434	0.119565174	0	5.01E-13	4.36E-11
TCONS_00039672	WBGene00189995	C06E7.88	IV	18	596	1	579	1.747692063	4.324695804	1.73764346	5.621934025	1.465863635	5.502109597	2.363801568	3.01E-05	0.00127003
TCONS_00006661	WBGene00194656	T05F1.15	I	45	263	1	219	0	0	1.741388465	0.016571135	0.005103181	0	0.007008339	1.26E-10	9.22E-09
TCONS_00065460	WBGene00194795	F42F12.14	X	1	224	12348565	12348788	3.224547218	15.40160082	1.646102995	60.72941279	1.389802889	1.753043261	1.887062866	4.35E-11	3.30E-09
TCONS_00039000	WBGene00194829	mir-2217	IV	1	62	5280136	5280075	1.292574579	9.431225623	0.068816591	6.740759131	0	0	0	6.27E-38	3.27E-35
TCONS_00011442	WBGene00194935	M02G9.4	II	6	185	1	180	0.092326756	7.619540203	0.058603539	0.099426812	0.146545783	0.039084783	0	6.43E-08	3.64E-06
TCONS_00011441	WBGene00194935	M02G9.4	II	86	264	10317400	10317578	0.046163378	5.066835563	0.02930177	0.049713406	0.085307615	0.02945316	0	3.81E-08	2.19E-06
TCONS_00005681	WBGene00194992	H27M09.6	I	1	165	170	6	0.007000282	0	0	0.029335649	0.869717624	0	0.035041693	2.73E-10	1.92E-08
TCONS_00060983	WBGene00195060	linc-14	V	1	255	1	255	2.517891701	0.452119496	3.265627261	2.214749646	4.130998634	0.459463436	2.318803753	1.01E-07	5.61E-06
TCONS_00060984	WBGene00195060	linc-14	V	1	187	39	225	2.517891701	0.452119496	3.265627261	2.214749646	4.130998634	0.459463436	2.318803753	1.01E-07	5.61E-06
TCONS_00001101	WBGene00195069	W03G9.10	I	1	309	1	309	0.069245067	0.10003305	0.122313605	0.071808253	0.076929615	2.729312985	0.039725364	0.00030451	0.011313042
TCONS_00067685	WBGene00195081	C04F6.7	X	163	1506	1	1344	0.047393237	1.186048151	3.02332196	0.331422707	2.726607188	0.260782085	11.76997873	9.98E-15	9.56E-13
TCONS_00011455	WBGene00195392	C07E3.13	II	1	137	1	137	2.573608314	0	1.472675038	1.052213826	3.776353718	0	0	4.77E-11	3.59E-09
TCONS_00022417	WBGene00195511	C03C10.10	III	1	148	1	148	2.114085075	0	0	0	0	0	2.771006253	8.54E-19	1.24E-16
TCONS_00026799	WBGene00195531	C02B10.71	IV	1	110	1	110	1.604177379	7.425299173	0.006881659	5.929941571	0	0	0	1.03E-42	1.04E-39
TCONS_00052292	WBGene00195547	T28B11.2	V	1	143	1	143	0.380847867	0	1.479556697	0.957338519	1.300132003	0.005412934	0.925100694	2.72E-14	2.56E-12
TCONS_00067696	WBGene00195727	ZK377.4	X	1	196	1	196	0	0.134852198	0.357846271	0	0	0	1.128278917	8.69E-10	5.78E-08
TCONS_00058540	WBGene00195733	T28B11.3	V	1	150	1	150	0.380847867	0	1.471398406	0.957338519	1.305235184	0	0.918092355	1.30E-14	1.24E-12
TCONS_00017721	WBGene00195737	Y82E9BR.27	III	1	217	1	217	0.007000282	0.564693581	0.005605028	0.01914677	0.552695033	0.005412934	0.950059074	3.92E-05	0.001641836
TCONS_00058535	WBGene00195748	T28B11.4	V	1	284	286	3	0.410929838	0.008428262	1.471398406	0.982867546	1.312166726	0.024040226	0.930946699	5.60E-13	4.86E-11
TCONS_00039791	WBGene00195753	B0350.69	IV	1	168	1	168	0	0.825985459	1.128592085	0.702048248	0	0.005412934	0	0.000992788	0.032563445
TCONS_00041390	WBGene00195807	F56H11.8	IV	1	143	1	143	0.019770986	0.016856525	0.019368346	0.028477104	0.833646816	0.017433047	0.007008339	1.05E-05	0.000470151
TCONS_00058545	WBGene00195832	T28B11.5	V	1	217	1	217	0.401848712	0.016856525	1.485161724	0.950956262	1.300132003	0.006607179	0.918092355	1.19E-13	1.08E-11
TCONS_00037553	WBGene00195864	F41A4.3	IV	1	90	1	90	3.066123387	2.447810923	0	0	0	0	0	5.22E-18	7.03E-16
TCONS_00052289	WBGene00195881	T28B11.6	V	1	127	1	127	0.380847867	0	1.465793379	0.957338519	1.310338365	0.02706467	0.925100694	3.58E-13	3.16E-11
TCONS_00030056	WBGene00195927	F54D1.17	IV	1	147	1	147	0	0	0	0.006382257	0	3.975133201	0	1.52E-20	2.43E-18
TCONS_00011450	WBGene00196173	C07E3.14	II	1	148	1	148	2.573608314	0	1.472675038	1.057737538	3.776353718	0.028258915	0	4.10E-08	2.35E-06
TCONS_00058537	WBGene00196176	T28B11.7	V	1	298	1	298	0.380847867	0.006029091	1.472675038	0.963720776	1.312166726	0.030647405	0.918092355	8.27E-13	7.08E-11
TCONS_00022272	WBGene00196329	C48D5.4	III	1	149	1	149	0.478945045	2.038985418	1.132215585	1.583607483	0	0.845718898	0.609725457	4.75E-06	0.000215891
TCONS_00022271	WBGene00196486	C48D5.5	III	1	150	1	150	0.478945045	2.038985418	1.132215585	1.58998974	0	0.837917474	0.609725457	5.02E-06	0.0002281
TCONS_00022416	WBGene00196490	C03C10.11	III	1	141	1	141	2.114085075	0	0	0.006382257	0.005103181	0	2.771006253	2.53E-16	2.87E-14
TCONS_00058544	WBGene00196501	T28B11.8	V	1	346	1	346	0.387848149	0.025284787	1.465793379	0.968385943	1.317269907	0.006607179	0.925100694	2.52E-13	2.24E-11
TCONS_00026789	WBGene00196535	C02B10.72	IV	1	118	1	118	2.145466383	8.042104699	0.961754503	6.656691758	0.789107386	0.59742291	0.689485205	2.31E-07	1.25E-05
TCONS_00027879	WBGene00196539	M03D4.75	IV	1	64	1	64	0	0	0	0.011047424	0.776332753	0	0	3.01E-10	2.06E-08
TCONS_00042094	WBGene00196590	B0035.19	IV	1	144	1	144	0	0.022885616	0.020644977	0.023811937	0.039760893	4.031015076	0.012854343	3.15E-08	1.82E-06
TCONS_00058541	WBGene00196660	T28B11.9	V	1	142	1	142	0.386618289	0	1.465793379	0.957338519	1.317269907	0.010825868	0.925100694	5.06E-14	4.70E-12

TCONS_00005594	WBGene00196721	F57B10.17	I	1	206	1	206	0.040771831	0.089143292	0.074421618	0.060388388	0.080968243	1.135444233	0.045571369	0.000763235	0.026203358
TCONS_00052288	WBGene00196735	T28B11.10	V	1	284	1	284	0.380847867	0	1.471398406	0.957338519	1.305235184	0.005412934	0.925100694	2.68E-14	2.53E-12
TCONS_00062694	WBGene00196772	ZK813.9	X	1	50	1	50	0	0.134852198	0.369056326	0.06076083	0.330885675	0	1.458833165	3.95E-12	3.26E-10
TCONS_00027929	WBGene00196792	C11D2.99	IV	1	253	1	253	0	0.825985459	0.728653594	0	0.561349877	0	0	0.000925679	0.031029854
TCONS_00039792	WBGene00196826	B0350.72	IV	1	102	1	102	0	0.006029091	1.128592085	0.012764514	0	0	0	5.08E-09	3.15E-07
TCONS_00038801	WBGene00196900	C02B10.73	IV	1	148	1	148	0.409699978	0.307047366	0.005605028	1.883150363	0.006931542	0.019821537	0	2.77E-21	4.67E-19
TCONS_00030099	WBGene00196972	F22B3.13	IV	1	83	1	83	0.005770422	0	0	0.005523712	0	3.964307333	0	1.58E-20	2.51E-18
TCONS_00030382	WBGene00196985	F35G2.12	IV	1	2155	12216202	12218356	0.507045345	0.050569574	0.070591724	0.247077263	0.724293654	0.039084783	1.119764889	1.68E-07	9.25E-06
TCONS_00022415	WBGene00197172	C03C10.12	III	1	146	1	146	2.114085075	0	0	0.006382257	0	0.006607179	2.771006253	4.58E-15	4.43E-13
TCONS_00022270	WBGene00197446	C48D5.6	III	1	147	1	147	0	1.187730916	1.132215585	0.931809492	0	0.83250454	0.609725458	0.000493902	0.01758735
TCONS_00027783	WBGene00197469	H16O14.77	IV	1	99	1	99	0.959038594	0.840442812	0.006881659	0.721195018	0.776332753	1.406833835	0.507405744	2.04E-05	0.000878052
TCONS_00058538	WBGene00197493	T28B11.11	V	1	137	1	137	0.386618289	0	1.465793379	0.963720776	1.310338365	0.018627292	0.960142387	1.05E-13	9.49E-12
TCONS_00054808	WBGene00197676	Y59A8B.29		NA	NA	NA	NA	0.007000282	0	0.879989341	1.082843376	0.005103181	1.340592076	0	1.53E-07	8.46E-06
TCONS_00062705	WBGene00197750	ZK377.5	X	1	145	1	145	0.296564785	0.639628817	0.912865513	0.006382257	0.526797225	0.29732305	1.860839146	1.56E-08	9.25E-07
TCONS_00052287	WBGene00197769	T28B11.12	V	1	195	1	195	0.380847867	0	1.483885093	0.982867546	1.305235184	0.006607179	0.929784365	3.69E-14	3.45E-12
TCONS_00037853	WBGene00198080	Y38F2AL.8	IV	1	99	99	1	0.691133513	0.307483638	0.030578401	0.568020855	0	0.376609197	0.405321304	2.82E-07	1.52E-05
TCONS_00038912	WBGene00198105	C24D10.47	IV	1	143	1	143	0.045312394	7.508413373	0.03746006	0.750903109	0.096277785	0.039643073	0.114492421	5.74E-10	3.86E-08
TCONS_00029560	WBGene00198130	F25H8.11	IV	1	132	1	132	0	0.012058182	0	0	0	0	1.625934553	1.02E-11	8.06E-10
TCONS_00029415	WBGene00198132	F56H11.11	IV	1	143	1	143	0.019770986	0.03371305	0.019368346	0.028477104	0.828543635	0.017433047	0.007008339	0.000124576	0.004964243
TCONS_00062712	WBGene00198199	C12D12.9	X	1	191	243	53	0	0.134852198	0.357846271	0	0.005103181	0	1.122432913	4.88E-10	3.31E-08
TCONS_00038028	WBGene00198247	Y67D8C.17	IV	1	150	1	150	1.862074934	1.314341826	3.015516531	2.862697392	0.006931543	2.673955661	1.282525962	0.000836494	0.028433899
TCONS_00016029	WBGene00198282	M02G9.6	II	1	49	1	49	0.005770422	2.514130923	0	0	0	0	0	3.85E-18	5.22E-16
TCONS_00062708	WBGene00198450	ZK377.6	X	1	136	1	136	0	0.134852198	0.363451298	0	0.013863085	0.037254584	1.135287256	6.92E-09	4.23E-07
TCONS_00020353	WBGene00198459	R10E11.11	III	1	150	1	150	0.042852675	0.768202626	0.030578401	0.054006131	0.025515904	0.041473273	0.045571369	6.64E-05	0.002716077
TCONS_00041527	WBGene00198674	F25H8.13	IV	1	347	1	347	0	0.012058182	0	0	0	0	1.625934553	1.02E-11	8.06E-10
TCONS_00067695	WBGene00198761	ZK377.7	X	1	148	1	148	0	0.169795996	0.363451298	0	0.006931542	0.019821537	1.122432913	3.33E-08	1.92E-06
TCONS_00058539	WBGene00198793	T28B11.13	V	1	144	1	144	0.380847867	0.006029091	1.471398406	0.963720776	1.305235184	0.006607179	0.94612571	5.02E-14	4.68E-12
TCONS_00052285	WBGene00198804	T28B11.14	V	1	567	1	567	0.380847867	0.006029091	1.493320015	0.987532713	1.346824439	0.03184165	0.932109033	7.47E-13	6.41E-11
TCONS_00052291	WBGene00198878	T28B11.15	V	1	121	1	121	0.380847867	0	1.478280065	0.950956262	1.307063546	0.005412934	0.92393836	2.39E-14	2.27E-12
TCONS_00067698	WBGene00198912	ZK377.8	X	1	136	1	136	0	0.134852198	0.363451298	0	0	0	1.122432913	7.64E-10	5.11E-08
TCONS_00041528	WBGene00198914	F25H8.15	IV	1	196	1	196	0	0.012058182	0	0.006382257	0	0	1.625934553	3.83E-10	2.61E-08
TCONS_00027876	WBGene00199126	M03D4.76	IV	1	144	1	144	0.011540844	0	0	0.01742968	0.776332753	0	0.007008339	8.00E-10	5.33E-08
TCONS_00012397	WBGene00199162	ZK131.12	II	1	50	1	50	0.729538859	0.412003408	0.720896437	1.41274486	6.452969753	1.908872483	3.108048731	0.000707145	0.024379323
TCONS_00039784	WBGene00199242	K02B2.35	IV	1	150	1	150	0	0.008428262	0	0	0	0.869759123	0.007008339	1.26E-11	9.87E-10
TCONS_00067682	WBGene00199313	C04F6.8	X	1	123	1	123	0.005770422	0.134852198	0.357846271	0.038665983	0.318850952	0	1.458833165	3.91E-13	3.43E-11
TCONS_00022269	WBGene00199419	C48D5.7	III	1	105	1	105	0	1.187730916	1.132215585	0.931809492	0.006931543	0.83250454	0.609725458	0.000610644	0.021284126

TCONS_00011436	WBGene00199470	ZK970.10	II	1	86	1	86	0	2.514130923	0	0	0	0	0	3.85E-18	5.22E-16
TCONS_00067705	WBGene00199515	F56B6.7	X	1	145	1	145	0.021000845	0.134852198	0.390977935	0.01914677	0	0.006607179	1.142295595	8.33E-08	4.66E-06
TCONS_00018294	WBGene00199522	T24A11.6	III	1	150	1	150	0.26563183	0.299974501	4.396845841	0.284998362	0.381535578	1.473279302	2.010265177	0.000232549	0.00880649
TCONS_00022307	WBGene00199625	F34D10.10	III	1	81	1	81	0.975201357	4.619513758	4.013807323	2.674165597	2.422936818	3.78205385	0	0.000219109	0.008335876
TCONS_00022419	WBGene00199655	C30D11.2	III	1	143	1	143	2.170087329	0.055430242	0.044341719	0.040383073	0.144798582	0.05651783	2.817739956	7.55E-08	4.23E-06
TCONS_00068531	WBGene00199678	T10E10.12	X	1	189	1	189	8.084123414	4.311874635	1.016406643	0.505719511	0.301087662	0.026428716	0	0.000416079	0.014971558
TCONS_00058543	WBGene00199795	T28B11.16	V	1	364	1	364	0.380847867	0	1.465793379	0.957338519	1.317269907	0.037254584	0.918092355	8.54E-13	7.28E-11
TCONS_00041529	WBGene00199877	F25H8.17	IV	1	140	1	140	0	0.012058182	0	0.006382257	0	0	1.625934553	3.83E-10	2.61E-08
TCONS_00042132	WBGene00199957	H02I12.11	IV	1	93	93	1	0	0	0	0	0	4.017164764	0	2.68E-20	4.16E-18
TCONS_00012400	WBGene00200004	ZK131.13	II	1	50	1	50	0.765391252	0.42406159	0.715291409	0.854540441	20.24209362	1.431340045	3.172354783	2.97E-07	1.60E-05
TCONS_00067701	WBGene00200042	C12D12.10	X	1	259	1	259	0	0.134852198	0.357846271	0	0.005103181	0	1.122432913	4.88E-10	3.31E-08
TCONS_00027666	WBGene00200059	K02B2.36	IV	1	135	1	135	0	0	0	0.011905969	0.005103181	0.858933255	0.026871021	1.88E-08	1.11E-06
TCONS_00004013	WBGene00200067	Y54E5B.7		NA	NA	NA	NA	0.011540844	0	0.022420111	2.771988981	0	0	0	4.75E-11	3.58E-09
TCONS_00027878	WBGene00200103	M03D4.77	IV	1	181	1	181	0	0	0.006881659	0.011047424	0.776332753	0	0	1.62E-10	1.16E-08
TCONS_00029558	WBGene00200109	F25H8.18	IV	1	286	1	286	0	0.012058182	0	0.012764514	0	0	1.625934553	7.75E-09	4.68E-07
TCONS_00018295	WBGene00200134	T24A11.7	III	1	149	1	149	0	0	4.013807323	0	0	1.185432547	1.689009601	7.28E-08	4.09E-06
TCONS_00005633	WBGene00200159	T23B3.7	I	1	57	1	57	0	0	0	1.314643405	0.85257972	0	1.841491498	0.000331385	0.012190485
TCONS_00022319	WBGene00200225	T24A11.8	III	1	120	1	120	0	0	4.008202295	0	0.005103181	1.185432547	1.696017939	3.88E-08	2.22E-06
TCONS_00011456	WBGene00200273	C07E3.15	II	1	70	1	70	2.573608314	0	1.472675038	1.058596083	3.788388441	0	0	4.95E-11	3.72E-09
TCONS_00052284	WBGene00200347	T28B11.18	V	1	351	1	351	0.410929838	0.008428262	1.471398406	0.99563206	1.317269907	0.024040226	0.936792704	5.94E-13	5.14E-11
TCONS_00022267	WBGene00200413	C48D5.8	III	1	59	1	59	0.511486735	2.096814831	1.214017364	1.62656619	0.022241085	1.933640969	1.833830783	8.37E-05	0.003398042
TCONS_00042049	WBGene00200538	C08F8.13	IV	1	138	1	138	0.012770704	0.014457353	0.02930177	0.01742968	0.013863085	3.982934624	0.007008339	1.48E-09	9.66E-08
TCONS_00026784	WBGene00200553	C02B10.74	IV	1	101	1	101	2.157007227	8.050532961	1.00354296	6.699650466	0.816833555	0.614855957	0.726885901	4.02E-07	2.13E-05
TCONS_00001693	WBGene00200700	F08B6.6	I	1	59	1	59	0.011540845	0.024116364	0.05250001	1.36055019	0.89378707	1.084975292	0.043246701	0.000473196	0.016908527
TCONS_00029559	WBGene00200727	F25H8.22	IV	1	205	1	205	0	0.012058182	0	0	0	0.010825868	1.625934553	1.57E-10	1.13E-08
TCONS_00039788	WBGene00200800	K02B2.37	IV	1	209	1	209	0	0	0.011210055	0	0.005103181	0.869759123	0	7.82E-08	4.38E-06
TCONS_00058546	WBGene00200830	T28B11.20	V	1	279	1	279	0.387848149	0.012058182	1.471398406	0.950956262	1.307063546	0.005412934	0.92393836	7.90E-14	7.20E-12
TCONS_00037541	WBGene00200850	C18H7.16	IV	1	74	620555	620482	0.08324563	2.500841994	0.077971885	0.129248564	0.138931593	0.05890632	0.063075048	0.00031832	0.011741322
TCONS_00062707	WBGene00200880	ZK377.11	X	1	111	1	111	0	0.134852198	0.357846271	0	0.013863085	0.006607179	1.122432913	5.46E-10	3.67E-08
TCONS_00052290	WBGene00201002	T28B11.21	V	1	292	1	292	0.380847867	0	1.465793379	0.957338519	1.305235184	0.028258915	0.918092355	4.27E-13	3.74E-11
TCONS_00022420	WBGene00201040	C30D11.4	III	1	150	1	150	2.198088455	0.075916686	0.058105037	0.048482419	0.100316413	0.048716406	2.843448642	3.88E-06	0.000177213
TCONS_00063096	WBGene00201047	F43C9.7	X	1	116	1	116	0.377537164	0.203758344	0.041788456	0.076587082	0.074036701	1.143677902	0.239651859	0.000178888	0.00686918
TCONS_00053738	WBGene00201155	VF23B12L.1	V	1	133	1	133	0	0.03371305	0.006881659	0.005523712	0	1.179102286	0.005846005	1.30E-12	1.09E-10
TCONS_00058536	WBGene00201401	T28B11.22	V	1	349	351	3	0.380847867	0	1.493320015	0.981150456	1.346824439	0.025234471	0.932109033	2.15E-13	1.93E-11
TCONS_00011415	WBGene00201428	F33H1.9	II	1	89	1	89	2.573608314	2.514130923	1.472675038	1.046690114	3.783285261	0.005412934	4.918706583	1.90E-06	9.41E-05
TCONS_00030095	WBGene00201441	F22B3.19	IV	1	147	1	147	0	0	0	0	0	3.964307333	0	3.12E-20	4.81E-18

TCONS_00018256	WBGene00201466	C32A3.6	III	1	154	1	154	0.025541408	0.022885616	1.183438963	0.041241618	0.031000989	0.050468942	0.021025016	0.001140325	0.036758282
TCONS_00026919	WBGene00201467	F36H12.65	IV	1	81	1	81	0.432781667	9.521661987	0.169707088	6.805699025	0.122175593	0.078169566	0.028033354	2.79E-25	7.18E-23
TCONS_00001729	WBGene00201494	H27M09.7	I	1	175	1	175	0.007000282	0.008428262	0	0.029335649	0.869717624	0	0.035041693	4.01E-09	2.50E-07
TCONS_00027670	WBGene00201592	B0350.81	IV	1	157	1	157	0	0.825985459	1.128592085	0.702048248	0	0.005412934	0	0.000992788	0.032563445
TCONS_00067697	WBGene00201762	ZK377.12	X	1	213	1	213	0	0.134852198	0.357846271	0	0	0	1.122432913	8.94E-10	5.92E-08
TCONS_00022421	WBGene00201819	C30D11.5		NA	NA	NA	NA	2.133856061	0.128885432	0.136076923	0.132682745	0.122557498	0.167645626	2.85746532	0.001127069	0.036359473
TCONS_00062696	WBGene00201845	ZK813.10	X	1	140	1	140	0	0.134852198	0.357846271	0	0	0	1.122432913	8.94E-10	5.92E-08
TCONS_00058542	WBGene00201874	T28B11.23	V	1	129	1	129	0.380847867	0	1.465793379	0.957338519	1.310338365	0.02706467	0.925100694	3.58E-13	3.16E-11
TCONS_00052286	WBGene00201919	T28B11.24	V	1	144	1	144	0.380847867	0.006029091	1.471398406	0.963720776	1.305235184	0.006607179	0.94612571	5.02E-14	4.68E-12
TCONS_00041526	WBGene00201929	F25H8.25	IV	1	145	1	145	0	0	0	0	0.005103181	0	1.625934553	2.07E-16	2.35E-14
TCONS_00005531	WBGene00202057	C10G11.16	I	1	150	663	514	3.585437631	2.013716375	1.669689887	1.040307858	0.018966266	2.237323564	0	1.71E-14	1.63E-12
TCONS_00052293	WBGene00202074	T28B11.25	V	1	269	1	269	0.387848149	0	1.465793379	0.962003686	1.32420145	0.006607179	0.918092355	2.85E-14	2.68E-12
TCONS_00018273	WBGene00202195	T27D1.4	III	1	150	1	150	0	0	1.132215585	0	0.005103181	0	0	6.13E-14	5.66E-12
TCONS_00060001	WBGene00202249	F23B12.11	V	1	148	1	148	0	0.025284787	0.006881659	0.005523712	0.006931542	1.161669239	0.007008339	1.03E-10	7.51E-09
TCONS_00021845	WBGene00202323	Y82E9BR.30	III	1	145	145	1	0.007000282	0.556265318	0.005605028	0.012764514	0.526797225	0.005412934	0.950059074	2.83E-05	0.001201119
TCONS_00062709	WBGene00202370	ZK377.13	X	1	113	1	113	0	0.140881289	0.381543013	0.006382257	0	0	1.122432913	5.09E-09	3.16E-07
TCONS_00062706	WBGene00202397	ZK377.14	X	1	277	1	277	0	0.134852198	0.357846271	0	0	0.005412934	1.134124922	1.56E-09	1.01E-07
TCONS_00020212	WBGene00206421	T26G10.8	III	1	252	1	252	0	0	1.14342564	0	0	0	0.007008339	5.27E-14	4.87E-12
TCONS_00024173	WBGene00206422	T26G10.9	III	1	252	1	252	0	0	1.14342564	0	0	0	0.007008339	5.27E-14	4.87E-12
TCONS_00048653	WBGene00219241	Y116A8A.150	IV	185	673	16829382	16828894	0.168857745	0.05666099	0.213550305	0.370915778	0.298958556	0.129832752	1.730177058	9.30E-05	0.003744979
TCONS_00048654	WBGene00219241	Y116A8A.150	IV	185	673	16829382	16828894	0.168857745	0.05666099	0.213550305	0.370915778	0.298958556	0.129832752	1.730177058	9.30E-05	0.003744979
TCONS_00056262	WBGene00219256	mir-4922.1	V	1	65	3380209	3380145	0.55187969	0.086806445	0.924829562	1.13364265	0.412674989	0.033035894	0.57129977	0.000106866	0.004283387
TCONS_00005773	WBGene00219281	K10D3.8	I	1	177	1	177	0.017311267	0	0.011210055	1.12769575	0.015309542	0.02706467	0	4.64E-10	3.16E-08
TCONS_00018399	WBGene00219299	C01G12.16	III	1	300	1	300	4.254941417	0	0.005605028	0.045906784	0.010206361	0.021651736	8.367923134	8.16E-10	5.44E-08
TCONS_00030277	WBGene00219327	ZK822.9	IV	1	180	1	180	0.844096319	0.034943798	0.589269416	0.142871623	0.089728147	0.05468763	0.345973608	3.22E-05	0.001353429
TCONS_00030276	WBGene00219327	ZK822.9	IV	1	105	1	105	1.028656596	0.04700198	1.152288827	0.190495497	0.103591232	0.065513498	0.413766661	7.44E-06	0.000336299
TCONS_00030059	WBGene00219550	B0035.20	IV	1	57	1	57	0.111246757	0.066257676	0.097340231	0.076959523	0.07731152	8.009172721	0.065468386	7.30E-05	0.002983955
TCONS_00061474	WBGene00219562	linc-28	V	1	114	1	114	0.081543662	0.341196688	0.033131664	0.318513075	0.422499445	0.631405415	0.149534114	0.0005369	0.018954521
TCONS_00010998	WBGene00219590	linc-72	II	1	343	1	343	0.611099413	0.183209575	0.222207098	0.397076027	1.438600531	0.257277014	0.824149328	0.000627265	0.02182648
TCONS_00048618	WBGene00219633	anr-55	IV	1	1132	1	1132	0	0.006029091	0.487637405	0.006382257	0	0	1.403041141	6.17E-20	9.44E-18
TCONS_00011432	WBGene00219642	anr-52	II	1	479	1	479	0.154006198	9.037715576	0.005605028	3.865480925	7.63291187	3.837770211	0.01169201	1.65E-17	2.19E-15
TCONS_00038908	WBGene00219653	linc-145	IV	1	902	1	902	0	7.442155697	0	0.643749391	0	0	0	9.32E-34	4.08E-31
TCONS_00014221	WBGene00219672	anr-54	II	1	1275	1	1275	1.163742793	2.159800791	0.208723407	1.125574761	1.947496043	3.420489211	0.80886731	0.000133175	0.005261056
TCONS_00018389	WBGene00219843	C30D11.6	III	1	171	1	171	2.170087329	0.055430242	0.044341719	0.028477104	0.124003955	0.048716406	2.817739956	2.36E-08	1.37E-06
TCONS_00018342	WBGene00219850	C36E8.11	III	1	183	1	183	3.103286995	2.579220175	0.041289954	2.730747363	1.368406775	2.620661529	1.708872282	1.20E-06	6.10E-05
TCONS_00005661	WBGene00219882	F08B6.7	I	1	161	1	161	0	0	0.018091714	0.018288225	0.8576829	0.012020113	0.025708687	1.67E-09	1.08E-07

TCONS_00067183	WBGene00219885	F09E10.14	X	1	115	1	115	12.67977748	20.98452264	41.15615681	18.65167511	54.71742675	13.30772723	24.79227559	0.001106585	0.035839084
TCONS_00007987	WBGene00219910	F21F12.2	I	1	94	1	94	0.226561968	2.222724426	2.739690913	5.363961581	0.159344316	0.301697067	0.210318829	0.000674098	0.023318124
TCONS_00005680	WBGene00219993	H27M09.8	I	1	162	165	4	0.007000282	0	0	0.029335649	0.869717624	0	0.035041693	2.73E-10	1.92E-08
TCONS_00001720	WBGene00220000	K02F2.11	I	1	126	1	126	0.03131183	0.04337206	0.048171614	0.030194194	0.866442805	0.036060339	0.021025016	0.000368248	0.013367326
TCONS_00005771	WBGene00220018	K10D3.9	I	1	96	1	96	0.044082534	0.026515535	0.033131664	0.605518765	0.025897808	0.090747969	0.033879359	1.25E-05	0.00056069
TCONS_00008373	WBGene00220026	M01D1.11	II	1	113	1	113	1.325116269	2.795783945	5.654438451	2.291230189	4.721812419	0.049910651	5.568088733	2.33E-08	1.36E-06
TCONS_00022330	WBGene00220044	R13G10.5	III	1	68	1	68	3.089286432	5.807244674	2.460607137	3.535864636	2.428039998	3.810312765	0.035041693	2.18E-07	1.19E-05
TCONS_00000359	WBGene00220136	Y20F4.11	I	1	92	1	92	0.005770422	0.006029091	0	0	0.006931542	0.562945137	0	3.18E-09	2.02E-07
TCONS_00048305	WBGene00220176	Y51H4A.940	IV	1	153	1	153	0.005770422	0	0.013763318	0	0	0.644139147	0	4.36E-08	2.49E-06
TCONS_00007986	WBGene00220241	ZK270.5	I	1	130	1	130	0	2.064924287	2.580415751	5.115167227	0.032447446	0.079286147	0	1.24E-07	6.86E-06
TCONS_00051473	WBGene00235318	K11D5.2	V	1	146	8427131	8427276	0.119098023	0.197729253	0.011210055	0.586681522	0.395918989	0.902236727	0.209259502	2.25E-05	0.000961852

Supplementary Table 5 List of all *C. elegans* strains.

Strain	Genotype	Gene mutated/ silenced	Non avoidance condition		Avoidance condition	
			**P value relative to OP50 fed worm**	**Figure in text**	**P value relative to OP50 fed worm (Figure in text)**	**Figure in text**
BA15	*rrf-3(hc15)*	*rrf-15*	< 0.0001	Fig. 1	< 0.0001	Supplementary Fig. 1
N2	N2**	*C. elegans* wild type	< 0.0001	Fig. 1	< 0.0001	Supplementary Fig. 1
		**P value relative to N2 exposed to *F. oxysporum***				
VC199	*sir-2.1(ok434)IV.*	*sir-2.1*	< 0.0001	Fig. 8a	nd#	
CB5414	*srd-1(eh1)II*	*srd-1*	< 0.0001	Fig. 8a	nd#	
RB1000	*gcy-5(ok921)II.*	*gcy-5*	0.0006	Fig. 8a	nd#	
TJ356	zls356IV. (daf-16:GFP rollerstrain)			Fig. 8b	nd#	
VP303	*rde-1 (ne219)* V; kbls 7	*rde-1*	< 0.0001	Fig. 8c	nd#	

Effect of silencing *daf-16*

Genotype	P value relative to N2 grown on dsRNA of vector control and exposed to *F. oxysporum*			
N2;daf-16 RNAi**	*daf-16*	< 0.0001	Fig. 6a	
**P value relative to N2 grown on dsRNA of daf-16 and exposed to *F. oxysporum***				
pmk-1(km25)IV.;daf-16 RNAi	*pmk-1 & daf-16*	ns*	Fig. 6c	nd#
pmk-3(ok169);daf-16 RNAi	*pmk-3 & daf-16*	ns* (Fig. 26B)	Fig. 6c	nd#
jnk-1(gk7)IV.;daf-16 RNAi	*jnk-1 & daf-16*	ns*(Fig. 26B)	Fig. 6c	nd#
nsy-1(ag3)II.;daf-16 RNAi	*nsy-1 & daf-16*	0.0024 (Fig. 26B)	Fig. 6c	nd#
sek-1(km4)X.;daf-16 RNAi	*sek-1 & daf-16*	0.0016 (Fig. 26B)	Fig. 6c	nd#
sma-6(ok2894)II.;daf-16 RNAi	*sma-6 & daf-16*	ns*	Fig. 6d	nd#
sma-9(ok1628)X.;daf-16 RNAi	*sma-9 & daf-16*	ns*	Fig. 6d	nd#
daf-3(ok3610)X.;daf-16 RNAi	*daf-3 & daf-16*	ns*	Fig. 6d	nd#
mek-2(q484)I;sDP2(I;f);daf-16 RNAi	*mek-2 & daf-16*	< 0.0001	Fig. 6a	nd#
tol-1(nr2033)I.;daf-16 RNAi	*tol-1 & daf-16*	0.0002	Fig. 6b	nd#
trf-1(nr2014)III.;daf-16 RNAi	*trf-1 & daf-16*	0.0702	Fig. 6b	nd#
ikb-1(nr2027)I.;daf-16 RNAi	*ikb-1 & daf-16*	< 0.0001	Fig. 6b	nd#
sir-2.1(ok434)IV.;daf-16 RNAi	*sir-2.1 & daf-16*	ns*	Fig. 8a	nd#
srd-1(eh1)II;daf-16 RNAi	*srd-1 & daf-16*	0.0007	Fig. 8a	nd#
gcy-5(ok921)II.;daf-16 RNAi	*gcy-5 & daf-16*	ns*	Fig. 8a	nd#
VP303;*daf-16* RNAi	*rde-1 (ne219) V; kbls 7 & daf-16*	< 0.0001	Fig. 8c	

*ns= not significant

**the strain has been used as a control

#nd= not done

Supplementary Table 6 Information of library construction for high-throughput sequencing.

Sample	Treatment	Type of Reads	PE/SE	Reads Length
BA15 (B1)	0 hour uninfected	RNA-seq	PE	101
BA15 (B2)	6 hour uninfected	RNA-seq	PE	101
BA15 (B3)	6hpi	RNA-seq	PE	101
BA15 (B4)	24 hour uninfected	RNA-seq	PE	101
BA15 (B5)	24hpi	RNA-seq	PE	101
BA15 (B6)	48 hour uninfected	RNA-seq	PE	101
BA15 (B7)	48hpi	RNA-seq	PE	101

Supplementary Table 7 Pearson correlation coefficients for expression levels in control and infected samples

Control		0 hour	6 hour	24 hour	48 hour
	**0 hour**	1	0.7974659	0.584818	0.8307319
	**6 hour**	0.7974659	1	0.8480388	0.931159
	**24 hour**	0.584818	0.8480388	1	0.8337594
	**48 hour**	0.8307319	0.931159	0.8337594	1

Infected		6hpi	24hpi	48hpi
	**6hpi**	1	0.8036873	0.876311
	**24hpi**	0.8036873	1	0.7989672
	**48hpi**	0.876311	0.7989672	1

Supplementary Table 8 List of primers for qRT-PCR.

Gene	Forward Primer (5'-3')	Reverse Primer (5'-3')
act-1	CCATCATGAAGTGCGACATTG	CATGGTTGATGGGGCAAGAG
Y82E9BR.27	GCCGAAATGCGCGAAA	TTCCTCGCTAATTTGTTTGATGATT
T22F3.11	CATCCCAGCCGAGAACAAGT	TGACACCCACACAGCACATAGTC
R13G10.4	TGCTAGTCTCTGGTTCGTGACAGT	AAGAATCTGCCCAATACAAGTGAAG
C17F4.7	CGAACTGGAAATCCGCATGT	TGCCATAACACAGGCAATCTCT
dos-3	CCAACACCTCGACCATTCATC	GGTCCATGTTTCCGTCACAAT
kqt-1	CAAGATCACATTCGCCTGGTT	TCATCGTCATCATCTGCATCTATTT
gcy-5	TGATGGAATGACTGGAAGAGTTCA	CCACTTGCTCGTATTTTTCATCTAAA
srb-6	CTGAACAATTGCGTTGTATTTGC	GGATTCACTAACAAATCACACTTGGA
